# Platinum(IV) Complexes with Tridentate, *NNC*-Coordinating Ligands: Synthesis, Structures, and Luminescence

**DOI:** 10.1021/acs.inorgchem.2c04116

**Published:** 2023-01-16

**Authors:** Yana M. Dikova, Dmitry S. Yufit, J. A. Gareth Williams

**Affiliations:** Department of Chemistry, Durham University, DurhamDH1 3LE, United Kingdom

## Abstract

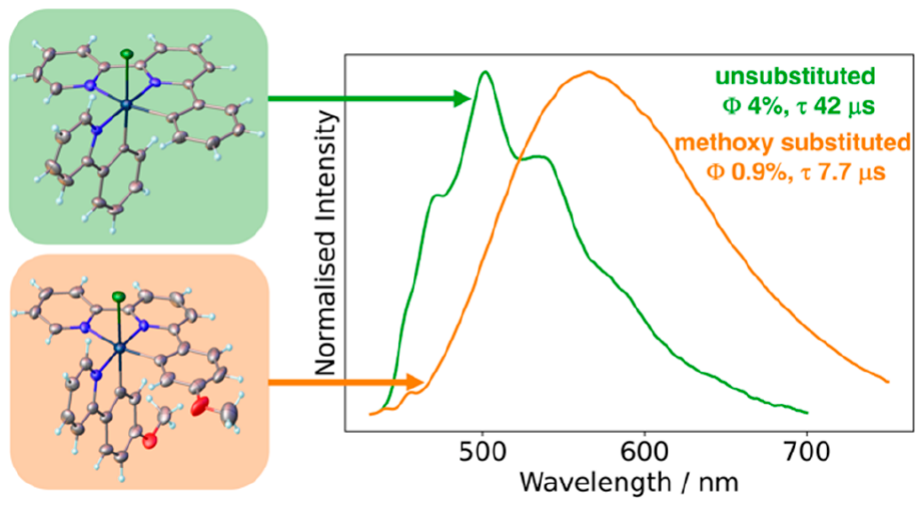

Platinum(II) complexes of *NNC*-cyclometalating ligands based on 6-phenyl-2,2′-bipyridine (HL^1^) have been widely investigated for their luminescence properties. We describe how PtL^1^Cl and five analogues with differently substituted aryl rings, PtL^2–6^Cl, can be oxidized with chlorine and/or iodobenzene dichloride to generate Pt(IV) compounds of the form Pt(*NNC*-L^*n*^)Cl_3_ (*n* = 1–6). The molecular structures of several of them have been determined by X-ray diffraction. These PtL^*n*^Cl_3_ compounds react with 2-arylpyridines to give a new class of Pt(IV) complex of the form [Pt(*NNC*)(*NC*)Cl]^+^. Elevated temperatures are required, and the reaction is accompanied by competitive reduction processes and generation of side-products; however, four examples of such complexes have been isolated and their molecular structures determined. Reaction of PtL^1^Cl_3_ with HL^1^ similarly generates [Pt(*NNC*-L^1^)_2_]^2+^, which we believe to be the first example of a bis-tridentate Pt(IV) complex. The lowest-energy bands in the UV–vis absorption spectra of all the PtL^*n*^Cl_3_ compounds are displaced to higher energy relative to the Pt(II) precursors, but they red-shift with the electron richness of the aryl ring, consistent with predominantly ^1^[π_Ar_ → π*_NN_] character to the pertinent excited state. A similar trend is observed for the [Pt(*NNC*)(*NC*)Cl]^+^ complexes. They display phosphorescence in solution at room temperature, centered around 500 nm for [PtL^1^(ppy)Cl]^+^ and [Pt(L^1^)_2_]^2+^, and 550 nm for methoxy-substituted derivatives. The lifetimes are in the microsecond range, rising to hundreds of microseconds at 77 K, consistent with triplet excited states of primarily ^3^[π_Ar_ → π*_NN_] character with relatively little participation of the metal.

## Abstract 2

There are few known platinum(IV) complexes with tridentate ligands, in contrast to the well-established chemistry of other d^6^ metal ions with such ligands. We describe the synthesis and solid-state structures of several cyclometalated Pt(IV) complexes featuring tridentate, *NNC*-coordinating ligands: 6-phenylbipyridine and derivatives. When the coordination sphere is completed by a second bi- or tridentate cyclometalating ligand, room-temperature phosphorescence is triggered, with the emission energy being influenced by the electron richness of the aryl ring.

## Introduction

The +2-oxidation state predominates in most research into platinum complexes and their applications. The excited-state properties of Pt(II) complexes have been widely explored over the past 30 years, often focusing on applications such as OLED devices,^1^ bioimaging,^2^ chemosensors,^3^ energy conversion,^4^ singlet oxygen generation,^5^ and photodynamic therapy.^6^ The d^8^ configuration of the Pt(II) ion leads to square-planar complexes, sometimes with different properties compared to the pseudo-octahedral complexes formed by d^6^ metal ions such Ir(III), Os(II), Re(I), Rh(III), and Ru(II). The +4 oxidation state of platinum also has the d^6^ electronic configuration: it is isoelectronic with Ir(III). However, Pt(IV) complexes have been much less investigated. Part of the reason may be the kinetic inertness of Pt(IV), rendering the synthesis of its complexes often difficult. Moreover, the lower energy of its 5d orbitals is expected to lead to rather inefficient mixing with ligand orbitals, whereas efficient mixing underpins many of the applications mentioned above, such as the relaxation of the spin-selection rule facilitating phosphorescence that is exploited in OLEDs.^7^

**Figure 1:**
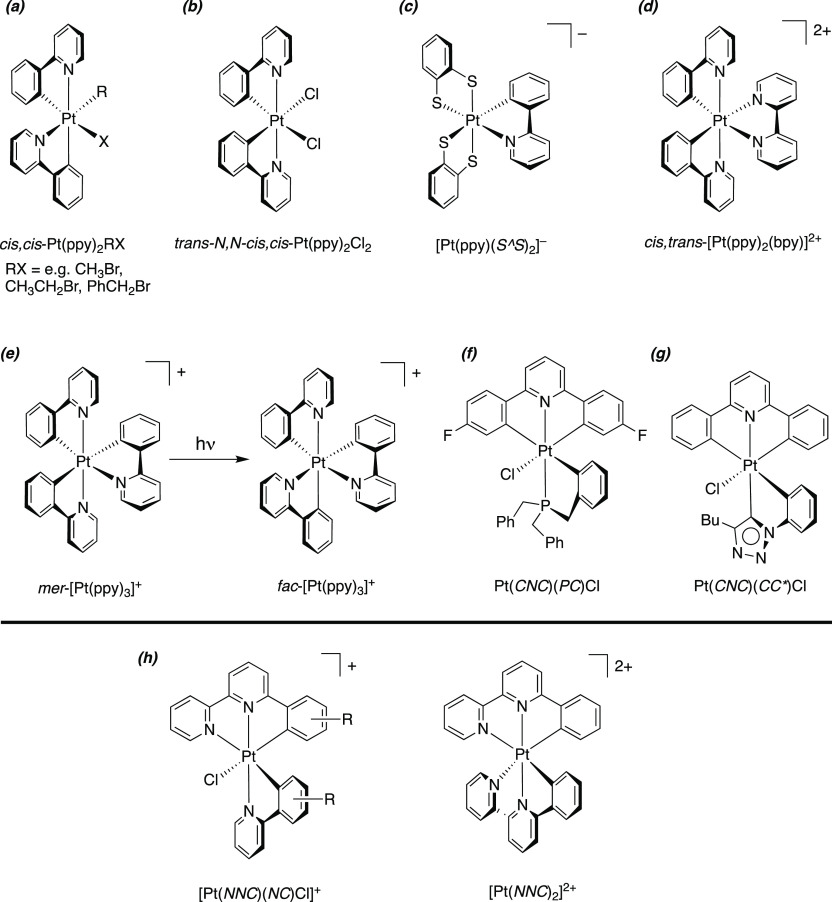
Examples of Pt(IV) complexes with cyclometalating ligands. (a) Pt(*NC*)_2_RX complexes from oxidative addition of alkyl halides to *cis*-Pt(*NC*)_2_.^8^ (b) A variety of isomeric complexes of the form Pt(*NC*)_2_Cl_2_ can be prepared by oxidation of Pt(II) analogues, of which one is shown.^10^ (c) Example of an anionic, mono-cyclometalated complex, where *S*^*S* = 1,2-benzendithiolate.^13^ (d) Parent structure of the type [Pt(*NC*)_2_(NN)]^2+^.^14^ (e) Photochemical conversion of the meridional to facial isomer of [Pt(ppy)_3_]^+^.^15^ (f) CNC-coordinated Pt(IV) complex that undergoes reductive coupling to a CNP-coordinated Pt(II) complex.^20^ (g) Pt(IV) complex incorporating a CNC-coordinating tridentate ligand and a carbene coligand.^21^ (h) Generic structures of the two new types of Pt(IV) complex investigated in this work, containing *NNC*-coordinating ligands.

Nevertheless, there has been steady interest in cyclometalated Pt(IV) complexes since at least the 1980s. Von Zelewsky^8^ and, later, Swager^9^ explored oxidation addition of alkyl halides (RX) to Pt(*NC*)_2_ complexes (where *NC* represents a bidentate cyclometalating ligand such as 2-phenylpyridine) to generate a variety of 6-coordinate Pt(IV) products of the form Pt(*NC*)_2_RX (Figure 1a). Rourke and co-workers have carried out a series of fascinating studies into the synthesis, isomerism, reactivity, and interconversion of such materials (Figure 1b),^10^ while related themes have been explored by Whitfield and Sanford.^11^ Bruce and colleagues explored luminescent, liquid crystalline derivatives of Pt(*NC*)_2_Cl_2_, featuring dodecyl pendants on the *NC* ring,^12^ while Ionescu et al. reported an interesting series of anionic, mono-cyclometalated complexes of the form [Pt(*NC*)(*S^S*)]^−^ (where *S^S* represents 1,2-benzene-dithiolate, Figure 1c).^13^ In the context of photophysical properties and applications in light-emitting electrochemical cells (LEECs), Jenkins and Bernhard prepared a family of complexes of the type [Pt(*NC*)_2_(*NN*)]^2+^, where *NN* is a bidentate ligand based on 2,2′-bipyridine (the parent example is shown in Figure 1d).^14^ Some of them were luminescent in solution at room temperature, with lifetimes as long as 260 μs. Low radiative rate constants *k*_r_ were attributed to the minimal participation of the metal in the excited states (reflecting the low energy of the 5d orbitals mentioned above); the emissive states were formulated as ^3^ILCT. Superior performance was subsequently reported by González-Herrero and co-workers, who have pioneered elegant research into tris-cyclometalated Pt(IV) complexes and related systems over the past decade. For example, they have prepared a range of *fac* and *mer* isomers of homoleptic [Pt(*NC*)_3_]^+^ (Figure 1e) and heteroleptic [Pt(*NC*)_2_(*N′C′*)]^+^ complexes and investigated their photophysical properties.^15^ Despite the similarly low *k*_r_ values, the strong ligand field in the *fac* isomers ensures that nonradiative decay processes are minimized, and high quantum yields are observed as a result. We also note earlier work (albeit on a non-cyclometalated complex), by Kunkely and Vogler, who reported room-temperature emission from PtMe_3_I(bpy), assigned to the ^3^π–π* state of the bipyridine ligand.^16^

There are very few reported Pt(IV) complexes with tridentate ligands. Van Koten and colleagues described how a Pt(II) “pincer” complex of the type Pt^II^(*NCN*)Cl (where *NCN* represents a para-substituted 2,6-bis(dimethylaminomethylene)benzene) underwent oxidation with CuCl_2_ to generate Pt^IV^(*NCN*)Cl_3_.^17^ Recently, the group of Connick has described the properties of [Pt^IV^(tpy)Cl_3_]^+^ complexes,^18^ while Gabbiani and co-workers reported a related Pt(IV) terpyridyl complex in the context of photo-cytotoxic agents, with two acetate coligands and one chloride.^19^ Rourke and colleagues described the preparation and reactivity of a *CNC*-coordinated Pt(IV) complex (Figure 1f), which can undergo a reductive coupling reaction to generate an extraordinary square-planar Pt(II) complex featuring *CNP* coordination and a 9-membered chelate ring.^20^ González-Herrero and co-workers also studied a *CNC*-coordinated Pt(IV) complex, in their case containing a bidentate N-heterocyclic carbene ligand (CC*), of the form Pt^IV^(*CNC*)(CC*)Cl (Figure 1g).^21^ This complex was only weakly luminescent in solution at room temperature, perhaps associated in part with the mutually *trans* disposition of the metalated rings of the *CNC* ligand, a feature that similarly compromises the emission of *mer*-[Pt(*NC*)_3_]^+^ and *mer*-[Ir(*NC*)_3_] complexes.^22,23^

To our knowledge, there are no known examples of bis-tridentate Pt(IV) complexes, neither with cyclometalated ligands nor with more classical ligands such as diethylenetriamine or terpyridine. Such complexes are very well-established for most other d^6^ metal ions and offer structural advantages over bis- and tris-bidentate analogues in many instances.^24^ The lack of examples of Pt(IV) coordinated by two tridentate ligands is probably another reflection of its kinetic inertness.

Here, we describe our studies into the synthesis and photophysical properties of Pt(IV) complexes incorporating a tridentate, *NNC*-coordinating ligand, namely, 6-phenyl-2,2-bipyridine or a derivative thereof. The products have the form [Pt(*NNC*)(*NC*)Cl]^+^ or [Pt(*NNC*)_2_]^2+^ (Figure 1h) and are prepared via the intermediacy of Pt(*NNC*)Cl_3_. We believe [Pt(*NNC*)_2_]^2+^ to be the first reported example of a bis-tridentate Pt(IV) complex. The cationic complexes are found to be moderately luminescent in deoxygenated solution at room temperature, with lifetimes in the microsecond range, and we compare and contrast their photophysical properties with those of the Pt(II) analogues.

## Results and Discussion

Numerous studies have focused on Pt(II) complexes of the form Pt(*NNC*)Cl since Constable et al. described Pt(*NNC*-phbpy)Cl just over 30 years ago (phbpy = 6-phenyl-2,2′-bipyridine).^25^ Metathesis of the chloride ligand offers rich diversity.^26^ In particular, Che and co-workers have demonstrated how stronger σ donors like acetylides promote phosphorescence from ^3^MLCT states that may be formulated as ^3^[d_Pt_|π_NNC_ → π*_NNC_].^27^ Potential applications of such complexes include as OLED emitters and chemosensors.^28,29^ There is also an extensive chemistry of di- and trinuclear platinum(II) compounds with such ligands in combination with polytopic bridging ligands including bis- and tris-phosphines and xanthene-bis-acetylides.^30−32^ However, there are no Pt(IV) complexes of *NNC*-coordinating ligands, to our knowledge.

### Synthesis of Pt(*NNC*)Cl_3_ Complexes

**Scheme 1:**
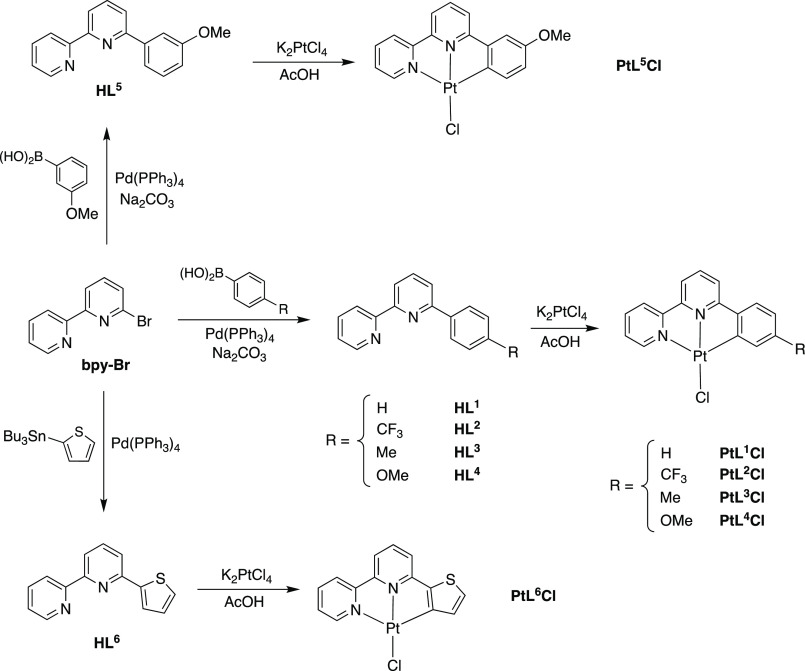
Synthetic Routes to the *NNC* Proligands Used in This Work and Thence the Pt(II) Complexes of the Form Pt(*NNC*)Cl

Six *NNC* proligands, HL^1^–HL^6^, have been examined during this work, as illustrated in Scheme 1. Proligands HL^1^–HL^5^ were prepared by Suzuki coupling of 6-bromo-2,2′-bipyridine (**bpy-Br**) with the corresponding aryl boronic acid, as described recently for HL^2^ and HL^4^.^33^ The other HL^*n*^ compounds had also been reported previously, albeit prepared via methodology such as Krohnke condensation (HL^1^, HL^3^, HL^6^)^34^ or reaction of 2,2′-bipyridine with an aryl lithium (HL^5^).^35^ HL^6^ was prepared by Stille coupling of **bpy-Br** with 2-(tributyltin)thiophene. The Pt(II) complexes were then prepared by reaction of the requisite proligand with K_2_PtCl_4_ in refluxing acetic acid.

**Figure 2:**
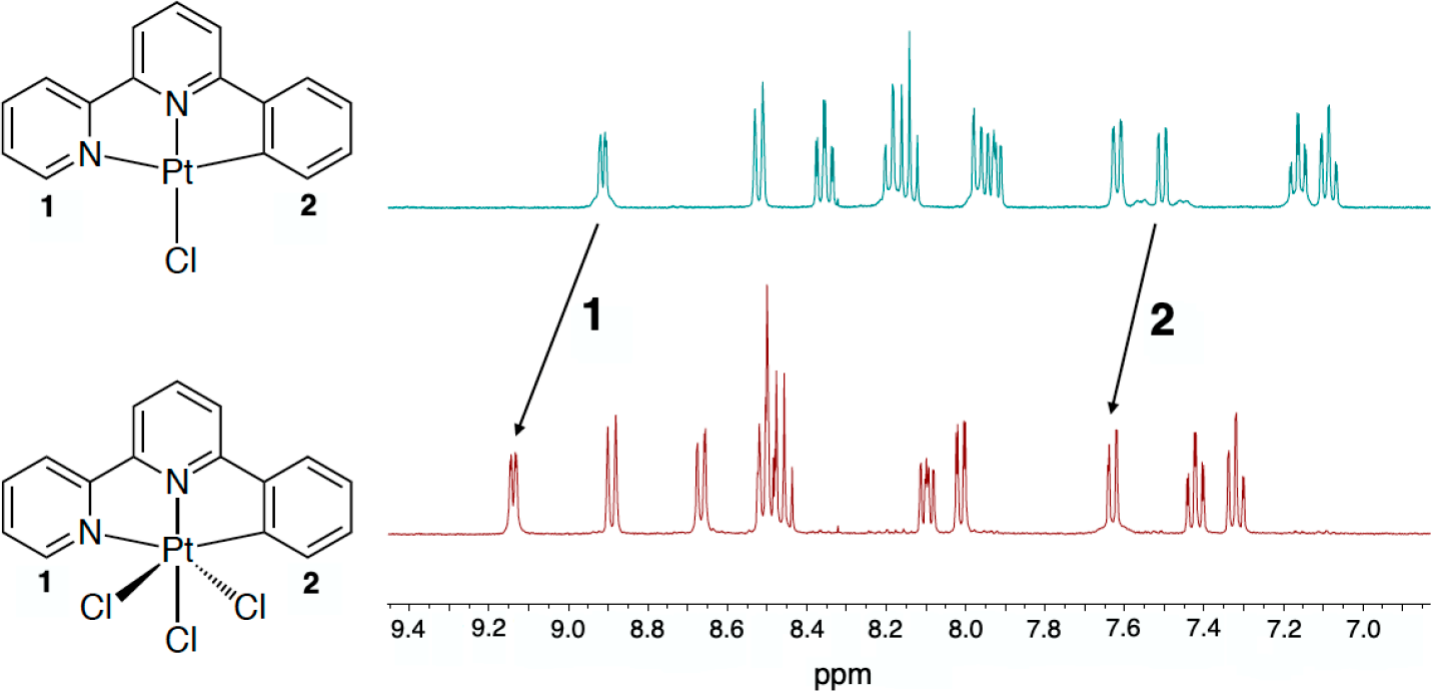
^1^H NMR spectra of PtL^1^Cl and PtL^1^Cl_3_ in *d*_6_-DMSO at 400 MHz. The arrows show the shifts of the protons *ortho* to the metal-coordinated N and C atoms of the lateral rings, labeled **1** and **2**, respectively.

**Scheme 2:**
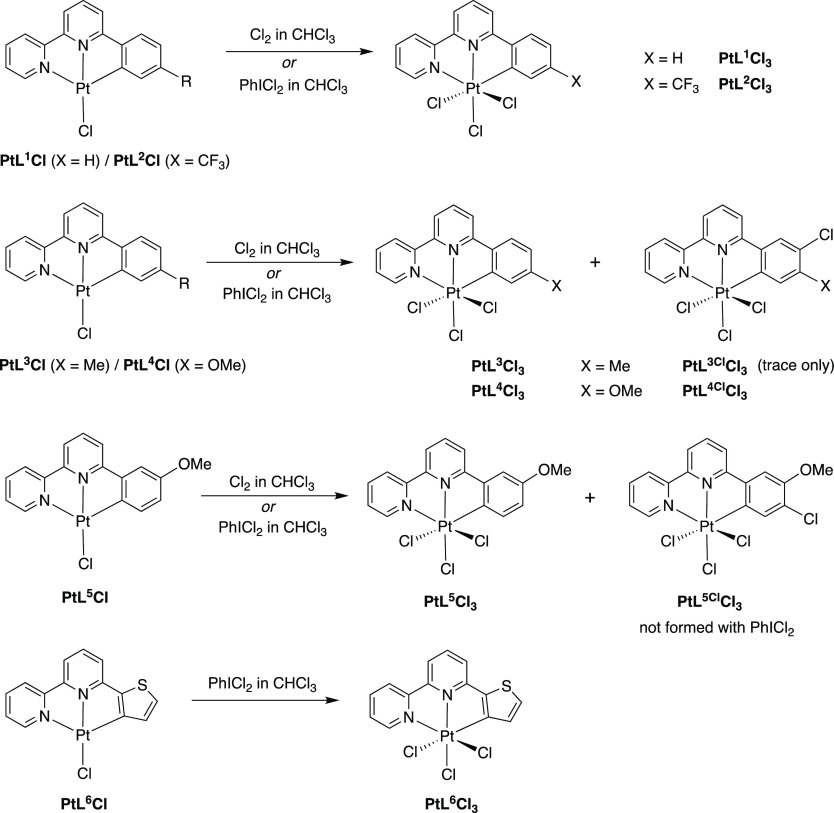
Oxidation of the PtL^*n*^Cl Precursors to the Corresponding PtL^*n*^Cl_3_ Complexes Using Cl_2_ or PhICl_2_, Showing Those Instances in Which Side-Products Containing a Partially Chlorinated Phenyl Ring Are Formed, Denoted PtL^*n*Cl^Cl_3_

The oxidation of PtL^1^Cl to PtL^1^Cl_3_ was achieved by bubbling chlorine gas through a solution of the Pt(II) complex in chloroform at room temperature (Scheme 2; see the Experimental Details section for details). Oxidation occurs within a few minutes and is visually apparent from the solution becoming paler in color. The Pt(IV) product is pale yellow compared to the more vibrant yellow-orange hue of the Pt(II) precursor. The ^1^H NMR spectrum shows deshielding of all protons upon oxidation, with a downfield shift of each set of resonances by around 0.3–0.4 ppm (Figure 2). The ^195^Pt–^1^H coupling constant for the proton *ortho* to the Pt–C bond is notably smaller in the Pt(IV) product compared to the Pt(II) precursor (around 27 and 43 Hz, respectively); that for the proton *ortho* to the Pt–N is also diminished upon oxidation, to the extent that it is not readily resolved at 600 MHz. A reduction in the magnitude of coupling constants to ^195^Pt is quite typical upon oxidation from Pt(II) to Pt(IV) in other systems, rationalized in terms of the lower s contribution in the d^2^sp^3^-hybridized Pt(IV) relative to the dsp^2^ Pt(II) center.^36,37^ Coupling to ^195^Pt is anticipated also in the ^13^C spectrum, at least for the carbon atom bound to the metal and neighboring ones, but the spectra of the Pt(IV) compounds in this study were generally too weak to unequivocally assign ^195^Pt satellite peaks (the most convincing case is PtL^2^Cl_3_, shown in the Supporting Information Figure S4.41, for which a ^1^*J*^195^Pt–^13^C coupling of 550 Hz is evident). The identity of PtL^1^Cl_3_ was confirmed by X-ray crystallography (Supporting Information Figure S2.1, and as described in the next section).

The CF_3_-substituted complex PtL^2^Cl reacted with Cl_2_ in the same way as PtL^1^Cl, to give PtL^2^Cl_3_ as the sole product. The corresponding reaction of PtL^3^Cl likewise led to PtL^3^Cl_3_ (Scheme 2). In this instance, however, ^1^H NMR spectroscopy showed that the product was accompanied by a small proportion of a second, related complex. X-ray diffraction analysis of a crystal of PtL^3^Cl_3_ subsequently showed that the desired product had cocrystallized with a complex (which we shall refer to as PtL^3Cl^Cl_3_) incorporating a chlorine atom in the metalated phenyl ring, at the position *para* to the C–Pt bond (Scheme 2, Figure S2.2). The use of shorter reaction times failed to give a pure sample of the non-phenyl-chlorinated product, suggesting that the rate of chlorination of the phenyl ring may be competitive with Pt(II) oxidation (*vide infra*). Benzene itself does undergo electrophilic substitution with Cl_2_ at room temperature, but only in the presence of a Lewis acid catalyst such as AlCl_3_. The activation of the phenyl ring in the complex to electrophilic chlorination is to be anticipated, since cyclometalation of aryl heterocycles increases the electron density in the aryl ring. Indeed, a variety of cyclometalated iridium(III) complexes with 2-phenylpyridine or related tridentate ligands have been shown to undergo facile electrophilic bromination with NBS at room temperature, invariably at the position *para* to the C–Ir bond.^38^ The fact that the formation of neither PtL^1^Cl_3_ nor PtL^2^Cl_3_ (with its electron-withdrawing CF_3_ group) was accompanied by such a product, in contrast to PtL^3^Cl_3_, is evidently an indication that the mildly electron-donating influence of the methyl substituent promotes the electrophilic substitution. [We note that *prolonged* treatment of PtL^1^Cl with Cl_2_ did show evidence of side reactions becoming significant, although the *para*-chlorinated product was not unequivocally identified.] The complex PtL^5^Cl showed significant chlorination of the phenyl ring, in line with the electron-donating nature of the methoxy substituent. X-ray diffraction analysis of PtL^5Cl^Cl_3_ revealed that the chlorine atom is introduced into the position *meta* to the C–Pt bond (i.e., *para* to the pyridine). The thienyl complex PtL^6^Cl gave an intractable mixture of products when the same conditions were employed, suggesting that the thienyl ring—already more electron-rich than phenyl—becomes too reactive toward Cl_2_ upon cyclometalation.

The oxidizing agent iodobenzene dichloride, PhICl_2_, was investigated by Whitfield and Sanford in an inspiring study of the oxidative chemistry of *cis*-Pt(ppy)_2_.^11^ They obtained a mixture of *cis*- and *trans*-Pt(ppy)_2_Cl_2_ under mild conditions (*cis* and *trans* refer here to the relative disposition of the Cl ligands in the products). This reagent has since been employed successfully as a mild chlorine-supplying oxidant in the work of Rourke and Gonzalez-Herrero mentioned in the introduction.^10,15,20^ We tested the reactivity of PtL^1–6^Cl toward this reagent in stoichiometric amount. The desired PtL^*n*^Cl_3_ complex was obtained in each case using 1 equiv of PhICl_2_, after around 12 h in CHCl_3_ at ambient temperature. The milder nature of this reagent compared to Cl_2_ is evident from the fact that (i) none of the chlorinated side-product PtL^5Cl^Cl_3_ was formed alongside PtL^5^Cl_3_, and (ii) PtL^6^Cl_3_ was successfully isolated, in contrast to the intractable mixture found when PtL^6^Cl was treated with Cl_2_. The methoxy-substituted PtL^4^Cl, with no substituent in the position *para* to Pt, nevertheless still showed some competitive aryl chlorination even with this reagent, but it proved possible to separate the two products chromatographically.

We cannot conclusively state that the chlorination of the aryl ring occurs on the Pt(II) precursor complex rather than on the Pt(IV) product. Since the electrophilic substitution relies on the electron richness of the aryl ring, it might be intuitive to expect the lower-oxidation state form to be the more reactive. An indication that the rate of aryl chlorination of PtL^4^Cl may be faster than that of oxidation of the Pt(II) center to Pt(IV) is offered by the fact that the aryl chlorinated platinum(II) complex PtL^4Cl^Cl was isolated as a side product during the purification of PtL^4Cl^Cl_3_ and structurally characterized by X-ray diffraction (see Figure S2.3). The conclusion that this is indicative of chlorination of the Pt(II) form remains tentative, however, because in several of the preparations, irrespective of the reagent, there was some evidence that reduction of the PtL^*n*^Cl_3_ materials back to the PtL^*n*^Cl precursors could occur, a point we return to below.

### Structural Characterization of PtL^*n*^Cl_3_ and PtL^*n*Cl^Cl_3_ Complexes in the Solid State

**Figure 3:**
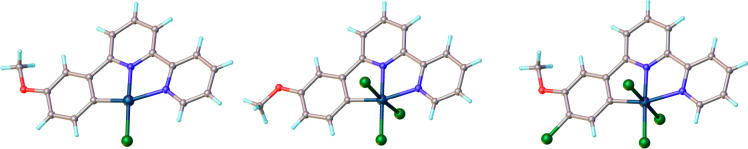
Molecular structures of PtL^5^Cl, PtL^5^Cl_3_, and PtL^5Cl^Cl_3_ in the solid state at 120 K, determined by X-ray diffraction.

Crystals of six of the complexes for X-ray diffraction analysis were obtained from solutions in dimethyl sulfoxide. All of the complexes show the expected pseudo-octahedral coordination of the Pt(IV) ions, with three mutually orthogonal Pt–Cl bonds lying in the plane perpendicular to that of the *NNC*-coordinating ligand (Figure 3 and Figure S2.1; Table 1 and Table S2.1). The unsubstituted complex shows disorder between the phenyl and lateral pyridyl rings (as they are isoelectronic and isolobal), but the presence of a substituent (or thiophene as opposed to phenyl ring in the case of PtL^6^Cl_3_) removes this issue in all the other molecules. For the purposes of a comparison with the Pt(II) precursors, PtL^5^Cl_3_ is taken as a representative example in Table 1, as the structure of its precursor PtL^5^Cl was also determined during this work. This complex also allows the effect of aryl chlorination on the structure to be probed—if any—through the structure of PtL^5Cl^Cl_3_ which was also determined.

**Table 1:** Selected Bond Lengths (Å) and Angles (deg) for PtL^5^Cl, PtL^5^Cl_3_, and PtL^5Cl^Cl_3_

bond length (Å) or bond angle (deg)	PtL^5^Cl	PtL^5^Cl_3_	PtL^5Cl^Cl_3_
Pt–C	1.9937(19)	2.004(2)	2.011(4)
Pt–N1 (lateral)	2.1067(15)	2.137(2)	2.158(4)
Pt–N2 (central)	1.9476(15)	1.9649(19)	1.975(4)
Pt–Cl^equat.^^a^	2.3157(5)	2.3175(6)	2.3146(11)
Pt–Cl^axial^^a^		2.3250(6)	2.3228(12)
Pt–Cl^axial^^a^		2.3137(6)	2.3034(12)
N2–Pt–C	81.82(7)	82.98(9)	81.46(18)
N2–Pt–N1	79.97(6)	79.97(8)	79.40(16)
N1–Pt–Cl^equat.^^a^	98.64(4)	99.58(6)	98.10(11)
C–Pt–Cl^equat.^^a^	99.56(6)	97.48(7)	101.03(15)

a Note: the equatorial plane is considered here, and in the text, to be that of the tridentate ligand. Thus, Cl^equat.^ denotes the chloride positioned *trans* to the central nitrogen of the *NNC* ligand and Cl^axial^ the other two.

Comparing the structures of PtL^5^Cl_3_ and PtL^5^Cl (Figure 3 and Table 1), it can be seen that the oxidation from Pt(II) to Pt(IV) is accompanied by a slight increase in the lengths of all four of the pre-existing bonds to the platinum center, perhaps partly to accommodate the additional ligands at the “axial” positions. The lateral Pt–N bonds expand rather more than the Pt–C or the central Pt–N, which may reflect decreased π back-bonding to the heterocycles. The axial Pt–Cl bonds are similar in length to the equatorial one. The N2–Pt–N1 angle is invariant with oxidation. The other bond angles subtended by the metal change by 1–2°, but the N2–Pt–C angle remains slightly larger than the N2–Pt–N1, as observed in other structurally characterized Pt(*NNC*)Cl complexes. The main effect of chlorination of the aryl ring is to slightly lengthen all three bonds from the metal to the tridentate ligand.

### Synthesis of [PtL^*n*^(*NC*)Cl]^+^ Complexes

**Scheme 3:**
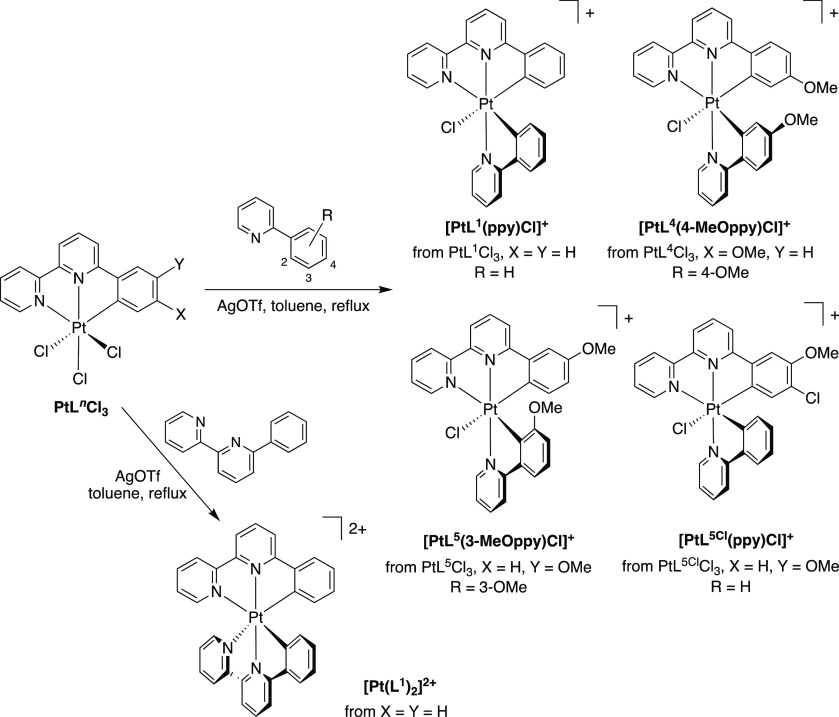
Synthesis of [Pt(*NNC*)(*NC*)Cl]^+^ and [Pt(*NNC*)_2_]^2+^ Complexes from Pt(*NNC*)Cl_3_ Precursors

The reaction of PtL^1^Cl_3_ with 2-phenylpyridine (ppyH) in toluene at reflux for 16 h, in the presence of silver triflate (2 equiv), was found to give the target complex [PtL^1^(ppy)Cl]^+^ (Scheme 3). The selection of these conditions was guided by methodology we had established previously for the preparation of isoelectronic iridium(III) complexes of the form Ir(*NCN*)(*NC*)Cl from [Ir(*NCN*)Cl(μ-Cl)]_2_ precursors.^39,40^ Silver triflate was used to help scavenge the two liberated chloride ions. The product that precipitated from the reaction mixture was separated, washed, and subjected to anion exchange with aqueous KPF_6_ to generate the hexafluorophosphate salt [PtL^1^(ppy)Cl]PF_6_. The yield was typically low, between 10% and 15%, in large part due to the formation of substantial amounts of Pt(II) species. Evidently, PtL^1^Cl_3_ is thermally unstable with respect to reduction back to the Pt(II) precursor or related materials. We sought to optimize the yield of the desired product by using shorter reaction times and/or lower temperatures, but then it does not form in significant amounts. The kinetic inertness of Pt(IV) clearly necessitates the harsh conditions. The use of microwave irradiation in conjunction with shorter reaction times did not improve the yield.

The same procedure was applied to the two methoxy-substituted materials, PtL^4^Cl_3_ and PtL^5^Cl_3_, in reaction with 2-(4-methoxyphenyl)pyridine (4-MeOppyH) and 2-(3-methoxyphenyl)pyridine (3-MeOppyH), respectively, as *NC* proligands. The desired complexes [PtL^4^(4-MeOppy)Cl]PF_6_ and [PtL^5^(3-MeOppy)Cl]PF_6_ were isolated, albeit again in low yield due to extensive competitive reduction to the Pt(II) precursors and the arduous purification needed. A sample of [PtL^5Cl^(ppy)Cl]PF_6_ was also prepared by reaction of Pt^5Cl^Cl_3_ with ppyH. Repeated attempts to prepare corresponding complexes from PtL^2^Cl_3_ and PtL^3^Cl_3_ were plagued by competitive reduction and persistence of impurities in the isolated products. The attempted synthesis of [PtL^6^(thpy)Cl]PF_6_ upon reaction of PtL^6^Cl with 2-thienylpyridine (thpyH) under the same conditions did lead to a pure sample of the desired product, confirmed by X-ray crystallography, but this complex proved to be unstable in solution, probably through dissociation of the chloride ligand.

These [PtL^*n*^(*NC*)Cl]^+^ complexes form a hitherto unreported new class of complexes. In principle, two possible isomers could form according to whether it is the pyridine ring or the aryl ring of the *NC* ligand that is positioned *trans* to the central pyridine of the *NNC* ligand. ^1^H NMR spectroscopic evidence indicates that it is the former isomer that was isolated (as shown in the Scheme and confirmed by crystallography, see below). For example, the proton *ortho* to the coordinating nitrogen atom of the *NC* ligand is strongly deshielded at around 10 ppm, consistent with its proximity to the Pt–Cl bond. A similar trend was observed for the corresponding proton in structurally related Ir(*NCN*)(*NC*)Cl complexes, with which the core structure of these new Pt(IV) complexes is isoelectronic, and also in [Ir(*NNN*)(*NC*)Cl]^+^ complexes.^39−41^ Meanwhile, the proton *ortho* to the C–Pt of the metalated aryl ring of the *NC* ligand is strongly shielded, appearing at <6.5 ppm in each case. Such an effect is anticipated due to the C–H bond being positioned over the plane of the central pyridyl ring of the *NNC* ligand and thus in the zone of the diamagnetic ring current exerted by the latter. The proton in the tridentate ligand *ortho* to C–Pt is similarly shifted to low frequency upon introduction of the ppy ligand, lying as it does over the plane of the *NC* pyridyl ring; e.g., in [PtL^1^(ppy)]PF_6_, Δδ_H_ for this proton is around −0.4 ppm. In the methoxy-substituted complexes, the electron-donating effect of the substituent augments the ring current effect, leading to particularly low-frequency δ values for these *ortho*-to-C–Pt protons (e.g., 5.68 and 5.62 ppm in [PtL^4^(4-MeOppy)Cl]PF_6_ for the bi- and tridentate ligands respectively). In the Pt(IV) complexes, all the protons *ortho* to C–Pt or N–Pt bonds show coupling to ^195^Pt (best resolved at low fields). The resulting characteristic satellites appear more clearly than those in the PtL^*n*^Cl_3_ intermediates, probably a consequence of the smaller anisotropy of the [Pt(*NNC*)(*NC*)Cl]^+^ complexes compared to the trichlorides, where the set of ligands in the two planes are very different from one another.^42^

In the case of [PtL^5^(3-MeOppy)Cl]^+^, further isomerism can be envisaged, according to whether the *NC* ligand binds in such a way that its methoxy substituent is positioned *para* to the Pt–C bond (i.e., as observed for the methoxy substituent in the *tridentate* ligand of the Pt(II) precursor PtL^5^Cl) or *ortho* to the Pt–C bond. The latter was exclusively observed, identified by ^1^H NMR spectroscopy (and subsequently confirmed by crystallography—see below). Thus, there is a substantial difference in δ for the OMe protons of the bidentate ligand (*ortho* to C–Pt, 3.16 ppm) compared to the tridentate ligand (*para* to C–Pt, 3.81 ppm), in contrast to a pair of values similar to one another in the case of [PtL^4^(4-MeOppy)Cl]^+^ (3.59 and 3.67 ppm for OMe in the bi- and tridentate ligands, respectively). The shift to high frequency for the OMe protons of the bidentate ligand in [PtL^5^(3-MeOppy)Cl]^+^ is again a result of being positioned in the zone of the ring current exerted by the central pyridine of the tridentate ligand. Further support for the assignment comes from the NOESY spectrum, which shows the two expected cross peaks for the OMe protons of the tridentate ligand (enhancements due to the protons on the carbons *meta* to the C–Pt bond), but only one such cross peak for the OMe protons of the bidentate ligand.

### Structural Characterization of [PtL^*n*^(*NC*)Cl]^+^ Complexes

**Figure 4:**
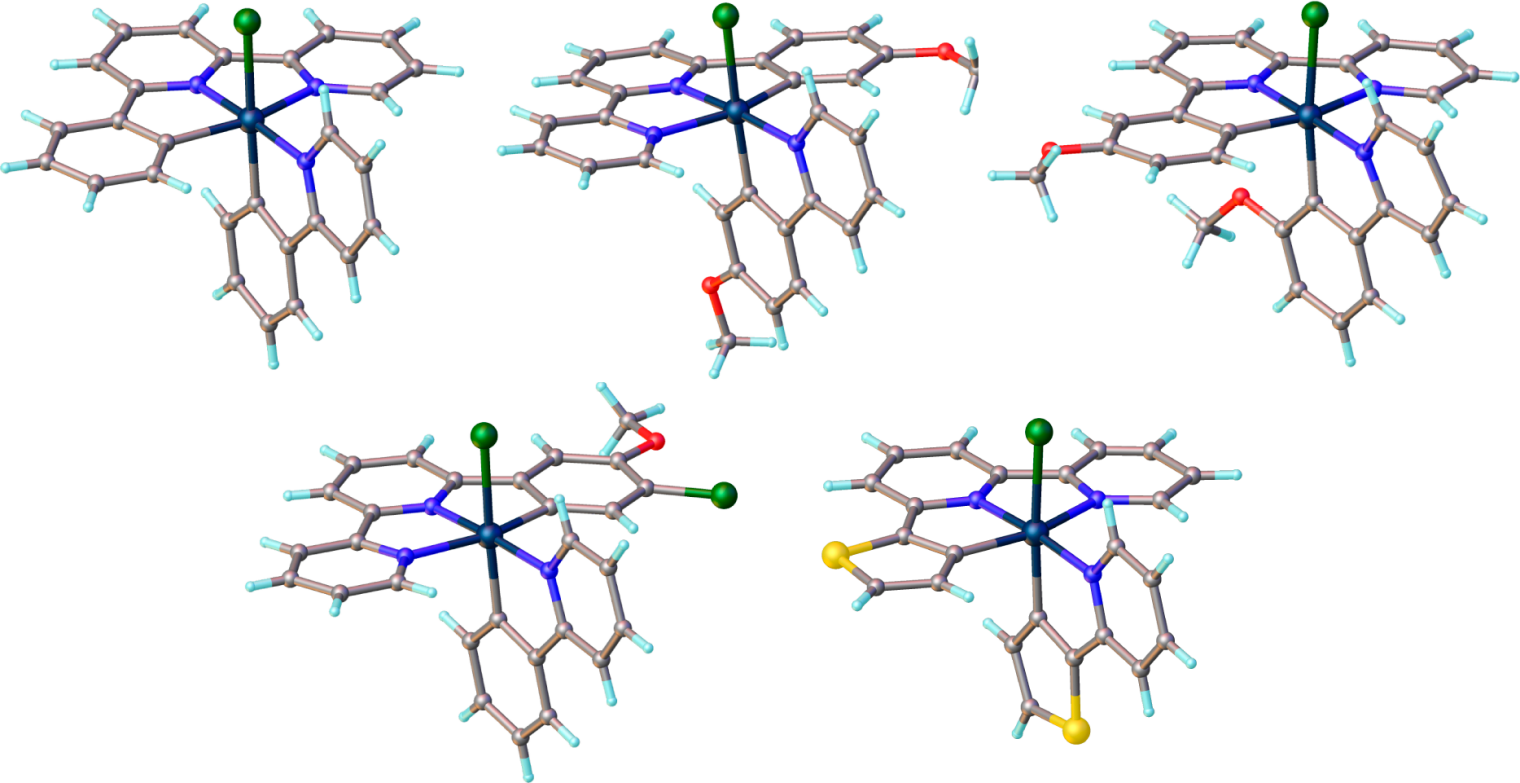
Structures of [PtL^1^(ppy)Cl]^+^, [PtL^4^(4-MeOppy)Cl]^+^, [PtL^5^(3-MeOppy)Cl]^+^, [PtL^5Cl^(ppy)Cl]^+^, and [PtL^6^(thpy)Cl]^+^ determined by X-ray diffraction of the hexafluorophosphate or trifluoromethanesulfonate salts at 120 K. Note that the complexes are chiral; the structures of [PtL^5^(3-MeOppy)Cl]PF_6_ and [PtL^6^(thpy)Cl]PF_6_ are for the opposite enantiomer to the other three.

Crystals suitable for X-ray diffraction analysis were obtained for [PtL^1^(ppy)Cl]OTf, [PtL^4^(4-MeOppy)Cl]PF_6_, [PtL^5^(3-MeOppy)Cl]OTf, [PtL^5Cl^(ppy)Cl]PF_6_, and [PtL^6^(thpy)Cl]PF_6_. They crystallize in centrosymmetric space groups (*P*2_1_/*c* for [PtL^1^(ppy)Cl]OTf and *P*1̅ for the others) and the crystals thus comprise a racemic mixture of the two enantiomers of each complex. Each structure shows the expected pseudo-octahedral geometry around the Pt(IV) center, with the plane of the bidentate *NC* ligand perpendicular to that of the tridentate *NNC* ligand, and with the coordination number of 6 being completed by the monodentate chloride (Figure 4 and Table 2). In each case, the conclusions from ^1^H NMR as to the orientation of the *NC* ligand are confirmed: the pyridyl ring of the *NC* ligand is bound *trans* to the pyridyl ring of the *NNC* ligand, with the phenyl ring of the *NC* ligand metalated *trans* to the chloride ligand. The preference for this isomer probably arises from the relative *trans* influences of the ligating atoms, namely, C^–^ > *N* > Cl.^39^

**Table 2:** Selected Bond Lengths (Å) and Angles (deg) for [Pt(*NNC*)(*NC*)Cl]^+^ Complexes

bond length (Å) or bond angle (deg)	[PtL^1^(ppy)Cl]^+^^a^	[PtL^4^(4-MeO-ppy)Cl]^+^^b^	[PtL^5^(3-MeO-ppy)Cl]^+^^a^	[PtL^5Cl^(ppy)Cl]^+^^b^	[PtL^6^(thpy)Cl]^+^^b^
Pt–C^*NNC*^	2.089(5)/2.067(4)	1.996(6)	2.027(6)	2.018(4)	2.021(7)
Pt–N1^*NNC*^ (lateral)	2.067(4)/2.089(5)	2.141(5)	2.154(5)	2.134(4)	2.124(6)
Pt–N2^*NNC*^ (central)	1.972(4)	1.962(5)	1.983(5)	1.965(4)	1.981(6)
Pt–C^*NC*^	2.013(4)	2.031(6)	2.035(6)	2.015(4)	2.014(7)
Pt–N^*NC*^	2.037(4)	2.035(5)	2.046(4)	2.046(4)	2.048(6)
Pt–Cl	2.417(1)	2.4232(13)	2.4199(14)	2.427(1)	2.4121(17)
N1^*NNC*^–Pt–C^*NNC*^	160.70(18)	161.7(2)	161.0(2)	161.20(17)	159.6(3)
N2^*NNC*^–Pt–C^*NNC*^	80.40(16)/80.34(17)	82.1(2)	82.2(2)	81.89(16)	81.0(3)
N2^*NNC*^–Pt–N1^*NNC*^	80.34(17)/80.40(16)	79.65(19)	78.81(19)	79.33(15)	78.6(3)
N2^*NNC*^–Pt–N^*NC*^	176.44(15)	174.77(19)	178.0(2)	176.49(15)	174.7(2)
N1^*NNC*^–Pt–Cl	90.46(12)/90.02(12)	86.96(13)	88.12(14)	88.20(11)	89.57(17)
N^*NC*^–Pt–C^*NC*^	80.84(17)	81.3(2)	80.7(2)	81.28(17)	81.1(3)
N^*NC*^–Pt–Cl	96.01(12)	95.46(14)	93.62(14)	95.51(11)	94.26(18)
C^*NC*^–Pt–Cl	176.74(13)	176.59(17)	173.01(18)	176.73(14)	175.2(2)

a Trifluoromethanesulfonate salt.

b Hexafluorophosphate salt.

The identity of [PtL^5^(3-MeOppy)Cl]PF_6_ in the solid state is also confirmed, with the methoxy substituent of the *NC* ligand *ortho* to the C–Pt, as deduced by the solution-state NMR spectroscopy described above. The structure of [PtL^1^(ppy)Cl]PF_6_ shows disorder between the lateral pyridyl and the phenyl rings of the *NNC* ligand, just as there was in PtL^1^Cl_3_ and for the same reason. There is no such disorder in the other complexes, where the substituent(s) in the aryl ring ensure that the aryl and pyridyl rings are no longer structurally equivalent. Table 2 compiles selected bond lengths and angles for the parent unsubstituted complex, together with those for [PtL^5Cl^(ppy)Cl]PF_6_ as a representative example of one of the others—with no disorder—and thus allowing definitive assignment of Pt–C and lateral Pt–N bond lengths. The data show the usual trends for complexes of other metal ions with phbpy: a Pt–N significantly shorter for the bond to the central pyridine than the lateral pyridine, reflecting the nonideal bite angle of tridentate ligands that form two 5-membered chelates, and a slightly shorter Pt–C bond compared to the Pt–N opposite it. The Pt–N bond length to the bidentate ligand lies between the values of those to the central and lateral pyridine rings of the tridentate ligand.

### Synthesis and Structural Characterization of a Bis-tridentate [Pt^IV^(*NNC*)_2_]^2+^ Complex

Encouraged by the modest success in preparing these unprecedented Pt(IV) complexes, despite the disappointing yields, we turned to the target of a bis-tridentate complex. The reaction of PtL^1^Cl_3_ with 6-phenyl-2,2′-bipyridine (HL^1^), under the same conditions as those used above but in this case using 3 equiv of silver triflate, led to [Pt(*NNC*-L^1^)_2_](PF_6_)_2_ (Scheme 3). The purification of this dicationic material was achieved by column chromatography using a highly polar eluant comprising acetonitrile, water, and potassium nitrate. The ^1^H NMR spectrum shows a large upfield shift of the proton in the phenyl ring *ortho* to the Pt–C bond, moving from δ_H_ = 7.5 ppm in PtL^1^Cl_3_ to 6.4 ppm in [Pt(L^1^)_2_]^2+^. The corresponding proton in the pyridyl ring also shifts upfield, albeit to a lesser extent. Upfield shifts of such protons have been widely observed in bis-tridentate complexes of other d^6^ ions, such as Ru(II) and Ir(III), due to shielding by the diamagnetic ring current of the central pyridine of the second ligand, above and below which they lie. Like the complexes with the bidentate ligand above, this complex also has *C*_1_ symmetry. It is a rare example of a homoleptic chiral Pt(IV) complex. Reactions of the other PtL^*n*^Cl_3_ complexes with HL^1^ showed evidence of the formation of corresponding heteroleptic complexes of the form [PtL^*n*^L^1^]^2+^, but the reactions were accompanied by the formation of several side-products, again including PtL^*n*^Cl. It has not proved possible to isolate analytically pure samples of these target heteroleptic compounds.

### Absorption and Emission Spectroscopy of the Pt^II^(*NNC*)Cl Precursor Complexes

**Figure 5:**
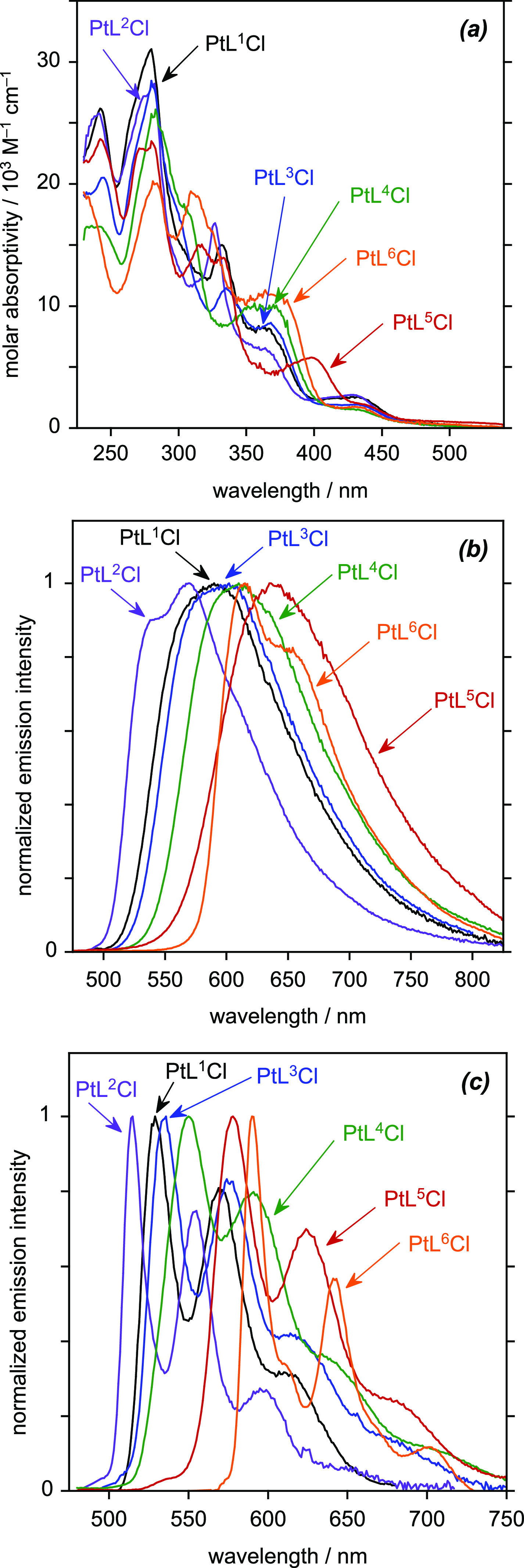
(a) UV–vis absorption spectra of PtL^1–6^Cl in CH_2_Cl_2_ solution at 295 K. (b) Emission spectra of PtL^1–6^Cl in deoxygenated CH_2_Cl_2_ at 295 K. (c) Emission spectra of PtL^1–6^Cl at 77 K in diethyl ether/isopentane/ethanol (2:2:1 v/v).

Although a wide range of Pt(*NNC*)Cl complexes have been investigated in the literature, there is surprisingly no systematic study of the influence of substituents in the aryl ring on the photophysical properties, nor have luminescence data for all the complexes used here been reported. Before considering the properties of the new families of Pt(IV) complexes, therefore, a brief discussion of our evaluation of the absorption and emission of the Pt(II) precursors is required. The general form of their UV–vis absorption spectra (Figure 5a and Table 3) is quite typical of cyclometalated complexes based on arylpyridine ligands. Intense bands at λ < 330 nm attributed to π–π* transitions within the ligands are accompanied by somewhat weaker bands at longer wavelengths stretching well into the visible region that have no counterparts in the proligands. These are typically assigned to transitions of d_Pt_|π _*NN*C_ → π*_NN*C*_ character (MLCT/LLCT). The spectra of all six complexes are similar to one another, with the lowest-energy band of significant intensity appearing around 435 nm in each case. The spectrum of PtL^5^Cl differs, however, in that it shows a well-defined band centered at about 400 nm whereas the other five complexes show a broader band around 370 nm.

**Table 3:** Photophysical Data for the Pt^II^(*NCN*-L^*n*^)Cl Precursor Complexes

	absorption^a^	emission at 295 K^a^			emission at 77 K^b^	
complex	λ_max_/nm(ε/M^–1^ cm^–1^)	λ_max_/nm	Φ_lum_/%^c^	τ/ns^d^	λ_max_/nm	τ/ns
PtL^1^Cl	242 (26000),279 (31000),332 (15000),365 (7900),433 (2600)	590	1.7	270 [180]	529, 571, 611	16000
PtL^2^Cl	240 (25000),273 (27000),281 (28000),327 (17000),367 (6100),430 (2700)	538sh, 570	4.3	440 [300]	515, 554, 597	23000
PtL^3^Cl	245 (21000),281 (28000),334 (11000),370 (8500),433 (1900)	599	2.7	470 [240]	535, 576, 616, 683	13000
PtL^4^Cl	240 (16000),284 (25000),307 (17000),365 (9600),433 (1500)	610	2.6	830 [300]	551, 590, 640, 706	11000
PtL^5^Cl	243 (22000),273 (22000),280 (22000),316 (14200),333 (13000),398 (5500),441 (1800)	640	1.2	250 [150]	578, 624, 679	10000
PtL^6^Cl	232 (19000),284 (21000),311 (20000),370 (11000),433 (1700)	614, 651	3.6	1300 [300]	590, 610sh, 642, 702	14000

a In CH_2_Cl_2_.

b In diethyl ether/isopentane/ethanol (2:2:1 v/v).

c Photoluminescence quantum yield in deoxygenated solution, measured using [Ru(bpy)_3_]Cl_2_ (aq) as the standard, for which Φ_lum_ = 0.04. The likely uncertainty on Φ_lum_ is around ±20%.

d In deoxygenated solution; corresponding values in parentheses refer to air-equilibrated solutions. Estimated uncertainty on τ is around ±10%.

The photoluminescence spectra show more variation with the aryl substituent (Figure 5b,c). At 77 K, all of the complexes show the same form of vibrationally well-resolved spectrum, with three vibrational components clearly visible, and with the 0,0 component being the most intense in each case. The λ(0,0) emission increases slightly upon introduction of the methyl group (i.e., PtL^1^Cl to PtL^3^Cl), more for the methoxy group in the same position (PtL^4^Cl) and even more when the methoxy is *para* to the Pt–C bond (PtL^5^Cl). This trend is consistent with the usual interpretation of the emissive state being of ^3^[d_Pt_|π_Ar_ → π*_NN_] character, where the HOMO is delocalized predominantly over the metal and aryl ring of the *NNC* ligand (denoted π_Ar_) and the LUMO is largely dominated by the diimine portion of the ligand (denoted π*_NN_) . Thus, the more electron-rich the substituents in the aryl ring (e.g., MeO versus Me), the smaller the HOMO–LUMO gap is expected to be, and the more red-shifted the emission. An electron-donating substituent *para* to the metal–carbon bond is more effective at increasing the electron density at the metal than when positioned *meta*, due to the conjugation pathway. The more electron-rich nature of thienyl compared to phenyl rings similarly accounts for PtL^6^Cl being substantially red-shifted compared to PtL^1^Cl. The blue-shift observed on going from PtL^1^Cl to PtL^2^Cl is similarly explained by the electron-withdrawing nature of the CF_3_ group in stabilizing the HOMO.

The complexes remain emissive in solution at room temperature, but the spectra become broader, with some vibrational structure evident for PtL^2^Cl and PtL^6^Cl only (Figure 5b). The order of emission λ_max_ is the same as at 77 K, except that λ_max_ for PtL^5^Cl is red-shifted to a little beyond that of PtL^6^Cl. The rigidochromic effect (difference between λ_max_ at 77 K compared to room temperature) is larger for the phenyl complexes PtL^1–5^Cl than for the thienyl complex PtL^6^Cl, probably reflecting the greater LLCT versus MLCT character of the emissive state in the latter, and as observed in related *NC*-coordinated thienylpyridine complexes of Pt(II) and Ir(III).^43^ Under these conditions, the quantum yields are around a few % and lifetimes of the order of a few hundred nanoseconds, typical of phosphorescence from Pt(*NNC*)Cl systems (Table 3). The most red-shifted complex PtL^5^Cl has the lowest quantum yield and shortest lifetime, consistent with the greater nonradiative decay expected for lower-energy excited states among structurally similar complexes.^44^ The thienyl complex PtL^6^Cl is again an outlier in displaying a longer lifetime despite its low-energy emission, a feature that is again consistent with lower metal-character to its emissive state,^43^ resulting in less efficient spin–orbit coupling to facilitate the formally forbidden phosphorescence transition.

### Absorption Spectra of the Pt^IV^(*NNC*)Cl_3_ Complexes

**Figure 6:**
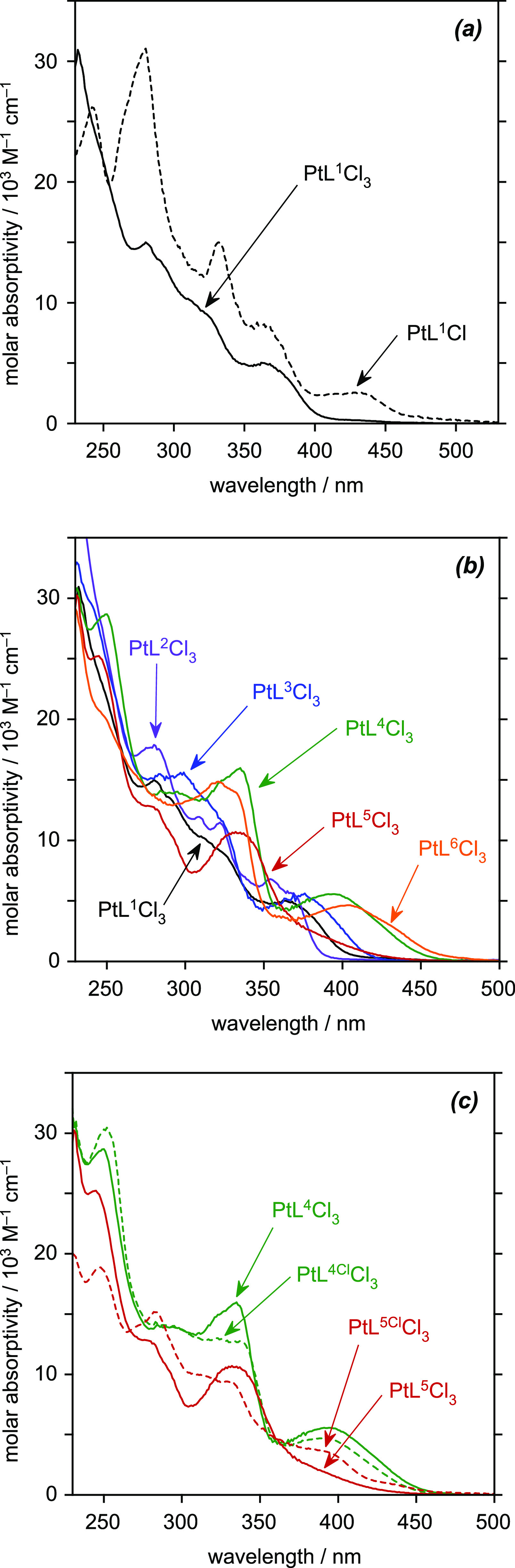
UV–vis absorption spectra in CH_2_Cl_2_ solution at 295 K: (a) spectra of PtL^1^Cl_3_ and PtL^1^Cl for comparison; (b) overlaid spectra of PtL^1–6^Cl_3_; and (c) spectra of the phenyl-chlorinated products PtL^4Cl^Cl_3_ and PtL^5Cl^Cl_3_ with the nonchlorinated analogues reproduced for comparison.

The absorption spectra of the trichloroplatinum(IV) complexes PtL^*n*^Cl_3_ have been recorded in dichloromethane solution at room temperature (Figure 6 and Table 4). The spectrum of the unsubstituted complex PtL^1^Cl_3_ is compared with that of its Pt(II) precursor PtL^1^Cl in Figure 6a. The most notable difference between the spectra is the absence in the Pt(IV) compound of the lowest-energy band around 430 nm that was present prior to oxidation, consistent with the visually much paler color of the Pt(IV) material. A similar observation is made for PtL^2–6^Cl_3_, each showing the disappearance of the longest wavelength band: pairs of corresponding spectra are provided in the Supporting Information (Figure S3.1). The change can readily be interpreted in terms of the change in the oxidation state at the metal, which will serve to lower the energy of the metal-centered orbitals in the ^1^[d_Pt_|π_Ar_ → π*_NN_] transitions, thus increasing the absorption energy. A second difference clearly visible in Figure 6a is the rather lower molar absorptivities of the Pt(IV) complex with fewer sharply defined bands, a trend generally also observed in the other complexes (Figure S3.1).

**Table 4:** UV–Vis Absorption Data for PtL^*n*^Cl_3_ in CH_2_Cl_2_ at 295 K

complex	absorption λ_max_/nm(ε/M^–1^ cm^–1^)
PtL^1^Cl_3_	281 (14900), 324sh (8970), 367 (4870)
PtL^2^Cl_3_	271 (17200),310 (11800),323 (11400),355 (6760),371 (5340)
PtL^3^Cl_3_	295 (15400),322sh (11800),374 (5500)
PtL^4^Cl_3_	248 (28500),292 (13900),334 (15900),394 (5570)
PtL^4Cl^Cl_3_	251 (30200),292 (13900),322 (12600),336 (12700),389 (4690)
PtL^5^Cl_3_	244 (25200),276 (12900),333 (10600), 390sh (1950)
PtL^5Cl^Cl_3_	248 (18900),284 (15100),330sh (9390),388sh (3690),437sh (840)
PtL^6^Cl_3_	247sh (20400),321 (14900),333sh (13900),403 (4640)

The spectra of the set of six complexes PtL^1–6^Cl_3_ are overlaid in Figure 6b. It is interesting to note that there is rather more variation in the spectra according to the substituents or identity of the aromatic ring than there was in the absorption of the Pt(II) complexes (Figure 5a), but some trends remain that were evident from the *emission* spectra of the Pt(II) complexes. For example, there is a blue-shift in the lowest-energy absorption band on introduction of the electron-withdrawing CF_3_ substituent in PtL^2^Cl_3_ and, conversely, a red-shift arising from the methyl substituent in PtL^3^Cl_3_. For the methoxy-substituted complex PtL^4^Cl_3_ and thienyl derivative PtL^6^Cl_3_, the band is red-shifted further to a λ_max_ around 400 nm and extending well into the visible region, tailing to λ > 450 nm. Such a trend suggests that the underlying transitions again feature significant π_Ar_ → π*_NN_ character and thus have an energy influenced heavily by the substituents in the aryl ring, or whether it is thienyl versus phenyl. Interestingly, the *para*-to-Pt methoxy complex PtL^5^Cl_3_ does not show a clear-cut band around 400 nm, although it does feature a very long tail in this region extending to about 450 nm. Moreover, the phenyl-chlorinated analogue of this compound, PtL^5Cl^Cl_3_, shows a band in this region, as evident from Figure 6c which compares the spectra of the two chlorinated products with their parents. It seems likely that such a band is present, therefore, also in PtL^5^Cl_3_ but that its intensity is suppressed relative to the other complexes.

None of the PtL^*n*^Cl_3_ complexes display any detectable photoluminescence in solution at room temperature. In some samples at 77 K, there was evidence of some weak emission, but the spectral profile was found to closely resemble that of the Pt(II) precursors. Given that we noted some competitive reduction of the PtL^*n*^Cl_3_ materials back to PtL^*n*^Cl (as noted earlier in the Synthesis of Pt(*NNC*)Cl3 Complexes section), some emission from Pt(II) contaminants is not unexpected.

### Absorption and Emission Spectroscopy of [Pt(*NNC*)(*NC*)Cl]^+^ Complexes

**Figure 7:**
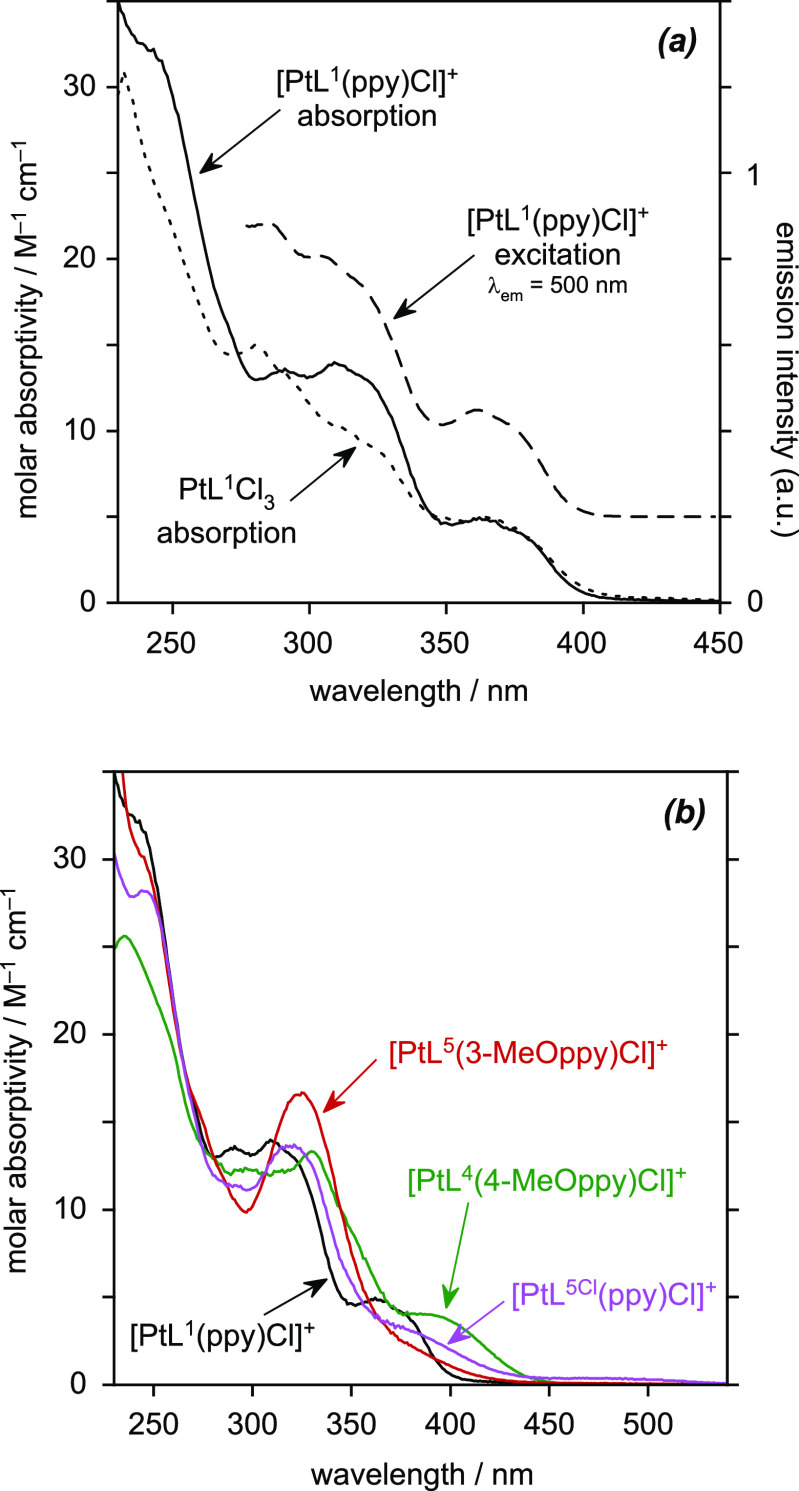
(a) UV–vis absorption spectrum and photoluminescence excitation spectrum of [PtL^1^(ppy)Cl]PF_6_ in MeCN at 295 K (solid and long dashed lines, respectively), with the absorption spectrum of its precursor PtL^1^Cl_3_ for comparison (short dashed line). (b) UV–vis absorption spectra of the four isolated [Pt^IV^(*NNC*)(*NC*)Cl]PF_6_ complexes, under the same conditions.

The absorption spectra of the four complexes of type [Pt(*NNC*)(*NC*)Cl]^+^ that could be isolated in sufficient purity for photophysical analysis, namely, [PtL^1^(ppy)Cl]^+^, [PtL^4^(4-MeOppy)Cl]^+^, [PtL^5^(3-MeOppy)Cl]^+^, and [PtL^5Cl^(ppy)Cl]^+^, are shown in Figure 7, with numerical data in Table 5. The first of these, [PtL^1^(ppy)Cl]^+^, can be considered the fully unsubstituted, “parent” complex of this type. Figure 7a compares its absorption spectrum with that of the precursor PtL^1^Cl_3_. The spectra are similar. The main difference is the higher molar absorptivity of [PtL^1^(ppy)Cl]^+^ across most of the shorter-wavelength range <350 nm, which can readily be attributed to the presence of the additional aromatic rings of the *NC* ligand and transitions associated with them. It is notable, on the other hand, that there is barely any difference between the spectra in the region of the lowest-energy band (i.e., λ > 350 nm). This observation is consistent with the notion that the unoccupied orbitals of lowest energy in this complex would be expected to be the π* of the bipyridine moiety of the *NNC* ligand. The extended conjugation over the two *ortho*-linked pyridine rings will lead to the lowest-energy charge-transfer transitions being those involving the *NNC* ligand as the acceptor, as opposed to the *NC* ligand. The fact that there is essentially no shift in this lowest-energy band relative to PtL^1^Cl_3_ then implies that the energy of filled orbitals involved in the transition is relatively unchanged, pointing toward a predominantly π_NNC_ → π*_NNC_ character to the transition. Similar observations are made for the other three complexes upon comparing them with their precursors: corresponding figures are provided in the Supporting Information (Figure S3.2).

**Table 5:** Photophysical Data for the [Pt^IV^(*NCN*)(*NC*)Cl]^+^ and [Pt^IV^(*NCN*)_2_]^2+^ Complexes

	absorption at 298 K^a^	emission at 298 K^a^					emission at 77 K^b^	
complex	λ_max_/nm(ε/M^–1^ cm^–1^)	λ_max_/nm	Φ_lum_/10^–2^^c^	τ/μs^d^	*k*_r_/10^3^ s^–1^^e^	∑*k*_nr_/10^3^ s^–1^^e^	λ_max_/nm	τ/μs
[PtL^1^(ppy)Cl]^+^	242sh (32100),290 (13400),310 (13900),364 (4850),378sh (3960)	472, 501, 534	4.0	42 [−f]	0.94	23	461, 496, 527sh, 536, 571, 619	280
[PtL^4^(4-MeO-ppy)Cl]^+^	298 (12300),330 (13300),392 (3950)	565	0.90	7.7 [0.52]	1.2	130	487, 522, 562, 607	240
[PtL^5Cl^(ppy)Cl]^+^	247 (28100),319 (13700),383 (2980),464sh (360)	548	1.5	5.0 [0.7]	3.0	200	493, 530, 569, 623	180
[PtL^5^(3-MeO-ppy)Cl]^+^	246sh (29800),324 (16500),386sh (1850)	559	0.40	0.77 [0.39]	6.0	1300	490, 526, 561, 616	120
[Pt(L^1^)_2_]^2+^	248 (60400),287 (29200),319 (17300),362 (13900),378sh (11000)	474, 503, 539, 586sh	1.0	30 [−f]	0.33	33	465, 499, 530sh, 540, 577, 633	250

a In CH_3_CN.

b In butyronitrile.

c Photoluminescence quantum yield in deoxygenated solution, measured using [Ru(bpy)_3_]Cl_2_ (aq) as the standard, for which Φ_lum_ = 0.04. The likely uncertainty on Φ_lum_ is around ±20%.

d In deoxygenated solution; corresponding values in parentheses refer to air-equilibrated solutions. Estimated uncertainty on τ is around ±10%.

e Radiative, *k*_r_, and nonradiative, ∑*k*_nr_, rate constants estimated assuming that the emitting state is formed with unit efficiency such that *k*_r_ = Φ_lum_/τ and ∑*k*_nr_ = (1 – Φ_lum_)/τ.

f Quenching by oxygen led to an emission intensity that was too low to reliably determine a lifetime in air-equilibrated solution.

The variation in the absorption spectra according to the substituent in the aryl ring (Figure 7b) shows that there is a red-shift in the lowest-energy band for [PtL^4^(4-MeOppy)Cl]^+^ and [PtL^5Cl^(ppy)Cl]^+^, and a long-wavelength tail in [PtL^5^(3-MeOppy)Cl]^+^, just as there was in the PtL^*n*^Cl_3_ precursors. The trend is consistent with an increase in electron density on the aryl ring in the π_NNC_ → π*_NNC_ transition upon introduction of a methoxy substituent.

**Figure 8:**
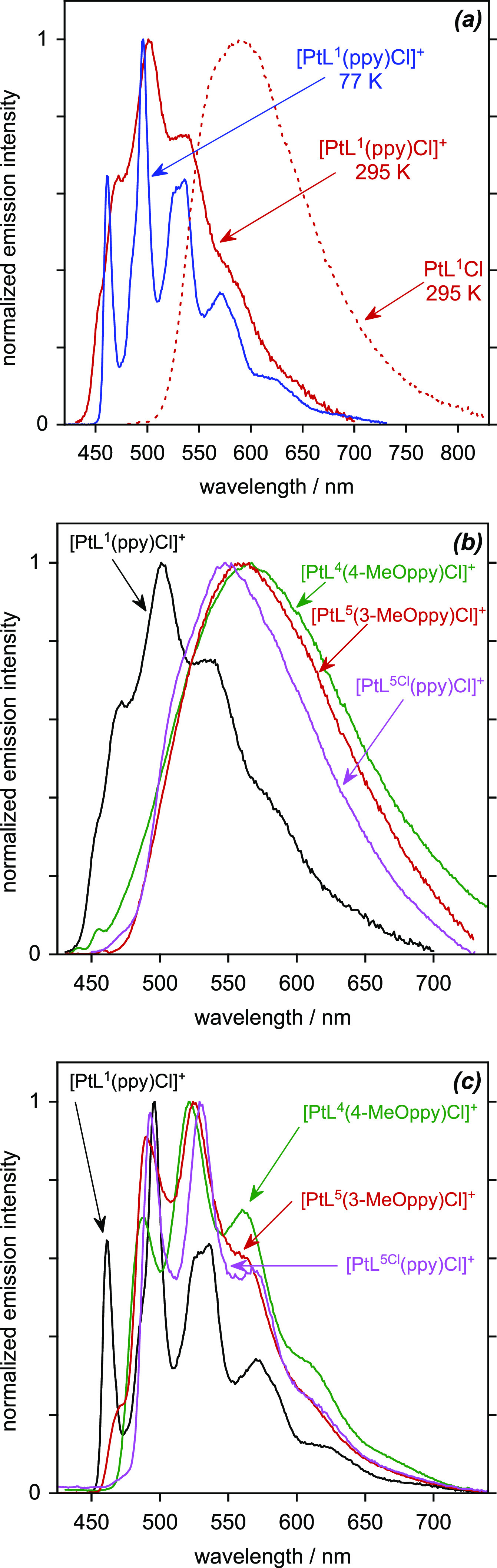
(a) Emission spectra of [PtL^1^(ppy)Cl]^+^ in MeCN at 295 K (solid red line) and in EPA at 77 K (blue line), together with the spectrum of PtL^1^Cl in CH_2_Cl_2_ at 295 K for comparison. (b) Emission spectra of the four isolated [Pt(*NCN*)(*NC*)Cl]^+^ complexes in MeCN at 295 K. (c) Corresponding spectra in butyronitrile at 77 K.

All four members of this new class of platinum(IV) complexes are found to be luminescent in deoxygenated solution at room temperature (Figure 8 and Table 5), and their luminescence excitation spectra closely match the absorption spectra (Figure 7a and Figure S3.2). Considering first the unsubstituted, parent complex [PtL^1^(ppy)Cl]^+^, the emission spectra at 295 K and at 77 K are shown in Figure 8a, together with the spectrum of PtL^1^Cl (reproduced from Figure 5b) for comparison. The Pt(IV) complex displays a somewhat structured spectrum, with a vibrational progression of around 1300 cm^–1^, typical of coupling to aromatic C=C vibrations, and with the (0,1) band apparently the component of highest intensity. There is a large blue-shift in the emission spectrum relative to that of the Pt(II) precursor, mirroring the trend already observed in absorption (Figure 6a). The luminescence quantum yield under these conditions is 4%, and the temporal decay of the emission follows monoexponential kinetics with a lifetime of 42 μs. The emission is severely quenched in the presence of oxygen, as might be expected for such a long-lived excited state: air-equilibrated solutions show only a very weak signal. The long lifetime is consistent with the emission being phosphorescence from the triplet state, but the value is 2 orders of magnitude longer than that of PtL^1^Cl. Making the approximation that the emitting state is formed with unit efficiency (a reasonable approximation given the close match of the excitation and absorption spectra), the radiative rate constant, *k*_r_, is estimated to be around 940 s^–1^ (Table 5). This value is small compared not only with efficient organometallic emitters like Ir(ppy)_3_ and Pt(dpyb)Cl but also with the Pt(*NNC*)Cl precursor complexes (for which *k*_r_ is around 10^4^–10^5^ s^–1^), suggesting that spin–orbit coupling pathways are much less efficient in the Pt(IV) complex. Such a conclusion is consistent with an excited state of predominantly ^3^[π_NNC_ → π*_NNC_] character, with relatively little metal character, in line with the conclusions about the lowest-energy singlet states from absorption spectroscopy above.

This interpretation is supported by the data at 77 K. First, the lifetime at this temperature is increased to a very long value of 280 μs (compared to 16 μs for Pt^1^Cl). Second, the emission spectrum—which now displays very well-resolved vibrational structure—is only marginally blue-shifted compared to the room-temperature spectrum, whereas PtL^1^Cl displays a large blue-shift of around 2000 cm^–1^ on cooling. Large rigidochromic effects are more typical of complexes with excited states of MLCT character than those where the excited state is more ligand-localized.

The presence of the methoxy substituent in the other three complexes, either *meta* or *para* to the platinum, is seen to lead to a red-shift in the emission to around 560 nm and a loss of the vibrational structure at room temperature (Figure 8b). Structure similar to the parent complex is retained at low temperature (Figure 8c), and the red-shift can be quantified from the positions of the 0,0 bands to be around 1500 cm^–1^. There is little difference in the spectra among these three methoxy complexes. A lowering in energy of the excited state upon introduction of a methoxy substituent in the phenyl ring is consistent with a ^3^ILCT assignment, as discussed above for the corresponding singlet state in absorption. The red-shift is accompanied by a significant reduction in the lifetime and quantum yield, which appears to stem primarily from a substantial increase in the nonradiative decay rate ∑*k*_nr_ (Table 5). The complex [PtL^5^(3-MeOppy)Cl]^+^ suffers from especially severe nonradiative decay, and it seems likely that this is associated with a steric influence of the methoxy substituent *ortho* to the C–Pt bond in weakening the binding of the *NC* ligand and hence the overall ligand field strength, as observed in complexes of other metal ions with substituents *ortho* to M–L bonds.^45^ Interestingly, the detrimental effect of the faster nonradiative decay in the two L^5^ complexes appears to be partially offset by a modest increase in the radiative rate constant, particularly for [PtL^5^(3-MeOppy)Cl]^+^. This may be due to an effect of the methoxy substituents—when disposed *para* or *ortho* to the platinum—in increasing the electron density at the metal and hence its participation in the excited state: higher metal character typically facilitates the formally spin-forbidden triplet radiative decay. We note that a trend to shorter lifetimes is also observed at 77 K.

### Absorption and Emission Spectroscopy of [Pt(*NNC*)_2_]^2+^

**Figure 9:**
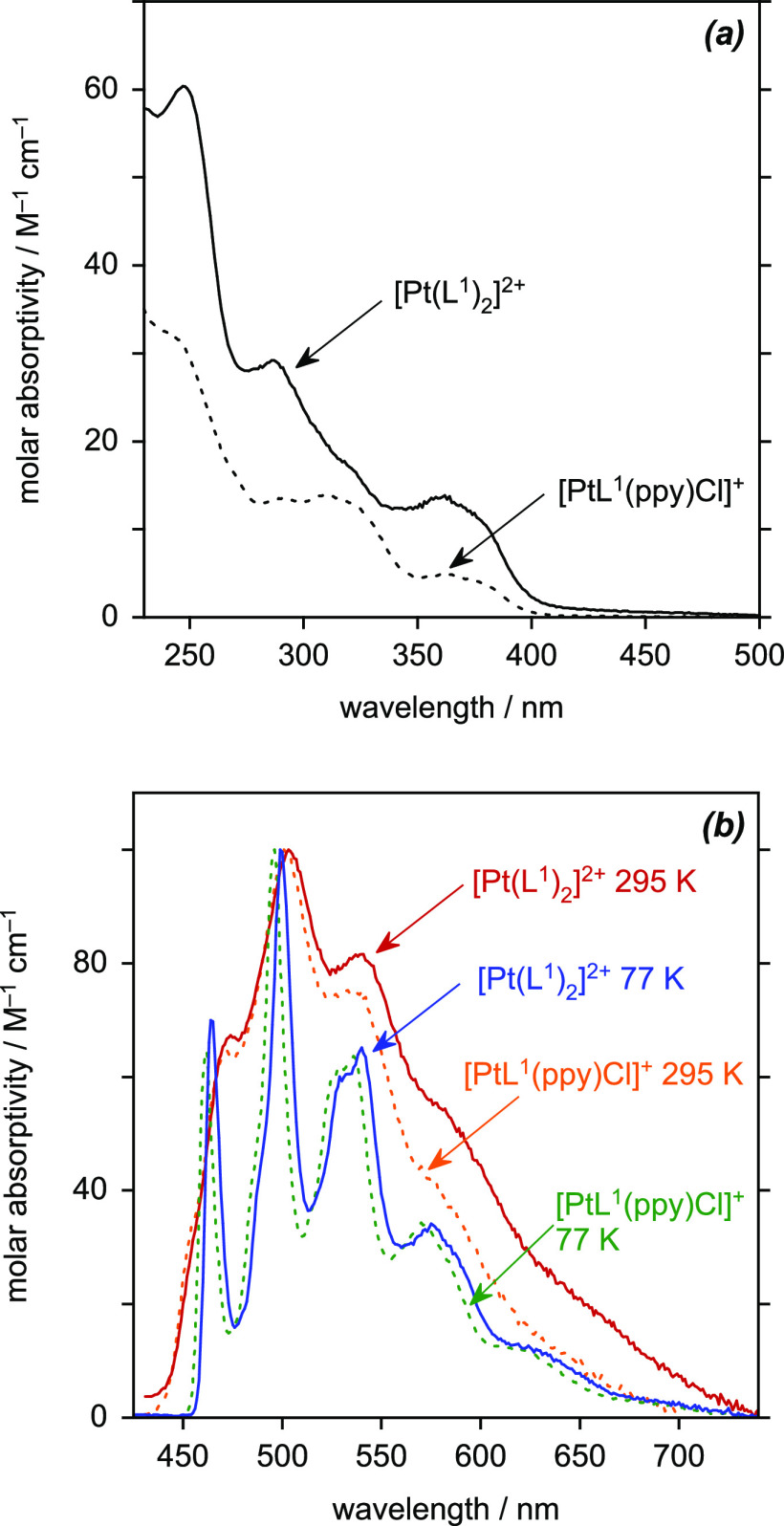
(a) UV–vis absorption spectrum of [Pt(L^1^)_2_](PF_6_)_2_ in MeCN at 295 K (solid line), together with that of [PtL^1^(ppy)Cl]PF_6_ from Figure 7 for comparison. (b) Emission spectra of [Pt(L^1^)_2_](PF_6_)_2_ in MeCN at 295 K (red line) and in butyronitrile at 77 K (blue line), together with the corresponding spectra of [PtL^1^(ppy)Cl]PF_6_ from Figure 8 for comparison (dashed orange and green lines, respectively).

The bis-tridentate complex [Pt(L^1^)_2_]^2+^ displays absorption and emission spectra that are very similar in profile to those of [PtL^1^(ppy)Cl]^+^ (Figure 9 and Table 5). Given that the lowest-energy singlet and triplet excited states in the latter were concluded above to be largely localized on the tridentate ligand, the similarity in the spectral profiles is probably to be anticipated. Evidently, the replacement of the monodentate chloride ligand by a pyridine ring has little effect on the excited state energy. Indeed, the main difference in the absorption spectra between the two complexes is the higher molar absorptivities across the wavelength range, reflecting the presence of the second tridentate ligand with its more extended conjugation. In emission, there is just a very small red-shift in the λ_max_ values of the homoleptic complex (a few nm only) and slightly more intense tail to long wavelengths. The quantum yield and lifetime are a little reduced, but otherwise, the optical properties are very similar.

## Concluding Discussion

Pt(IV) complexes of tridentate *NNC*-coordinating ligands L are shown by this study to be readily accessible, but only for the trichloro complexes, PtLCl_3_, where the remaining ligands are chlorides. The Pt(IV) ion remains relatively inert to further cyclometalation, such that quite forcing conditions are required to introduce a further bidentate *NC* or a second tridentate *NNC* ligand into the coordination sphere of the metal. Competitive reduction, coupled with the formation of trace side products and difficult purification, renders the yield of such products low. The situation is, indeed, somewhat reminiscent of the chemistry of the isoelectronic compound Ir(*NNN*)Cl_3_ (where *NNN* is a tridentate ligand such as terpyridine), where the introduction of additional ligands is similarly troublesome.^46^

From absorption spectroscopy, it is evident that the lowest-energy spin-allowed excited states are significantly increased in energy compared to the Pt(II) precursors, a trend that is fully consistent with the work of others on related tris-bidentate Pt(IV) complexes (e.g., Figure 1d,e).^14,15^ The influence of electron-donating substituents in the aryl ring in red-shifting the absorption can be interpreted in terms of a likely ^1^[d_Pt_|π_Ar_ → π*_NN_] character, just as in the Pt(II) complexes, but with the occupied orbitals at lower energy thanks to the higher charge on the metal. The lack of detectable luminescence from the PtL^*n*^Cl_3_ complexes can probably be attributed to the combination of (i) poor mixing of metal and ligand orbitals and hence inefficient spin–orbit coupling, such that the T_1_ → S_0_ phosphorescence process remains highly spin-forbidden; and (ii) a weak ligand field such that highly deactivating d–d excited states probably lie low in energy, serving as a thermally accessible deactivation pathway for the ^3^CT state. The introduction of a cyclometalating *NC* ligand in place of two of the chloride ligands does not significantly change the nature of the lowest-energy excited state, based on the similarity of the absorption spectra, but it does allow phosphorescence to be observed. By analogy with principles established from isoelectronic iridium(III) chemistry,^47^ this influence of the additional cyclometalating ligand is likely due to the combined effects of it serving to raise the energy of metal-centered orbitals thus promoting the necessary mixing with ligand orbitals, and increasing the strength of the ligand field experienced by the metal, ensuring that d–d states are displaced to higher energy. The few examples reported here show that some control over the emission energy is achieved through simple modification of the aryl ring. Despite the synthetic difficulties, these results could open up new opportunities in the chemistry of Pt(IV) with tridentate ligands.

## Experimental Details

### General

Reagents were obtained from commercial sources and used without further purification unless stated otherwise. All solvents used in preparative work were at least Analar grade, and water was purified using the Purite_STILL_ plus system. Dry solvents were obtained from HPLC grade solvent that had been passed through a Pure Solv 400 solvent purification system and stored over activated 3 or 4 Å molecular sieves. For procedures involving dry solvent, glassware was oven-dried at 110 °C prior to use. Reactions requiring an inert atmosphere were carried out using Schlenk-line techniques under an atmosphere of nitrogen. Thin-layer chromatography (TLC) was carried out using silica plates (MerckArt 5554) and visualized by UV radiation at 254 and/or 365 nm. NMR spectra were recorded on a Bruker Avance-400 spectrometer. Two-dimensional spectra (COSY, NOESY, HSQC, and HMBC) were acquired on a Varian VNMRS-600 (600 MHz) or Varian VNMRS-700 (700 MHz) instrument. Chemical shifts (δ) are in ppm, referenced to residual protio-solvent resonances, and coupling constants are given in hertz. Electrospray ionization mass spectral data (positive and negative modes) were obtained on an SQD mass spectrometer interfaced with an Acquity UPLC system with acetonitrile as the carrier solvent. Spectra acquired using an atmospheric solids atomization probe were recorded on a Waters Xevo QToF mass spectrometer.

### Synthetic Procedures and Characterization

The detailed synthesis and characterization of the proligands and of the PtL^*n*^Cl precursor complexes are described in the Supporting Information. Briefly, the proligands HL^1–5^ and the *NC* proligands 4-MeOppyH and 3-MeOppyH were synthesized through Suzuki cross-coupling reactions, while HL^6^ was synthesized through a Stille cross-coupling reaction. The PtL^*n*^Cl complexes were then obtained by refluxing the tridentate proligands with K_2_PtCl_4_ in acetic acid. While PtL^1^Cl and PtL^6^Cl were established from the work of Constable et al.,^25^ and PtL^2^Cl was explored by Che and co-workers,^30^ PtL^2–4^Cl were used solely en route to their Pt—C≡C—Ar acetylide adducts, and there is no previous report of PtL^5^Cl. We provide data for all six compounds in the Supporting Information. The synthesis and characterization of PtL^1^Cl_3_ and [PtL^1^(ppy)Cl]PF_6_ are described below, as representative examples of these two classes of new compounds, together with [Pt(L^1^)_2_](PF_6_)_2_. Details for all other compounds are given in the Supporting Information.

### PtL^1^Cl_3_

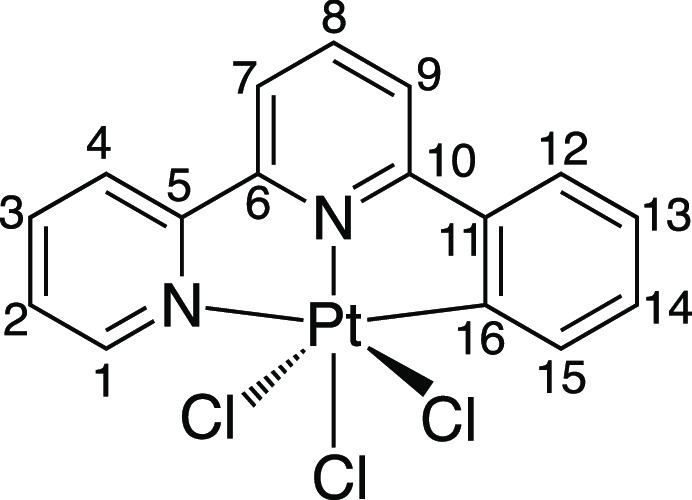



Chlorine gas, generated by dropwise addition of HCl to solid KMnO_4_, was bubbled through a solution of PtL^1^Cl (250 mg, 0.54 mmol) in CHCl_3_ for 10 min, with the partial exclusion of light. A pale-colored precipitate formed, and the mixture was left to stir for a further 1 h. The solid was separated and extracted into CH_2_Cl_2_ and the solvent removed under reduced pressure to yield the product as a pale-yellow solid (189 mg, 90% yield). ^1^H NMR (599 MHz, DMSO-*d*_6_): δ_H_ 9.10 (dd, *J* = 5.0, 1.5, 1H, H^1^), 8.85 (d, *J* = 8.0, 1H, H^4^), 8.63 (d, *J* = 8.0, 1H, H^7^), 8.49–8.44 (m, 2H, H^3^, H^9^), 8.42 (t, *J* = 8.0, 1H, H^8^), 8.06 (dd, *J* = 7.5, 5.5, 1H, H^2^), 7.97 (dd, *J* = 8.0, 1.5, 1H, H^12^), 7.59 (dd, ^3^*J*^195^_Pt–__^1^H_ ≈ 27, *J* = 8.0, 1.0, 1H, H^15^), 7.38 (td, *J* = 7.5, 1.5, 1H, H^14^), 7.28 (td, *J* = 7.5, 1.0, 1H, H^13^). ^13^C NMR (151 MHz, DMSO-*d*_6_): δ_C_ 148.8 (C^1^), 144.1 (C^8^), 142.4 (C^3^ or C^9^), 133.0 (C^14^), 132.6 (C^15^), 129.6 (C^2^), 127.7 (C^12^), 127.0 (C^13^), 126.4 (C^4^), 123.1 (C^3^, C^9^, or C^7^). HRMS (ES^+^) *m*/*z* = 536.0192 [M – Cl + MeCN]^+^; calcd for [C_18_H_14_N_3_Cl_2_^194^Pt]^+^ 536.0212.

### [PtL^1^(ppy)Cl]PF_6_

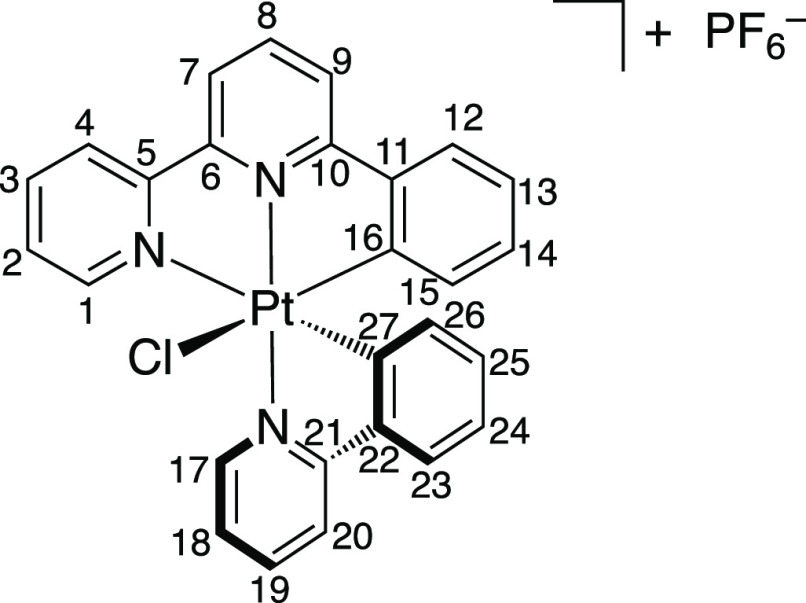



A mixture of PtL^1^Cl_3_ (25 mg, 0.05 mmol), 2-phenylpyridine (6.7 μL, 0.05 mmol), and silver(I) trifluoromethanesulfonate (24 mg, 0.09 mmol) was suspended in dry toluene (1.5 mL) in a dry Schlenk flask. The mixture was degassed by three freeze–pump–thaw cycles and heated at 125 °C for 18 h, with the partial exclusion of light. The resulting precipitate was isolated by centrifugation; washed successively with toluene (5 mL), hexane (5 mL), and diethyl ether (5 mL); and extracted into DCM. The solvent was removed under reduced pressure, the residue dissolved in acetone, and the resulting solution added dropwise to a saturated aqueous solution of KPF_6_ (20 mL) to precipitate the hexafluorophosphate of the complex. The precipitate was isolated by centrifugation, washed with water (10 mL), and dried. The complex was purified by two successive recrystallizations from acetone/diethyl ether, yielding a pale-yellow solid (5 mg, 14% yield). Crystals suitable for X-ray diffraction were obtained by layering a DCM solution of the complex with Et_2_O. ^1^H NMR (599 MHz, acetone-*d*_6_): δ_H_ 10.01 (dt, ^3^*J*^195^_Pt–^1^H_ ≈ 27, *J* = 6.0, 1.0, 1H, H^17^), 8.89 (dt, *J* = 8.5, 1.0, 1H, H^4^), 8.79–8.75 (m, 1H, H^7^), 8.71 (t, *J* = 8.0, 1H, H^8^), 8.65–8.57 (m, 3H, H^9^, H^20^, H^18^), 8.45 (td, *J* = 8.0, 1.5, 1H, H^3^), 8.41 (ddd, ^3^*J*^195^_Pt–_^1^_H_ ≈ 15, *J* = 5.5, 1.5, 0.5, 1H, H^1^), 8.06–7.97 (m, 3H, H^19^, H^12^, H^23^), 7.80 (ddd, *J* = 7.5, 5.5, 1.0, 1H, H^2^), 7.25 (dtd, *J* = 10.5, 7.5, 1.0, 2H, H^13^, H^24^), 7.13–7.06 (m, 1H, H^19^), 7.01 (ddd, *J* = 8.0, 7.5, 1.5, 1H, H^25^), 6.34 (dd, ^3^*J*^195^_Pt–__^1^H_ ≈ 33, *J* = 8.0, 1.0, 1H, H^26^), 6.22 (dd, ^3^*J*^195^_Pt–__^1^H_ ≈ 32, *J* = 8.0, 1.0, 1H, H^15^). HRMS (ES^+^) *m*/*z* = 614.0903 [M]^+^; calcd for [C_27_H_19_N_3_Cl^194^Pt]^+^ 614.0894.

### [Pt(L^1^)_2_](PF_6_)_2_

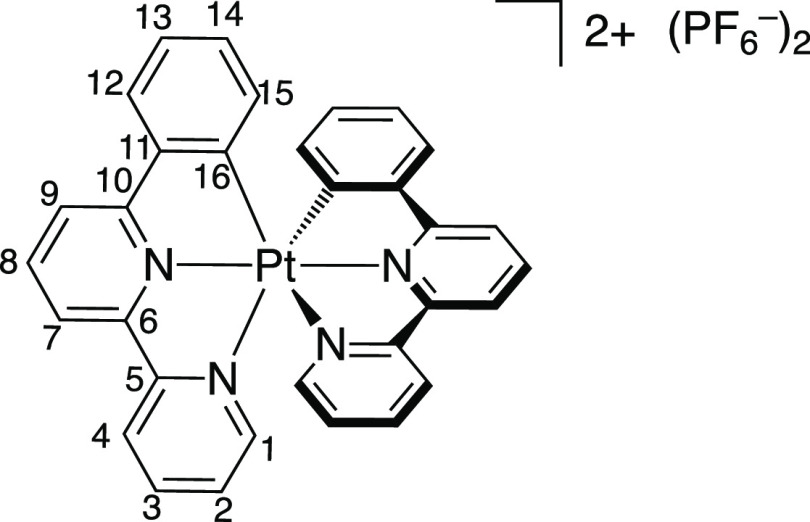



A mixture of PtL^1^Cl_3_ (30 mg, 0.06 mmol), 6-phenyl-2,2′-bipyridine (13 mg, 0.06 mmol), and silver(I) trifluoromethanesulfonate (43 mg, 0.17 mmol) was suspended in dry toluene (3 mL) in a dry Schlenk flask. The mixture was degassed by three freeze–pump–thaw cycles and heated at 125 °C overnight, with the partial exclusion of light. The resulting precipitate was isolated by centrifugation; washed successively with toluene (5 mL), DCM (5 mL), and diethyl ether (5 mL); and extracted into acetone. The resulting solution was then concentrated to a smaller volume and added dropwise to a saturated aqueous solution of KPF_6_ (20 mL) to precipitate the hexafluorophosphate of the complex. The precipitate was isolated by centrifugation, washed with water (10 mL), and dried. The crude product was purified by column chromatography on alumina, gradient elution from MeCN to MeCN/H_2_O/KNO_3_ (aq, satd.) (80:19.5:0.5, v/v), followed by two recrystallizations from acetone/diethyl ether, yielding the product as a pale-green solid (7 mg, 13% yield). ^1^H NMR (599 MHz, acetone-*d*_6_): δ_H_ 9.03–9.00 (m, 1H, H^4^), 8.98 (dd, *J* = 8.0, 1.0, 1H, H^7^), 8.89–8.84 (m, 1H, H^8^), 8.83–8.79 (m, 1H, H^9^), 8.63 (ddd, ^3^*J*^195^_Pt–__^1^H_ ≈ 12, *J* = 5.5, 1.5, 1.0, 1H, H^1^), 8.46 (td, *J* = 8.0, 1.5, 1H, H^3^), 8.15 (dd, *J* = 8.0, 1.5, 1H, H^12^), 7.75 (ddd, *J* = 7.5, 5.5, 1.5, 1H, H^2^), 7.33 (td, *J* = 7.5, 1.0, 1H, H^13^), 7.14–7.07 (m, 1H, H^14^), 6.39 (dd, ^3^*J*^195^_Pt–__^1^H_ ≈ 34, *J* = 8.0, 1.0, 1H, H^15^). ^13^C NMR (151 MHz, acetone-*d*_6_): δ_C_ 161.5 (C^10^), 155.3 (C^5^), 152.2 (C^6^), 150.6 (C^1^), 144.9 (C^8^), 143.5 (C^11^), 143.0 (C^3^), 134.6 (C^16^), 133.3 (C^14^), 129.5 (C^2^), 128.5 (C^15^), 128.4 (C^12^), 128.1 (C^13^), 127.0 (C^4^), 124.1 (C^7^), 124.0 (C^9^). HRMS (ES^+^) *m*/*z* = 328.0712 [M]^2+^; calcd for [C_32_H_20_N_4_^194^Pt]^2+^ 328.0736.

### X-ray Crystallography

The X-ray single crystal data have been collected at a temperature of 120.0(2) K using MoKα radiation (λ = 0.71073 Å) on a Bruker D8 Venture (Photon III MM C7 CPAD detector, IμS-microsource, focusing mirrors, or Photon III MM C14 CPAD detector, IμS–III-microsource, focusing mirrors) 3-circle diffractometer equipped with a Cryostream (Oxford Cryosystems) open-flow nitrogen cryostat. All structures were solved by various direct methods and refined by full-matrix least-squares on *F*^2^ for all data using Olex2^48^ and SHELXTL^49^ software. All nondisordered nonhydrogen atoms were refined in anisotropic approximation: hydrogen atoms were placed in the calculated positions and refined in riding mode. Some solvent molecules in the structures of [PtL^4^(4-MeOppy)Cl]^+^ and [PtL^5Cl^(ppy)Cl]^+^ could not be reliably modeled and refined, and their contribution to the structural factors was taken into account by applying the MASK procedure of the Olex2 program package. Crystal data and parameters of refinement are listed in the Supporting Information, Tables S2.2–2.6. Crystallographic data for all structures have been deposited with the Cambridge Crystallographic Data Centre as supplementary publications CCDC 2219176–2219189.

### Solution-State Photophysics

UV–vis absorption spectra were recorded on a Biotek Instruments Uvikon XS spectrometer operated with LabPower software. Emission spectra were acquired on a Jobin Yvon Fluoromax-2 spectrometer equipped with a Hamamatsu R928 photomultiplier tube. All samples were contained within 1 cm path length quartz cuvettes modified for connection to a vacuum line. Degassing was achieved by at least three freeze–pump–thaw cycles while connected to the vacuum manifold: final vapor pressure at 77 K was <5 × 10^–2^ mbar. Emission was recorded at 90° to the excitation source, and spectra were corrected after acquisition for dark count and for the spectral response of the detector. The quantum yields were determined relative to an aqueous solution of [Ru(bpy)_3_]Cl_2_, for which Φ_lum_ = 0.04.^50^ Emission spectra at 77 K were recorded in 4 mm diameter tubes held within a liquid-nitrogen-cooled quartz Dewar, using the same spectrometer.

The luminescence lifetimes τ in solution at 295 K were measured by time-correlated single-photon counting, for τ < 10 μs, using an EPL405 pulsed-diode laser as excitation source (405 nm excitation, pulse length of 60 ps, repetition rate 20 kHz, or faster for shorter lifetimes). The emission was detected at 90° to the excitation source, after passage through a monochromator, using an R928 PMT thermoelectrically cooled to −20 °C. The longer luminescence lifetimes ≥10 μs of [PtL^1^(ppy)Cl]^+^ and [Pt(L^1^)_2_]^2+^ at 295 K, and of all the complexes at 77 K, were recorded using the same detector operating in multichannel scaling mode, following excitation with a microsecond pulsed xenon lamp. For all measurements, the decays were much longer than the instrument response, and data were analyzed by least-squares tail fitting to the following equation:
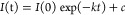
where *I*(t) is the intensity of light detected at time *t*, *k* is the first-order rate constant for decay (*k* = 1/τ), and *c* is a constant reflecting the intrinsic “dark count” during the measurement. The quality of the fit was assessed by referring to the residuals (difference between fit and experimental data). In most cases, the data fit well to the above equation (see Supporting Information), with no convincing improvement upon introducing additional components.

## Supporting Information Available

Synthetic and characterization data for all other proligands and complexes; additional figures showing molecular structures and packing in the crystals; tables of crystal data and refinement parameters; additional emission spectra; time-resolved luminescence data and least-squares fits to monoexponential decay; and ^1^H and ^13^C NMR spectra (PDF)
