# Supplementary material for: Platinum(IV) Complexes with Tridentate, NNC-Coordinating Ligands: Synthesis, Structures, and Luminescence
Source: Inorg Chem. 2023 Jan 16;62(4):1306–22. doi: 10.1021/acs.inorgchem.2c04116 (PMC9890496; doi:10.1021/acs.inorgchem.2c04116)
Supplement: Supplementary file 1 — ic2c04116_si_001.pdf [file ic2c04116_si_001.pdf]

# Supporting Information

# Platinum(IV) complexes with tridentate, *NNC*-coordinating ligands: synthesis, structures, and luminescence

*Yana M. Dikova, Dmitry S. Yufit and J. A. Gareth Williams**

*Department of Chemistry, Durham University, Durham, DH1 3LE, U.K.*
** E-mail: j.a.g.williams@durham.ac.uk*

**Section 1:** Synthetic procedures and characterisation for proligands and complexes not presented in the main text........................................................ **page 2**

**Section 2:** X-ray crystallography details and additional figures of molecular and crystal structures ..................................................................... **page 15**

**Section 3:** Additional absorption / emission spectra and luminescence decay data...... **page 22**

**Section 4:** $^{1}$H and $^{13}$C NMR spectra ........................................................... **page 35**

## Section 1 Synthetic procedures and characterisation for proligands and complexes not presented in the main text

Generic experimental information, including details of the instrumentation employed and data for representative examples of the new families of complex {*i.e.*, $PtL^1Cl_3$, $[PtL^1(ppy)Cl]PF_6$ and $[Pt(L^1)_2](PF_6)_2$}, are given in the main text in the Experimental Section. For the compound characterisation details given in this Section, NMR coupling constants are given in Hz and chemical shifts are in ppm, referenced using residual protio solvent resonances for $^1H$ spectra, or to the $^{13}C$ of $CDCl_3$ in the case of $^{13}C$ spectra.

### 6-Bromo-2,2'-bipyridine, bpy-Br

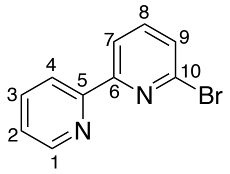


This key precursor was prepared by a Stille coupling. 2,6-Dibromopyridine (1 g, 4.22 mmol) and 2-(tributyltin)pyridine (1.54 g, 4.22 mmol) were dissolved in toluene (20 mL) and degassed by three freeze-pump-thaw cycles. The catalyst $Pd(PPh_3)_4$ (244 mg, 0.22 mmol) was added under nitrogen and the resulting yellow solution was heated at reflux for 18 h. After cooling, a saturated aqueous solution of KF (10 mL) was added and the mixture was stirred for 30 min. The precipitate was filtered off and then the solvent was removed under reduced pressure. The crude product was extracted into $CH_2Cl_2$ (3 × 15 mL), washed with a 5% aqueous solution of $NaHCO_3$ (3 × 15 mL), and dried over anhydrous $MgSO_4$. The solvent was then removed under reduced pressure and the residue purified by column chromatography on silica, gradient elution from hexane to hexane / ethyl acetate (60 : 40 v/v), to yield a white solid (616 mg, 68 %). $^1H$ NMR (400 MHz, $CDCl_3$) $\delta_H$ = 8.69 ($J$ = 5.0, 2.0, 1.0, 1H, $H^1$), 8.45 – 8.39 (m, 2H, $H^4$ and $H^7$), 7.85 (td, $J$ = 7.5, 2.0, 1H, $H^3$), 7.70 (t, $J$ = 8.0, 1H, $H^8$), 7.52 (dd, $J$ = 8.0, 1.0, 1H, $H^9$), 7.36 (ddd, $J$ = 7.5, 5.0, 1.0, 1H, $H^2$). MS $ESI^+$ *m/z* = 236 $[M+H]^+$.

### General procedure for the Suzuki cross-couplings to prepare the *NNC* proligands $HL^{1–5}$

A mixture of 6-bromo-2,2'-bipyridine, the requisite boronic acid (1 equiv.), $Na_2CO_3$ (8 equiv.), water (approximately 1 mL per mmol of $Na_2CO_3$), and dimethoxyethane (the same volume as the water) was degassed in a Schlenk by three freeze-pump-thaw cycles, and the vessel then back-filled with nitrogen gas. The catalyst $Pd(PPh_3)_4$ (0.05 equiv.) was added under a gentle nitrogen flow,

and the mixture was then heated at reflux temperature under nitrogen for 72 h. After cooling, water was added and the crude product was extracted into $CH_2Cl_2$. The organic phase was dried over anhydrous $MgSO_4$, filtered, and the solvent was removed under reduced pressure. The residue was purified by column chromatography on silica with a gradient elution of hexane and ethyl acetate.

**6-Phenyl-2,2'-bipyridine, HL$^1$**

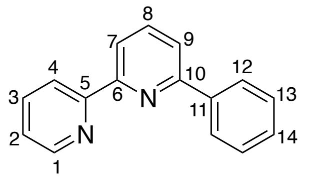


This compound was prepared from **bpy-Br** (615 mg, 2.62 mmol) and phenylboronic acid (319 mg, 2.62 mmol) using $Na_2CO_3$ (2.218 g, 20.93 mmol) and $Pd(PPh_3)_4$ (151 mg, 0.131 mmol), according to the general procedure described above. The product was obtained as an off-white solid (419 mg, 71% yield), $R_f$ = 0.5 in hexane / EtOAc, 8 : 2 v/v. $^1$H NMR (400 MHz, $CDCl_3$) $\delta_H$ = 8.72 (ddd, $J$ = 5.0, 2.0, 1.0, 1H), 8.67 (dt, $J$ = 8.0, 1.0, 1H), 8.40 (dd, $J$ = 8.0, 1.0, 1H), 8.22 – 8.14 (m, 2H), 7.96 – 7.85 (m, 2H), 7.81 (dd, $J$ = 8.0, 1.0, 1H), 7.57 – 7.51 (m, 2H), 7.50 – 7.43 (m, 1H), 7.36 (ddd, $J$ = 7.5, 5.0, 1.0, 1H). MS ESI$^+$ $m/z$ = 232 (100%) $[M + H]^+$.

**6-[4-(Trifluoromethyl)phenyl]-2,2'-bipyridine, HL$^2$**

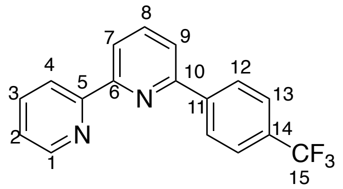


**HL$^2$** was prepared from **bpy-Br** (300 mg, 1.28 mmol) and 4-(trifluoromethyl)phenylboronic acid (244 mg, 1.28 mmol) using $Na_2CO_3$ (1.082 g, 10.21 mmol) and $Pd(PPh_3)_4$ (74 mg, 0.06 mmol), according to the general procedure above. The product was obtained as an off-white solid (334 mg, 87% yield), $R_f$ = 0.4 in hexane / EtOAc, 8 : 2 v/v. $^1$H NMR (400 MHz, $CDCl_3$) $\delta_H$ = 8.73 (ddd, $J$ = 5.0, 2.0, 1.0, 1H), 8.64 (dt, $J$ = 8.0, 1.0, 1H), 8.47 (dd, $J$ = 8.0, 1.0, 1H), 8.32 – 8.25 (m, 2H), 7.96 (t, $J$ = 8.0, 1H), 7.89 (td, $J$ = 7.5, 2.0, 1H), 7.83 (dd, $J$ = 8.0, 1.0, 1H), 7.81 – 7.77 (m, 2H), 7.37 (ddd, $J$ = 7.5, 5.0, 1.0 Hz, 1H). MS ESI$^+$ $m/z$ = 300 (100%) $[M + H]^+$.

**6-(4-Methylphenyl)-2,2'-bipyridine, HL$^3$**

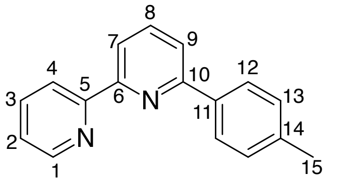

**HL$^3$** was prepared from **bpy-Br** (150 mg, 0.64 mmol) and *p*-tolylboronic acid (87 mg, 0.64 mmol) using $Na_2CO_3$ (541 g, 5.10 mmol) and $Pd(PPh_3)_4$ (37 mg, 0.03 mmol), according to the general procedure above. The product was obtained as an off-white solid (135 mg, 86% yield), $R_f$ = 0.5 in hexane / EtOAc, 8 : 2, v/v. $^1$H NMR (400 MHz, $CDCl_3$) $\delta_H$ = 8.72 (d, *J* = 5.0, 1H), 8.67 (d, *J* = 8.0, 1H), 8.37 (d, *J* = 8.0, 1H), 8.08 (d, *J* = 8.0, 2H), 7.88 (q, *J* = 8.0, 2H), 7.77 (d, *J* = 8.0, 1H), 7.34 (d, *J* = 8.0, 3H), 2.46 (s, 3H). MS ESI$^+$ *m/z* = 247 (100%) $[M + H]^+$.

**6-(4-Methoxyphenyl)-2,2'-bipyridine, HL$^4$**

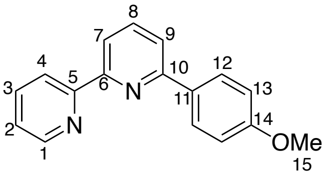

**HL$^4$** was prepared from **bpy-Br** (83 mg, 0.35 mmol) and 4-methoxyphenylboronic acid (54 mg, 0.35 mmol) using $Na_2CO_3$ (300 g, 2.83 mmol) and $Pd(PPh_3)_4$ (20 mg, 0.02 mmol), according to the general procedure above. The product was obtained as an off-white solid (79 mg, 85% yield), $R_f$ = 0.3 in hexane / EtOAc, 8 : 2, v/v. $^1$H NMR (400 MHz, $CDCl_3$) $\delta_H$ = 8.72 (ddd, *J* = 5.0, 2.0, 1.0, 1H), 8.65 (dt, *J* = 8.0, 1.0, 1H), 8.34 (dd, *J* = 8.0, 1.0, 1H), 8.19 – 8.10 (m, 2H), 7.92 – 7.83 (m, 2H), 7.74 (dd, *J* = 8.0, 1.0, 1H), 7.35 (ddd, *J* = 7.5, 5.0, 1.0, 1H), 7.10 – 7.02 (m, 2H), 3.91 (s, 3H). MS ESI$^+$ *m/z* = 262 (100%) $[M + H]^+$.

**6-(3-Methoxyphenyl)-2,2'-bipyridine, HL$^5$**

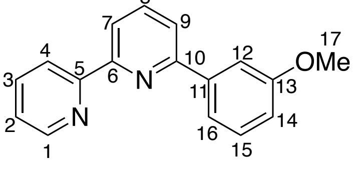

**HL$^5$** was prepared from **bpy-Br** (250 mg, 1.06 mmol) and 3-methoxyphenylboronic acid (162 mg, 1.06 mmol) using $Na_2CO_3$ (902 g, 8.57 mmol) and $Pd(PPh_3)_4$ (61 mg, 0.05 mmol), according to the general procedure above. The product was obtained as an off-white solid (197 mg, 66% yield), $R_f$ = 0.3 in hexane / EtOAc, 9 : 1, v/v. $^1$H NMR (400 MHz, $CDCl_3$) $\delta_H$ = 8.72 (ddd, *J* = 5.0, 2.0, 1.0, 1H), 8.66 (dt, *J* = 8.0, 1.0, 1H), 8.40 (dd, *J* = 8.0, 1.0, 1H), 7.96 – 7.83 (m, 2H), 7.83 – 7.76 (m,

2H), 7.73 (ddd, $J$ = 7.5, 1.5, 1.0, 1H), 7.45 (t, $J$ = 8.0, 1H), 7.35 (ddd, $J$ = 7.5, 5.0, 1.0, 1H), 7.02 (ddd, $J$ = 8.0, 2.5, 1.0, 1H), 3.95 (s, 3H). MS ESI$^+$ $m/z$ = 262 (100%) $[M + H]^+$.

**6-(Thien-2-yl)-2,2'-bipyridine, HL$^6$ by Stille cross-coupling**

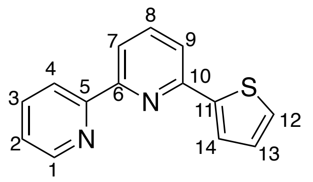


This compound was prepared by a Stille coupling of **bpy-Br** (200 mg, 0.85 mmol) and 2-(tributylstannyl)thiophene (270 mL, 0.85 mmol). A mixture of the two compounds in toluene (10 mL) was degassed by three freeze-pump-thaw cycles. The catalyst $Pd(PPh_3)_4$ (30 mg, 0.04 mmol) was added under nitrogen and the resulting yellow solution was heated at reflux temperature for 18 h. After cooling, a saturated aqueous solution of KF (10 mL) was added and the mixture was stirred for 30 min. The precipitate was filtered off and then the solvent was removed under reduced pressure. The crude product was extracted into $CH_2Cl_2$ (3 × 15 mL), washed with a 5% aqueous solution of $NaHCO_3$ (3 × 15 mL), and dried over anhydrous $MgSO_4$. Upon removal of the solvent, the residue was purified by column chromatography on silica, using a gradient elution of hexane and ethyl acetate, to yield a white solid (178 mg, 88% yield), $R_f$ = 0.5 in hexane : EtOAc, 8 : 2, v/v. $^1$H NMR (400 MHz, $CDCl_3$) $\delta_H$ = 8.70 (ddd, J = 5.0, 2.0, 1.0, 1H), 8.61 (dt, J = 8.0, 1.0, 1H), 8.32 (dd, J = 7.5, 1.0, 1H), 7.88 (ddd, J = 8.0, 7.5, 2.0, 1H), 7.84 (t, J = 8.0, 1H), 7.72 – 7.66 (m, 2H), 7.44 (dd, J = 5.0, 1.0, 1H), 7.35 (ddd, J = 7.5, 5.0, 1.0, 1H), 7.16 (dd, J = 5.0, 3.5, 1H). MS ESI$^+$ $m/z$ = 238 (100%) $[M + H]^+$.

**Synthesis of the *NC* proligands 3-MeOppyH and 4-MeOppyH**

These two compounds were prepared by Suzuki cross-coupling using the same general procedure described above for the tridentate proligands, but starting from 2-bromopyridine in place of 6-bromo-2,2'bipyridine.

**2-(3-methoxyphenyl)pyridine, 3-MeOppyH**

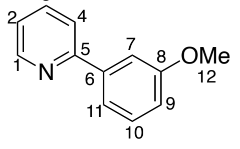


The title compound was prepared from 2-bromopyridine (61 mL, 0.63 mmol) and 3-methoxyphenyl-boronic acid (96 mg, 0.63 mmol) using $Na_2CO_3$ (537 mg, 5.06 mmol) and

$Pd(PPh_3)_4$ (37 mg, 0.032 mmol) in dimethoxyethane (5 mL) and water (5 mL), according to the general procedure. The product was obtained as a clear oil (54 mg, 67% yield), $R_f$ = 0.2 in hexane / EtOAc 8 : 2 v/v. $^1$H NMR (400 MHz, $CDCl_3$) $\delta_H$ = 8.72 (ddd, $J$ = 5.0, 2.0, 1.0, 1H), 7.81 – 7.72 (m, 2H), 7.61 (dd, $J$ = 2.5, 1.5, 1H), 7.57 (ddd, $J$ = 7.5, 1.5, 1.0, 1H), 7.41 (dd, $J$ = 8.0, 7.5, 1H), 7.30 – 7.22 (m, 2H), 7.00 (ddd, $J$ = 8.0, 2.5, 1.0, 1H), 3.93 (s, 3H). MS ESI$^+$ $m/z$ = 185 (100%) $[M + H]^+$.

**2-(4-Methoxyphenyl)pyridine, 4-MeOppyH**

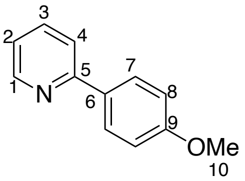


This compound was prepared from 2-bromopyridine (82 mL, 0.86 mmol) and 4-methoxyphenylboronic acid (135 mg, 0.86 mmol) using $Na_2CO_3$ (697 g, 6.54 mmol) and $Pd(PPh_3)_4$ (50 mg, 0.42 mmol) in dimethoxyethane (7 mL) and water (7 mL), according to the general procedure. The product was obtained as a clear oil (70 mg, 62% yield), $R_f$ = 0.2 in hexane / EtOAc, 8 : 2 v/v). $^1$H NMR (400 MHz, $CDCl_3$) $\delta_H$ = 8.68 (ddd, $J$ = 5.0, 2.0, 1.0, 1H), 8.02 – 7.93 (m, 2H), 7.76 – 7.67 (m, 2H), 7.19 (ddd, $J$ = 7.0, 5.0, 1.0, 1H), 7.05 – 6.99 (m, 2H), 3.89 (s, 3H) ESI$^+$ m/z 185 (100%) $[M + H]^+$.

**General procedure for the synthesis of $Pt^{II}$(*NNC*)Cl complexes**

A mixture of the proligand $HL^n$ and $K_2PtCl_4$ (1.14 equiv.) was dissolved in acetic acid (approx. 13 mL per 100 mg of proligand), degassed by three freeze-pump-thaw cycles, and then heated to reflux temperature under nitrogen for 72 h. Upon cooling, water was added. The resulting precipitate was isolated, washed successively with water, MeOH and diethyl ether (approx. 5 mL of each), and extracted into $CH_2Cl_2$. The solvent was then removed under reduced pressure to yield the final product.

**$PtL^1Cl$**

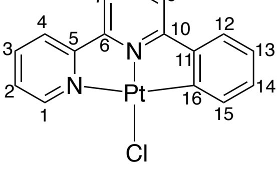


**$PtL^1Cl$** was prepared from $K_2PtCl_4$ (306 mg, 0.74 mmol) and 6-phenyl-2,2'-bipyridine (150 mg, 0.65 mmol), according to the general complexation procedure above, and obtained as a yellow solid

(250 mg, 83% yield). $^{1}$H NMR (400 MHz, DMSO-d$_6$) $\delta_H$ = 8.91 (ddd, $^{3}J_{^{195}Pt-^{1}H} \approx 19$, $J$ = 5.5, 1.5, 1.0, 1H), 8.52 (dt, $J$ = 8.0, 1.0, 1H), 8.36 (td, $J$ = 8.0, 1.7 Hz, 1H), 8.23 – 8.10 (m, 2H), 8.00 – 7.89 (m, 2H), 7.62 (dd, $J$ = 7.5, 1.5, 1H), 7.51 (dd, $^{3}J_{^{195}Pt-^{1}H} \approx 43$, $J$ = 7.5, 1.5, 1H), 7.16 (td, $J$ = 7.5, 1.5 Hz, 1H), 7.09 (td, $J$ = 7.5, 1.5 Hz, 1H). MS ASAP$^+$ $m/z$ = 468 (100%), [M – Cl$^-$ + MeCN]$^+$.

**PtL$^2$Cl**

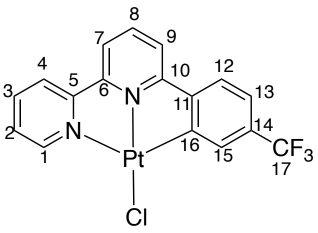


**PtL$^2$Cl** was prepared from $K_2PtCl_4$ (142 mg, 0.34 mmol) and 6-[4-(trifluoromethyl)phenyl]-2,2'-bipyridine (90 mg, 0.30 mmol), according to the general procedure above, and obtained as a yellow solid (140 mg, 88% yield). $^{1}$H NMR (599 MHz, DMSO-d$_6$) $\delta_H$ = 8.82 (dt, $^{3}J_{^{195}Pt-^{1}H} \approx 15$, $J$ = 5.5, 1.5 Hz, 1H, H$^1$), 8.49 (dt, $J$ = 8.0, 1.0, 1H, H$^4$), 8.32 (td, $J$ = 8.0, 1.5, 1H, H$^3$), 8.23 (dd, $J$ = 8.0, 1.0 Hz, 1H, H$^7$), 8.16 (t, $J$ = 8.0, 1H, H$^8$), 8.03 (dd, $J$ = 8.0, 1.0, 1H, H$^9$), 7.89 (ddd, $J$ = 7.5, 5.5, 1.0, 1H, H$^2$), 7.77 (d, $J$ = 8.0, 1H, H$^{12}$), 7.71 (d, $^{3}J_{^{195}Pt-^{1}H} \approx 46$, $J$ = 2.0, 1H, H$^{15}$), 7.40 – 7.35 (m, 1H, H$^{13}$). $^{13}$C NMR (151 MHz, DMSO-d$_6$) $\delta_C$ = 148.9 (C$^1$), 141.3 (C$^3$), 140.5 (C$^8$), 130.0 (C$^{15}$), 128.9 (C$^2$), 125.3 (C$^{12}$), 124.6 (C$^4$), 121.3 (C$^7$), 121.1 (C$^9$), 121.1 (C$^{13}$). MS HRMS (ASAP$^+$) $m/z$ = 570.0461 [M + MeCN]$^+$, calcd for $[C_{19}H_{14}N_3Cl^{194}PtF_3]^+$ 570.0455.

**PtL$^3$Cl**

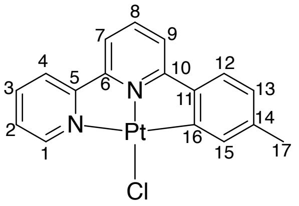


**PtL$^3$Cl** was prepared from $K_2PtCl_4$ (259 mg, 0.63 mmol) and 6-(4-methylphenyl)-2,2'-bipyridine (135 mg, 0.55 mmol), according to the general procedure above, and obtained as an orange solid (250 mg, 95% yield). $^{1}$H NMR (400 MHz, DMSO-d$_6$) $\delta_H$ = 8.89 (ddd, $J$ = 5.5, 1.5, 1.0, 1H), 8.49 (dt, $J$ = 8.0, 1.0, 1H), 8.38 – 8.29 (m, 1H), 8.17 – 8.03 (m, 2H), 7.94 – 7.85 (m, 2H), 7.48 (d, $J$ = 8.0, 1H), 7.30 (d, $^{3}J_{^{195}Pt-^{1}H} \approx 43$, $J$ = 2.0, 1H), 6.89 (ddd, $J$ = 8.0, 2.0, 1.0, 1H), 2.29 (s, 3H). MS ASAP$^+$ $m/z$ = 481 (100%), [M – Cl$^-$ + MeCN]$^+$.

**PtL⁴Cl**

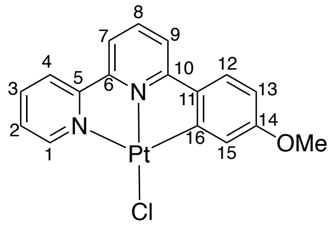


**PtL⁴Cl** was prepared from $K_2PtCl_4$ (88 mg, 0.21 mmol) and 6-(3-methoxyphenyl)-2,2'-bipyridine (49 mg, 0.19 mmol), according to the general procedure above, and obtained as an orange solid (70 mg, 77% yield). $^1$H NMR (599 MHz, DMSO-$d_6$) $\delta_H$ = 8.88 (dt, $J$ = 5.0, 1.5, 1H, $H^1$), 8.49 – 8.44 (m, 1H, $H^4$), 8.32 (td, $J$ = 8.0, 1.5, 1H, $H^3$), 8.08 – 8.01 (m, 2H, $H^7$ and $H^8$), 7.88 (ddd, $J$ = 7.5, 5.5, 1.5, 1H, $H^2$), 7.81 (dd, $J$ = 7.5, 1.5, 1H, $H^9$), 7.55 (d, $J$ = 8.5, 1H, $H^{12}$), 7.02 (d, $^3J_{^{195}Pt-^1H} \approx 50$, $J$ = 2.5, 1H, $H^{15}$), 6.64 (dd, $J$ = 8.5, 2.5, 1H, $H^{13}$), 3.75 (s, 3H, $H^{17}$). $^{13}$C NMR (151 MHz, DMSO-$d_6$) $\delta_C$ = 148.8 ($C^1$), 141.1 ($C^3$), 140.0 ($C^8$), 128.7 ($C^2$), 127.2 ($C^{12}$), 124.3 ($C^4$), 119.5 ($C^{15}$), 119.1 ($C^9$), 118.6 ($C^7$), 109.9, 55.4 ($C^{13}$). MS HRMS (ASAP$^+$) $m/z$ = 491.0422 $[M]^+$, calcd for $[C_{17}H_{14}N_2OCl^{194}Pt]^+$ 491.0421.

**PtL⁵Cl**

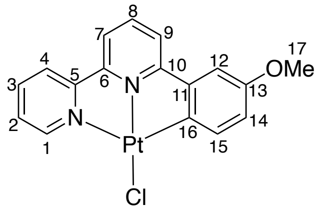


**PtL⁵Cl** was prepared from $K_2PtCl_4$ (235 mg, 0.57 mmol) and 6-(3-methoxyphenyl)-2,2'-bipyridine (130 mg, 0.50 mmol), according to the general procedure above, and obtained as an orange solid (200 mg, 82% yield). $^1$H NMR (599 MHz, DMSO-$d_6$) $\delta_H$ = 8.90 (d, $J$ = 5.5, 1H, $H^1$), 8.49 (d, $J$ = 8.0, 1H, $H^4$), 8.33 (dd, $J$ = 8.5, 7.0, 1H, $H^3$), 8.18 – 8.09 (m, 2H, $H^7$ and $H^8$), 8.00 (d, $J$ = 8.0, 1H, $H^9$), 7.92 – 7.87 (m, 1H, $H^2$), 7.34 (d, $^3J_{^{195}Pt-^1H} \approx 38$, $J$ = 8.5, 1H, $H^{15}$), 7.26 (d, $J$ = 3.0, 1H, $H^{12}$), 6.83 (dd, $J$ = 8.0, 3.0, 1H, $H^{14}$), 3.76 (s, 2H, $H^{17}$). $^{13}$C NMR (151 MHz, DMSO-$d_6$) $\delta_C$ = 148.5 ($C^1$), 140.9 ($C^3$), 140.1 ($C^8$), 135.4 ($C^{15}$), 128.8 ($C^2$), 124.4 ($C^4$), 120.0 ($C^7$ and $C^9$, one peak but two cross-peaks in HSQC), 116.9 ($C^{14}$), 111.2 ($C^{12}$), 55.6 ($C^{17}$). MS HRMS (ASAP$^+$) $m/z$ = 491.0422 $[M]^+$, calcd for $[C_{17}H_{14}N_2OCl^{194}Pt]^+$ 491.0421.

**PtL⁶Cl**

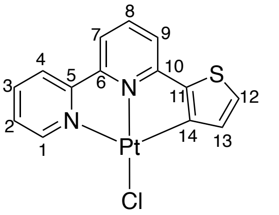


**PtL⁶Cl** was obtained from $K_2PtCl_4$ (72 mg, 0.17 mmol) and 6-(thiophen-2-yl)-2,2'-bipyridine (36 mg, 0.15 mmol), according to the general procedure above, and obtained as an orange solid (40 mg, 57% yield). $^1$H NMR (599 MHz, DMSO-d$_6$) $\delta_H$ = 8.84 (d, $^3J_{^{195}Pt-^1H} \approx 20$, $J$ = 5.5, 1H, H$^1$), 8.45 (d, $J$ = 8.0, 1H, H$^4$), 8.33 (t, $J$ = 7.8, 1H, H$^3$), 7.95 (dd, $J$ = 4.6, 1.6, 2H, H$^7$ and H$^8$ or H$^9$), 7.89 (t, $J$ = 6.3, 1H, H$^2$), 7.83 – 7.78 (m, 1H, H$^{12}$), 7.56 – 7.51 (m, 1H, H$^8$ or H$^9$), 6.96 (dd, $^3J_{^{195}Pt-^1H} \approx 19$, $J$ = 4.6, 0.9, 1H, H$^{13}$). $^{13}$C NMR (151 MHz, DMSO-d$_6$) $\delta_C$ = 148.7 (C$^1$), 141.3 (C$^3$), 141.3 (C$^7$, C$^8$ or C$^9$),133.1 (C$^{13}$), 130.4 (C$^{12}$), 128.9 (C$^2$), 124.6 (C$^4$), 117.3 (C$^8$ or C$^9$), 116.8 (C$^7$, C$^8$ or C$^9$). MS ASAP$^+$ m/z 467 (100%) [M]$^+$.

**Synthesis of Pt$^{IV}$L$^n$Cl$_3$ complexes by oxidation of Pt$^{II}$L$^n$Cl with Cl$_2$ or PhICl$_2$**

**PtL²Cl₃**

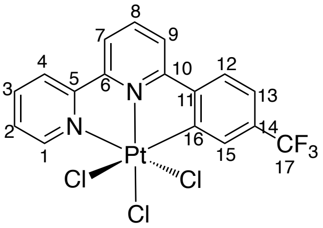


A mixture of PtL²Cl (138 mg, 0.26 mmol) and $PhICl_2$ (72 mg, 0.26 mmol) was stirred in $CHCl_3$ (approx. 25 mL) for 18 h, with partial exclusion of light. The solvent was removed under reduced pressure and the resulting residue washed with ether. The material was purified by column chromatography on silica, gradient elution with hexane / ethyl acetate, to yield the complex as a pale-yellow solid (78 mg, 50% yield). $^1$H NMR (599 MHz, DMSO-d$_6$) $\delta_H$ = 9.14 – 9.11 (m, 1H, H$^1$), 8.90 (dt, $J$ = 8.0, 1.0, 1H, H$^4$), 8.75 (dd, $J$ = 8.0, 1.0, 1H, H$^7$), 8.64 (dd, $J$ = 8.0, 1.0, 1H, H$^9$), 8.56 – 8.47 (m, 2H, H$^8$ and H$^3$), 8.26 (d, $J$ = 8.0, 1H, H$^{12}$), 8.09 (ddd, $J$ = 8.0, 5.5, 1.0, 1H, H$^2$), 7.86 – 7.82 (m, $^3J_{^{195}Pt-^1H} \approx 20$, 1H, H$^{15}$), 7.74 – 7.69 (m, 1H, H$^{13}$) $^{13}$C NMR (151 MHz, DMSO-d$_6$) $\delta_C$ / ppm 149.1 (C$^1$), 144.7 (C$^8$), 142.8 (C$^3$), 130.0 (C$^2$), 128.3 (C$^{12}$), 127.9 (C$^{15}$), 126.9 (C$^4$), 124.6 (C$^7$), 124.4 (C$^9$), 124.2 (C$^{13}$). HRMS (ASAP$^+$) m/z = 562.9792 [M – Cl]$^+$, calcd for [$C_{17}H_{10}N_2F_3Cl_2{}^{194}Pt$]$^+$ 562.9800.

**$PtL^3Cl_3$**

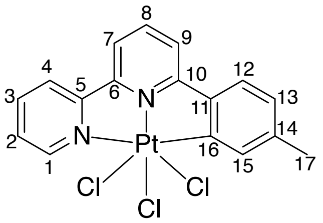


**$PtL^3Cl_3$** was obtained from $PtL^3Cl$ (231 mg, 0.42 mmol) by oxidation with $Cl_2$ as described for $PtL^1Cl_3$ in the main text. The product was extracted into $CH_2Cl_2$ and the solvent removed under reduced pressure to yield a pale-yellow solid (244 mg, 92% yield). $^1H$ NMR (599 MHz, DMSO-$d_6$) $\delta_H$ = 9.10 (ddd, $J$ = 5.0, 1.5, 0.5, 1H, $H^1$), 8.83 (dt, $J$ = 8.0, 1.0, 1H, $H^4$), 8.57 (dd, $J$ = 7.0, 2.0, 1H, $H^7$), 8.45 (td, $J$ = 8.0, 1.5, 1H, $H^3$), 8.41 – 8.34 (m, 2H, $H^9$ and $H^8$), 8.05 (ddd, $J$ = 7.5, 5.0, 1.0, 1H, $H^2$), 7.85 (d, $J$ = 8.0, 1H, $H^{12}$), 7.41 (dd, $^3J_{^{195}Pt-^1H} \approx 18$, $J$ = 1.5, 1.0, 1H, $H^{15}$), 7.11 (ddd, $J$ = 8.0, 1.5, 1.0, 1H, $H^{13}$), 2.40 (s, 3H, $H^{17}$). $^{13}C$ NMR (151 MHz, DMSO $d_6$) $\delta_C$ = 161.6 ($C^{10}$), 154.9 ($C^5$), 151.4 ($C^6$), 148.8 ($C^1$), 143.9 ($C^8$), 143.6 ($C^{14}$ or $C^{16}$), 142.4 ($C^3$), 141.4 ($C^{14}$ or $C^{16}$), 134.6 ($C^{11}$), 133.0 ($C^{15}$), 129.5 ($C^2$), 127.6 ($C^{13}$), 127.5 ($C^{12}$), 126.3 ($C^4$), 122.7 ($C^9$), 122.5 ($C^7$), 22.1 ($C^{17}$). HRMS (ASAP$^+$) $m/z$ = 543.9730 $[M]^+$, calcd for $[C_{17}H_{13}N_2Cl_3{}^{194}Pt]^+$ 543.9770.

**$PtL^4Cl_3$**

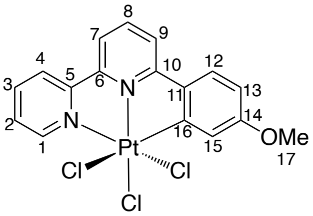


**$PtL^4Cl_3$** was obtained by oxidation of $PtL^4Cl$ (70 mg, 0.14 mmol) with $PhICl_2$ (39 mg, 0.14 mmol), as described above for $PtL^2Cl_3$. The product was purified by column chromatography on silica, gradient elution with hexane / ethyl acetate, to yield a pale-yellow solid (30 mg, 38% yield). $^1H$ NMR (599 MHz, DMSO-$d_6$) $\delta_H$ = 9.13 (ddd, $J$ = 5.5, 1.5, 0.5, 1H, $H^1$), 8.86 (dt, $J$ = 8.0, 1.0, 1H, $H^4$), 8.54 (dd, $J$ = 5.5, 3.5, 1H, $H^7$), 8.49 (td, $J$ = 8.0, 1.5, 1H, $H^3$), 8.39 – 8.34 (m, 2H, $H^8$ and $H^9$), 8.07 (ddd, $J$ = 7.5, 5.5, 1.0, 1H, $H^2$), 7.97 (d, $J$ = 8.5, 1H, $H^{12}$), 7.13 (d, $^3J_{^{195}Pt-^1H} \approx 24$, $J$ = 2.5, 1H, $H^{15}$), 6.93 (dd, $J$ = 8.5, 2.5, 1H, $H^{13}$), 3.90 (s, 3H, $H^{17}$). $^{13}C$ NMR (151 MHz, DMSO-$d_6$) $\delta_C$ = 148.9 ($C^1$), 143.6 ($C^9$), 142.4 ($C^3$), 129.5 ($C^2$), 129.2 ($C^{12}$), 126.2 ($C^4$), 122.3 ($C^8$), 121.7 ($C^7$), 117.6 ($C^{15}$), 112.3 ($C^{13}$), 56.1 ($C^{17}$). HRMS (ASAP$^+$) $m/z$ = 525.0016 $[M – Cl]^+$, calcd for $[C_{17}H_{13}Cl_2N_2O^{194}Pt]^+$ 525.0032.

**$PtL^{4Cl}Cl_3$**

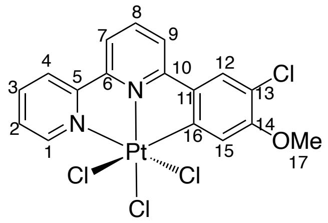


**$PtL^4Cl_3$** was obtained from $PtL^4Cl$ (65 mg, 0.13 mmol) by oxidation with $Cl_2$ as described for $PtL^1Cl_3$ in the main text. The product was extracted into $CH_2Cl_2$ and the solvent removed under reduced pressure. Recrystallization from $CH_2Cl_2$ / diethyl ether gave $PtL^{4Cl}Cl_3$ as a pale-yellow solid (70 mg, 89% yield). $^1$H NMR (599 MHz, DMSO-d$_6$) $\delta_H$ = 9.11 (ddd, $J$ = 5.5, 1.5, 0.5, 1H, $H^1$), 8.84 (dt, $J$ = 8.0, 1.0, 1H, $H^4$), 8.55 (dd, $J$ = 8.0, 1.0, 1H, $H^7$), 8.46 (td, $J$ = 8.0, 1.5, 1H, $H^3$), 8.42 (dd, $J$ = 8.5, 1.0, 1H, $H^9$), 8.37 (t, $J$ = 8.0, 1H, $H^8$), 8.22 (s, 1H, $H^{12}$), 8.05 (ddd, $J$ = 7.5, 5.5, 1.0, 1H, $H^2$), 7.26 (s, $^3J_{^{195}Pt-^1H}$ ≈ 21, 1H, $H^{15}$), 3.96 (s, 3H, $H^{17}$). $^{13}$C NMR (151 MHz, DMSO-d$_6$) $\delta_C$ = 149.0 ($C^1$), 144.0 ($C^8$), 142.6 ($C^3$), 129.7 ($C^2$), 128.7 ($C^{12}$), 126.5 ($C^4$), 122.9 ($C^9$), 122.4 ($C^7$), 115.1 ($C^{15}$), 57.1 ($C^{17}$). HRMS (ES$^+$) *m/z* = 599.9917 [M – Cl + MeCN]$^+$, calcd for $[C_{19}H_{15}N_3OCl_3{}^{194}Pt]^+$ 599.9907.

**$PtL^5Cl_3$**

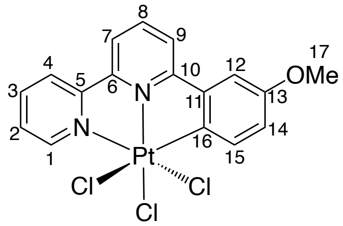


**$PtL^5Cl_3$** was obtained by oxidation of $PtL^5Cl$ (200 mg, 0.41 mmol) with $PhICl_2$ (224 mg, 0.82 mmol), as described above for $PtL^2Cl_3$. The product was extracted into $CH_2Cl_2$ to yield a pale-yellow solid (160 mg, 70% yield). $^1$H NMR (599 MHz, DMSO-d$_6$) $\delta_H$ = 9.10 (ddd, $J$ = 5.0, 1.5, 1.0, 1H, $H^1$), 8.86 (dt, $J$ = 8.0, 1.0, 1H, $H^4$), 8.63 (dd, $J$ = 8.0, 1.0, 1H, $H^7$), 8.54 (dd, $J$ = 8.0, 1.0, 1H, $H^9$), 8.50 – 8.40 (m, 2H, $H^3$ and $H^8$), 8.07 (ddd, $J$ = 7.5, 5.0, 1.0, 1H, $H^2$), 7.65 (d, $J$ = 3.0, 1H, $H^{12}$), 7.46 (d, $^3J_{^{195}Pt-^1H}$ ≈ 22, $J$ = 8.5, 1H, $H^{15}$), 7.08 (dd, $J$ = 8.5, 3.0, 1H, $H^{14}$), 3.86 (s, 3H, $H^{17}$). $^{13}$C NMR (151 MHz, DMSO-d$_6$) $\delta_C$ = 148.8 ($C^1$), 144.0 ($C^8$), 142.4 ($C^3$), 132.7 ($C^{15}$), 129.6 ($C^2$), 126.4 ($C^4$), 123.3 ($C^9$), 123.1 ($C^7$), 119.0 ($C^{14}$), 113.1 ($C^{12}$), 56.2 ($C^{17}$). HRMS (ES$^+$) *m/z* = 566.0297 [M – Cl + MeCN]$^+$, calcd for $[C_{19}H_{16}N_3OCl_2{}^{194}Pt]^+$ 566.0297.

**$PtL^{5Cl}Cl_3$**

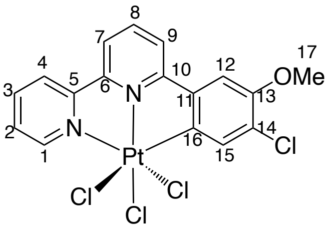


**$PtL^{5Cl}Cl_3$** was obtained as the product of oxidation of $PtL^5Cl$ (175 mg, 0.36 mmol) using $Cl_2$, as described for $PtL^1Cl$ in the main text. The product was extracted into $CH_2Cl_2$ and the solvent removed under reduced pressure. Recrystallization from $CH_2Cl_2$ / diethyl ether gave $PtL^{5Cl}Cl_3$ as a pale-yellow solid (60 mg, 28% yield). $^1$H NMR (599 MHz, DMSO-d$_6$) $\delta_H$ = 9.12 – 9.08 (m, 1H, $H^1$), 8.86 (d, $J$ = 8.0, 1H, $H^4$), 8.64 (dd, $J$ = 8.0, 1.0, 1H, $H^7$), 8.62 (dd, $J$ = 8.0, 1.0, 1H, $H^9$), 8.51 – 8.44 (m, 2H, $H^8$ and $H^3$), 8.08 (ddd, $J$ = 7.5, 5.5, 1.0, 1H, $H^2$), 7.85 (s, 1H, $H^{12}$), 7.49 (s, $^3J_{^{195}Pt-^1H} \approx$ 19, 1H, $H^{15}$), 3.99 (s, 3H, $H^{17}$). $^{13}$C NMR (151 MHz, DMSO-d$_6$) $\delta_C$ = 149.0 ($C^1$), 144.2 ($C^3$ or $C^8$), 142.6 ($C^3$ or $C^8$), 132.0 ($C^{13}$), 129.5 ($C^2$), 126.6 ($C^4$), 123.8 ($C^9$), 123.5 ($C^7$), 112.1 ($C^{12}$), 57.3 ($C^{17}$). HRMS (ES$^+$) $m/z$ = 599.9902 [M – Cl + MeCN]$^+$, calcd for $[C_{19}H_{15}N_3OCl_3{}^{194}Pt]^+$ 599.9907.

**$PtL^6Cl_3$**

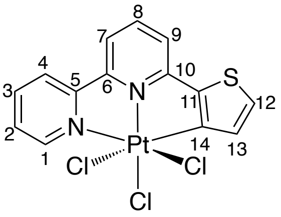


**$PtL^6Cl_3$** was obtained upon oxidation of $PtL^6Cl$ (220 mg, 0.47 mmol) with $PhICl_2$ (129 mg, 0.47 mmol), as described for $PtL^2Cl_3$ above. The product was extracted into $CH_2Cl_2$ to yield a yellow solid (238 mg, 94% yield). $^1$H NMR (599 MHz, DMSO-d$_6$) $\delta_H$ = 9.08 – 9.04 (m, 1H, $H^1$), 8.82 (d, $J$ = 8.0, 1H, $H^4$), 8.46 (td, $J$ = 7.9, 1.6, 1H, $H^3$), 8.44 – 8.40 (m, 1H, $H^7$), 8.29 (t, $J$ = 8.0, 1H, $H^8$), 8.11 (d, $J$ = 5.0, 1H, $H^{12}$), 8.09 (dd, $J$ = 8.0, 1.0, 1H, $H^9$), 8.06 (ddd, $J$ = 7.7, 5.3, 1.2, 1H, $H^2$), 7.25 (d, $^3J_{^{195}Pt-^1H} \approx$ 13, $J$ = 4.9, 1H, $H^{13}$). HRMS (ES$^+$) $m/z$ = 541.9771 [M – Cl + MeCN]$^+$, calcd for $[C_{16}H_{12}N_3SCl_2{}^{194}Pt]^+$ 541.9756.

## Synthesis and characterisation of [Pt(*NNC*)(*NC*)Cl]$PF_6$ complexes

### [$PtL^4$(4-MeOppy)Cl]$PF_6$

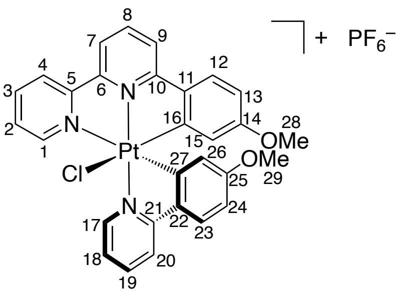

This compound was prepared as described for [$PtL^1$(ppy)Cl]$PF_6$ in the main text, from $PtL^4Cl_3$ (24 mg, 0.04 mmol), 2-(4-methoxyphenyl)pyridine (8 mg, 0.04 mmol) and AgOTf (22 mg, 0.09 mmol). The complex was purified by three fractional precipitations from $CH_2Cl_2$ / diethyl ether, yielding a yellow solid (5 mg, 14% yield). Crystals suitable for X-ray diffraction were obtained by the slow diffusion of $Et_2O$ into a $CH_2Cl_2$ solution of the complex. $^1$H NMR (599 MHz, acetone-d$_6$) $\delta_H$ = 9.90 (dt, $^3J_{^{195}Pt-^1H} \approx 26$, $J$ = 6.0, 1.0, 1H, $H^{17}$), 8.85 (d, $J$ = 8.0, 1H, $H^4$), 8.66 – 8.58 (m, 2H, $H^7$ and $H^8$), 8.51 – 8.41 (m, 4H, $H^3$,$H^{9,}$ $H^{19}$ and $H^{20}$), 8.39 (q, $^3J_{^{195}Pt-^1H} \approx 15$, $J$ = 5.0, 1H, $H^1$), 8.03 – 7.97 (m, 2H, $H^{12}$ and $H^{23}$), 7.86 (ddd, $J$ = 7.5, 6.0, 1.5, 1H, $H^{18}$), 7.79 (ddd, $J$ = 7.5, 5.5, 1.0, 1H, $H^2$), 6.86 (td, $J$ = 8.5, 2.5, 2H, $H^{13}$ and $H^{24}$), 5.68 (d, $^3J_{^{195}Pt-^1H} \approx 39$, $J$ = 2.5, 1H, $H^{26}$), 5.62 (d, $^3J_{^{195}Pt-^1H} \approx 37$, $J$ = 2.5, 1H, $H^{15}$), 3.67 (s, 3H, $H^{28}$), 3.59 (s, 3H, $H^{29}$). $^{13}$C NMR (151 MHz, acetone-d$_6$) $\delta_C$ = 149.4 ($C^1$), 148.9 ($C^{17}$), 143.4 ($C^8$), 142.6 ($C^{19}$), 142.0 ($C^3$), 129.3 ($C^{12}$), 129.0 ($C^2$), 128.0 ($C^{23}$), 126.2 ($C^4$), 124.5 ($C^{18}$), 122.3 ($C^9$), 121.5 ($C^{20}$), 121.3 ($C^7$), 115.1 ($C^{15}$), 112.9 ($C^{26}$), 111.3 ($C^{24}$), 110.7 ($C^{13}$), 55.0 ($C^{28}$), 54.9 ($C^{29}$). HRMS (ES$^+$) *m/z* = 674.1105 [M]$^+$, calcd for $[C_{29}H_{23}N_3O_2Cl^{194}Pt]^+$ 674.1106.

### [$PtL^5$(3-MeOppy)Cl]$PF_6$

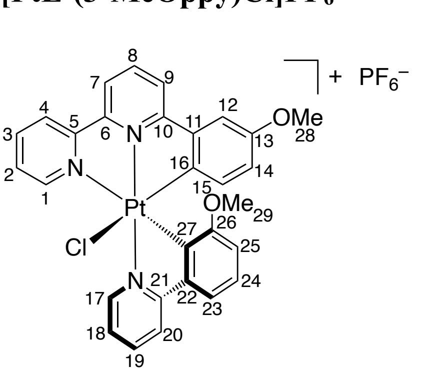

This compound was similarly prepared as described for [$PtL^1$(ppy)Cl]$PF_6$ in the main text, from $PtL^5Cl_3$ (40 mg, 0.07 mmol), 2-(3-methoxyphenyl)pyridine (13 mg, 0.07 mmol) and AgOTf (37 mg, 0.14 mmol). The complex was purified by gradient column chromatography on C8 reverse-

phase silica with water : MeCN, yielding a pale yellow solid (3 mg, 5% yield). Crystals suitable for X-ray diffraction were obtained by layering a $CH_2Cl_2$ solution of the complex with $Et_2O$. $^{1}$H NMR (599 MHz, acetone-d$_6$) $\delta_H$ = 10.11 – 10.00 (m, $^{3}J_{^{195}Pt-^{1}H} \approx 26$, 1H, $H^{17}$), 8.87 (d, $J$ = 8.0, 1H, $H^{4}$), 8.68 (dd, $J$ = 8.0, 1.0, 1H, $H^{7}$), 8.63 – 8.51 (m, 4H, $H^{8}$, $H^{9}$, $H^{19}$ and $H^{20}$), 8.44 (td, $J$ = 8.0, 1.5, 1H, $H^{3}$), 8.39 (d, $^{3}J_{^{195}Pt-^{1}H} \approx 14$, $J$ = 5.0, 1H, $H^{1}$), 7.96 – 7.88 (m, 1H, $H^{18}$), 7.79 (ddd, $J$ = 7.5, 5.5, 1.5, 1H, $H^{2}$), 7.69 (dd, $J$ = 8.0, 1.0, 1H, $H^{23}$), 7.63 – 7.57 (m, 1H, $H^{12}$), 7.23 (t, $J$ = 8.0, 1H, $H^{24}$), 6.72 (dd, $J$ = 8.5, 3.0, 1H, $H^{14}$), 6.60 (d, $J$ = 8.5, 1H, $H^{25}$), 6.05 – 5.95 (m, $^{3}J_{^{195}Pt-^{1}H} \approx 32$, 1H, $H^{15}$), 3.81 (s, 3H, $H^{28}$), 3.16 (s, 3H, $H^{29}$). HRMS (ES$^+$) $m/z$ = 674.1118 $[M]^+$, calcd for $[C_{29}H_{23}N_3O_2Cl^{194}Pt]^+$ 674.1106.

**[PtL$^{5Cl}$(ppy)Cl]PF$_6$**

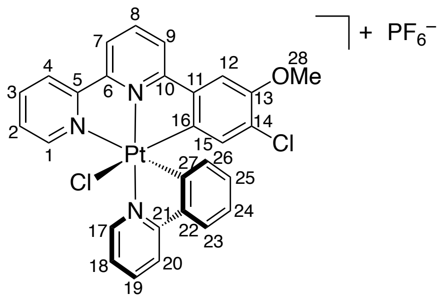


This compound was similarly prepared as described for [PtL$^1$(ppy)Cl]PF$_6$ in the main text, from PtL$^{5Cl}$Cl$_3$ (36 mg, 0.06 mmol), 2-phenylpyridine (9 mg, 0.06 mmol) and AgOTf (31 mg, 0.12 mmol). The complex was purified column chromatography on silica with MeCN : water : saturated aqueous $KNO_3$ (100 : 0 : 0 to 80 : 19.5 : 0.5), yielding an orange solid (2 mg, 4% yield). Crystals suitable for X-ray diffraction were obtained by the slow diffusion of $Et_2O$ into an acetone solution of the complex. $^{1}$H NMR (599 MHz, acetone-d$_6$) $\delta_H$ = 10.00 (d, $^{3}J_{^{195}Pt-^{1}H} \approx 26$, $J$ = 6.0, 1H, $H^{17}$), 9.05 (d, $J$ = 8.0, 1H, $H^{4}$), 8.94 (d, $J$ = 8.0, 1H, $H^{7}$), 8.86 (d, $J$ = 8.0, 1H, $H^{9}$), 8.73 (t, $J$ = 8.0, 1H, $H^{8}$), 8.65 – 8.59 (m, 2H, $H^{19}$ and $H^{20}$), 8.45 (d, $J$ = 8.0, 1H, $H^{3}$), 8.37 (d, $^{3}J_{^{195}Pt-^{1}H} \approx 15$, $J$ = 5.0, 1H, $H^{1}$), 8.07 (d, $J$ = 7.5 Hz, 1H, $H^{23}$), 8.01 (m, 1H, $H^{18}$), 7.95 (s, 1H, $H^{12}$), 7.83 – 7.78 (m, 1H, $H^{2}$), 7.27 (t, $J$ = 7.5 Hz, 1H, $H^{24}$), 7.03 (m, 1H, $H^{25}$), 6.34 (d, $^{3}J_{^{195}Pt-^{1}H} \approx 34$, $J$ = 8.0, 1H, $H^{26}$), 6.18 (s, $^{3}J_{^{195}Pt-^{1}H} \approx 35$, 1H, $H^{15}$), 4.00 (s, 3H, $H^{28}$). HRMS (ES$^+$) $m/z$ = 678.0631 $[M]^+$, calcd for $[C_{28}H_{20}N_3OCl_2{}^{194}Pt]^+$ 678.0610.

## Section 2 X-ray crystallography details and additional figures of molecular and crystal structures

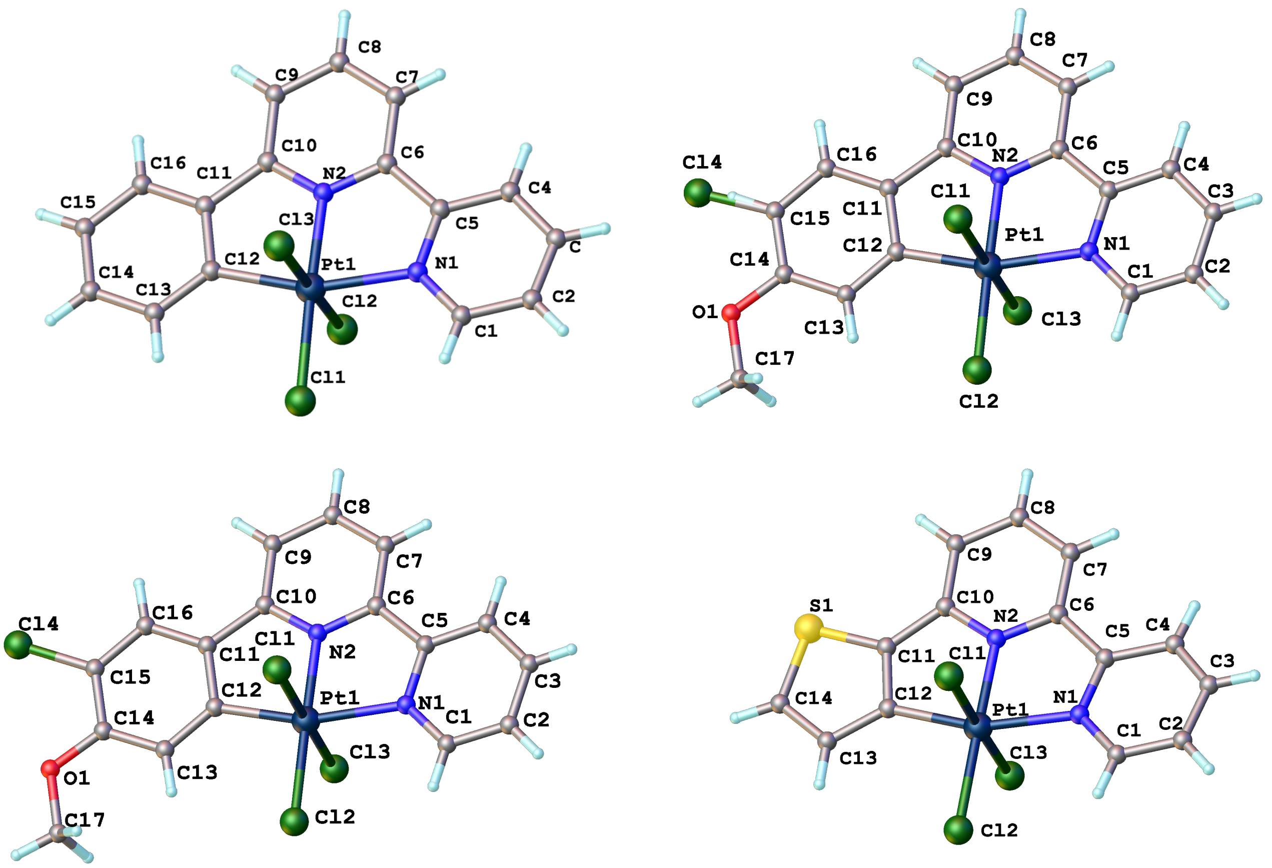


***Figure S2.1*** *Molecular structures of* $PtL^{1}Cl_{3}$*,* $PtL^{4}Cl_{3}$ *(with some co-crystallised* $PtL^{4Cl}Cl_{3}$*),* $PtL^{4Cl}Cl_{3}$*, and* $PtL^{6}Cl_{3}$ *in the crystals at 120 K, determined by X-ray diffraction.*

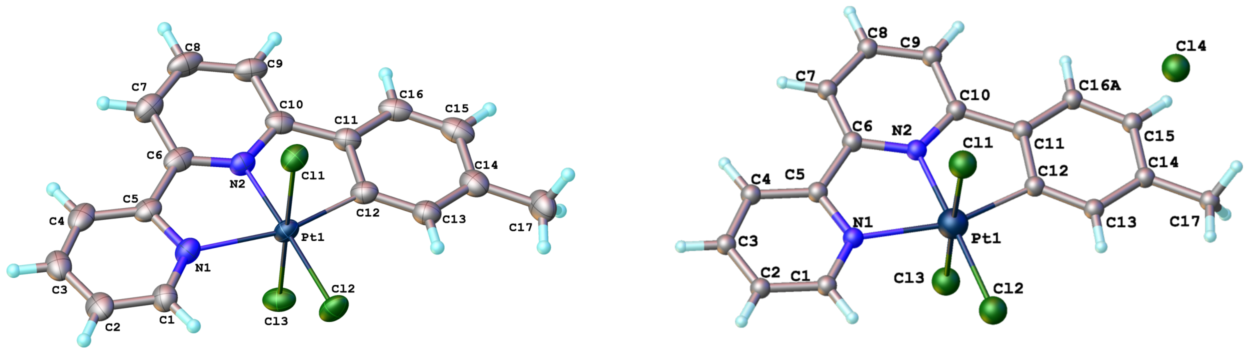


***Figure S2.2*** *Molecular structure of* $PtL^{3}Cl_{3}$ *(left) and of the chlorinated derivative* $PtL^{3Cl}Cl_{3}$ *also present in the crystal in a proportion of approximately 20%, determined by X-ray diffraction.*

**Table S2.1** Selected bond lengths (Å) and angles (°) for $PtL^1Cl_3$, $PtL^4Cl_3$, $PtL^{4Cl}Cl_3$ and $PtL^6Cl_3$

| **Bond length / Å or bond angle / °** | **$PtL^1Cl_3$,** | **$PtL^4Cl_3$** | **$PtL^{4Cl}Cl_3$,** | **$PtL^6Cl_3$** |
|---|---|---|---|---|
| Pt–C | 2.0543(19) / 2.1323(17) | 2.002(4) | 2.007(3) | 2.013(4) |
| Pt–N1 (lateral) | 2.1323(17) / 2.0543(19) | 2.143(3) | 2.143(3) | 2.136(3) |
| Pt–N2 (central) | 1.9747(16) | 1.982(3) | 1.975(3) | 1.977(4) |
| Pt–$Cl^{trans}$ *(a)* | 2.3112(5) | 2.3149(10) | 2.3141(9) | 2.3108(14) |
| Pt–$Cl^{cis}$ | 2.3279(5) | 2.3236(9) | 2.3214(9) | 2.3200(13) |
| Pt–$Cl^{cis}$ | 2.3135(5) | 2.3178(10) | 2.3262(9) | 2.3218(11) |
| N2–Pt–C | 81.42(7) / 79.92(7) | 82.40(15) | 82.36(13) | 81.57(17) |
| N2–Pt–N1 | 79.92(7) / 81.42(7) | 79.82(13) | 79.68(11) | 79.31(14) |
| N1–Pt–$Cl^{trans}$ *(a)* | 98.39(5) / 100.30(6) | 98.66(10) | 98.99(8) | 99.95(12) |
| C–Pt–$Cl^{trans}$ *(a)* | 100.30(6) / 98.39(5) | 99.11(12) | 98.96(10) | 99.18(15) |

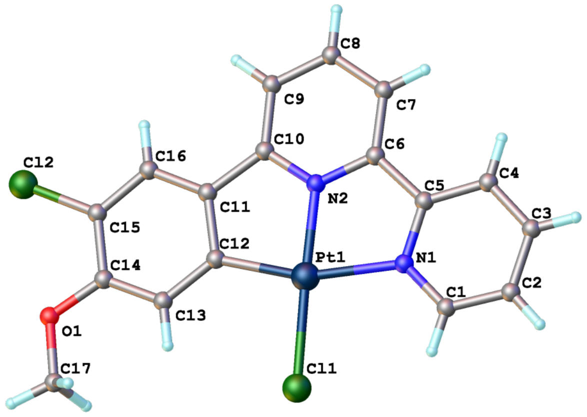


***Figure S2.3*** *Molecular structure of $PtL^{4Cl}Cl$ in the crystals at 120 K, determined by X-ray diffraction. Key bond lengths (Å) and angles (°): Pt–C 1.998(6); Pt–N1 (lateral) = 2.118(5); Pt–N2 (central) = 1.948(5); Pt–Cl 2.317(2); N2–Pt–C 82.0(2); N2–Pt–N1 79.9(2); N1–Pt–Cl 99.2(2); C–Pt–Cl 98.9(2).*

**Table S2.2** Crystal data and structure refinement for Pt(II) complexes $PtL^5Cl$ and $PtL^{4Cl}Cl$

| | **$PtL^5Cl$** | **$PtL^{4Cl}Cl$** |
|---|---|---|
| Identification code | 21srv436 | 22srv111 |
| Empirical formula | $C_{18}H_{16}ClN_2O_{1.5}PtS_{0.5}$ | $C_{17}H_{12}Cl_2N_2OPt$ |
| Formula weight | 530.90 | 526.28 |
| Temperature/K | 120.0 | 120.00 |
| Crystal system | monoclinic | triclinic |
| Space group | C2/c | P-1 |
| a/Å | 26.5322(6) | 8.1312(5) |
| b/Å | 8.0304(2) | 9.7780(6) |
| c/Å | 16.1422(4) | 9.9408(6) |
| α/° | 90 | 101.229(2) |
| β/° | 108.4900(10) | 102.073(2) |
| γ/° | 90 | 90.519(2) |
| Volume/$Å^3$ | 3261.78(14) | 757.06(8) |
| Z | 8 | 2 |
| $\rho_{calc}g/cm^3$ | 2.162 | 2.309 |
| $\mu/mm^{-1}$ | 8.842 | 9.624 |
| F(000) | 2024.0 | 496.0 |
| Crystal size/$mm^3$ | 0.14 × 0.08 × 0.06 | 0.12 × 0.03 × 0.01 |
| Radiation | Mo Kα (λ = 0.71073) | Mo Kα (λ = 0.71073) |
| 2Θ range for data collection/° | 5.322 to 59.998 | 4.252 to 59.992 |
| Index ranges | -37 ≤ h ≤ 37, -11 ≤ k ≤ 11, -22 ≤ l ≤ 22 | -11 ≤ h ≤ 11, -13 ≤ k ≤ 13, -13 ≤ l ≤ 13 |
| Reflections collected | 38081 | 16311 |
| Independent reflections | 4738 [$R_{int}$ = 0.0314, $R_{sigma}$ = 0.0184] | 4404 [$R_{int}$ = 0.0433, $R_{sigma}$ = 0.0449] |
| Data/restraints/parameters | 4738/2/238 | 4404/0/209 |
| Goodness-of-fit on $F^2$ | 1.040 | 1.116 |
| Final R indexes [I>=2σ (I)] | $R_1$ = 0.0143, $wR_2$ = 0.0306 | $R_1$ = 0.0392, $wR_2$ = 0.0775 |
| Final R indexes [all data] | $R_1$ = 0.0165, $wR_2$ = 0.0313 | $R_1$ = 0.0454, $wR_2$ = 0.0795 |
| Largest diff. peak/hole / e $Å^{-3}$ | 0.69/-0.52 | 7.61/-2.34 |

**Table S2.3** Crystal data and structure refinement for the $Pt^{IV}L^{n}Cl_3$ complexes of the unsubstituted phenyl and thienyl ligands: $PtL^1Cl_3$ and $PtL^6Cl_3$

| | **$PtL^1Cl_3$** | **$PtL^6Cl_3$** |
|---|---|---|
| Identification code | 21srv196 | 21srv447 |
| Empirical formula | $C_{16}H_{11}Cl_3N_2Pt$ | $C_{15}H_{12}Cl_3N_2O_2PtS_2$ |
| Formula weight | 532.71 | 617.83 |
| Temperature/K | 120.0 | 120.0 |
| Crystal system | monoclinic | triclinic |
| Space group | $P2_1/n$ | P-1 |
| a/Å | 8.2970(2) | 8.3113(2) |
| b/Å | 14.3443(4) | 10.6355(3) |
| c/Å | 13.5758(3) | 11.0657(3) |
| α/° | 90 | 82.1790(10) |
| β/° | 105.3560(10) | 83.5440(10) |
| γ/° | 90 | 88.0070(10) |
| Volume/$Å^3$ | 1558.04(7) | 962.72(4) |
| Z | 4 | 2 |
| $\rho_{calc}g/cm^3$ | 2.271 | 2.131 |
| $\mu/mm^{-1}$ | 9.516 | 7.932 |
| F(000) | 1000.0 | 586.0 |
| Crystal size/$mm^3$ | 0.18 × 0.09 × 0.04 | 0.12 × 0.04 × 0.005 |
| Radiation | MoKα (λ = 0.71073) | Mo Kα (λ = 0.71073) |
| 2Θ range for data collection/° | 4.212 to 59.994 | 3.738 to 59.998 |
| Index ranges | -11 ≤ h ≤ 11, -20 ≤ k ≤ 20, -19 ≤ l ≤ 19 | -11 ≤ h ≤ 11, -14 ≤ k ≤ 14, -15 ≤ l ≤ 15 |
| Reflections collected | 26205 | 23142 |
| Independent reflections | 4517 [$R_{int}$ = 0.0264, $R_{sigma}$ = 0.0186] | 5606 [$R_{int}$ = 0.0353, $R_{sigma}$ = 0.0329] |
| Data/restraints/parameters | 4517/0/199 | 5606/0/246 |
| Goodness-of-fit on $F^2$ | 1.082 | 1.086 |
| Final R indexes [I>=2σ (I)] | $R_1$ = 0.0140, $wR_2$ = 0.0292 | $R_1$ = 0.0337, $wR_2$ = 0.0699 |
| Final R indexes [all data] | $R_1$ = 0.0166, $wR_2$ = 0.0300 | $R_1$ = 0.0417, $wR_2$ = 0.0738 |
| Largest diff. peak/hole / e $Å^{-3}$ | 0.43/-0.60 | 2.70/-2.42 |

**Table S2.4** Crystal data and structure refinement for $PtL^5Cl_3$, $PtL^{5Cl}Cl_3$, $PtL^4Cl_3$ and $PtL^{4Cl}Cl_3$

| | **$PtL^5Cl_3$** | **$PtL^{5Cl}Cl_3$** | **$PtL^4Cl_3$** | **$PtL^{4Cl}Cl_3$** |
|---|---|---|---|---|
| Identification code | 21srv459 | 21srv410 | 22srv085 | 22srv129 |
| Empirical formula | $C_{17}H_{13}Cl_3N_2OPt$ | $C_{18}H_{15}Cl_4N_2O_{1.5}PtS_{0.5}$ | $C_{19}H_{18.5}Cl_{3.5}N_2O_2PtS$ | $C_{19}H_{18}Cl_4N_2O_2PtS$ |
| Formula weight | 562.73 | 636.24 | 658.08 | 675.30 |
| Temperature/K | 120.0 | 120.0 | 120.00 | 120.00 |
| Crystal system | monoclinic | monoclinic | monoclinic | monoclinic |
| Space group | $P2_1/n$ | $P2_1/n$ | $P2_1/n$ | $P2_1/n$ |
| a/Å | 8.3128(2) | 8.1467(2) | 13.0190(3) | 13.1070(4) |
| b/Å | 14.3004(4) | 18.5442(5) | 7.7027(2) | 7.8011(2) |
| c/Å | 14.4137(4) | 13.3755(4) | 21.5171(6) | 21.3095(6) |
| α/° | 90 | 90 | 90 | 90 |
| β/° | 94.5381(10) | 103.8530(10) | 93.4060(10) | 93.2290(10) |
| γ/° | 90 | 90 | 90 | 90 |
| Volume/Å$^3$ | 1708.08(8) | 1961.91(9) | 2153.95(10) | 2175.42(11) |
| Z | 4 | 4 | 4 | 4 |
| $\rho_{calc}$g/cm$^3$ | 2.188 | 2.154 | 2.029 | 2.062 |
| μ/mm$^{-1}$ | 8.690 | 7.764 | 7.064 | 7.057 |
| F(000) | 1064.0 | 1212.0 | 1264.0 | 1296.0 |
| Crystal size/mm$^3$ | 0.11 × 0.06 × 0.01 | 0.15 × 0.03 × 0.01 | 0.08 × 0.07 × 0.01 | 0.11 × 0.04 × 0.01 |
| Radiation | Mo Kα (λ = 0.71073) | Mo Kα (λ = 0.71073) | Mo Kα (λ = 0.71073) | Mo Kα (λ = 0.71073) |
| 2Θ range for data collection/° | 4.018 to 60 | 3.828 to 59.998 | 3.758 to 59.994 | 5.562 to 59.994 |
| Index ranges | -11 ≤ h ≤ 11, -20 ≤ k ≤ 20, -20 ≤ l ≤ 20 | -11 ≤ h ≤ 11, -26 ≤ k ≤ 26, -18 ≤ l ≤ 18 | -18 ≤ h ≤ 18, -10 ≤ k ≤ 10, -30 ≤ l ≤ 30 | -18 ≤ h ≤ 18, -10 ≤ k ≤ 10, -29 ≤ l ≤ 29 |
| Reflections collected | 40074 | 46848 | 51957 | 50536 |
| Independent reflections | 4977 [$R_{int}$ = 0.0339, $R_{sigma}$ = 0.0201] | 5712 [$R_{int}$ = 0.0511, $R_{sigma}$ = 0.0291] | 6276 [$R_{int}$ = 0.0420, $R_{sigma}$ = 0.0253] | 6335 [$R_{int}$ = 0.0381, $R_{sigma}$ = 0.0229] |
| Data/restraints/parameters | 4977/0/218 | 5712/12/254 | 6276/0/266 | 6335/0/265 |
| Goodness-of-fit on F$^2$ | 1.043 | 1.055 | 1.301 | 1.189 |
| Final R indexes [I>=2σ (I)] | $R_1$ = 0.0180, $wR_2$ = 0.0371 | $R_1$ = 0.0348, $wR_2$ = 0.0800 | $R_1$ = 0.0352, $wR_2$ = 0.0685 | $R_1$ = 0.0288, $wR_2$ = 0.0544 |
| Final R indexes [all data] | $R_1$ = 0.0231, $wR_2$ = 0.0388 | $R_1$ = 0.0409, $wR_2$ = 0.0831 | $R_1$ = 0.0395, $wR_2$ = 0.0698 | $R_1$ = 0.0329, $wR_2$ = 0.0555 |
| Largest diff. peak/hole / e Å$^{-3}$ | 1.34/-0.58 | 2.96/-1.63 | 2.31/-1.39 | 1.52/-1.36 |

**Table S2.5** Crystal data and structure refinement for $[PtL^1(ppy)Cl]OTf$ and $[PtL^6(thpy)Cl]PF_6$

| | $[PtL^1(ppy)Cl]^+$ $CF_3SO_3^-$ | $[PtL^6(thpy)Cl]^+$ $PF_6^-$ |
|---|---|---|
| Identification code | 21srv448 | 21srv110 |
| Empirical formula | $C_{29}H_{21}Cl_3F_3N_3O_3PtS$ | $C_{23.5}H_{16}Cl_2F_6N_3PPtS_2$ |
| Formula weight | 849.99 | 815.47 |
| Temperature/K | 120.0 | 120.00 |
| Crystal system | monoclinic | triclinic |
| Space group | $P2_1/c$ | P-1 |
| a/Å | 15.1961(6) | 9.8398(4) |
| b/Å | 13.4215(5) | 12.5659(6) |
| c/Å | 15.8875(6) | 12.9285(6) |
| α/° | 90 | 76.0700(10) |
| β/° | 111.904(3) | 71.2540(10) |
| γ/° | 90 | 84.3930(10) |
| Volume/Å$^3$ | 3006.4(2) | 1468.88(12) |
| Z | 4 | 2 |
| $\rho_{calc}$g/cm$^3$ | 1.878 | 1.844 |
| μ/mm$^{-1}$ | 5.059 | 5.212 |
| F(000) | 1648.0 | 782.0 |
| Crystal size/mm$^3$ | 0.11 × 0.08 × 0.01 | 0.08 × 0.06 × 0.01 |
| Radiation | Mo Kα (λ = 0.71073) | Mo Kα (λ = 0.71073) |
| 2Θ range for data collection/° | 4.104 to 59.994 | 4.212 to 54.998 |
| Index ranges | 21 ≤ h ≤ -21, 18 ≤ k ≤ -18, 22 ≤ l ≤ -22 | -12 ≤ h ≤ 12, -16 ≤ k ≤ 16, -16 ≤ l ≤ 16 |
| Reflections collected | 8629 | 24245 |
| Independent reflections | 8629 [$R_{int}$ = 0.0518, $R_{sigma}$ = 0.0500] | 6742 [$R_{int}$ = 0.0564, $R_{sigma}$ = 0.0637] |
| Data/restraints/parameters | 8629/6/388 | 6742/114/424 |
| Goodness-of-fit on F$^2$ | 1.051 | 1.042 |
| Final R indexes [I>=2σ (I)] | $R_1$ = 0.0390, $wR_2$ = 0.0888 | $R_1$ = 0.0471, $wR_2$ = 0.1159 |
| Final R indexes [all data] | $R_1$ = 0.0641, $wR_2$ = 0.0936 | $R_1$ = 0.0671, $wR_2$ = 0.1260 |
| Largest diff. peak/hole / e Å$^{-3}$ | 1.44/-2.81 | 1.72/-1.18 |

**Table S2.6** Crystal data and structure refinement for [PtL$^4$(4-MeOppy)Cl]$PF_6$, [PtL$^5$(3-MeOppy)Cl]OTf and [PtL$^{5Cl}$(ppy)Cl]$PF_6$

| | **[PtL$^4$(4-MeOppy)Cl]$PF_6$** | **[PtL$^5$(3-MeOppy)Cl]$^+$ $CF_3SO_3^-$** | **[PtL$^{5Cl}$(ppy)Cl]$PF_6$** |
|---|---|---|---|
| Identification code | 22srv026 | 21srv455 | 22srv141 |
| Empirical formula | $C_{29.5}H_{24}Cl_2F_6N_3O_2PPt$ | $C_{31}H_{25}Cl_3F_3N_3O_5PtS$ | $C_{29.5}H_{23}Cl_2F_6N_3O_{1.5}PPt$ |
| Formula weight | 863.48 | 910.04 | 854.47 |
| Temperature/K | 120.00 | 120.0 | 120.00 |
| Crystal system | triclinic | triclinic | triclinic |
| Space group | P-1 | P-1 | P-1 |
| a/Å | 13.8190(6) | 10.5820(4) | 11.6681(4) |
| b/Å | 15.4968(6) | 11.8805(4) | 13.1112(5) |
| c/Å | 15.5042(6) | 13.0407(4) | 13.7379(5) |
| α/° | 87.5698(16) | 88.2460(10) | 112.7128(11) |
| β/° | 83.6170(16) | 76.3250(10) | 104.4060(11) |
| γ/° | 87.6078(16) | 86.5130(10) | 102.3458(11) |
| Volume/Å$^3$ | 3294.4(2) | 1589.82(9) | 1761.17(11) |
| Z | 4 | 2 | 2 |
| $\rho_{calc}$g/cm$^3$ | 1.741 | 1.901 | 1.611 |
| μ/mm$^{-1}$ | 4.535 | 4.795 | 4.240 |
| F(000) | 1676.0 | 888.0 | 828.0 |
| Crystal size/mm$^3$ | 0.34 × 0.04 × 0.01 | 0.07 × 0.06 × 0.03 | 0.09 × 0.05 × 0.01 |
| Radiation | Mo Kα (λ = 0.71073) | Mo Kα (λ = 0.71073) | Mo Kα (λ = 0.71073) |
| 2Θ range for data collection/° | 3.802 to 59.998 | 4.482 to 57.998 | 4.252 to 59.994 |
| Index ranges | -19 ≤ h ≤ 19, -21 ≤ k ≤ 21, -21 ≤ l ≤ 21 | -14 ≤ h ≤ 14, -16 ≤ k ≤ 16, -17 ≤ l ≤ 17 | -16 ≤ h ≤ 16, -18 ≤ k ≤ 18, -19 ≤ l ≤ 19 |
| Reflections collected | 73924 | 38914 | 37817 |
| Independent reflections | 19211 [$R_{int}$ = 0.0721, $R_{sigma}$ = 0.0764] | 8443 [$R_{int}$ = 0.0676, $R_{sigma}$ = 0.0643] | 10264 [$R_{int}$ = 0.0575, $R_{sigma}$ = 0.0648] |
| Data/restraints/parameters | 19211/192/860 | 8443/0/430 | 10264/19/418 |
| Goodness-of-fit on F$^2$ | 1.021 | 1.064 | 1.039 |
| Final R indexes [I>=2σ (I)] | $R_1$ = 0.0521, w$R_2$ = 0.1107 | $R_1$ = 0.0458, w$R_2$ = 0.1011 | $R_1$ = 0.0417, w$R_2$ = 0.0937 |
| Final R indexes [all data] | $R_1$ = 0.0880, w$R_2$ = 0.1245 | $R_1$ = 0.0605, w$R_2$ = 0.1074 | $R_1$ = 0.0546, w$R_2$ = 0.0980 |
| Largest diff. peak/hole / e Å$^{-3}$ | 1.97/-1.75 | 1.66/-1.90 | 1.51/-1.17 |

## Section 3 Additional absorption and emission spectra

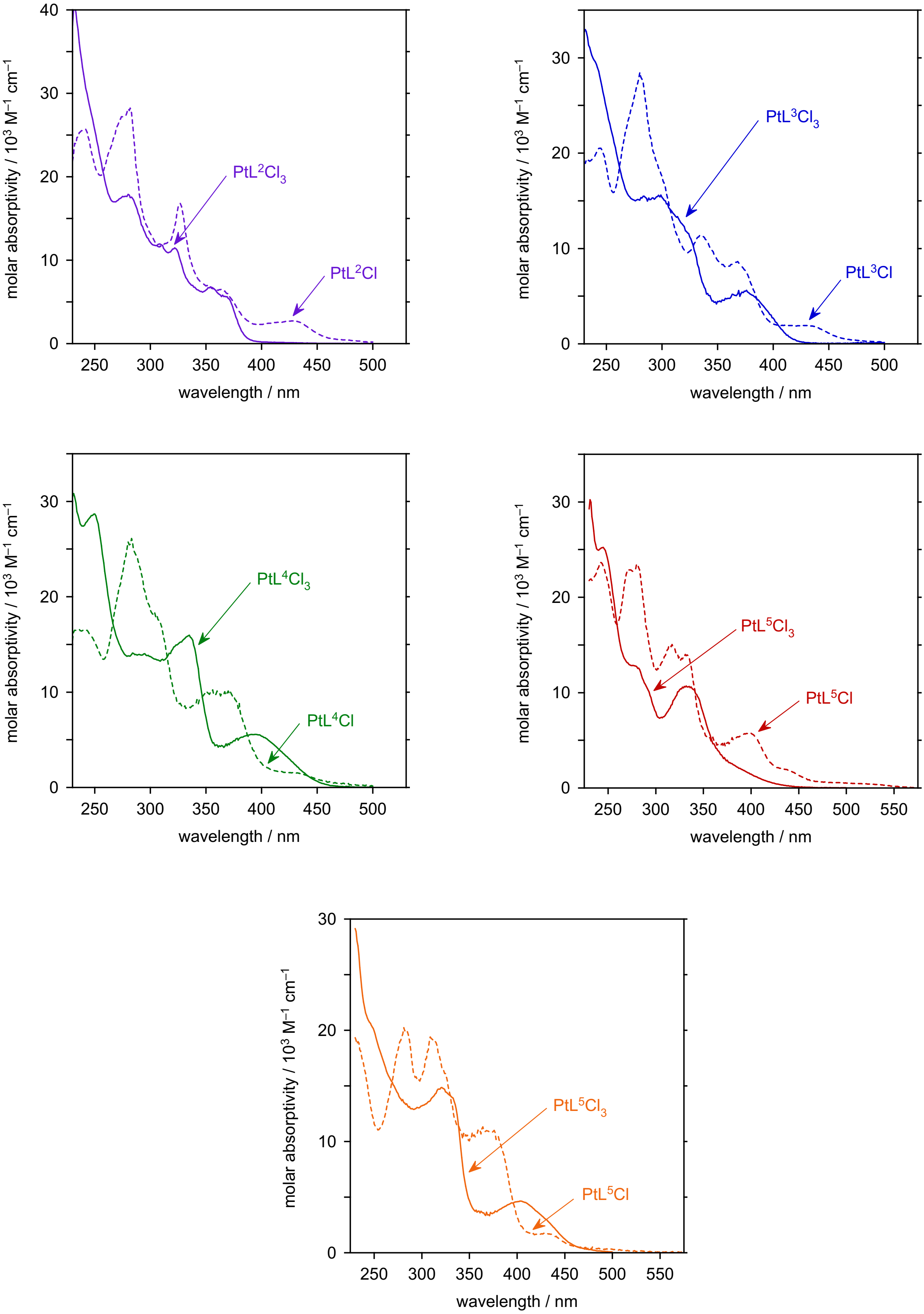


***Figure S3.1.*** *UV-visible absorption spectra of $PtL^nCl_3$ (solid lines) in $CH_2Cl_2$ at 295 K, with the spectra of the corresponding $PtL^nCl$ also shown for comparison in each case (dashed lines).*

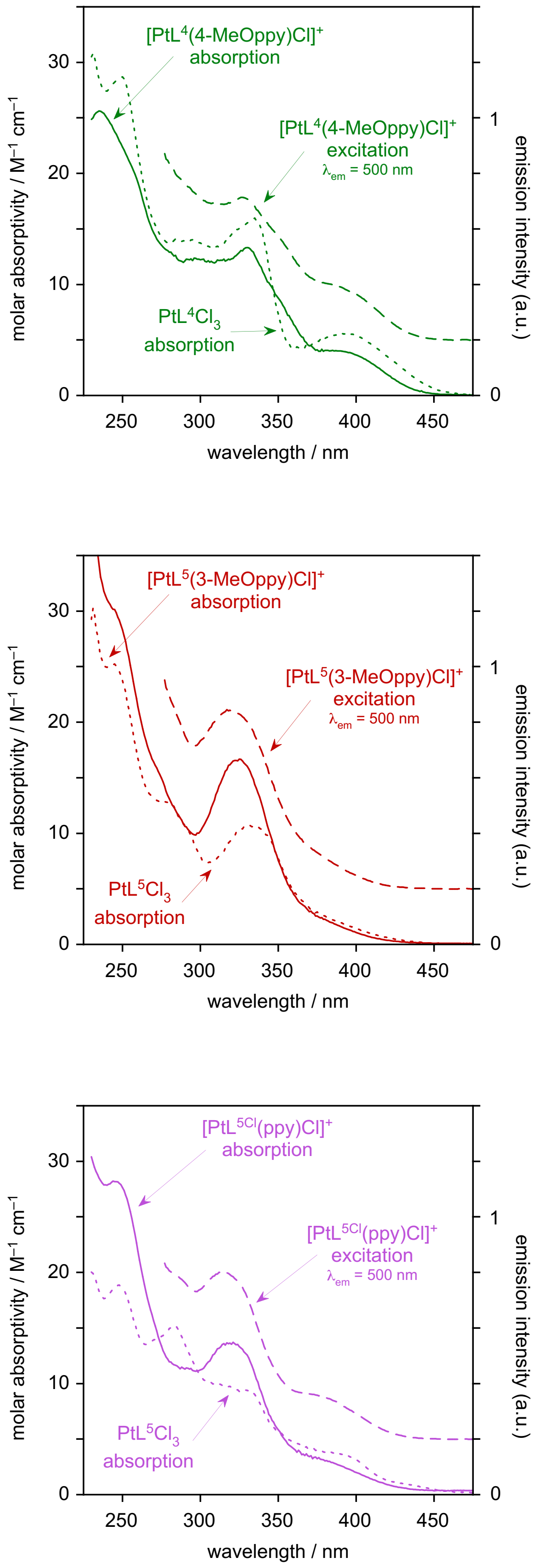


***Figure S3.2.*** *UV-visible absorption and photoluminescence excitation spectra of* $[PtL^4(4\text{-}MeOppy)Cl]^+$ *(top),* $[PtL^5(3\text{-}MeOppy)Cl]^+$ *(middle), and* $[PtL^{5}Cl(ppy)Cl]^+$ *(bottom) in MeCN at 295 K (solid and long dashed lines respectively), with the absorption spectrum of the respective precursor* $PtL^nCl_3$ *also shown for comparison in each case (short dashed line).*

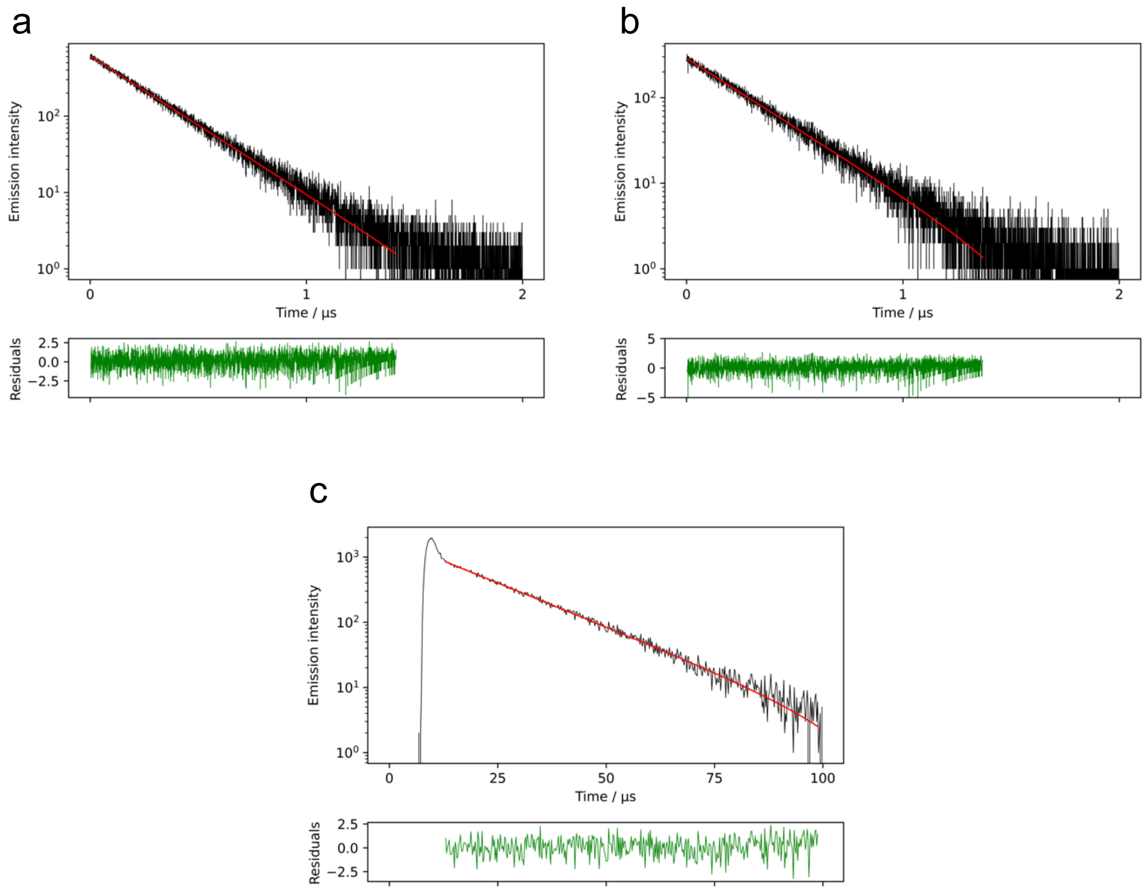


***Figure S3.3*** *Emission decay traces of PtL[1]Cl in (a) air-equilibrated $CH_2Cl_2$ at 295 K ($\chi^2$ = 1.089); (b) degassed $CH_2Cl_2$ at 295 K ($\chi^2$ = 1.073); and (c) diethyl ether / isopentane / ethanol (2:2:1 v/v) at 77K ($\chi^2$ = 0.984). Experimental data in black; monoexponential fitting in red; residuals in green.*

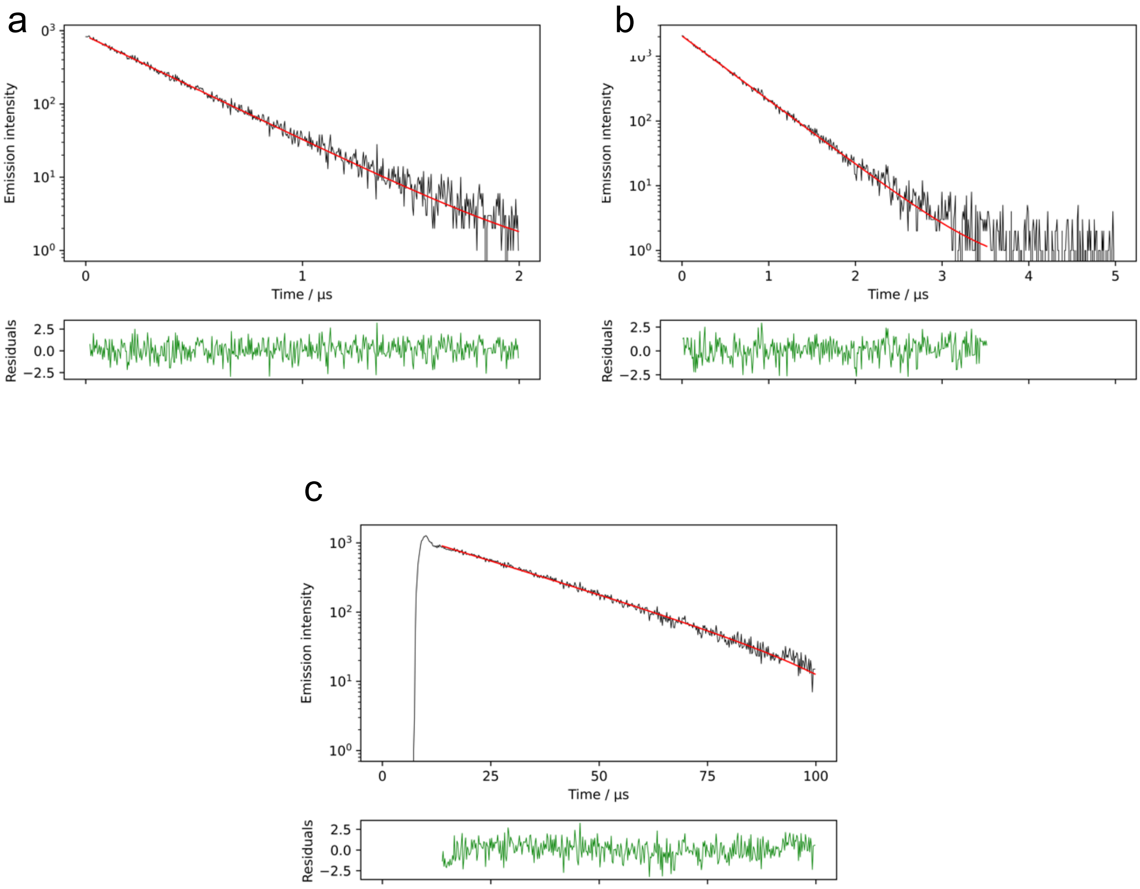


*__Figure S3.4__ Emission decay traces of PtL[2]Cl in (a) air-equilibrated $CH_2Cl_2$ at 295 K ($\chi^2$ = 0.992); (b) degassed $CH_2Cl_2$ at 295 K ($\chi^2$ = 1.069); and (c) diethyl ether / isopentane / ethanol (2:2:1 v/v) at 77K ($\chi^2$ = 1.180). Experimental data in black; monoexponential fitting in red; residuals in green.*

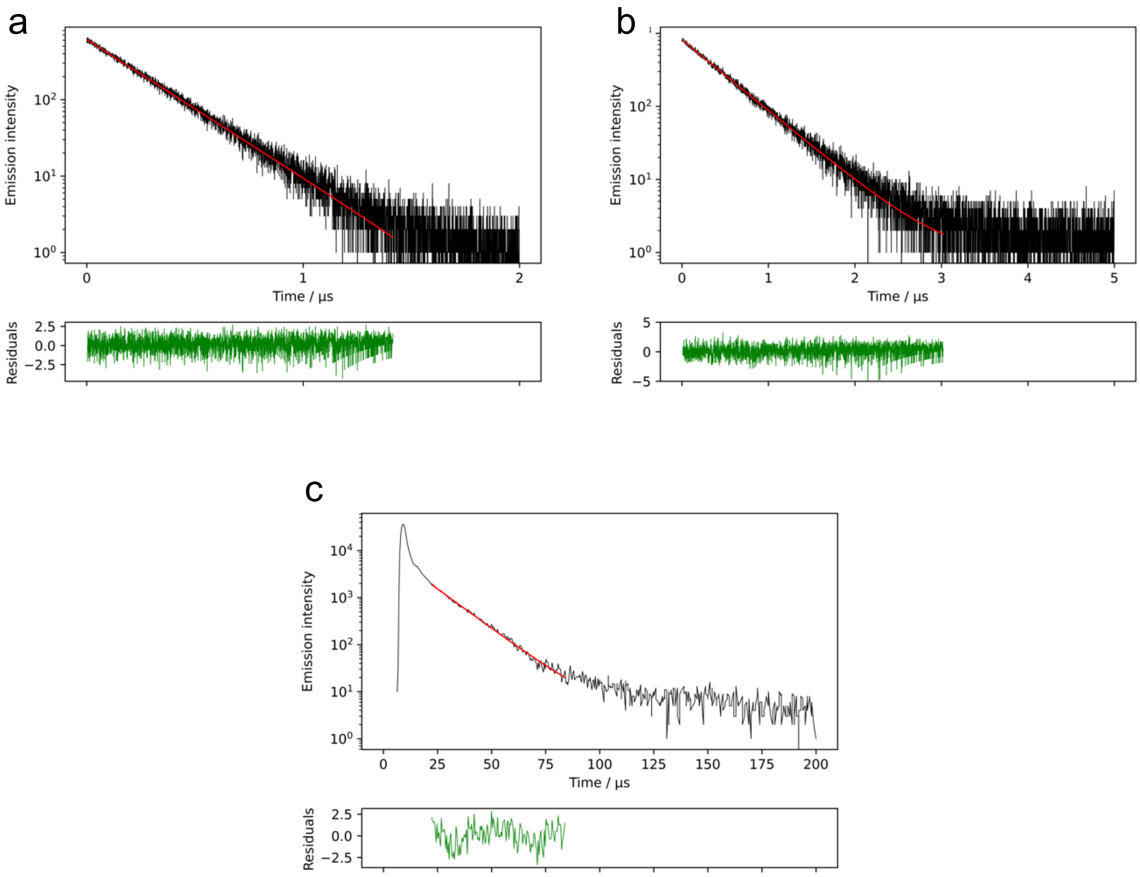


***Figure S3.5*** *Emission decay traces of PtL*$^3$*Cl in (a) air-equilibrated* $CH_2Cl_2$ *at 295 K (*$\chi^2$ *= 1.053); (b) degassed* $CH_2Cl_2$ *at 295 K (*$\chi^2$ *= 1.130); and (c) diethyl ether / isopentane / ethanol (2:2:1 v/v) at 77K (*$\chi^2$ *= 1.636). Experimental data in black; monoexponential fitting in red; residuals in green.*

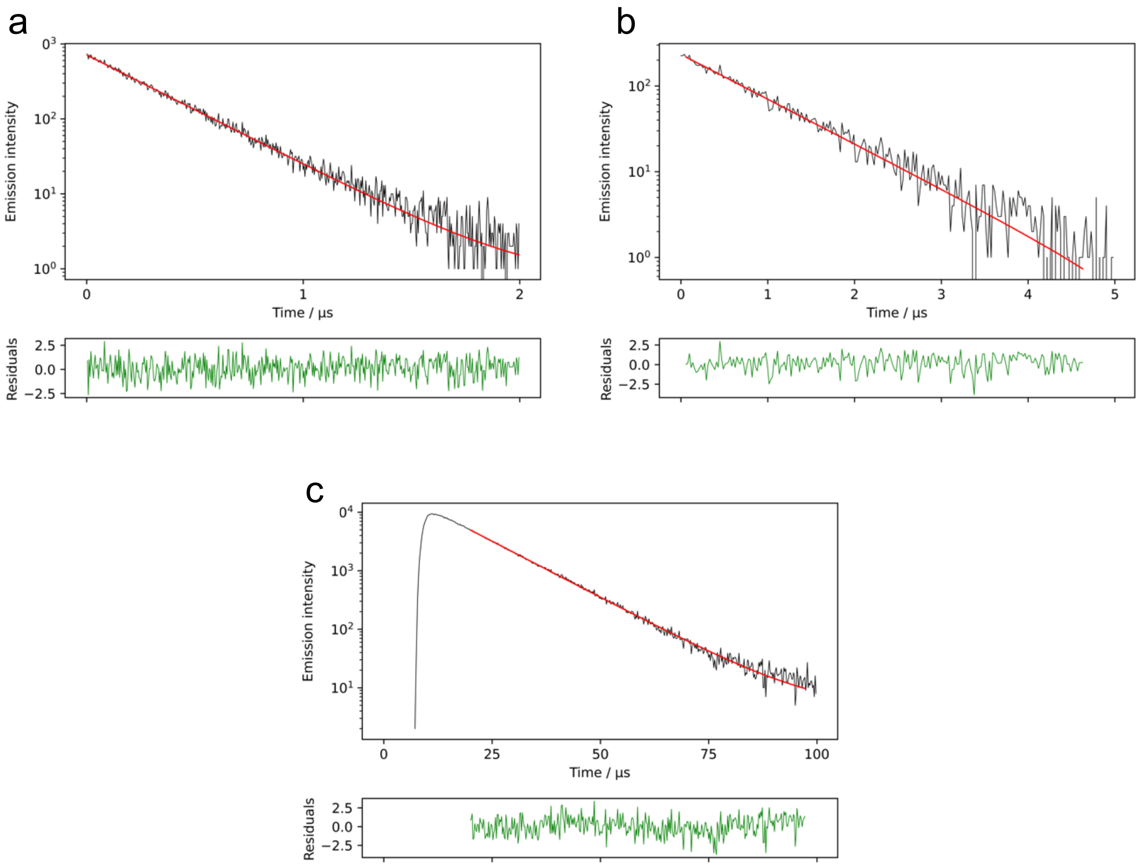


***Figure S3.6*** *Emission decay traces of $PtL^4Cl$ in (a) air-equilibrated $CH_2Cl_2$ at 295 K ($\chi^2$ = 1.028); (b) degassed $CH_2Cl_2$ at 295 K ($\chi^2$ = 1.074); and (c) diethyl ether / isopentane / ethanol (2:2:1 v/v) at 77K ($\chi^2$ = 1.448). Experimental data in black; monoexponential fitting in red; residuals in green.*

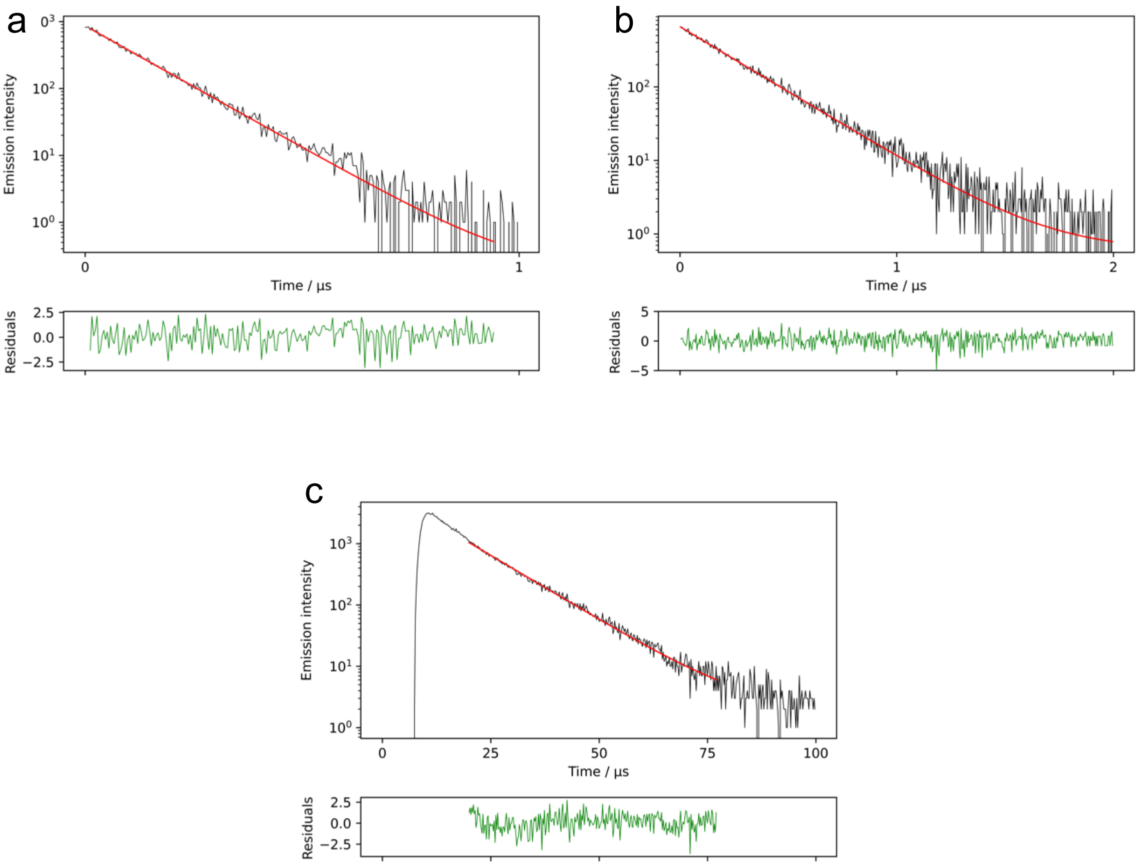


***Figure S3.7*** *Emission decay traces of PtL[5]Cl in (a) air-equilibrated $CH_2Cl_2$ at 295 K ($\chi^2$ = 1.077); (b) degassed $CH_2Cl_2$ at 295 K ($\chi^2$ = 1.091); and (c) diethyl ether / isopentane / ethanol (2:2:1 v/v) at 77K ($\chi^2$ = 1.102). Experimental data in black; monoexponential fitting in red; residuals in green.*

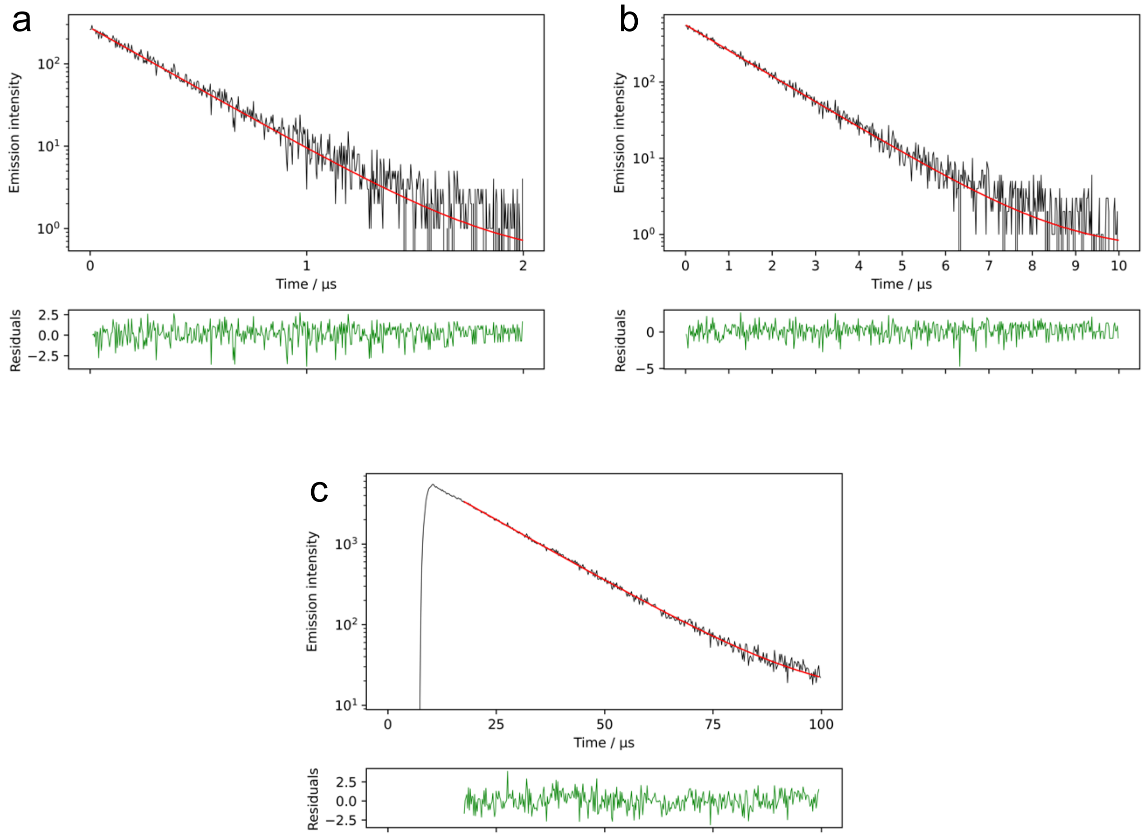


***Figure S3.8*** *Emission decay traces of PtL*$^{6}$*Cl in (a) air-equilibrated* $CH_2Cl_2$ *at 295 K (*$\chi^2$ *= 1.141); (b) degassed* $CH_2Cl_2$ *at 295 K (*$\chi^2$ *= 0.940); and (c) diethyl ether / isopentane / ethanol (2:2:1 v/v) at 77K (*$\chi^2$ *= 1.059). Experimental data in black; monoexponential fitting in red; residuals in green.*

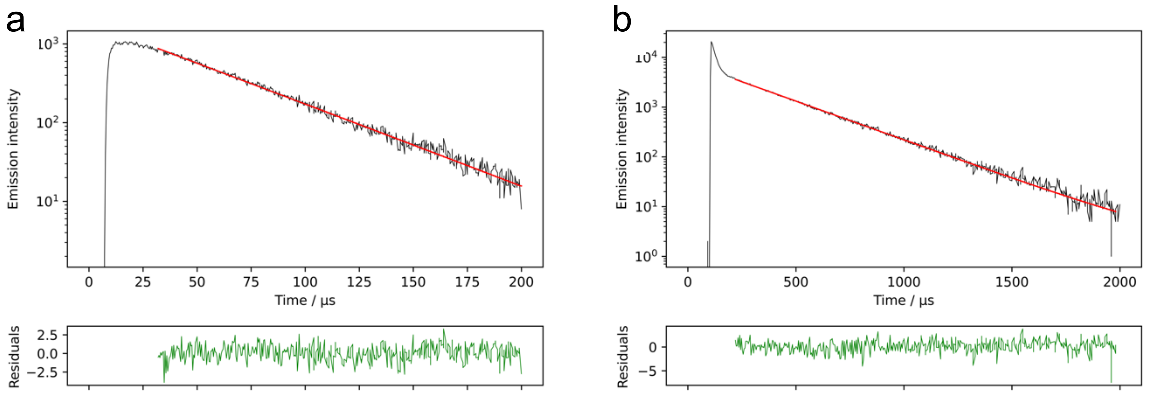


***Figure S3.9*** *Emission decay traces of [PtL*$^1$*(ppy)Cl]PF*$_6$ *in (a) degassed acetonitrile at 295 K (*$\chi^2$ *= 1.240); and (b) butyronitrile at 77K (*$\chi^2$ *= 1.396). Experimental data in black; monoexponential fitting in red; residuals in green.*

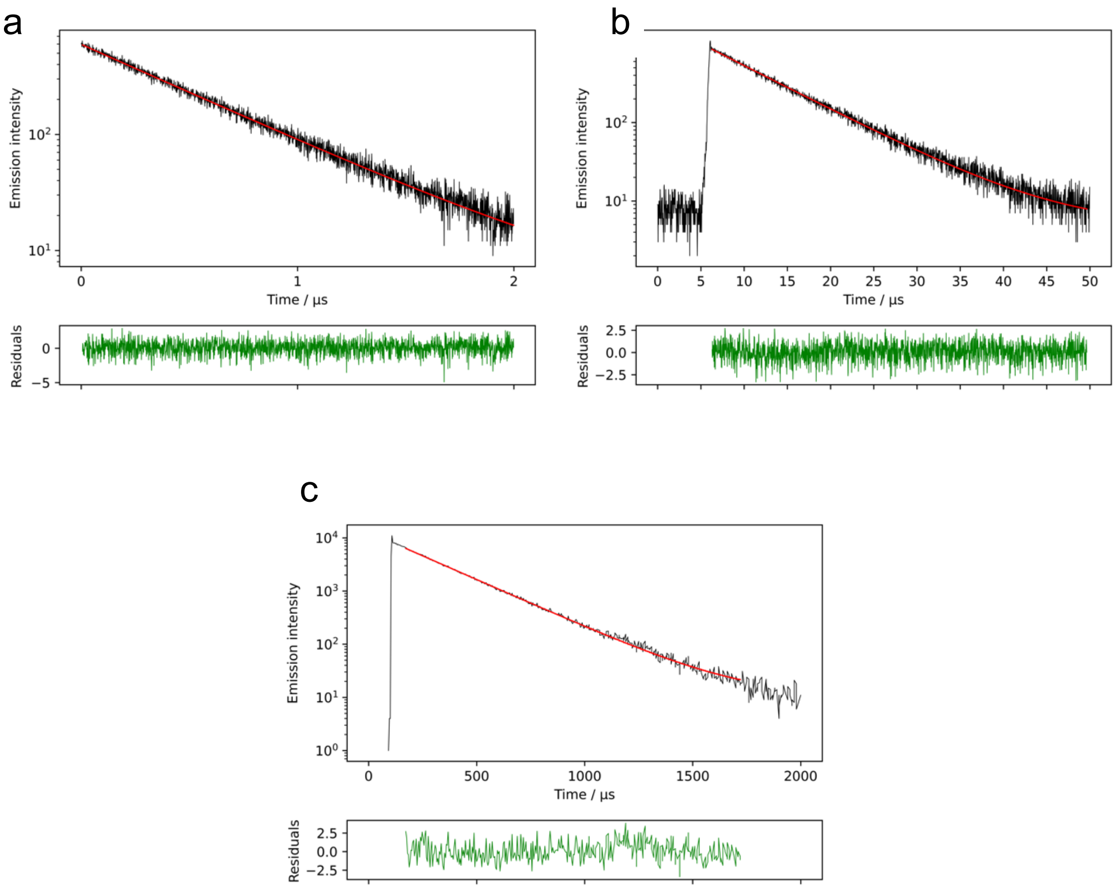


***Figure S3.10*** *Emission decay traces of [PtL*$^{4}$*(4-MeOppy)Cl]PF*$_6$ *in (a) air-equilibrated acetonitrile at 295 K (*$\chi^2$ *= 1.014); (b) degassed acetonitrile at 295 K (*$\chi^2$ *= 1.082); and (c) butyronitrile at 77K (*$\chi^2$ *= 1.604). Experimental data in black; monoexponential fitting in red; residuals in green.*

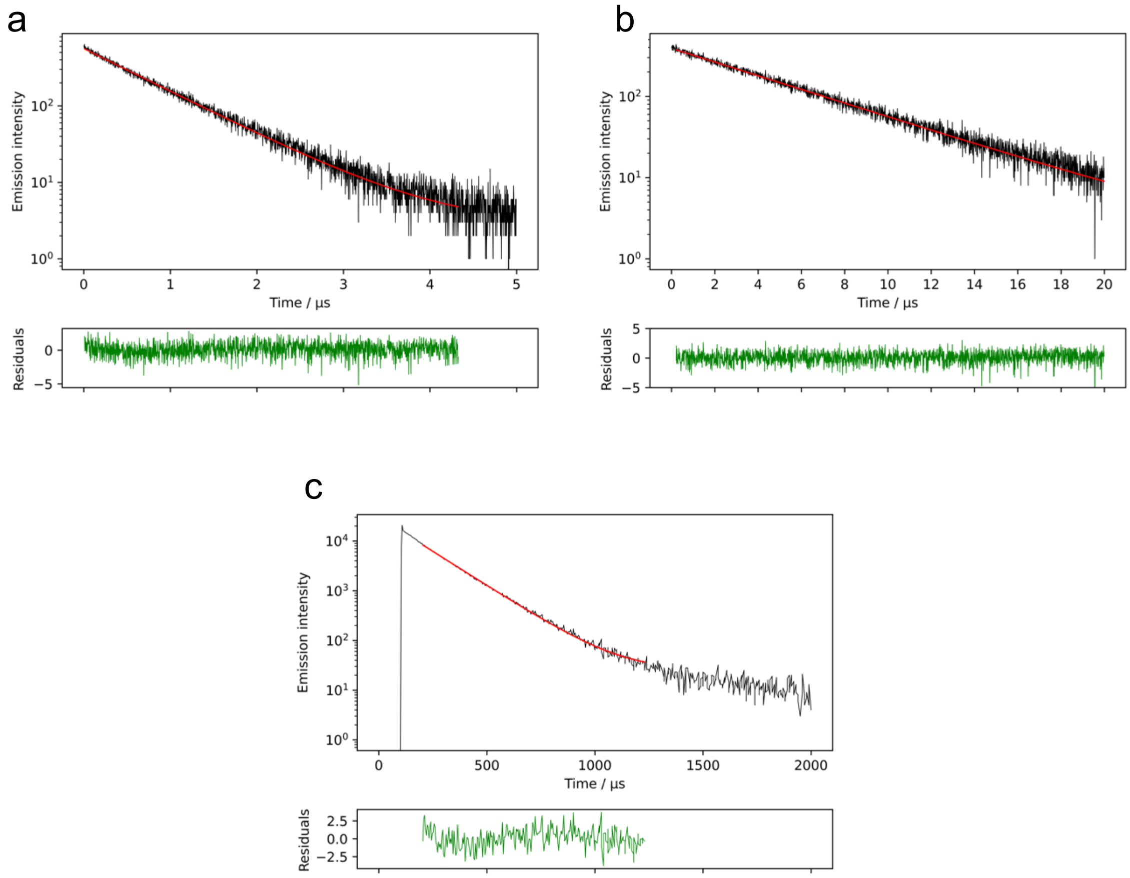


***Figure S3.11*** *Emission decay traces of [PtL$^{5Cl}$(ppy)Cl]PF$_6$ in (a) air-equilibrated acetonitrile at 295 K ($\chi^2$ = 1.046); (b) degassed acetonitrile at 295 K ($\chi^2$ = 1.012); and (c) butyronitrile at 77K ($\chi^2$ = 1.416). Experimental data in black; monoexponential fitting in red; residuals in green.*

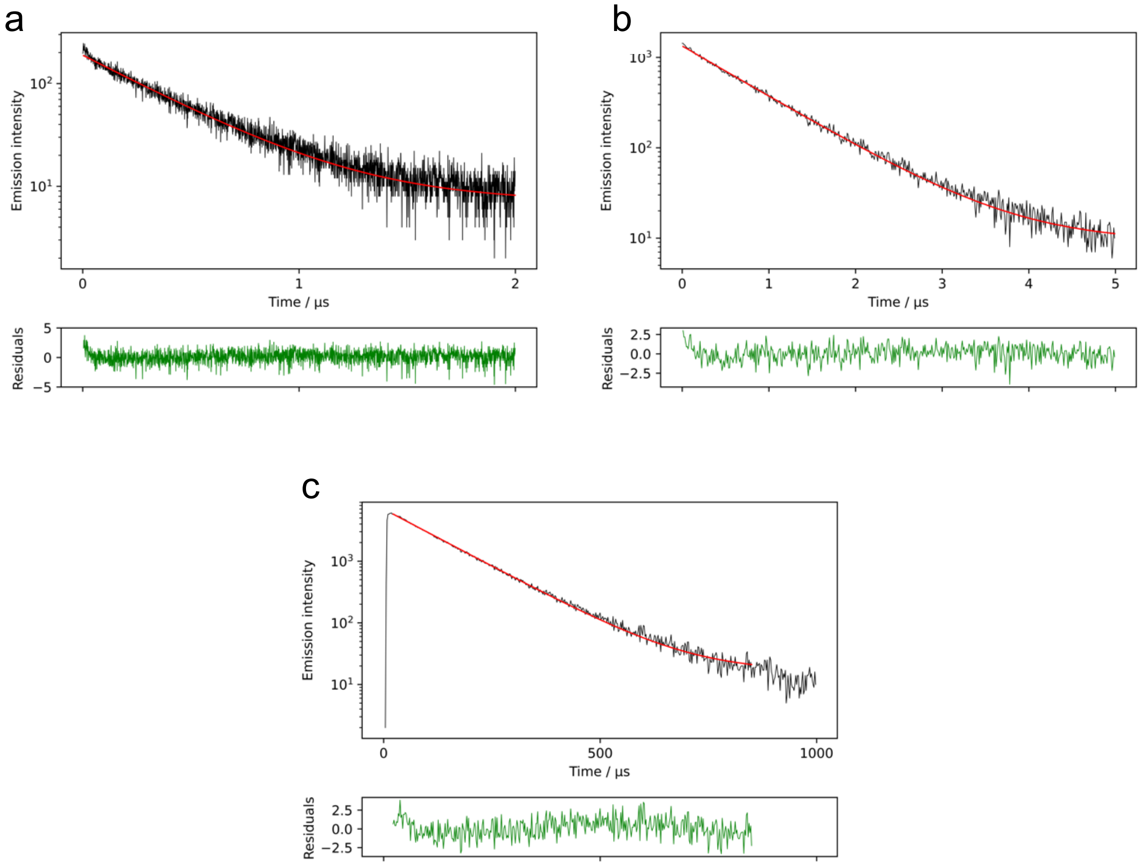


***Figure S3.12*** *Emission decay traces of [PtL⁵(3-OMeppy)Cl]PF₆ in (a) air-equilibrated acetonitrile at 295 K ($\chi^2$ = 1.112); (b) degassed acetonitrile at 295 K ($\chi^2$ = 1.040); and (c) butyronitrile at 77K ($\chi^2$ = 1.711). Experimental data in black; monoexponential fitting in red; residuals in green.*

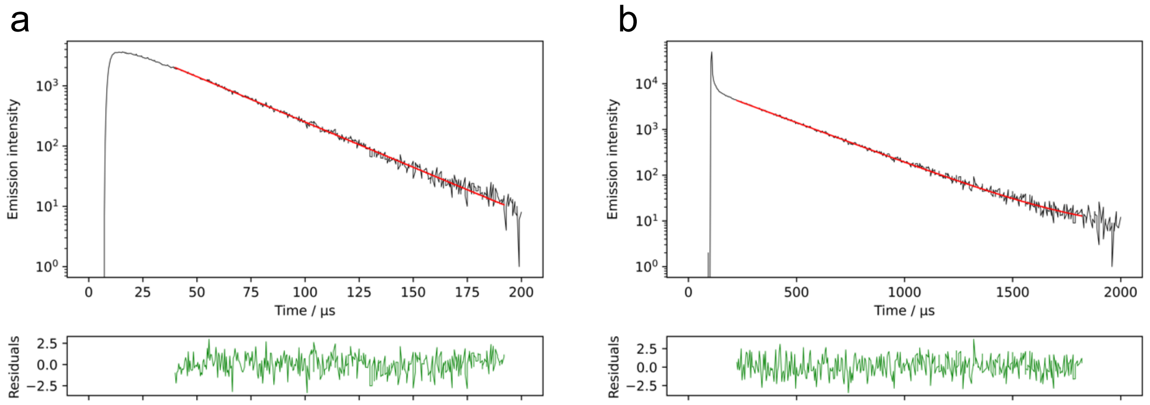


***Figure S3.13*** *Emission decay traces of [Pt(L$^2$)$_2$][PF$_6$]$_2$ in (a) degassed acetonitrile at 295 K ($\chi^2$ = 1.554); and (b) butyronitrile at 77K ($\chi^2$ = 1.786). Experimental data in black; monoexponential fitting in red; residuals in green.*

## Section 4    $^{1}$H and $^{13}$C NMR spectra

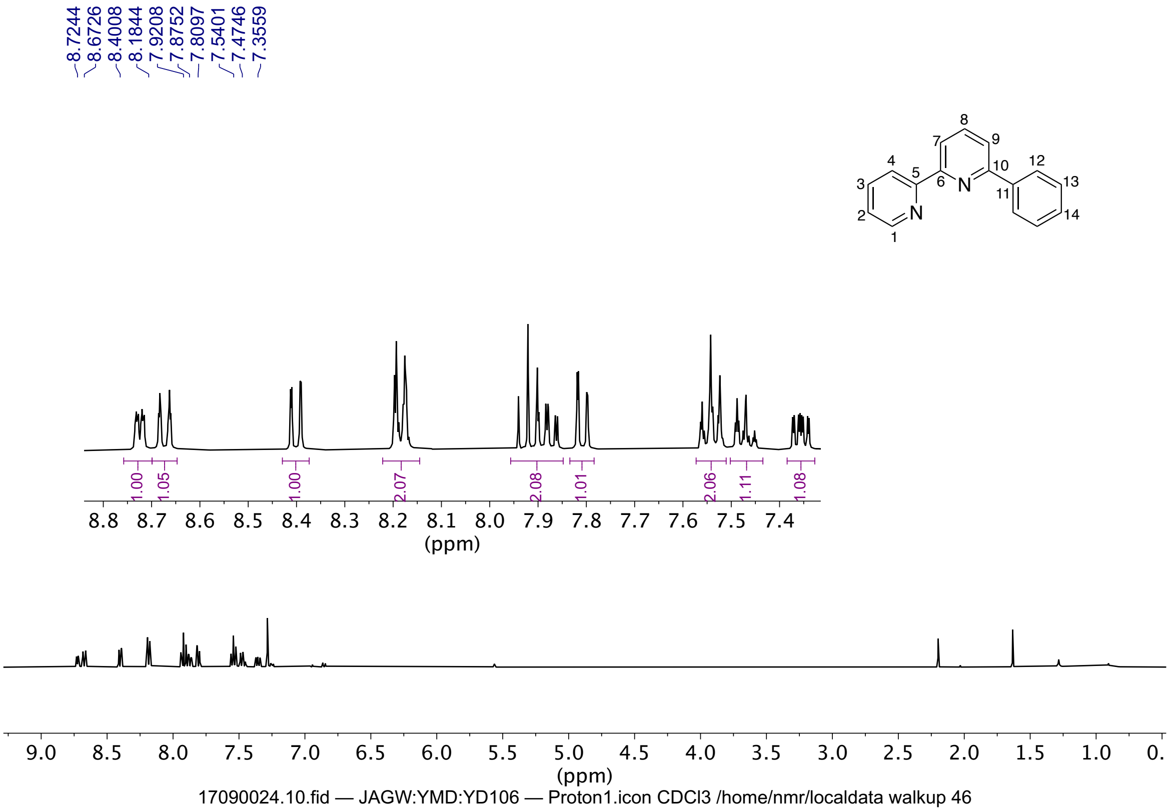


***Figure S4.1*** *$^{1}$H NMR spectrum of $HL^{1}$ in $CDCl_3$*

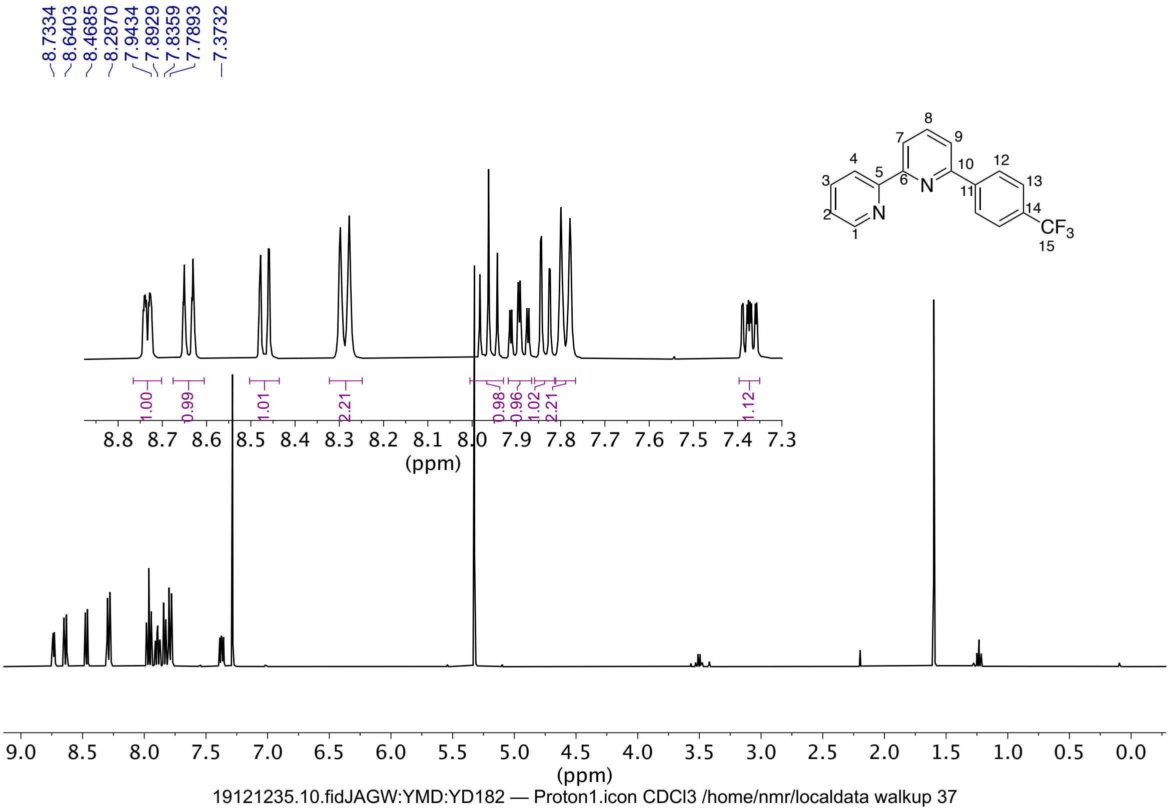


***Figure S4.2*** *$^{1}$H NMR spectrum of $HL^{2}$ in $CDCl_3$*

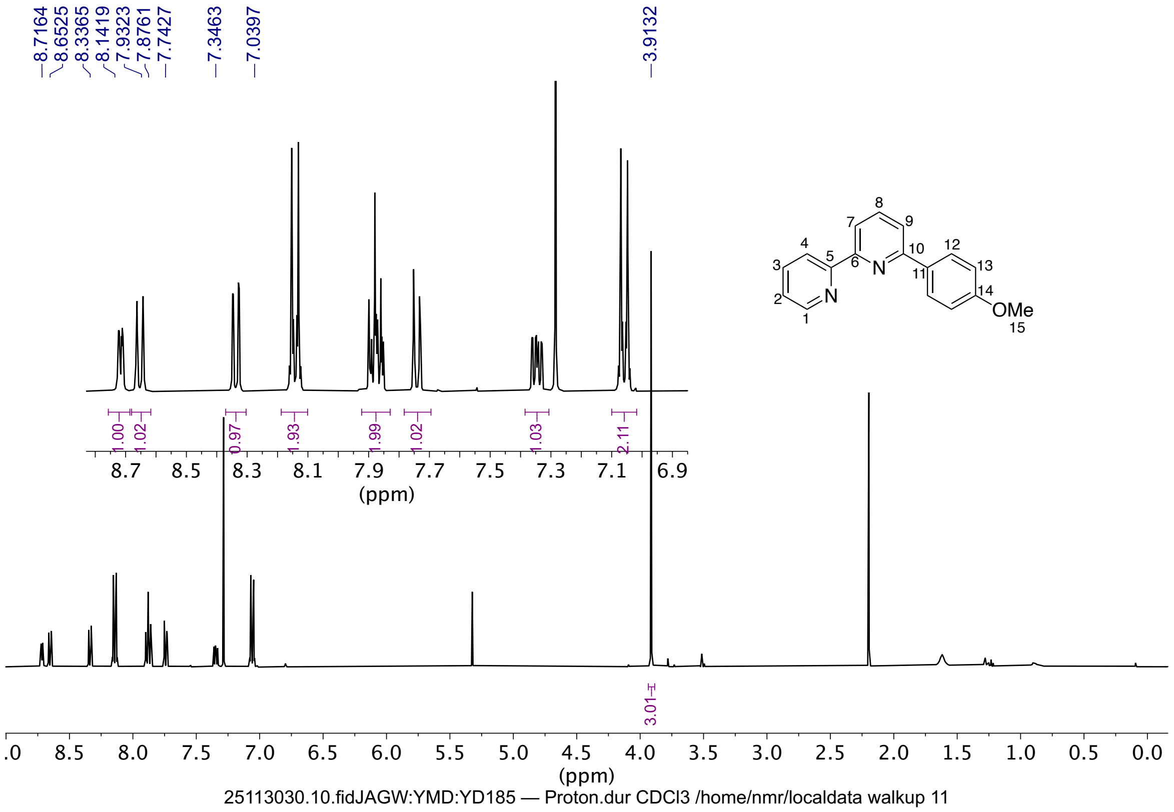

***Figure S4.3*** *$^{1}$H NMR spectrum of HL[4] in CDCl₃*

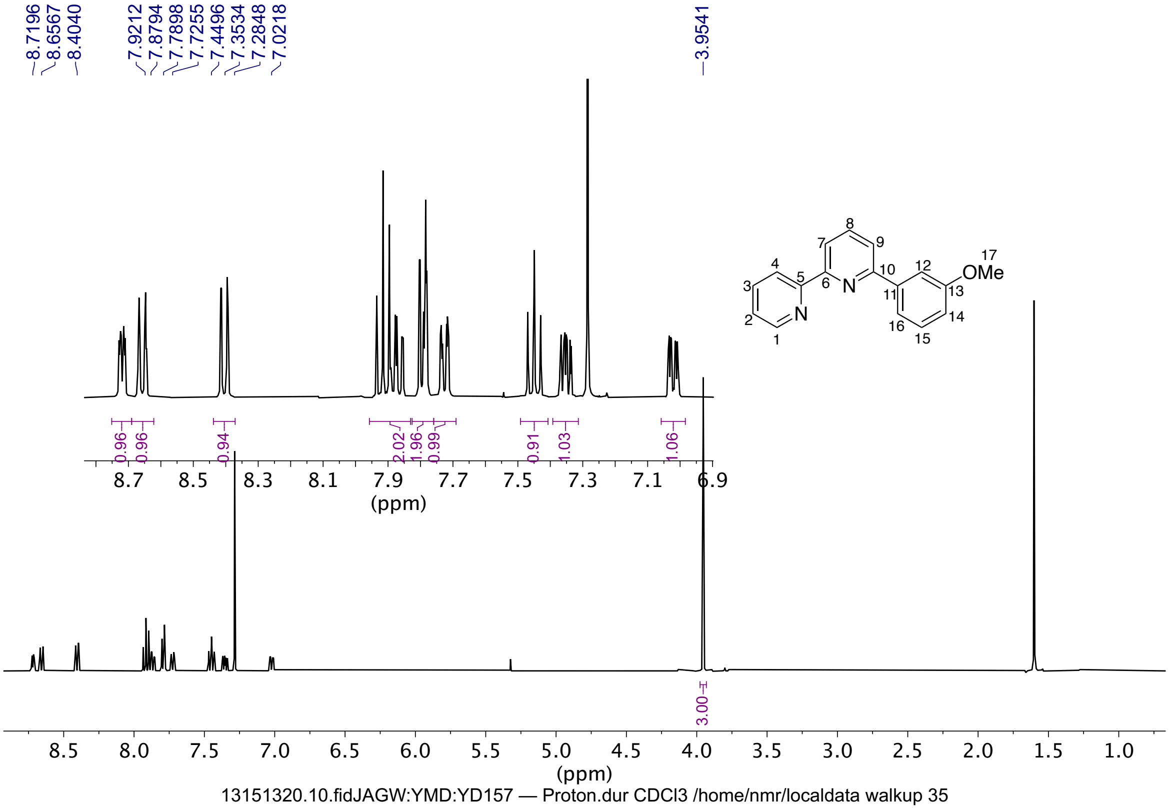

***Figure S4.4*** *$^{1}$H NMR spectrum of HL[5] in CDCl₃*

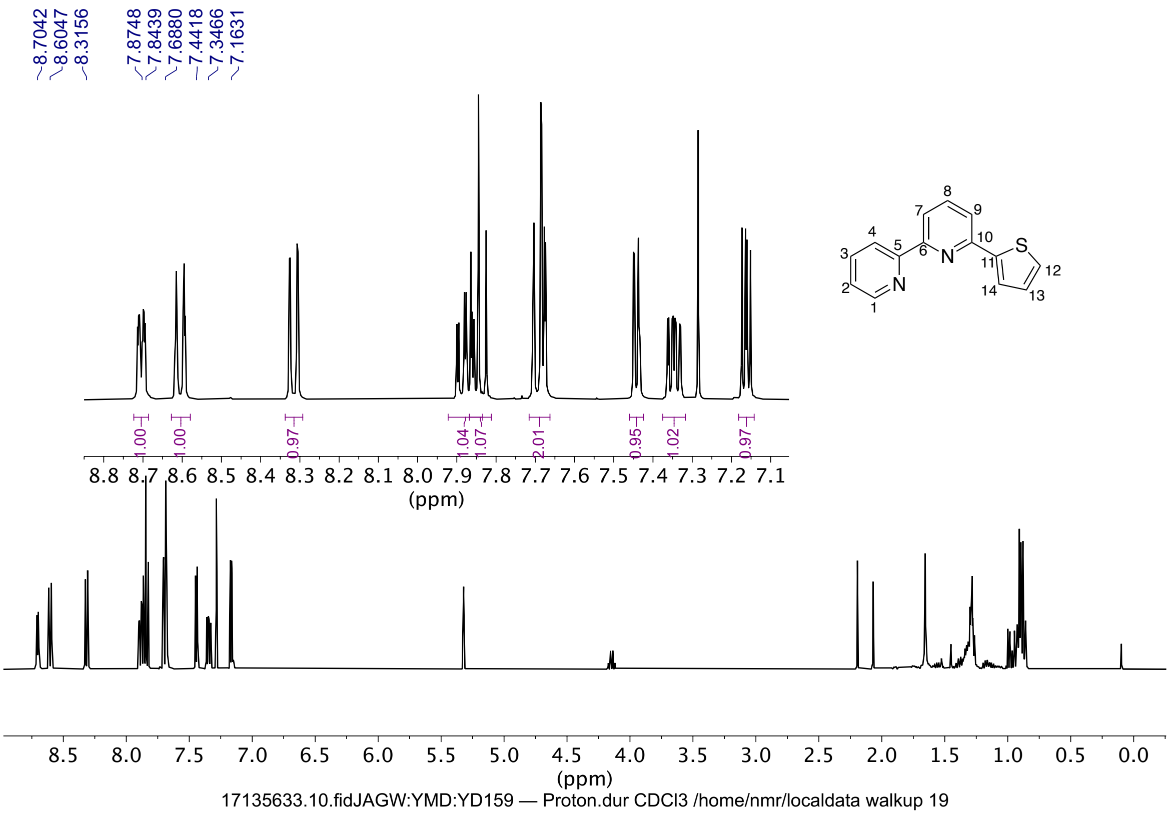


***Figure S4.5*** *$^{1}H$ NMR spectrum of $HL^{6}$ in $CDCl_3$*

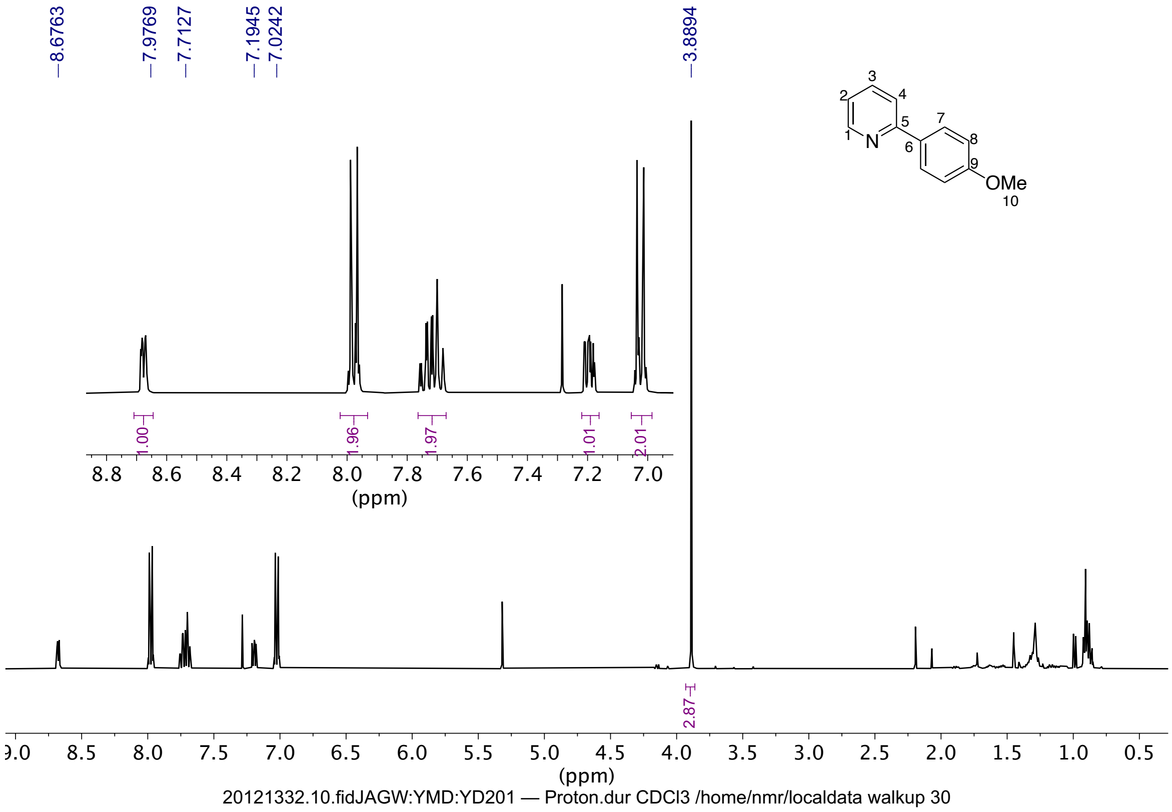


***Figure S4.6*** *$^{1}H$ NMR spectrum of 4-MeOppy in $CDCl_3$*

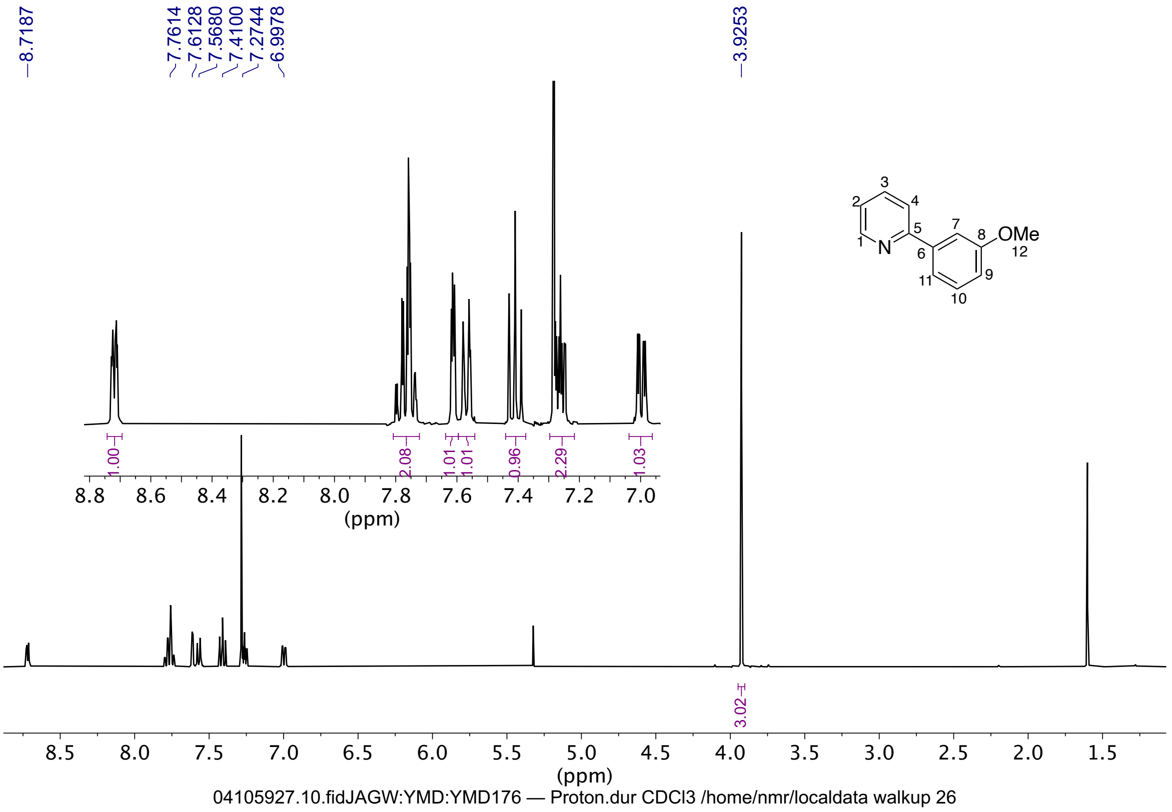


***Figure S4.7*** *$^{1}$H NMR spectrum of 3-MeOppy in $CDCl_3$*

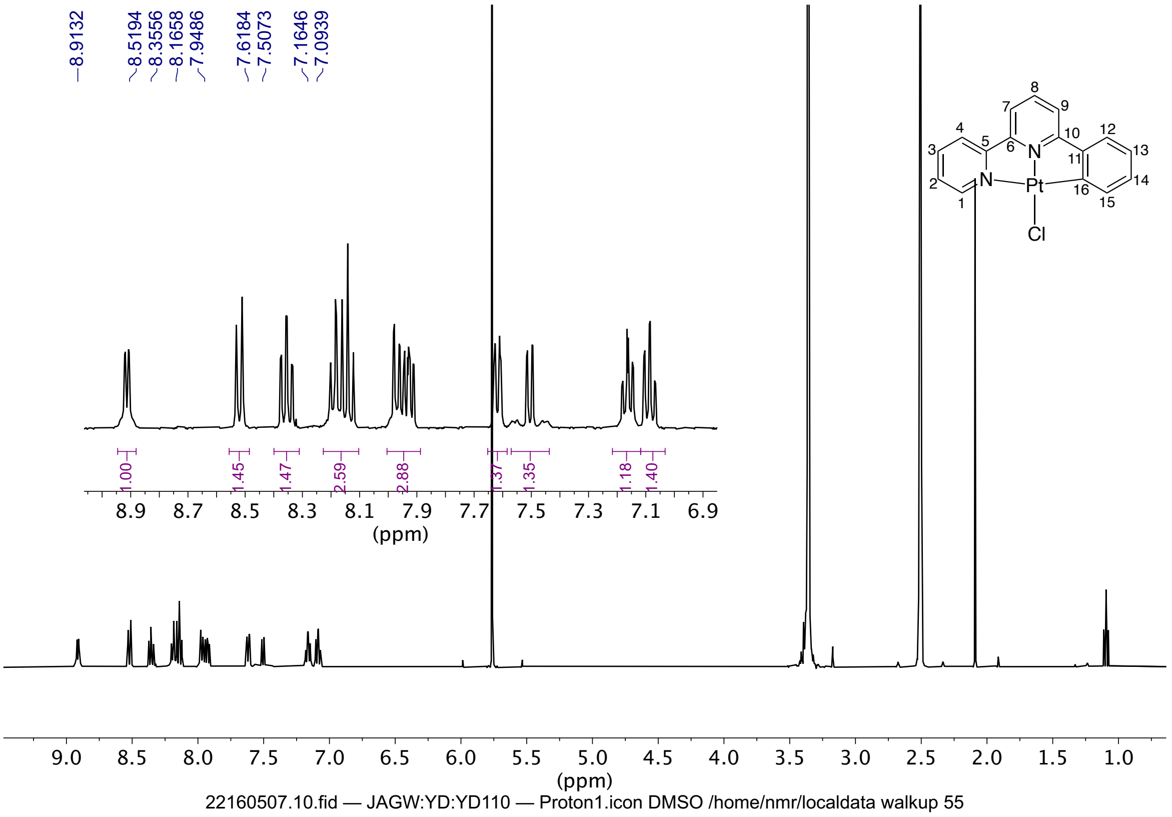


***Figure S4.8*** *$^{1}$H NMR spectrum of $PtL^1Cl$ in $d_6$-DMSO*

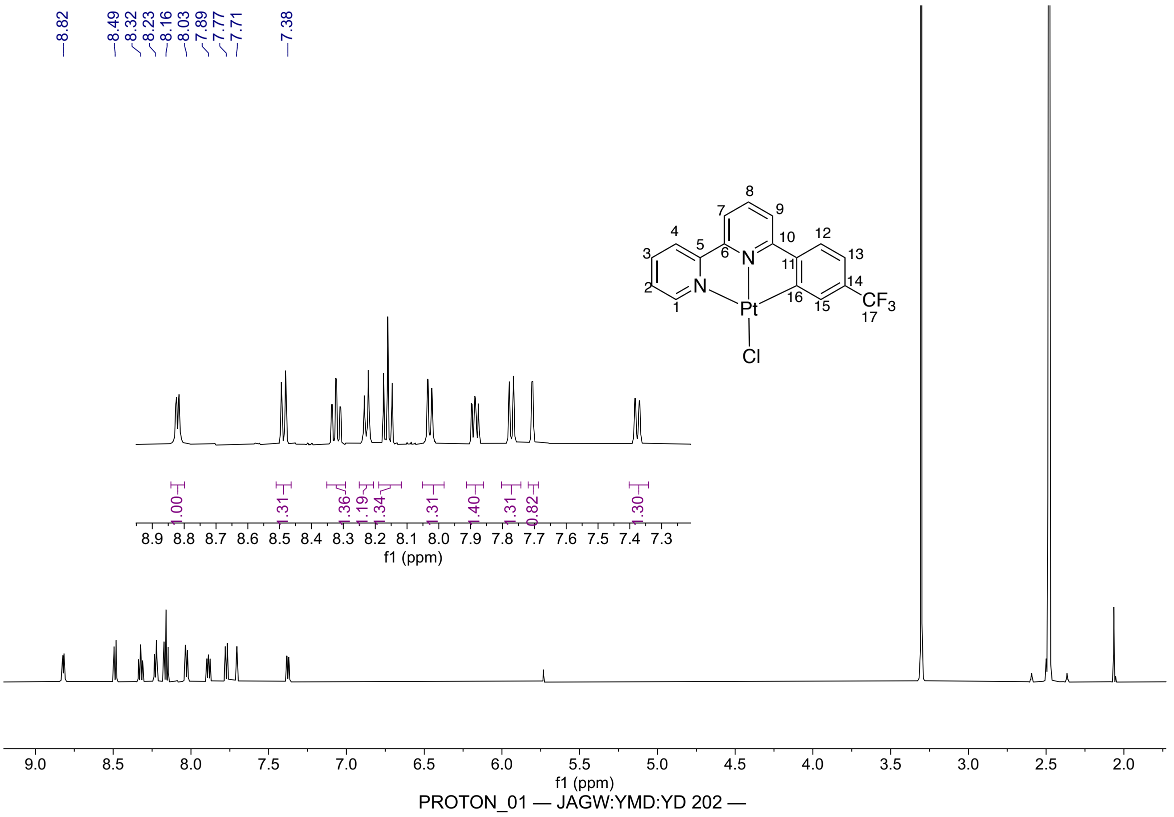


***Figure S4.9*** *$^{1}H$ NMR spectrum of $PtL^{2}Cl$ in $d_6$-DMSO*

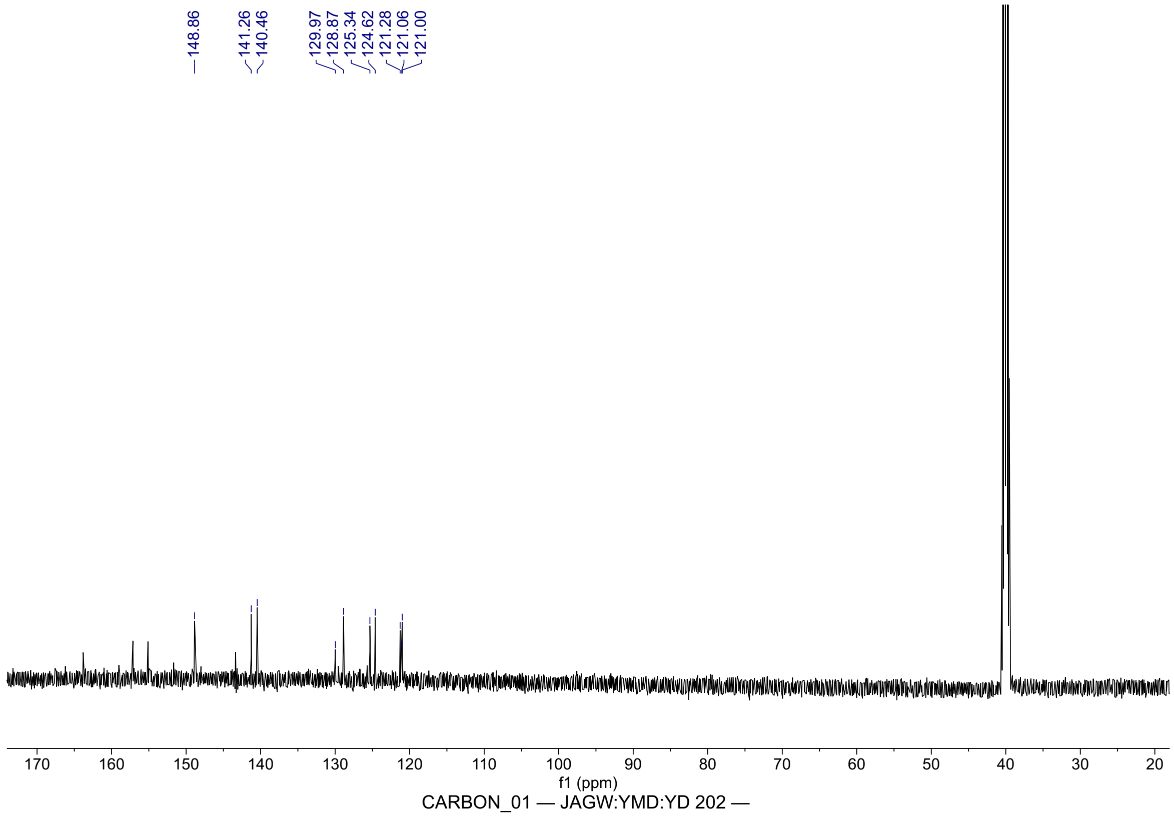


***Figure S4.10*** *$^{13}C$ NMR spectrum of $PtL^{2}Cl$ in $d_6$-DMSO*

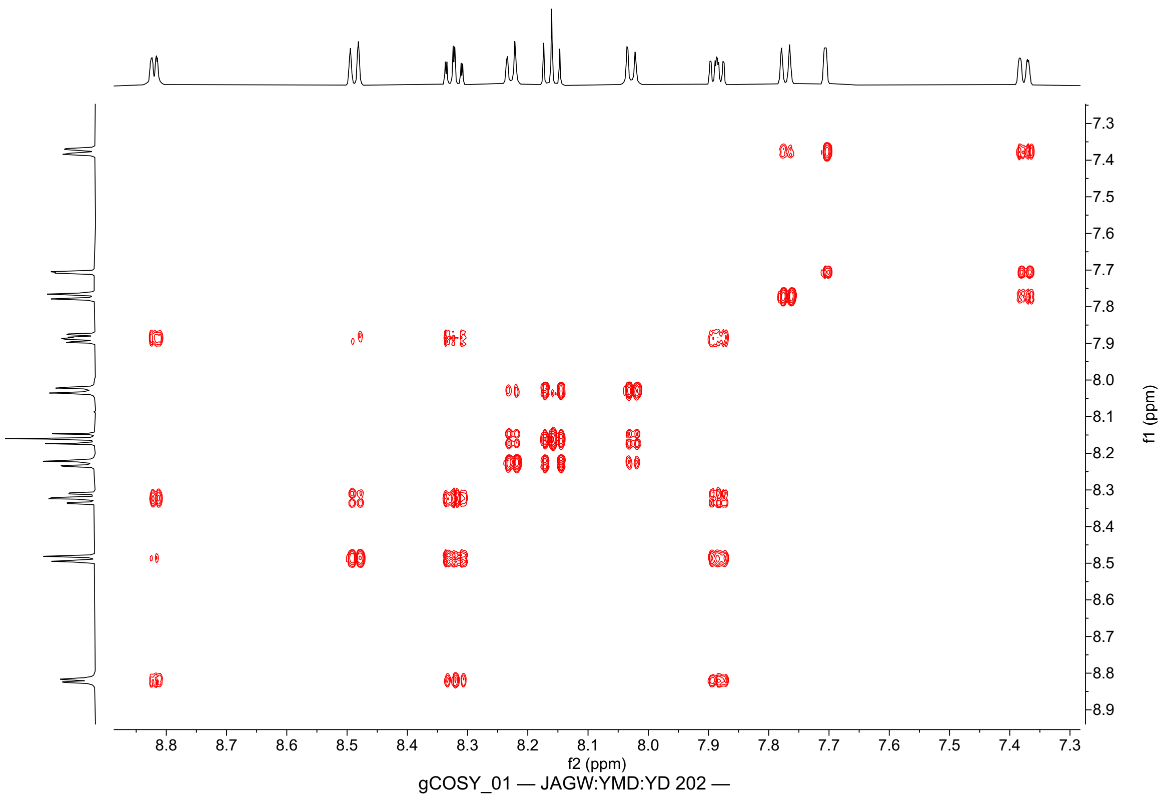


***Figure S4.11*** *COSY NMR spectrum of $PtL^2Cl$ in $d_6$-DMSO*

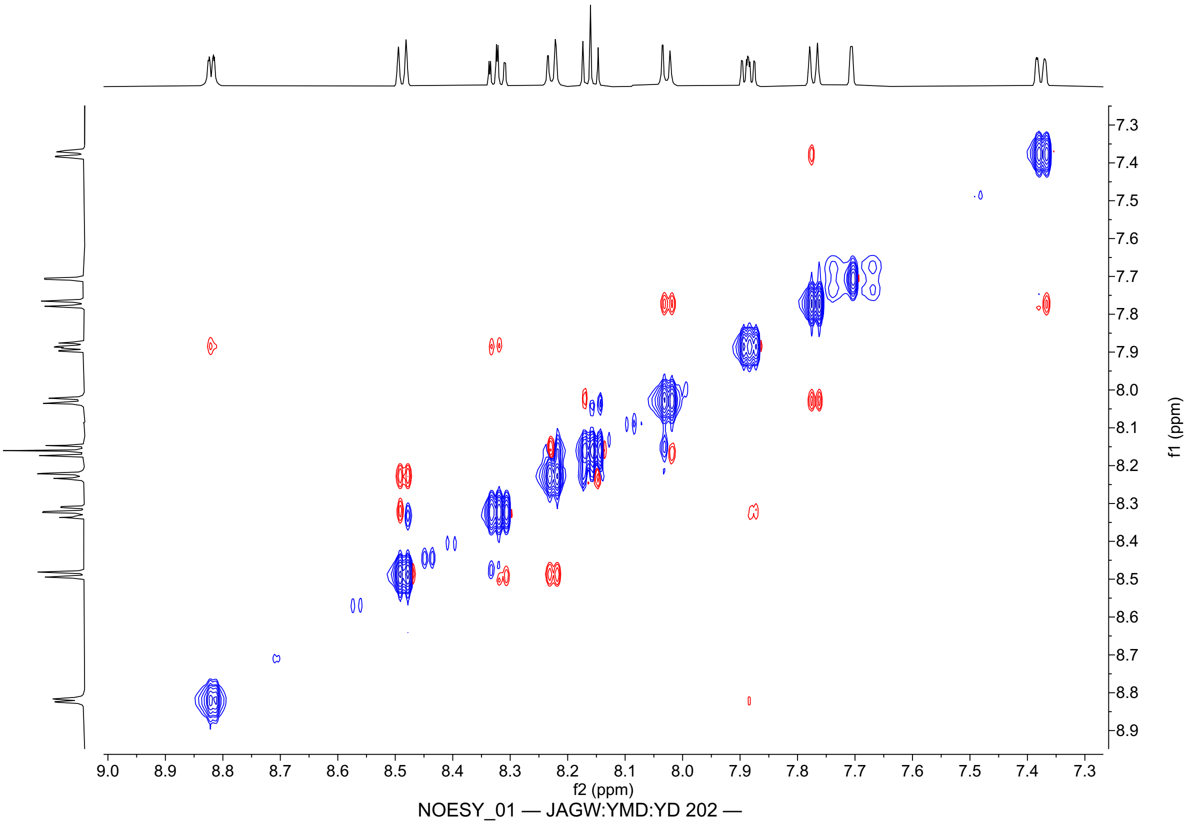


***Figure S4.12*** *NOESY NMR spectrum of $PtL^2Cl$ in $d_6$-DMSO*

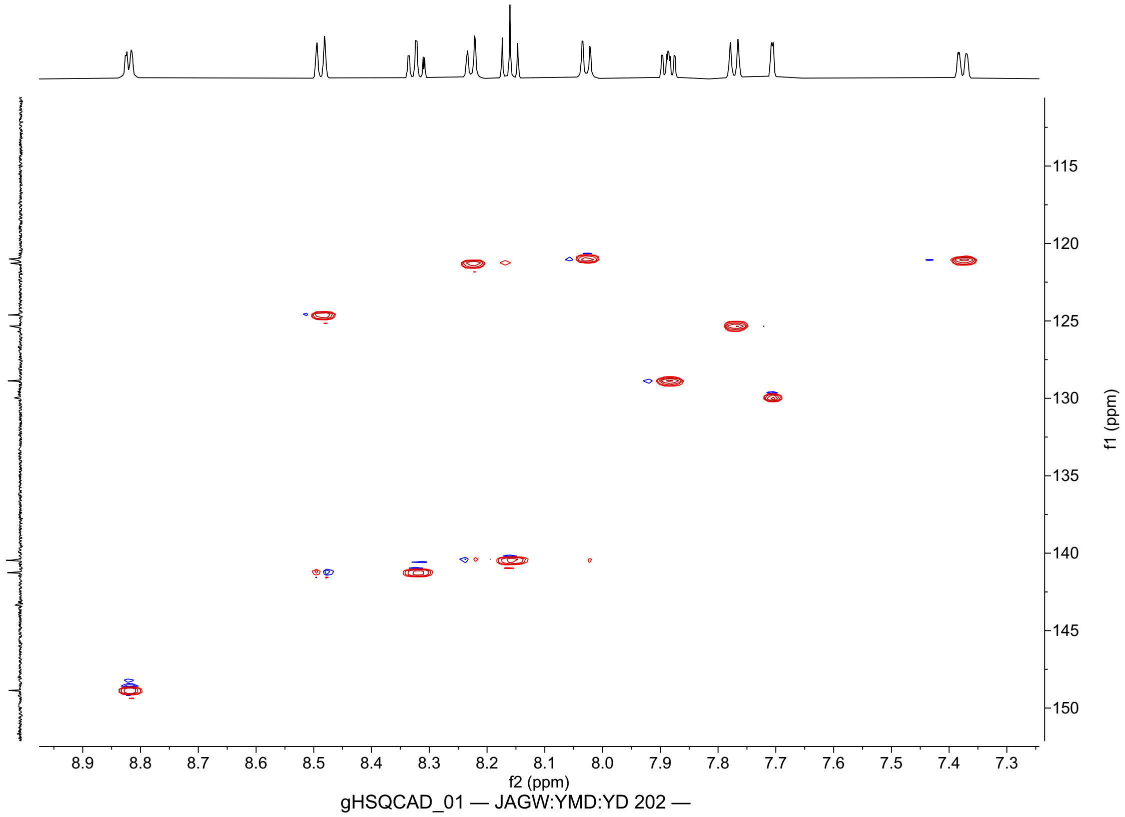


***Figure S4.13*** *HSQC NMR spectrum of $PtL^2Cl$ in $d_6$-DMSO*

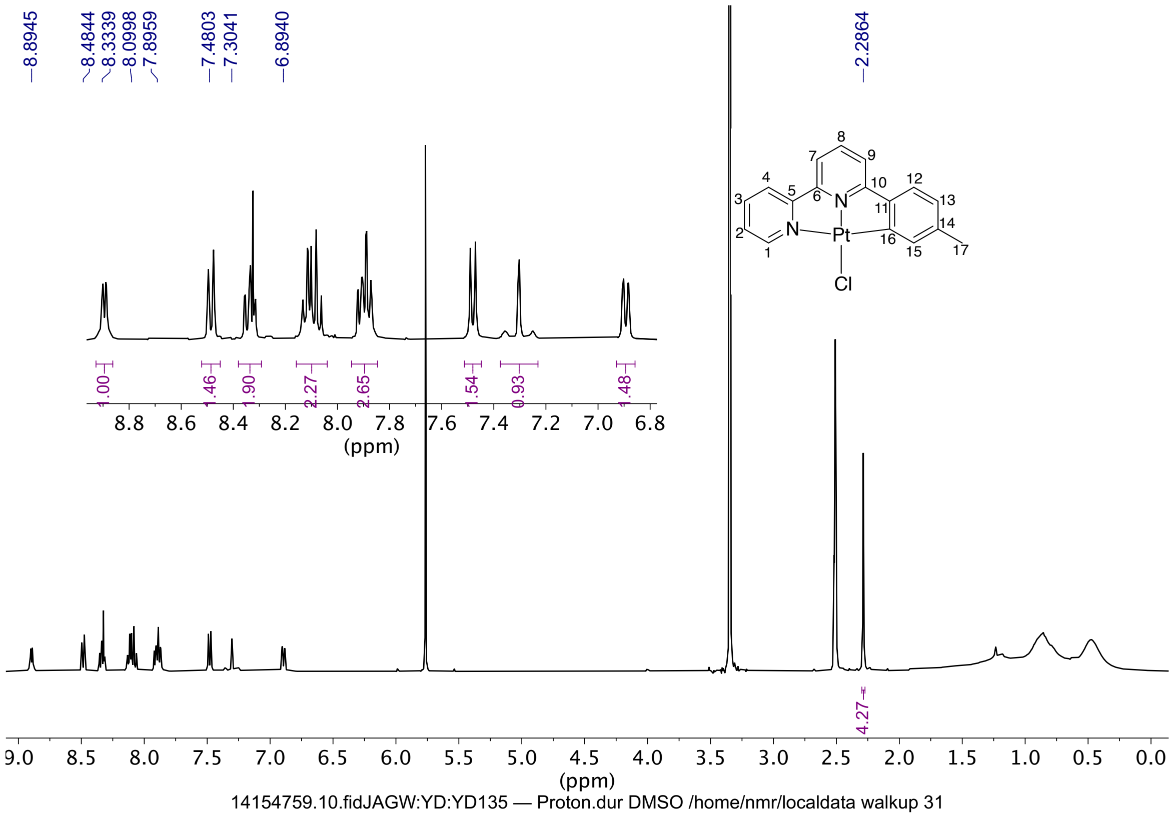


***Figure S4.14*** *$^1H$ NMR spectrum of $PtL^3Cl$ in $d_6$-DMSO*

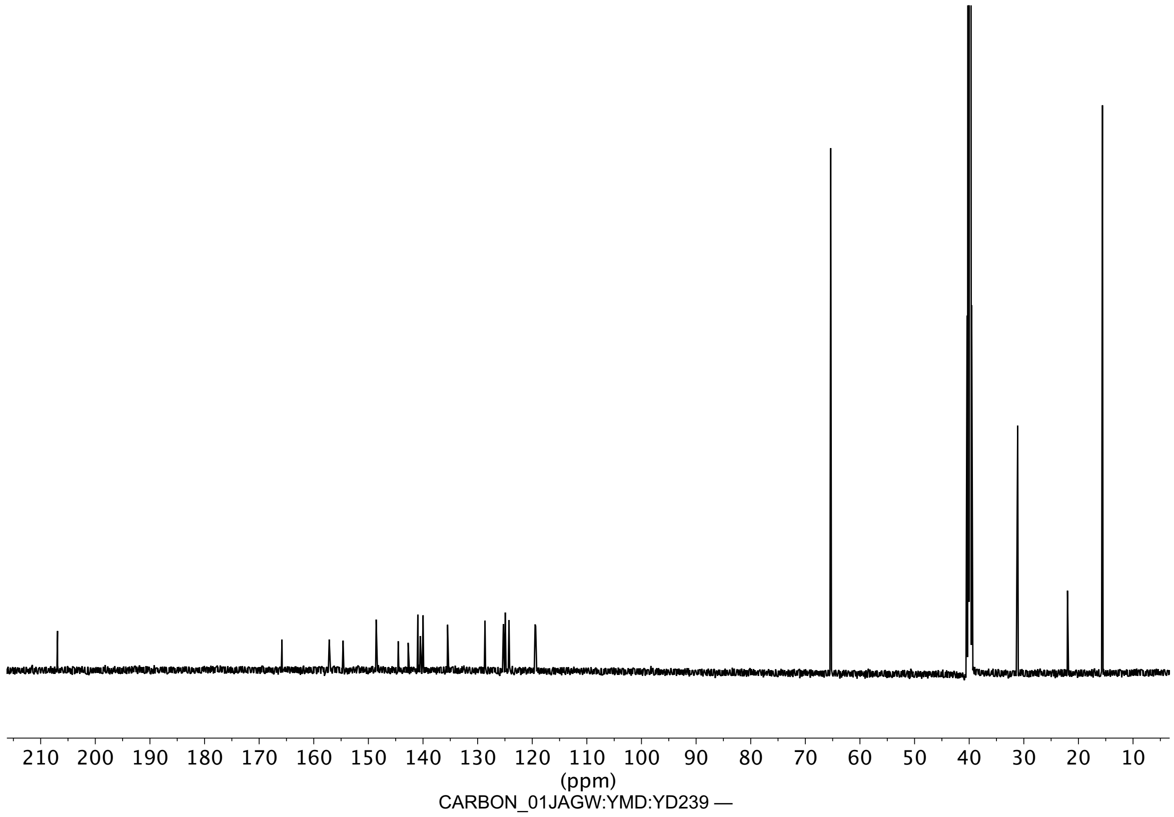


***Figure S4.15*** *$^{13}$C NMR spectrum of PtL$^{3}$Cl in d$_{6}$-DMSO*

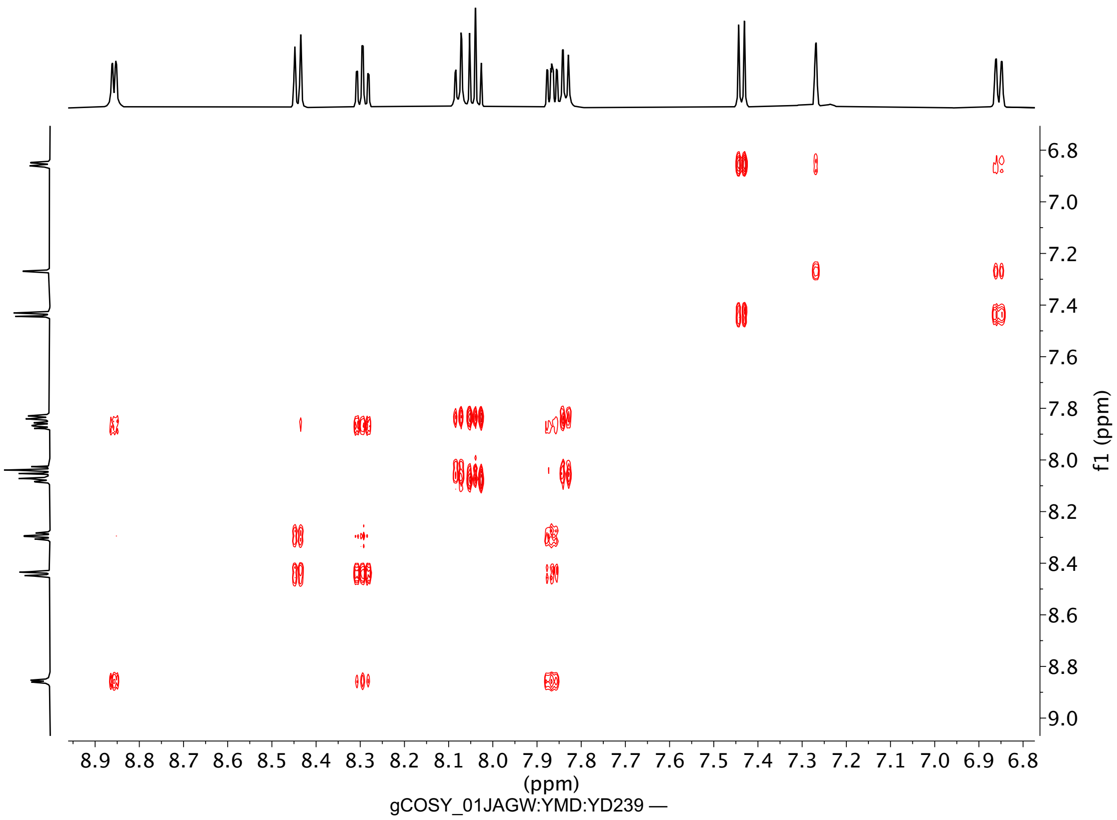


***Figure S4.16*** *COSY NMR spectrum of PtL$^{3}$Cl in d$_{6}$-DMSO*

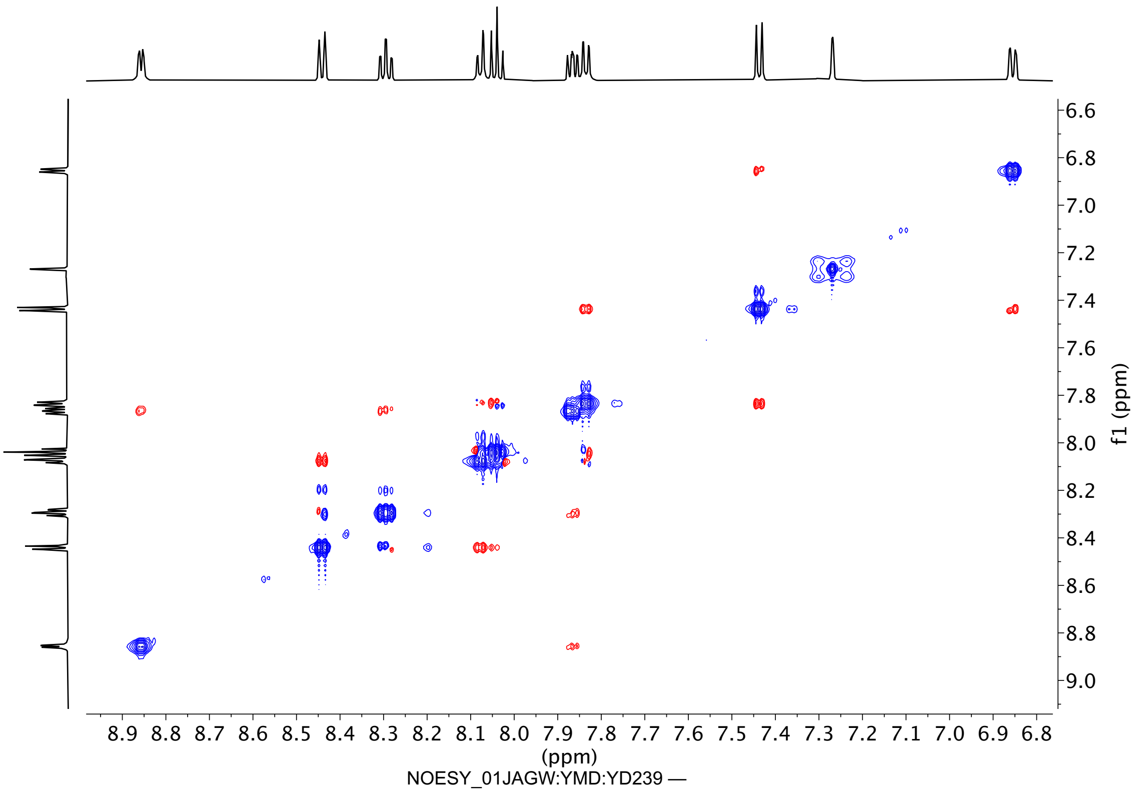


***Figure S4.17*** *NOESY NMR spectrum of* $PtL^3Cl$ *in* $d_6$*-DMSO*

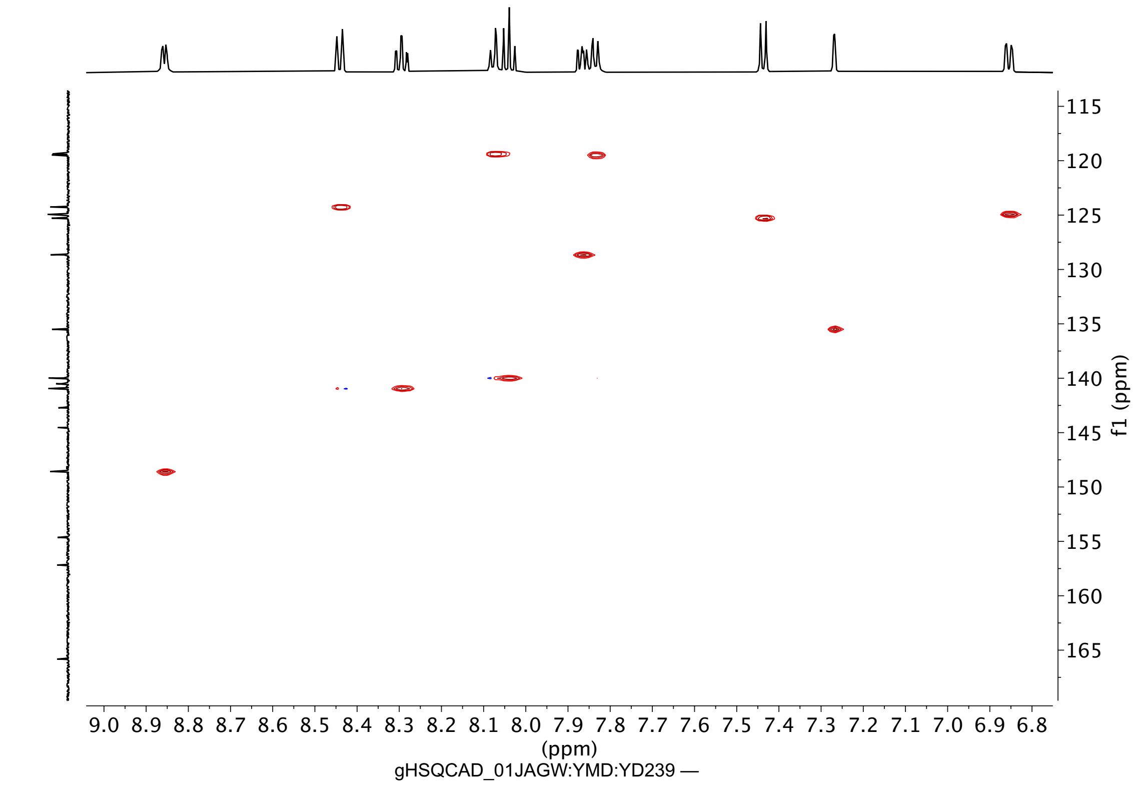


***Figure S4.18*** *HSQC NMR spectrum of* $PtL^3Cl$ *in* $d_6$*-DMSO*

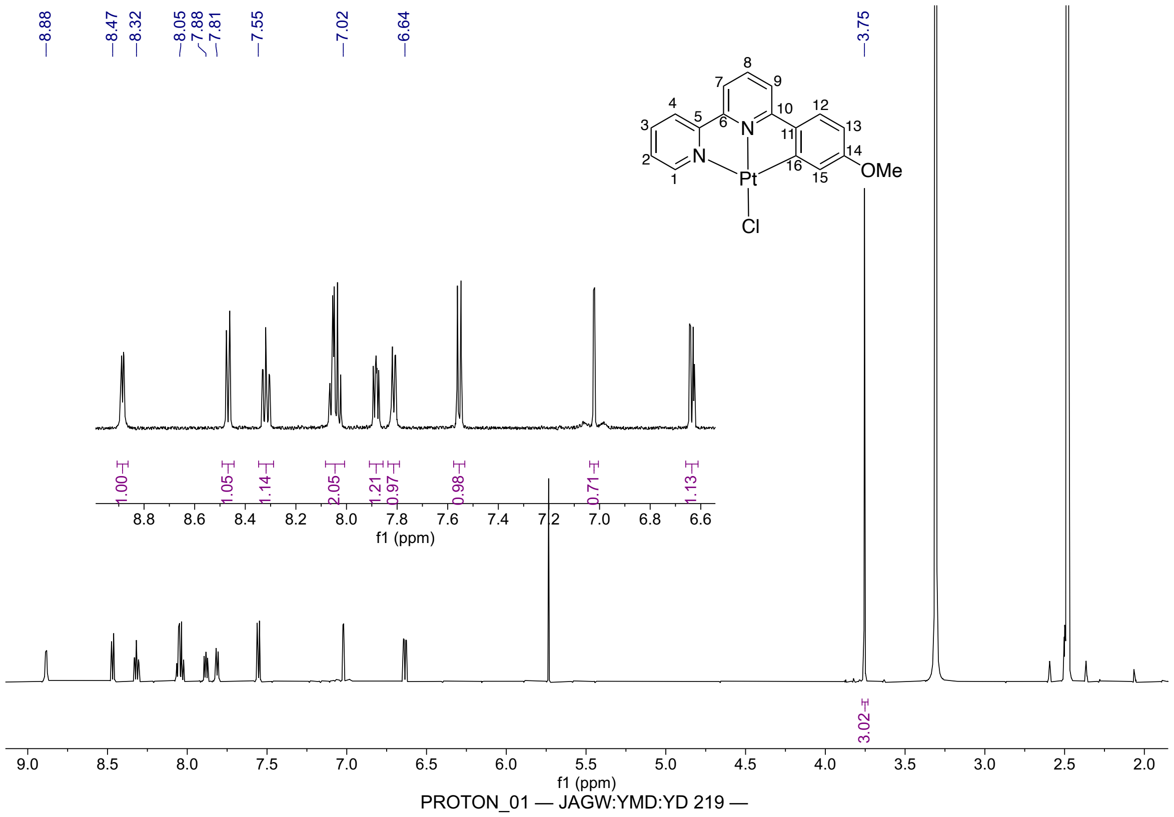


***Figure S4.19*** *$^{1}$H NMR spectrum of PtL$^{4}$Cl in $d_6$-DMSO*

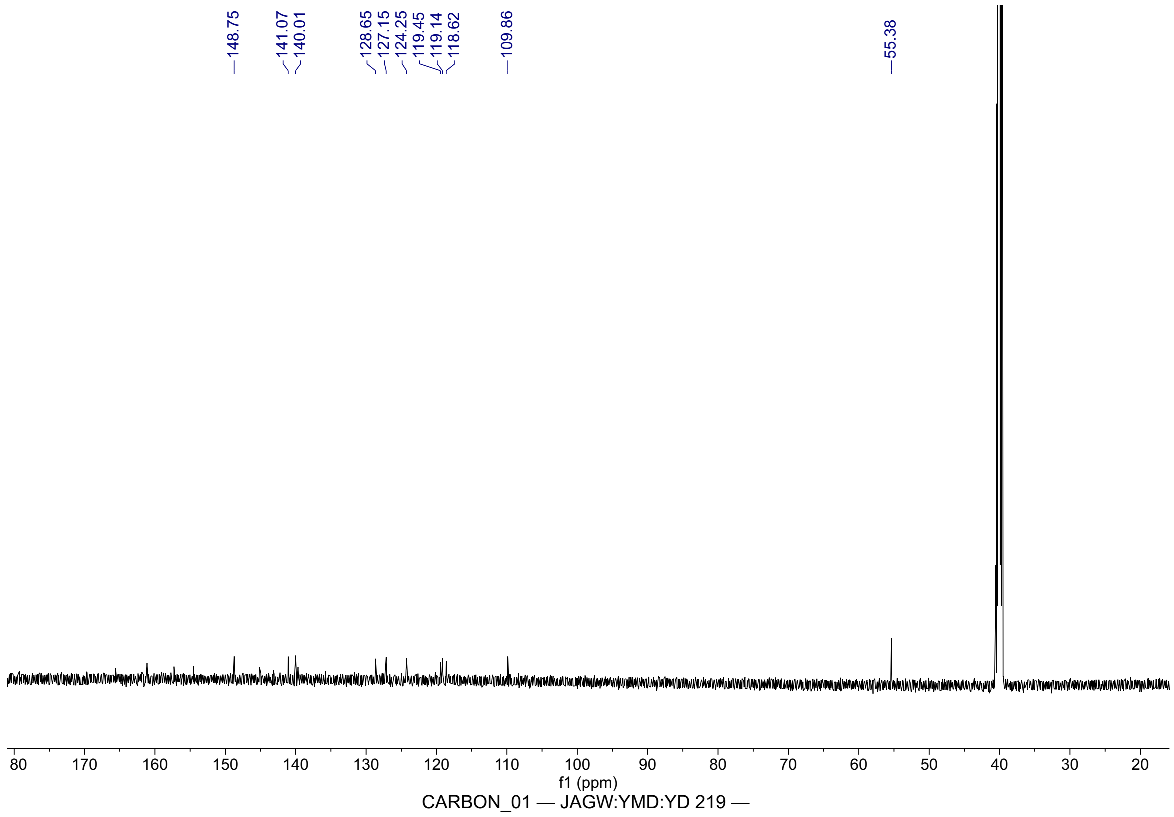


***Figure S4.20*** *$^{13}$C NMR spectrum of PtL$^{4}$Cl in $d_6$-DMSO*

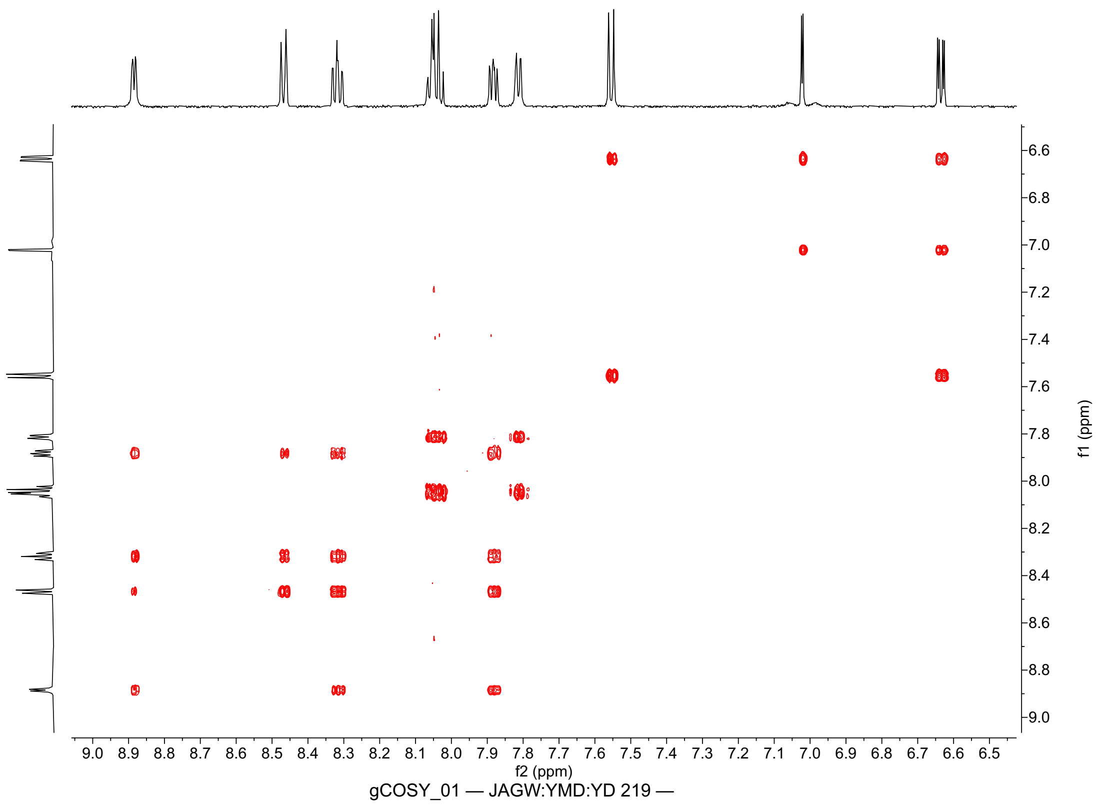


***Figure S4.21*** *COSY NMR spectrum of PtL$^4$Cl in d$_6$-DMSO*

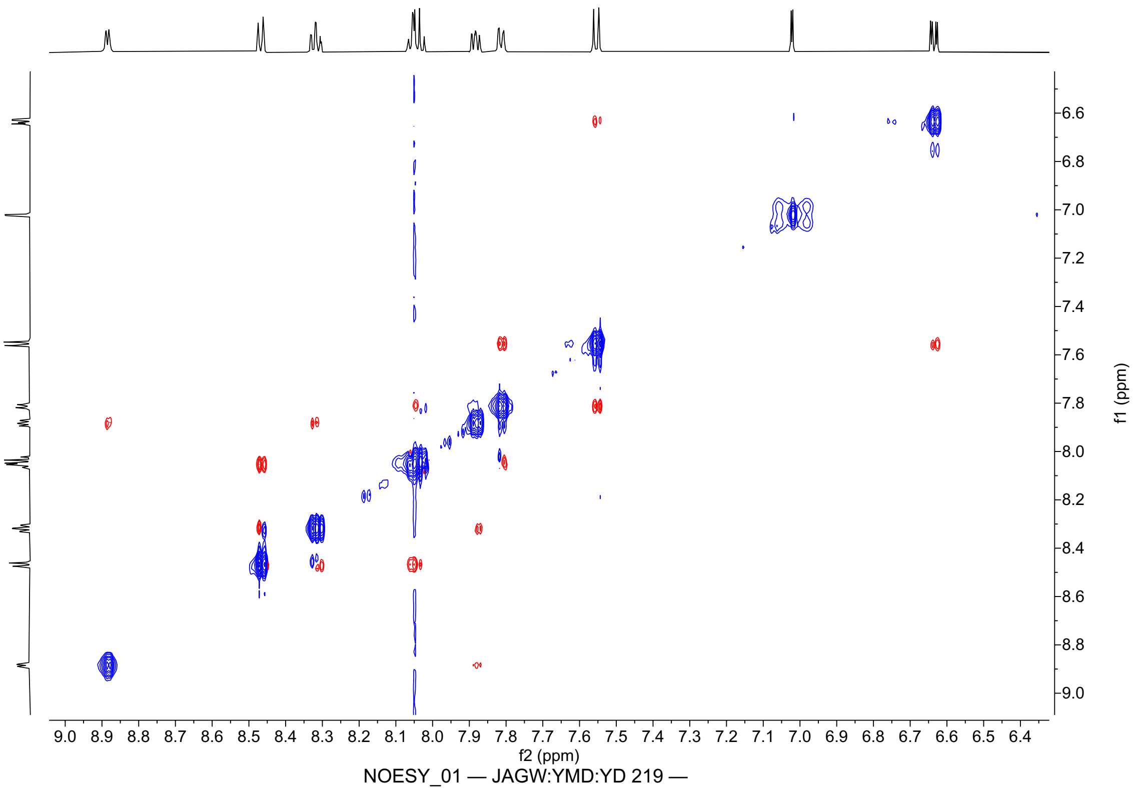


***Figure S4.22*** *NOESY NMR spectrum of PtL$^4$Cl in d$_6$-DMSO*

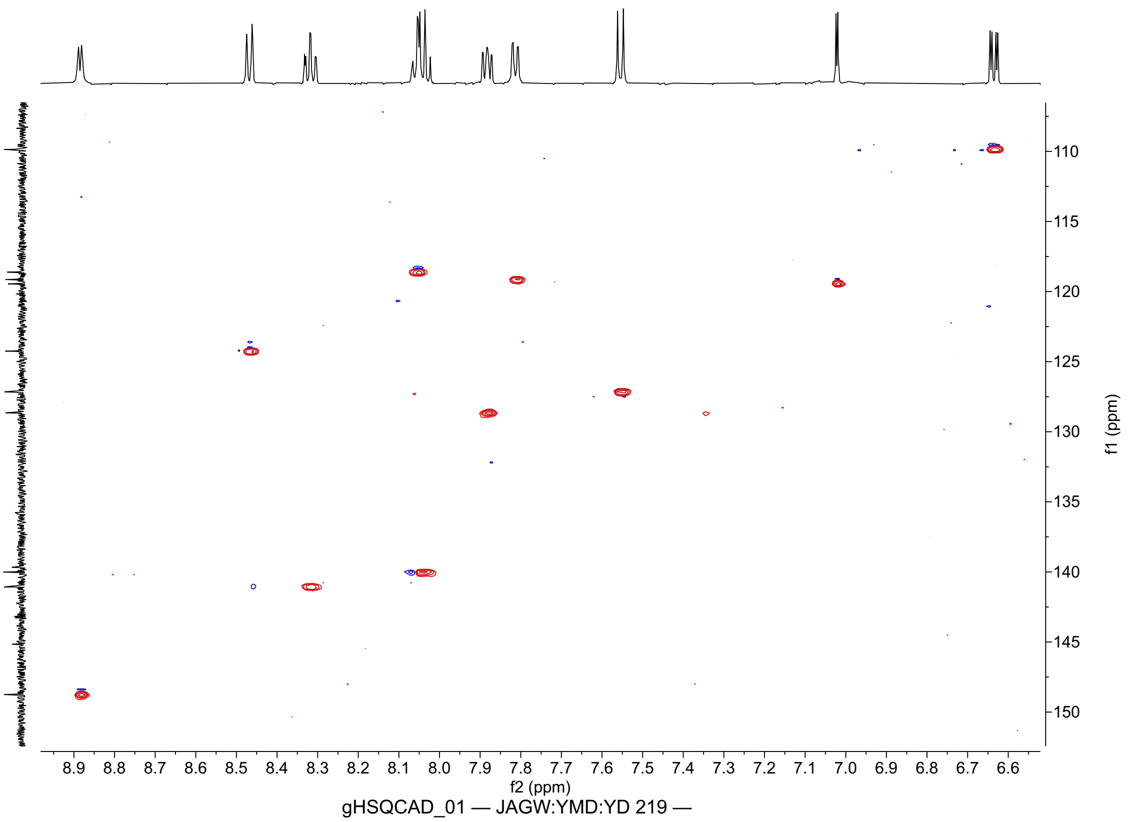


***Figure S4.23*** *HSQC NMR spectrum of $PtL^4Cl$ in $d_6$-DMSO*

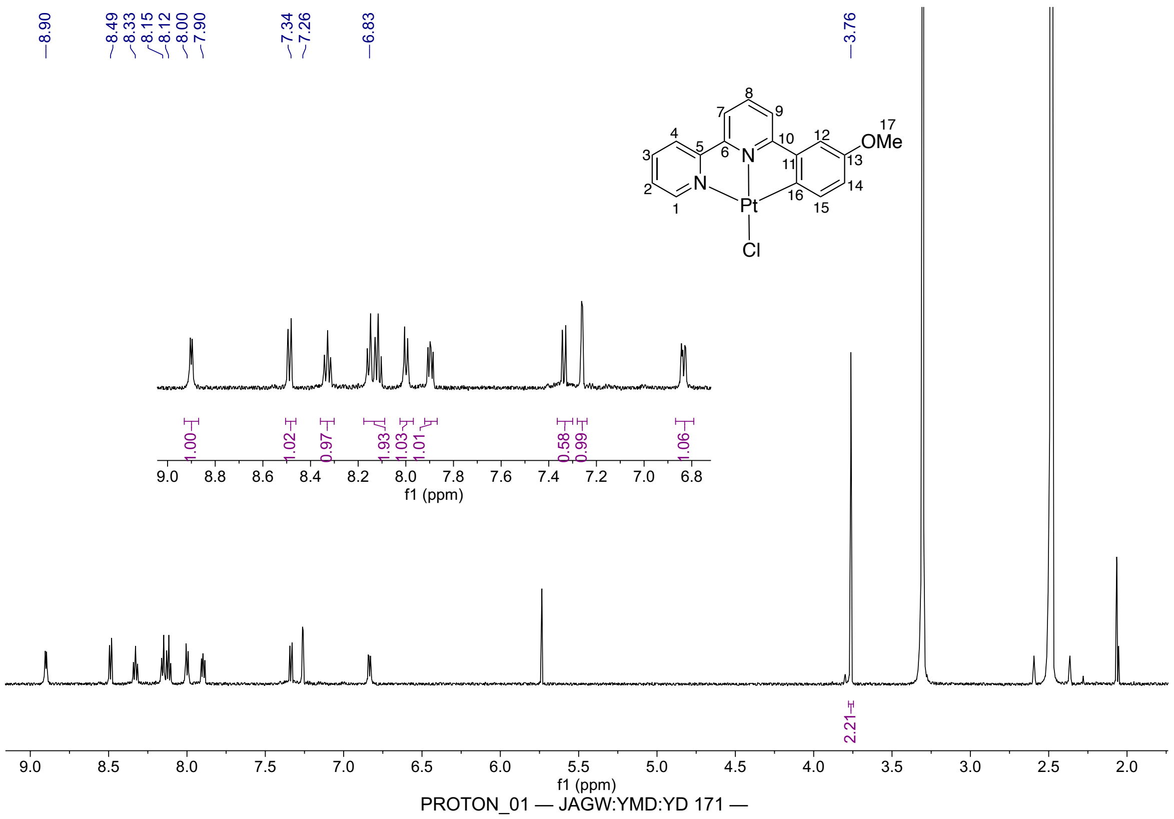


***Figure S4.24*** *$^1H$ NMR spectrum of $PtL^5Cl$ in $d_6$-DMSO*

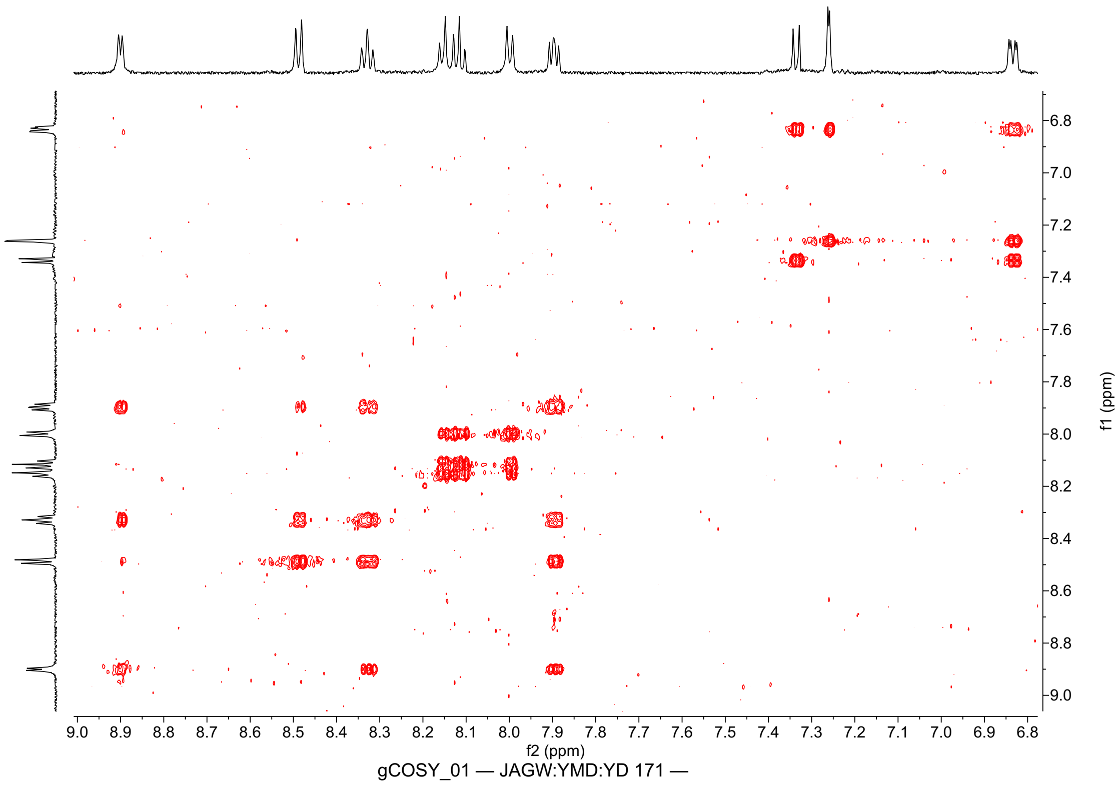


***Figure S4.25*** *COSY NMR spectrum of PtL$^5$Cl in d$_6$-DMSO*

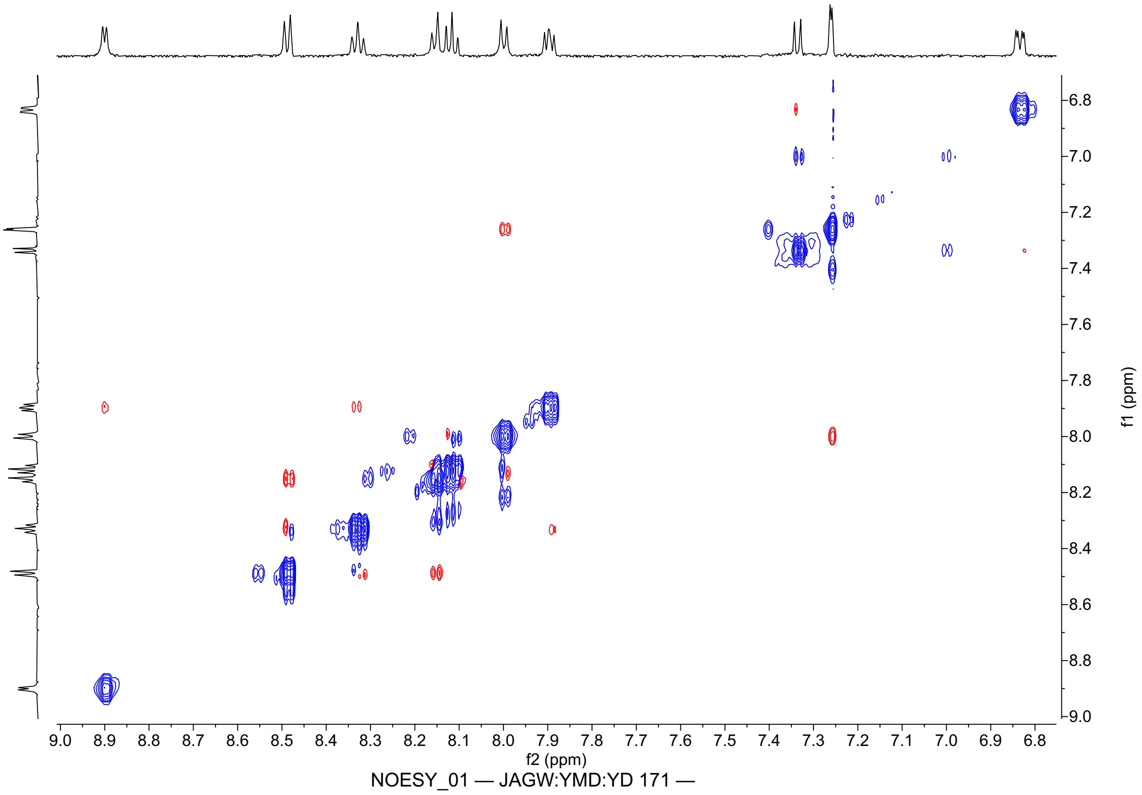


***Figure S4.26*** *NOESY NMR spectrum of PtL$^5$Cl in d$_6$-DMSO*

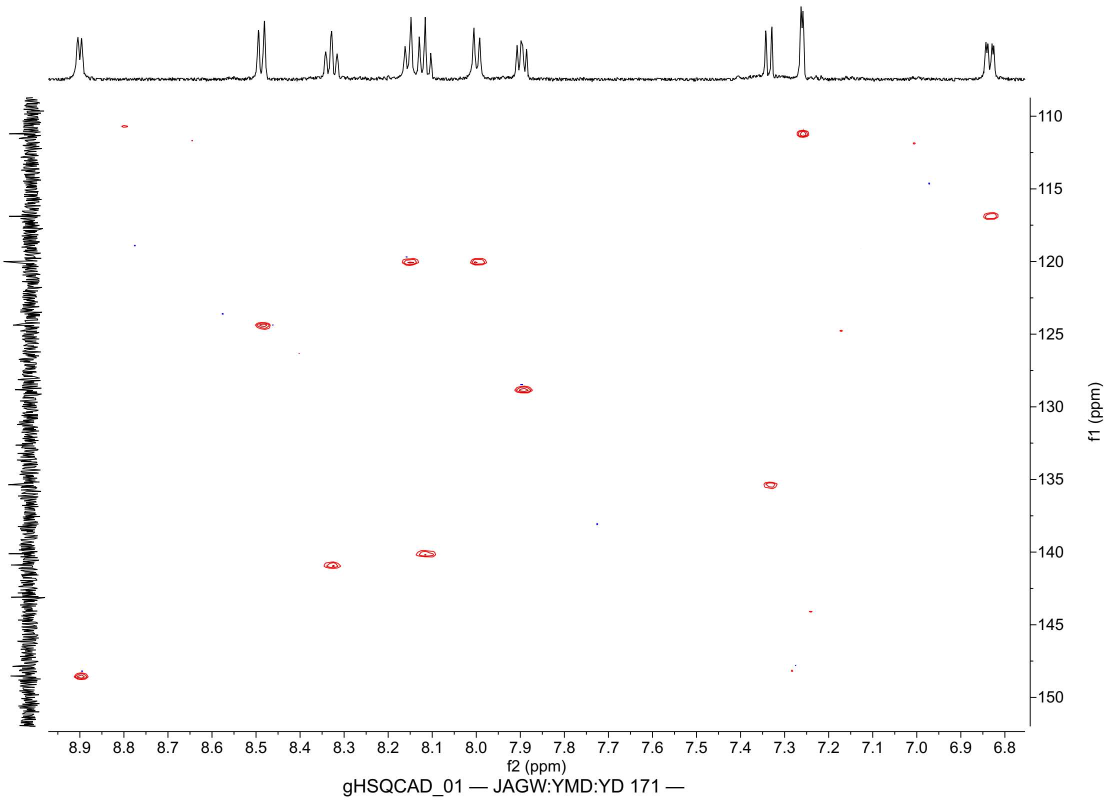


***Figure S4.27*** *HSQC NMR spectrum of PtL⁵Cl in $d_6$-DMSO*

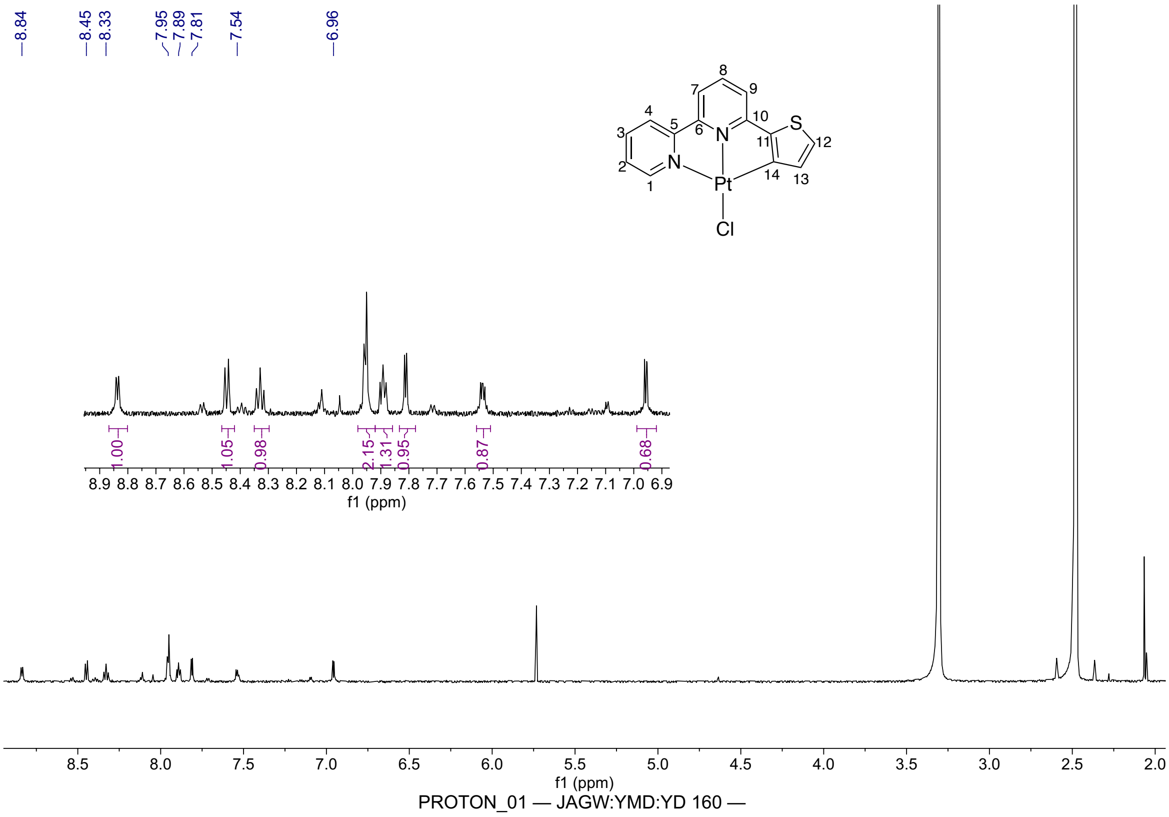


***Figure S4.28*** *$^1$H NMR spectrum of PtL⁶Cl in $d_6$-DMSO*

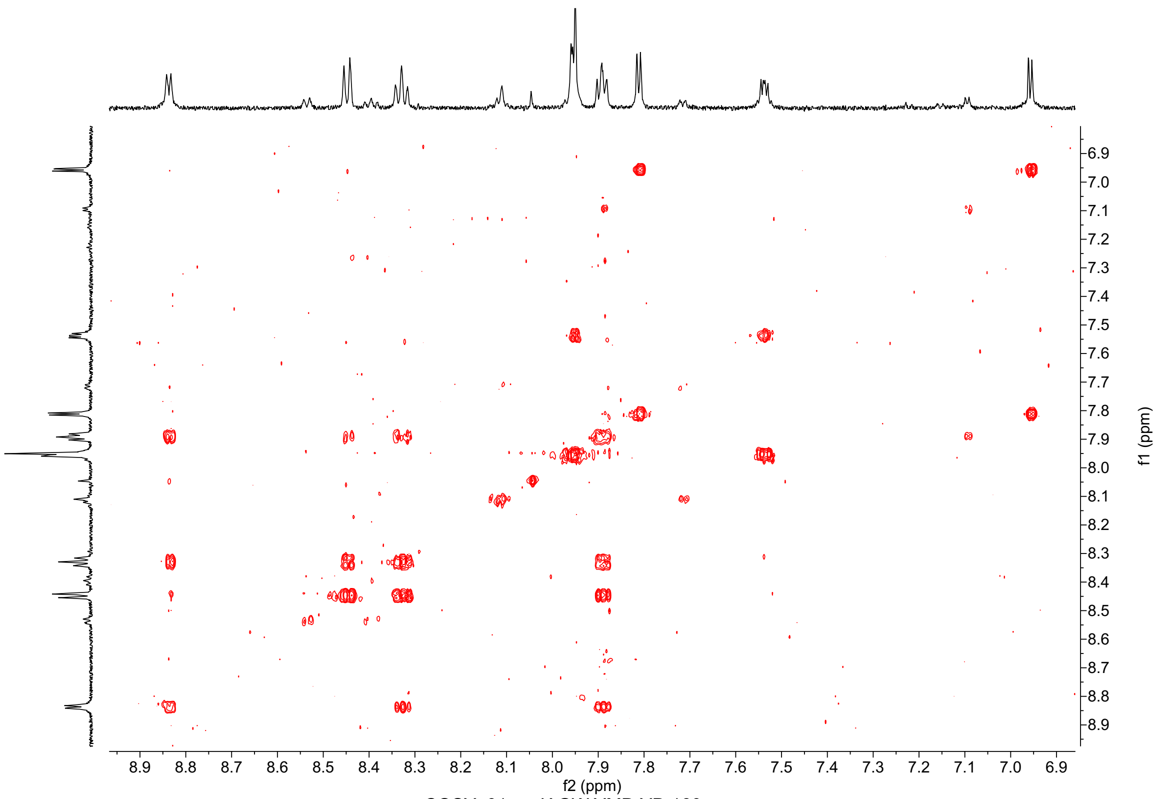


***Figure S4.29*** *COSY NMR spectrum of PtL$^6$Cl in d$_6$-DMSO*

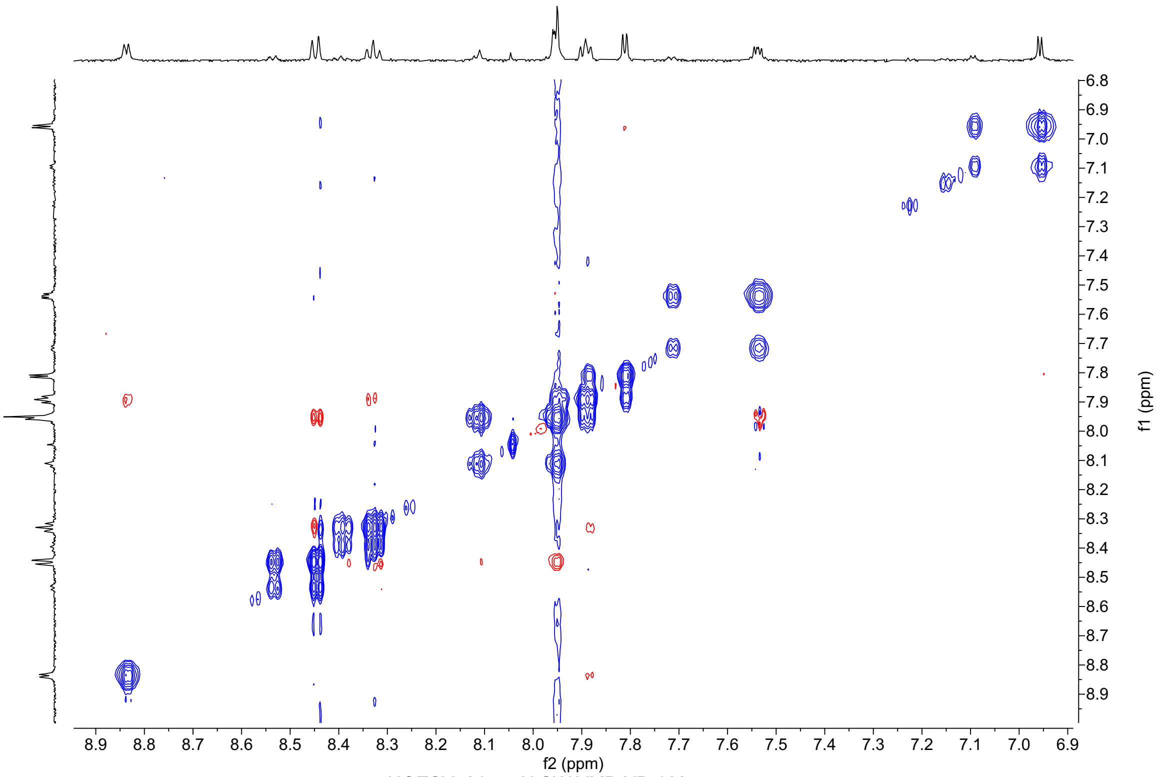


***Figure S4.30*** *NOESY NMR spectrum of PtL$^6$Cl in d$_6$-DMSO*

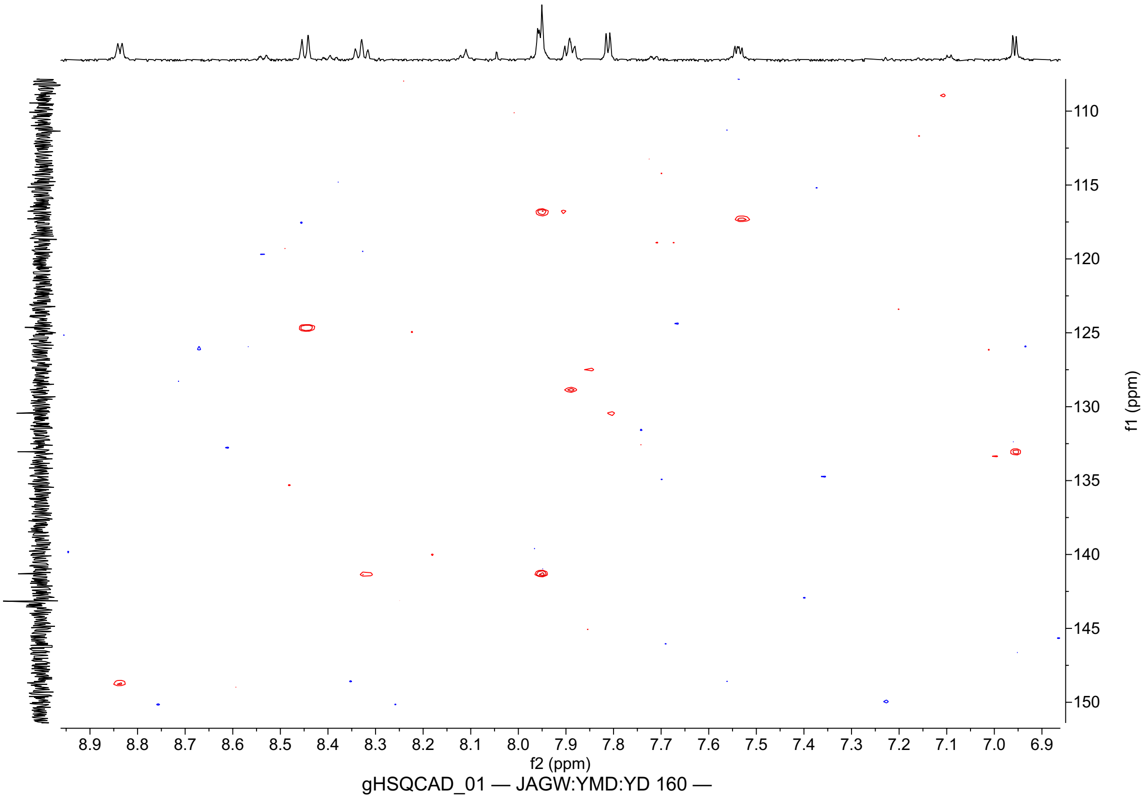


***Figure S4.31*** *HSQC NMR spectrum of* $PtL^6Cl$ *in* $d_6$*-DMSO*

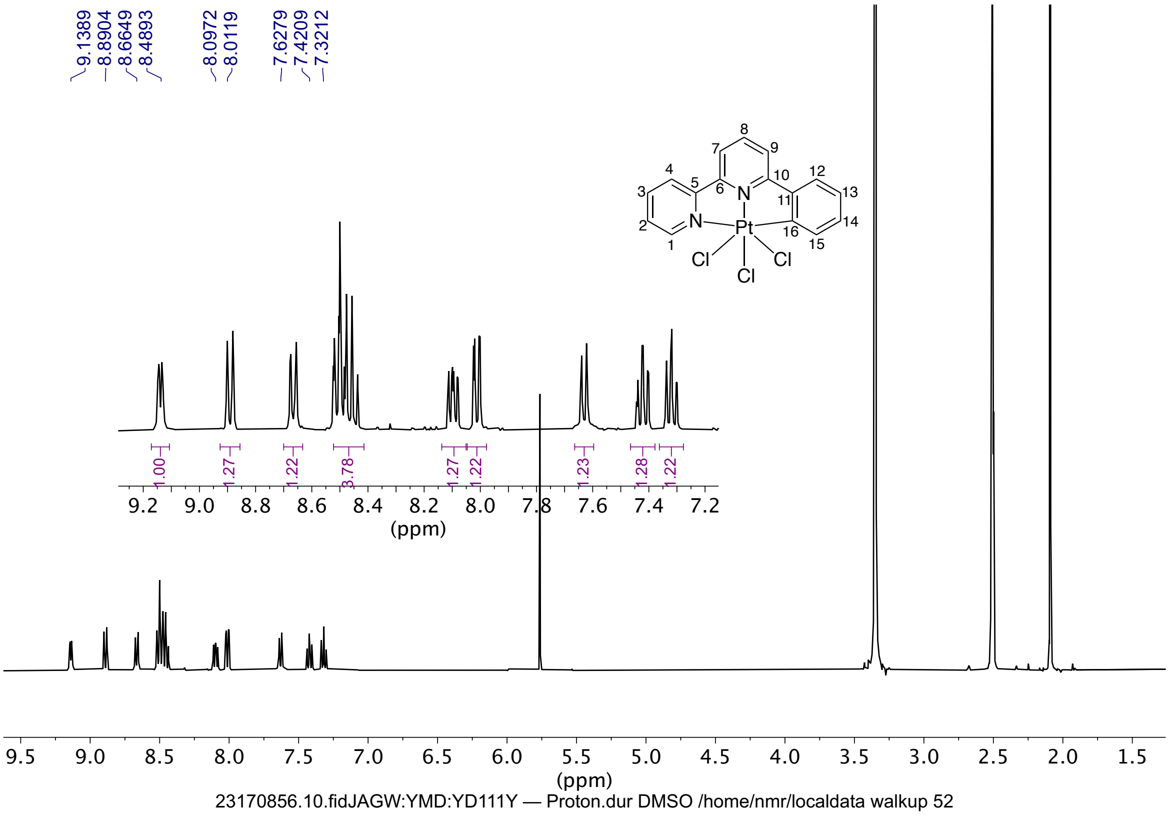


***Figure S4.32*** *$^{1}$H NMR spectrum of* $PtL^1Cl_3$ *in* $d_6$*-DMSO*

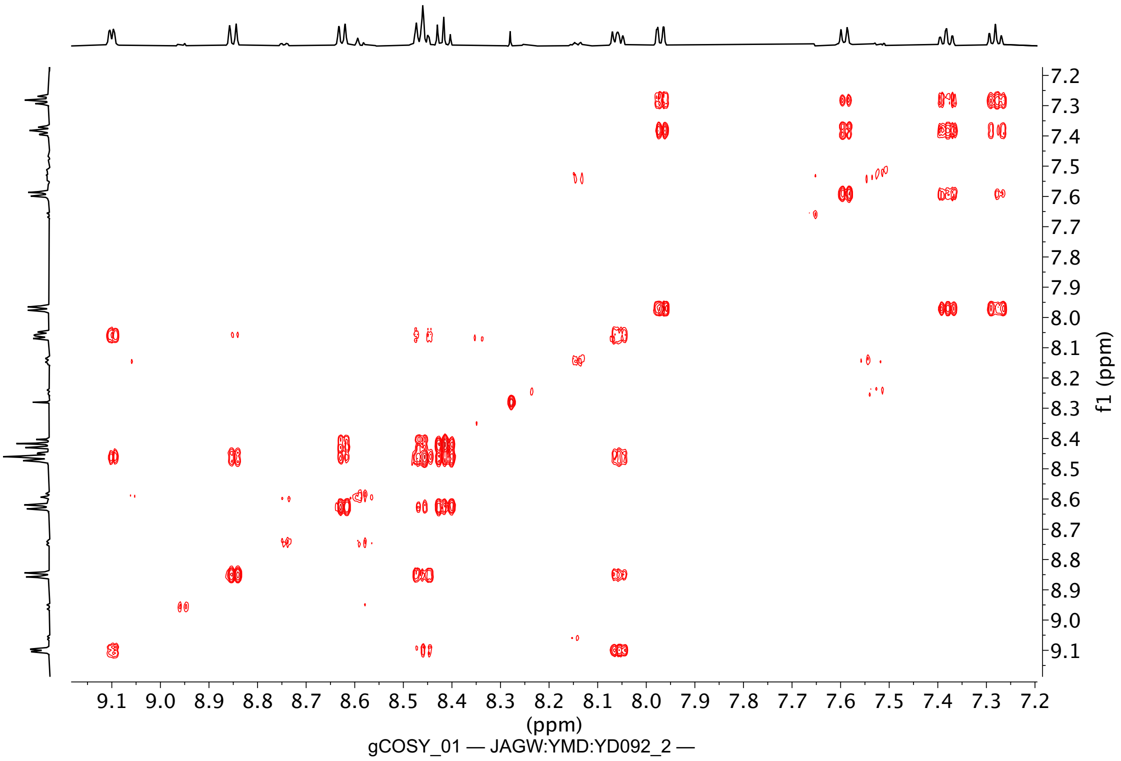


***Figure S4.33*** *COSY NMR spectrum of PtL[1]Cl$_3$ in d$_6$-DMSO*

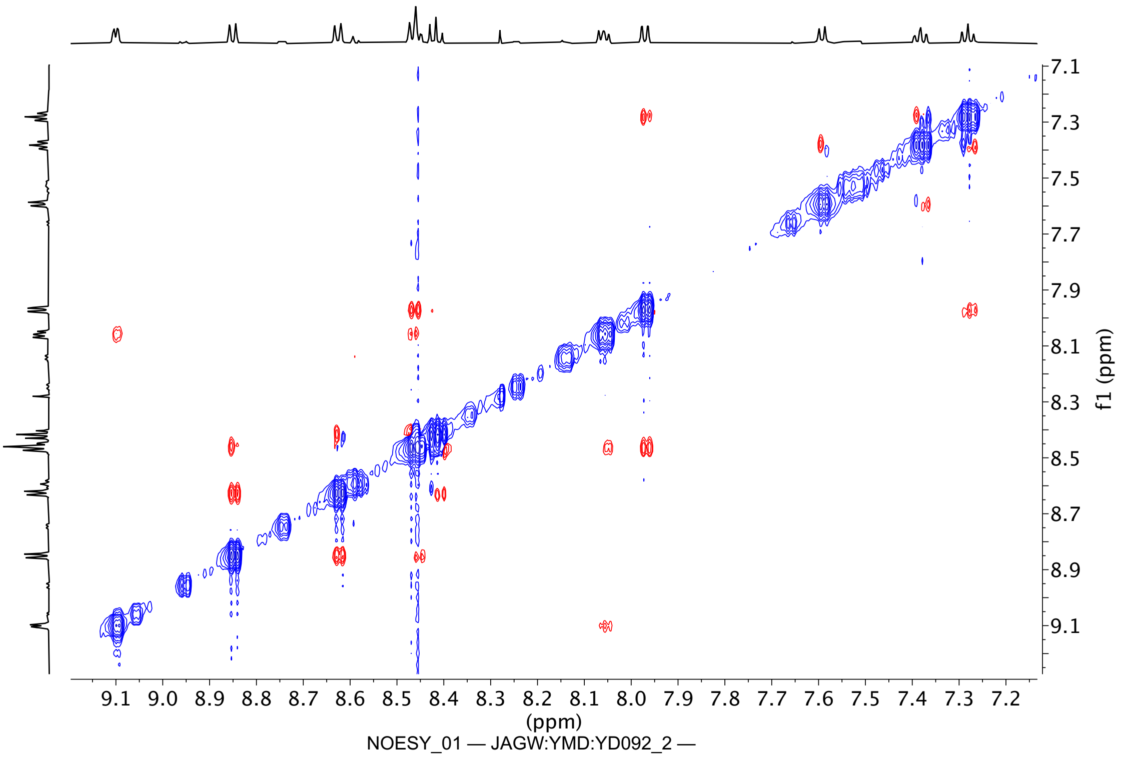


***Figure S4.34*** *NOESY NMR spectrum of PtL[1]Cl$_3$ in d$_6$-DMSO*

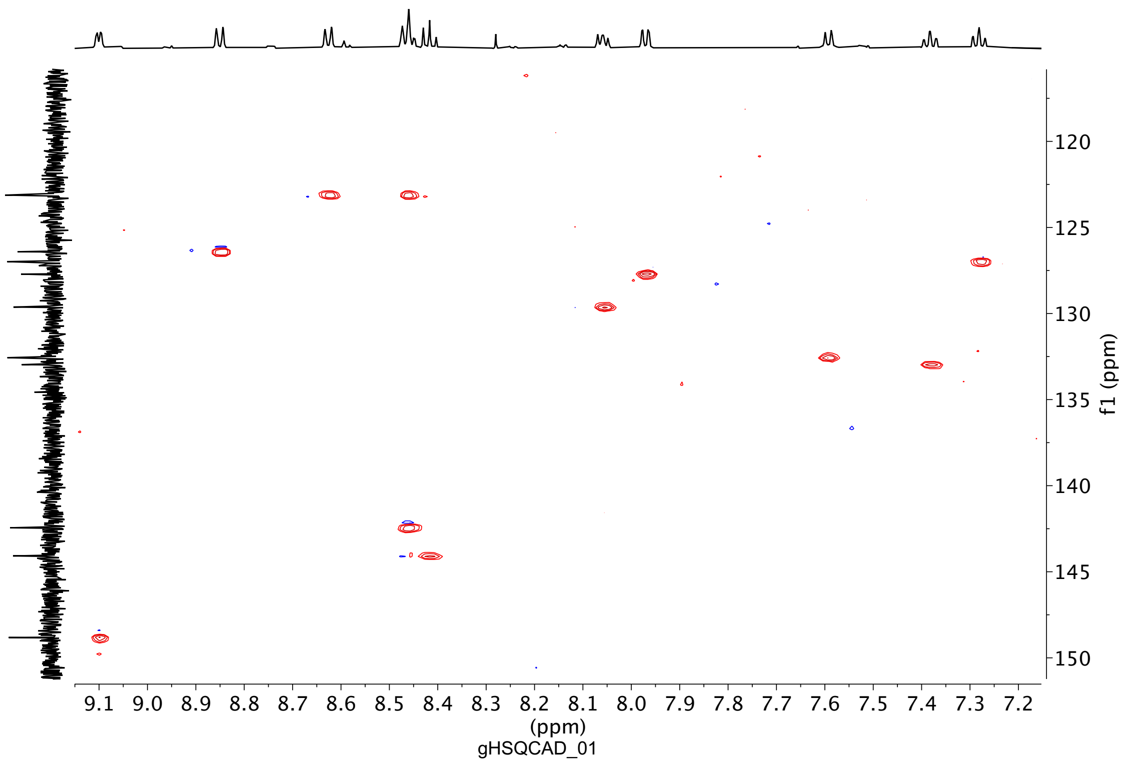


***Figure S4.35*** *HSQC NMR spectrum of $PtL^1Cl_3$ in $d_6$-DMSO*

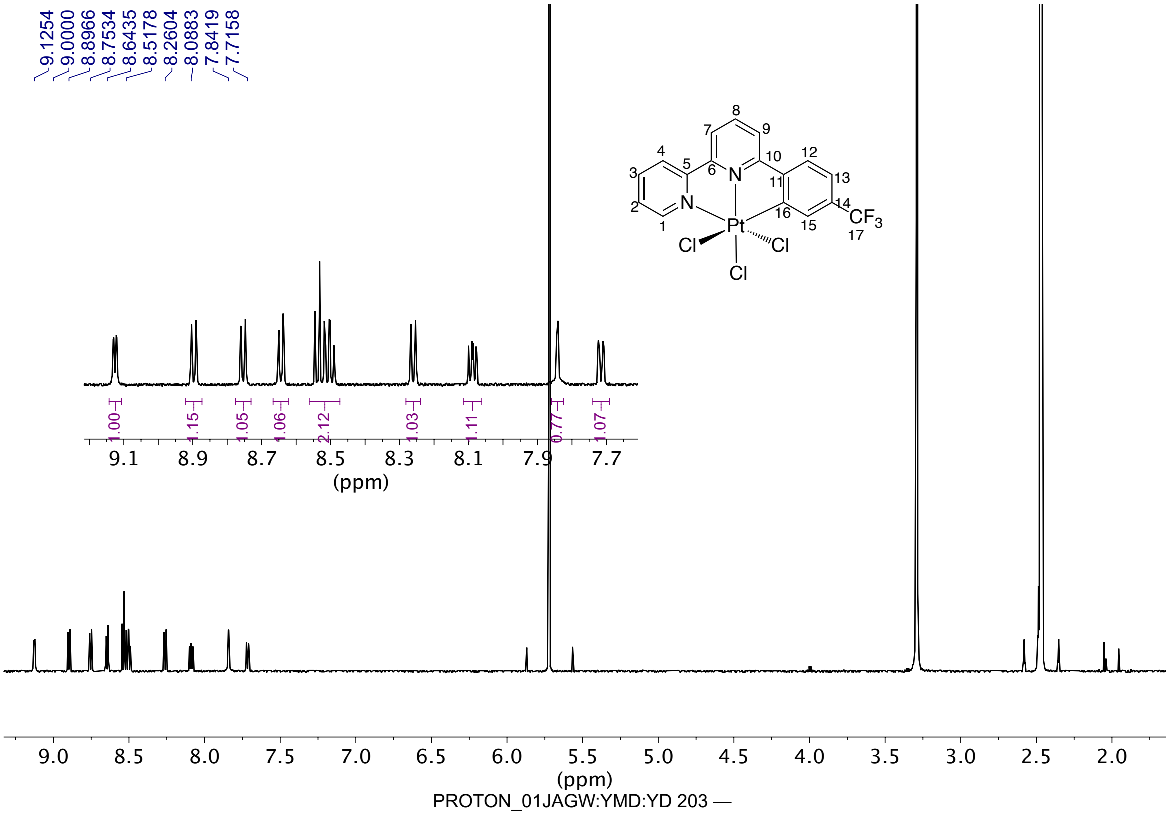


***Figure S4.36*** *$^1H$ NMR spectrum of $PtL^2Cl_3$ in $d_6$-DMSO*

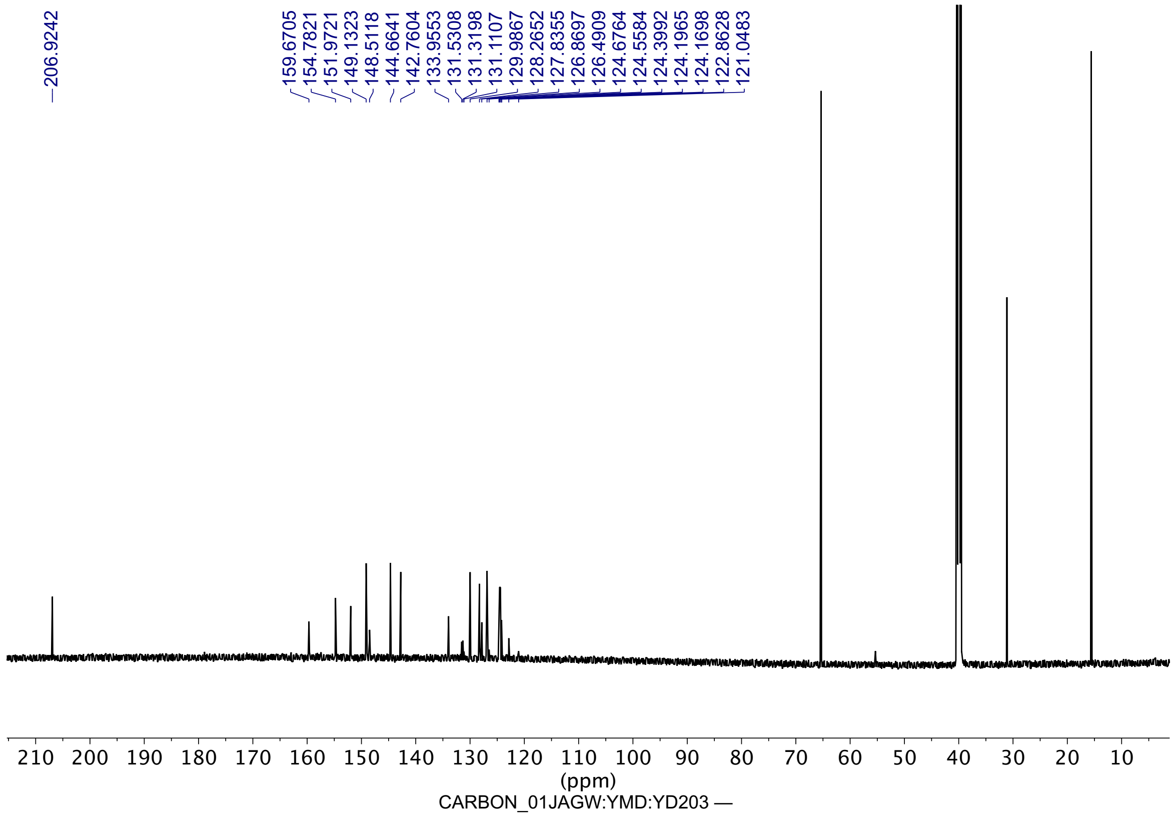


***Figure S4.37*** *$^{13}$C NMR spectrum of $PtL^2Cl_3$ in $d_6$-DMSO*

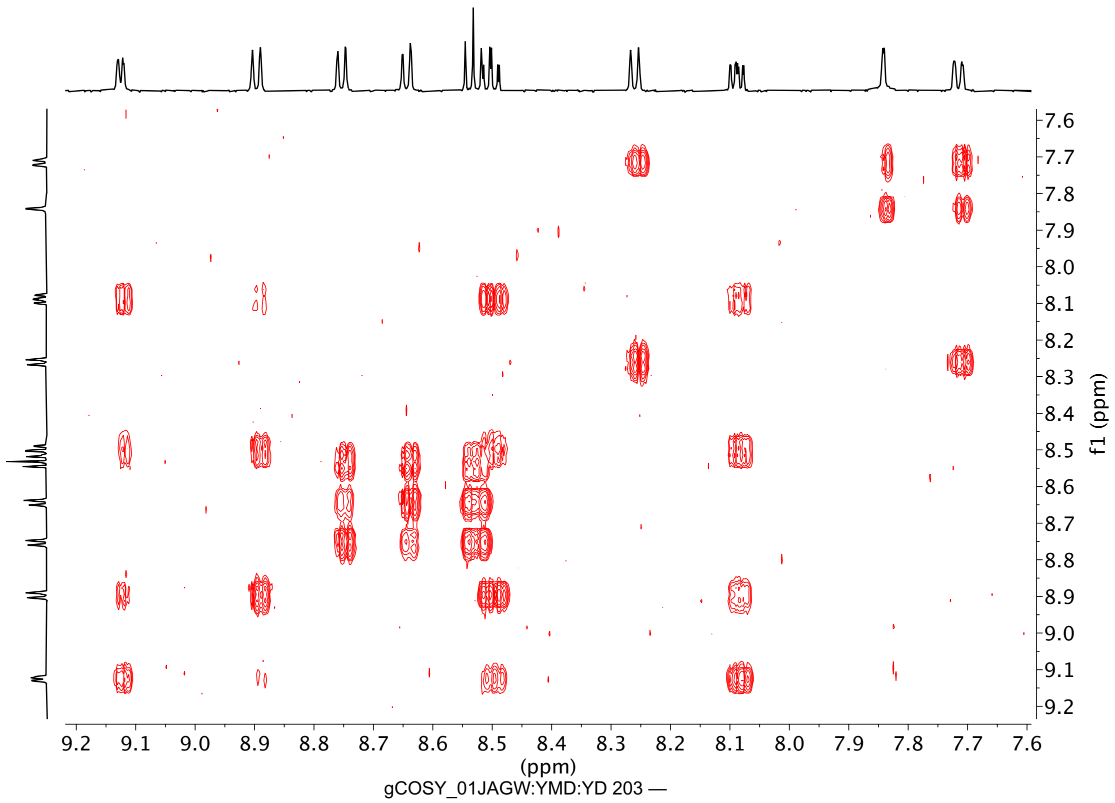


***Figure S4.38*** *COSY NMR spectrum of $PtL^2Cl_3$ in $d_6$-DMSO*

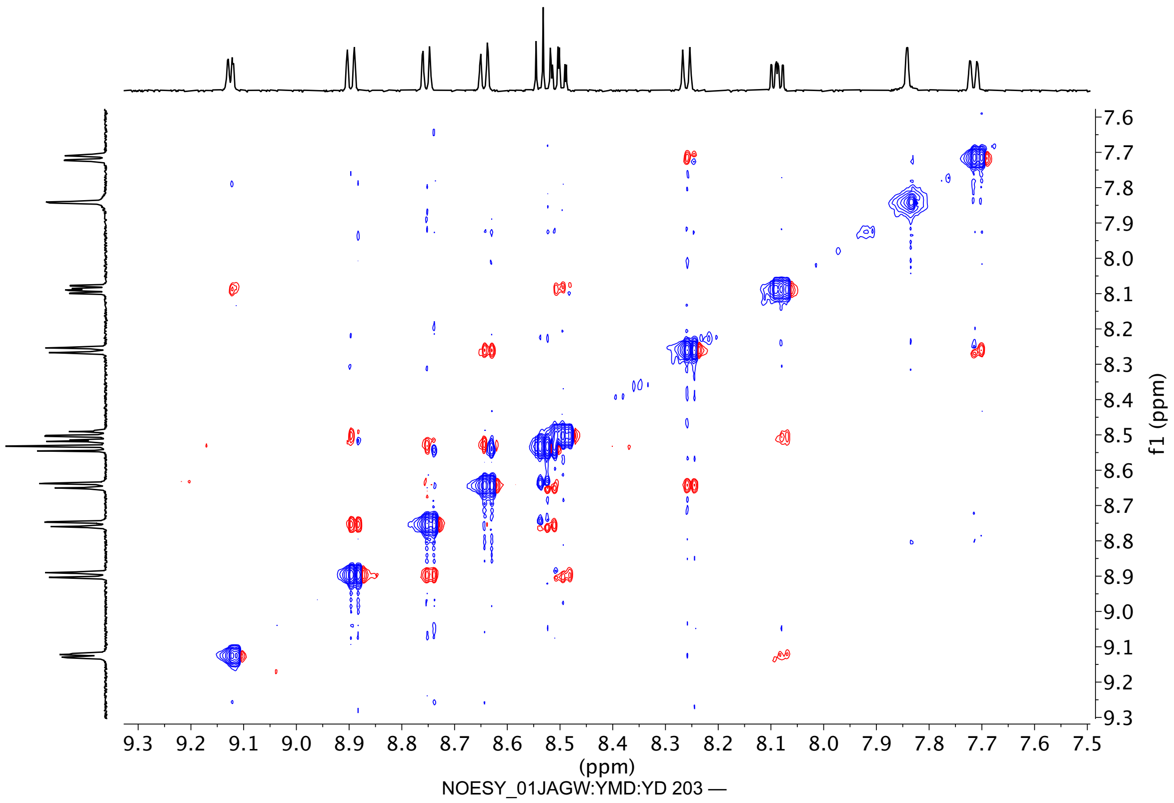


***Figure S4.39*** *NOESY NMR spectrum of $PtL^2Cl_3$ in $d_6$-DMSO*

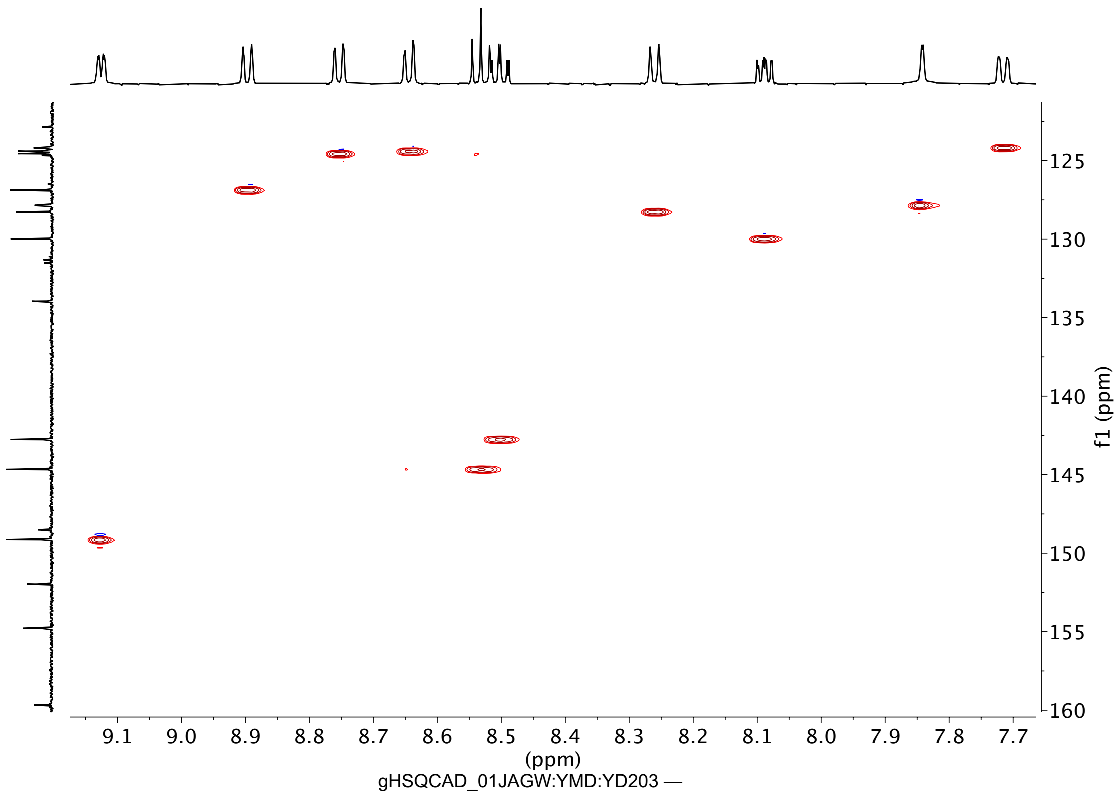


***Figure S4.40*** *HSQC NMR spectrum of $PtL^2Cl_3$ in $d_6$-DMSO*

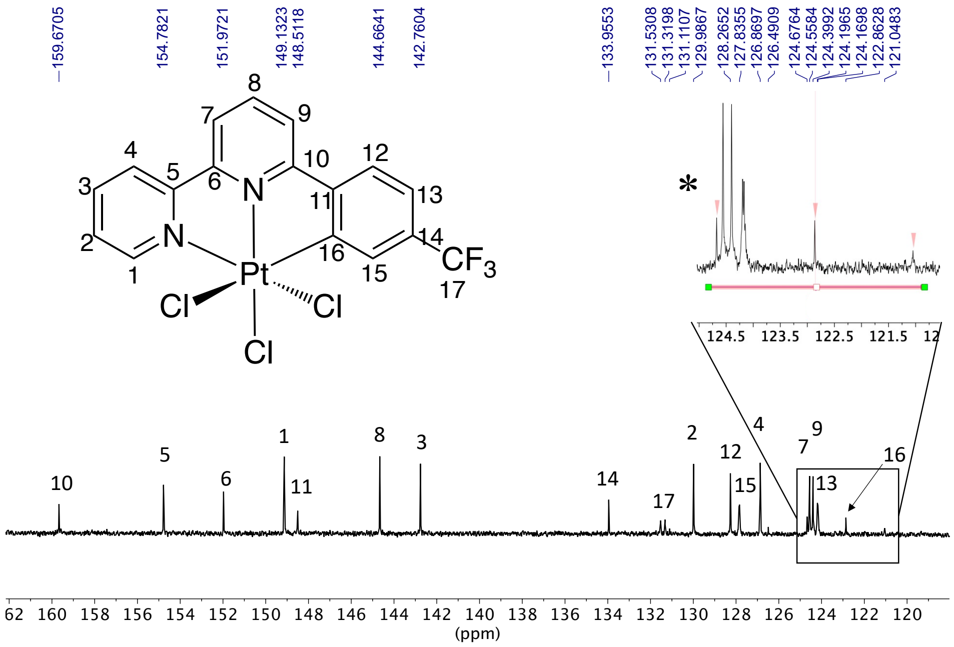


***Figure S4.41*** *$^{13}C$ spectrum of $PtL^2Cl_3$ with assigned C signals labelled with numbers. The expanded region (*) shows the peak corresponding to $C^{16}$ and satellites due to coupling to $^{195}Pt$ ($^1J$ = 550 Hz).*

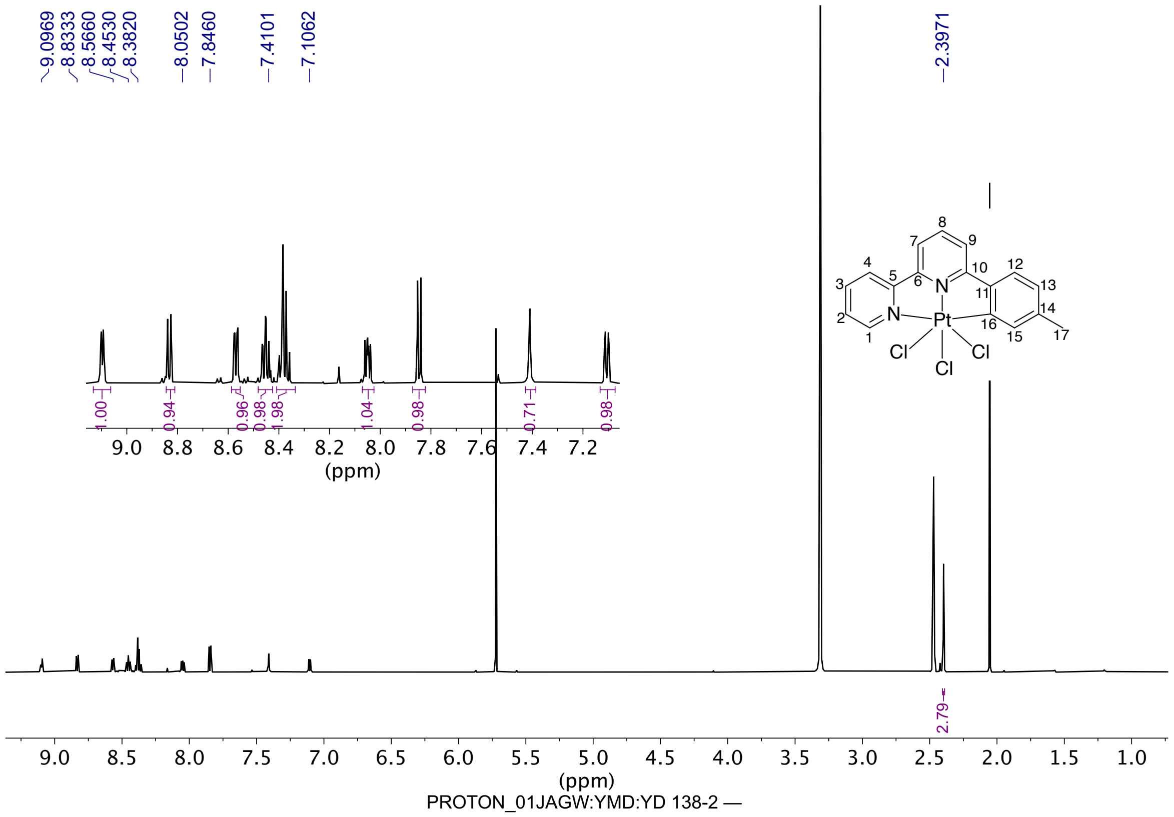


***Figure S4.42*** *$^{1}$H NMR spectrum of PtL$^{3}$Cl$_{3}$ in d$_{6}$-DMSO*

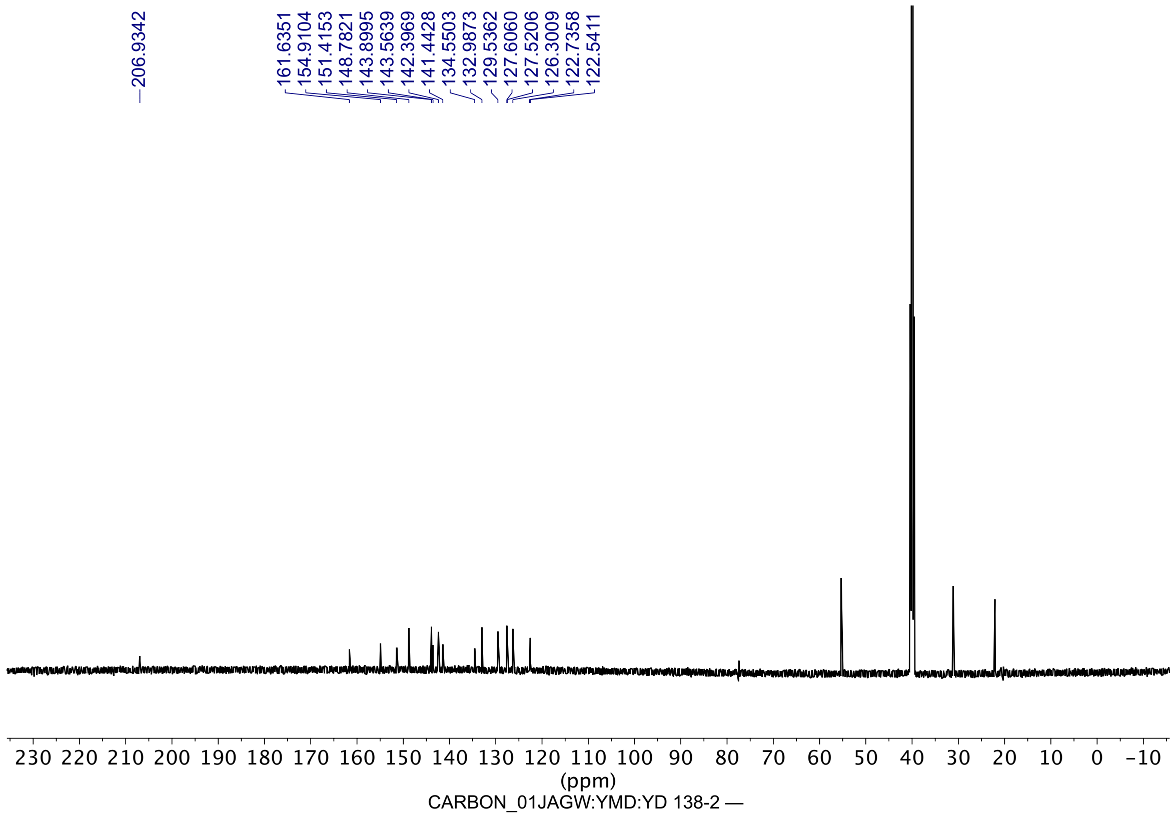


***Figure S4.43*** *$^{13}$C NMR spectrum of PtL$^{3}$Cl$_{3}$ in d$_{6}$-DMSO*

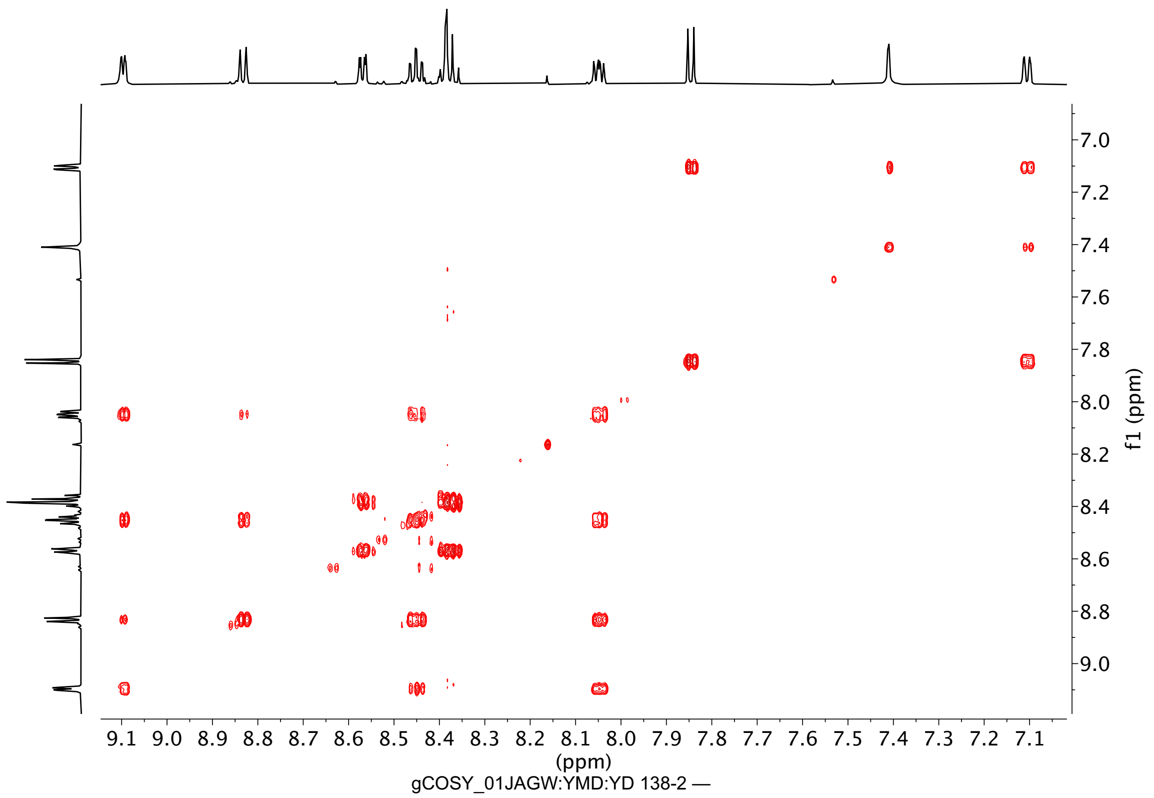


***Figure S4.44*** *COSY NMR spectrum of $PtL^3Cl_3$ in $d_6$-DMSO*

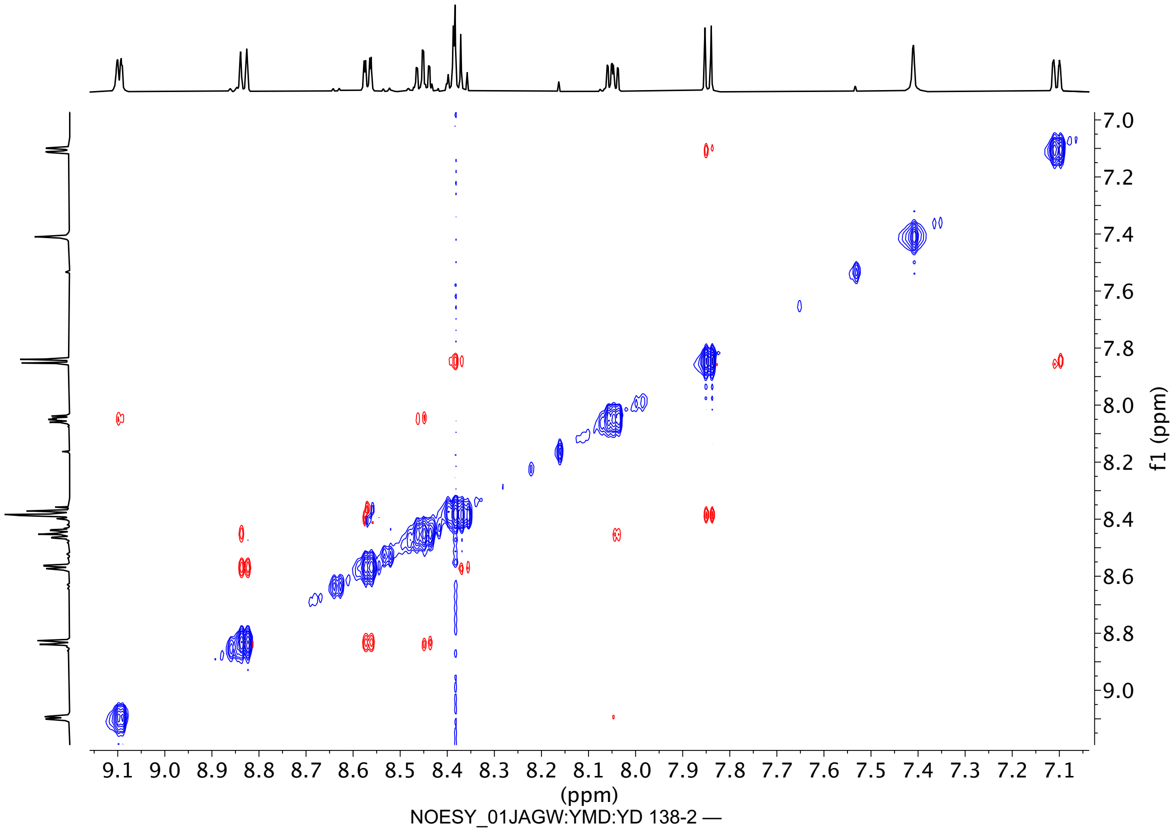


***Figure S4.45*** *NOESY NMR spectrum of $PtL^3Cl_3$ in $d_6$-DMSO*

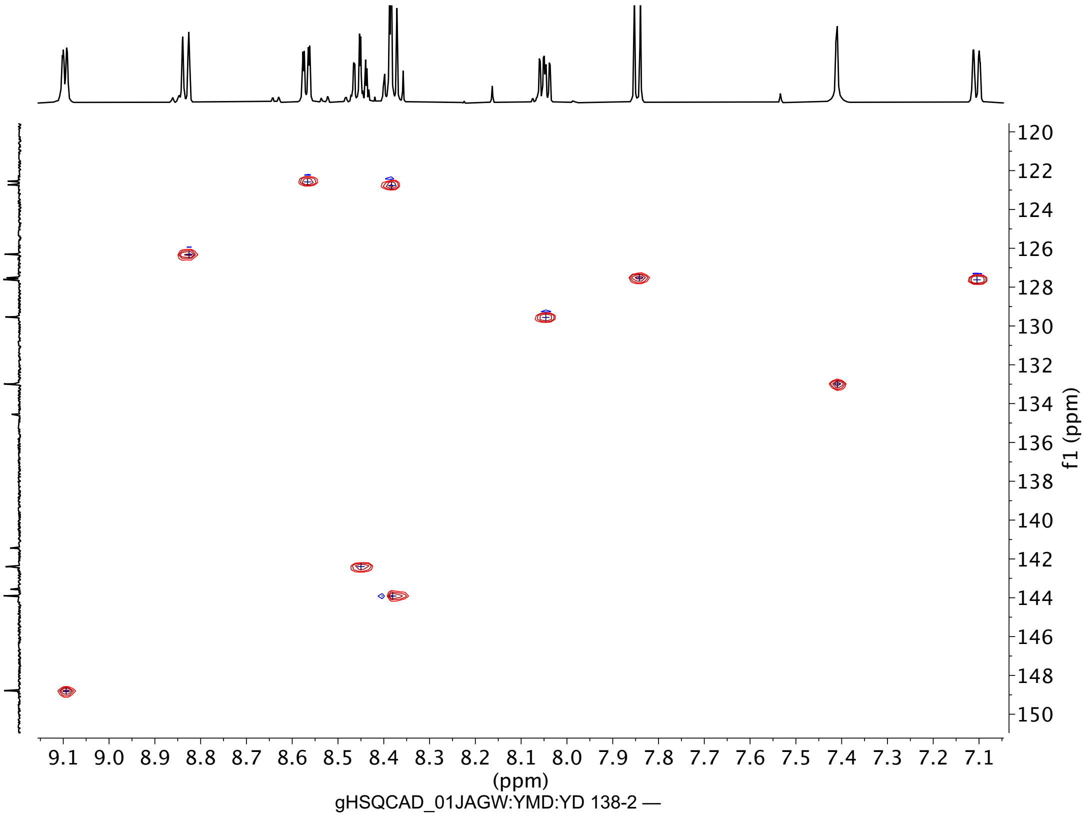


***Figure S4.46*** *HSQC NMR spectrum of $PtL^3Cl_3$ in $d_6$-DMSO*

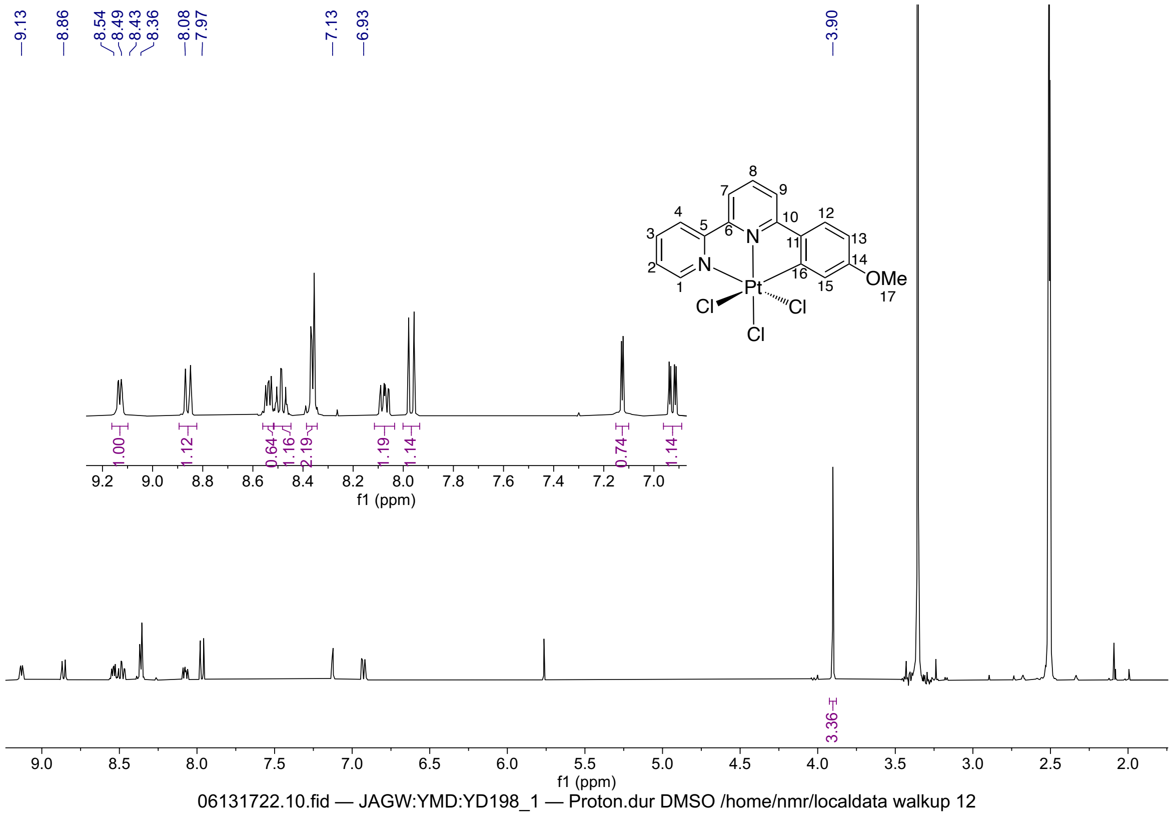


***Figure S4.47*** *$^1H$ NMR spectrum of $PtL^4Cl_3$ in $d_6$-DMSO*

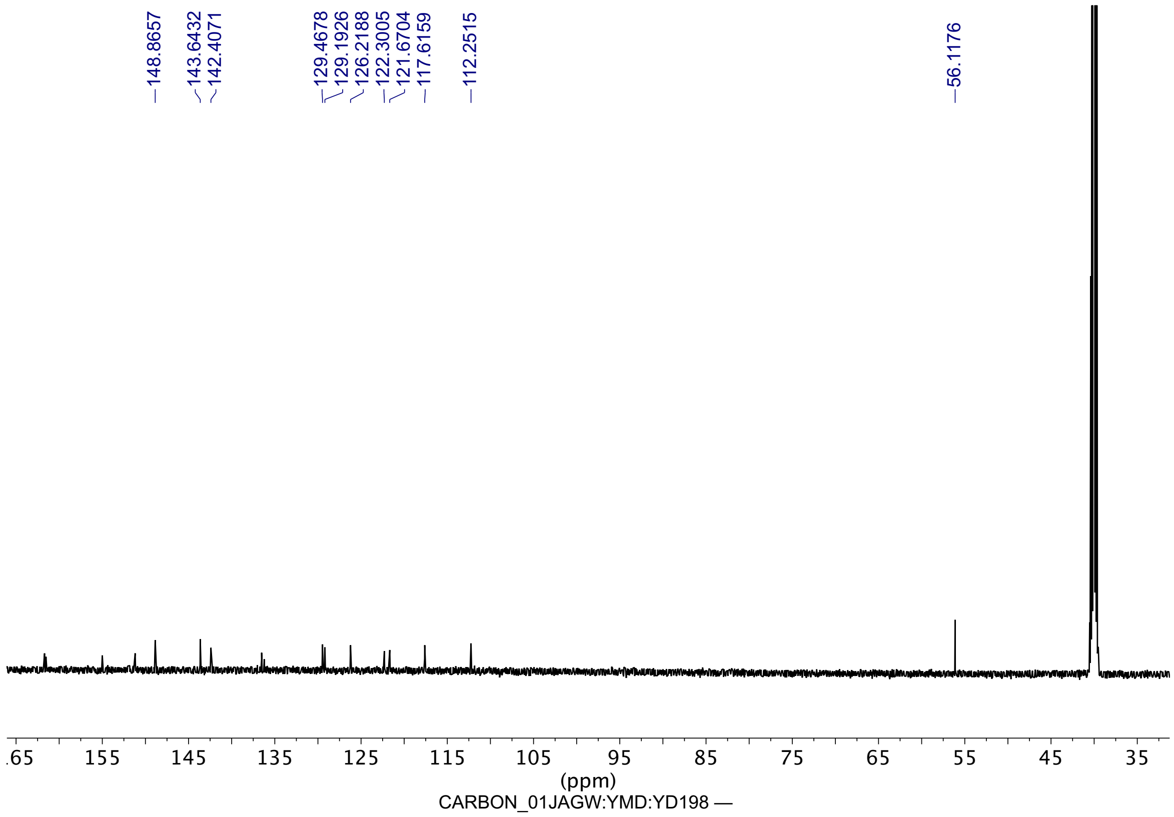


***Figure S4.48*** *$^{13}C$ NMR spectrum of $PtL^4Cl_3$ in $d_6$-DMSO*

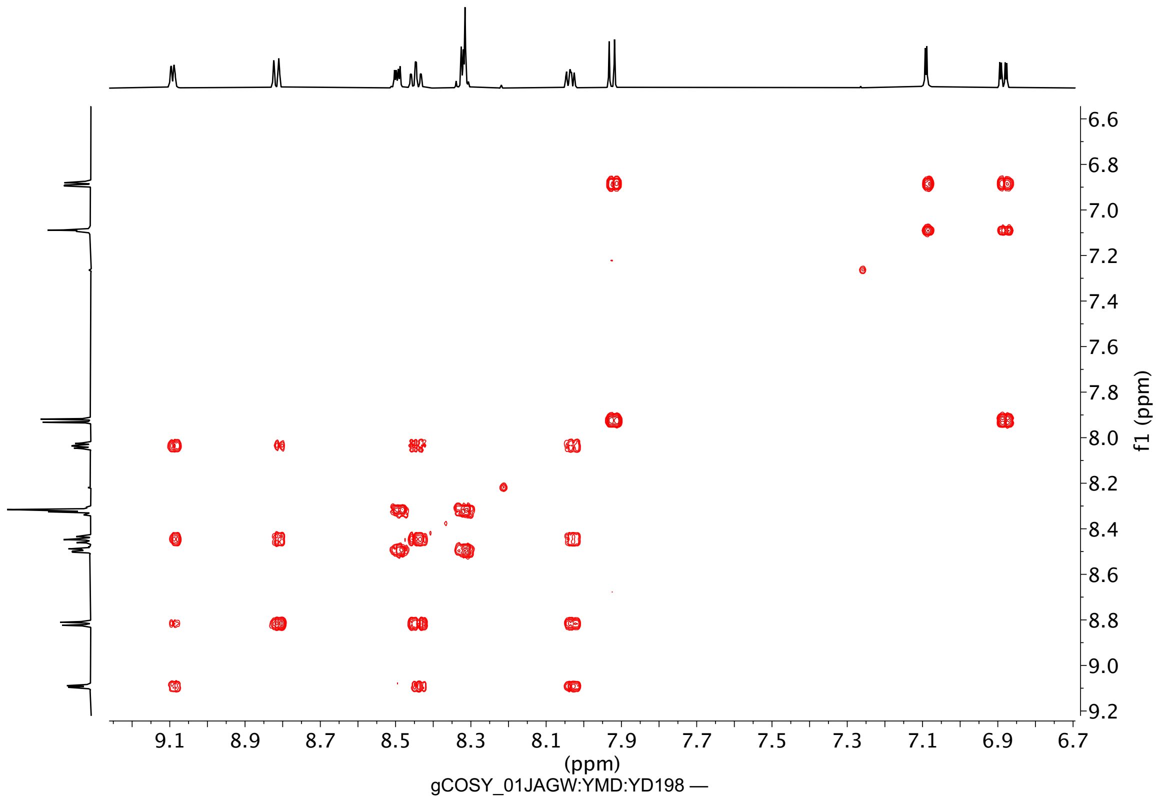


***Figure S4.49*** *COSY NMR spectrum of $PtL^4Cl_3$ in $d_6$-DMSO*

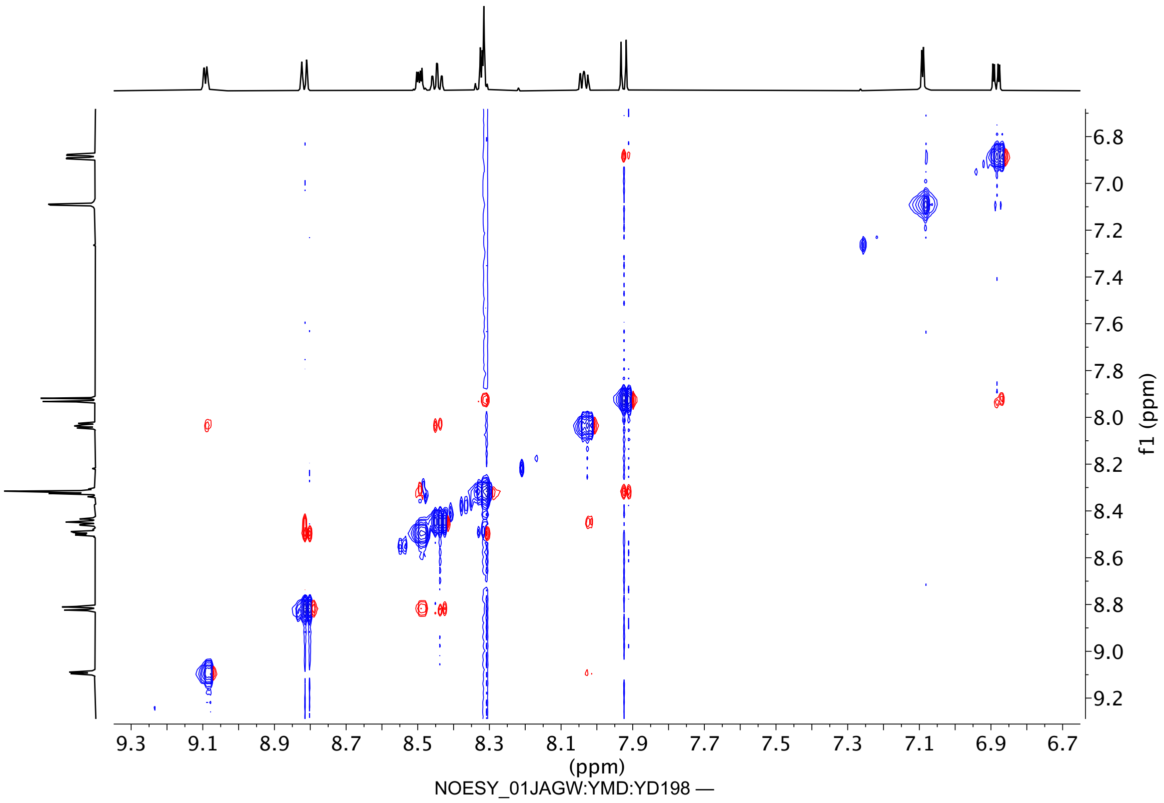


***Figure S4.50** NOESY NMR spectrum of $PtL^4Cl_3$ in $d_6$-DMSO*

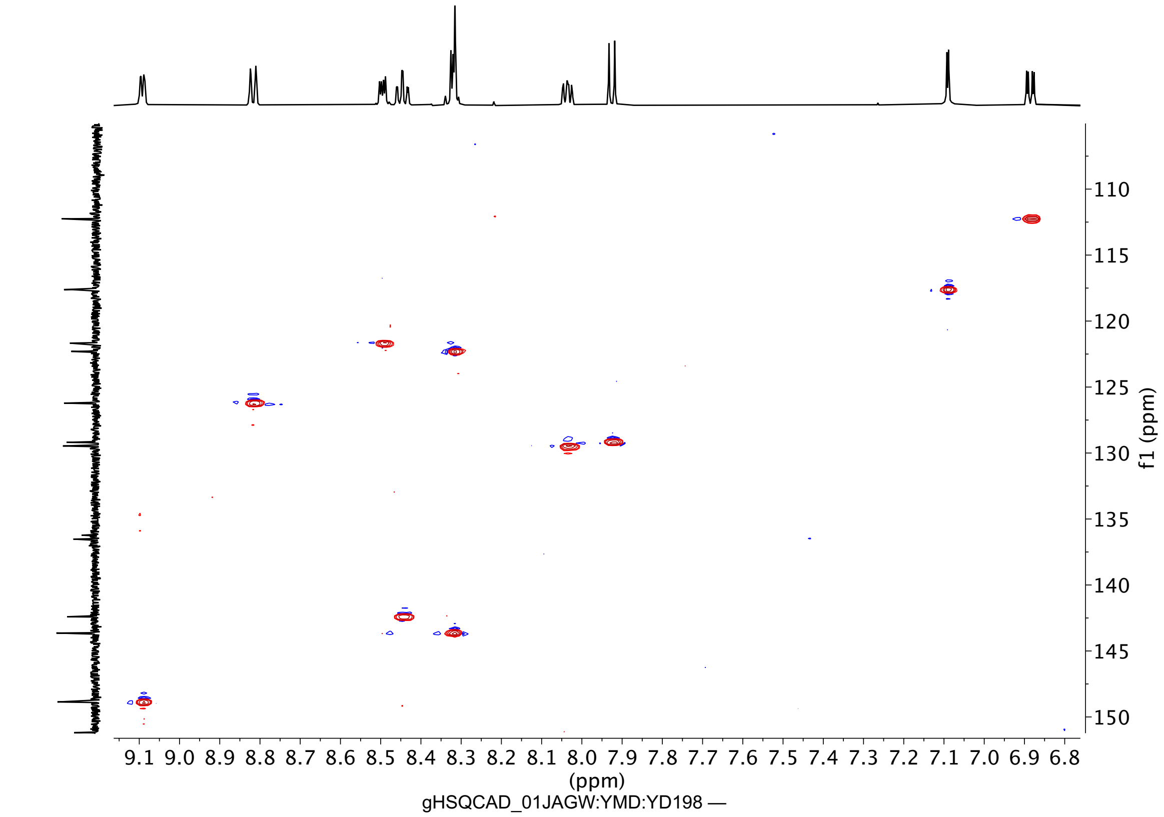


***Figure S4.51** HSQC NMR spectrum of $PtL^4Cl_3$ in $d_6$-DMSO*

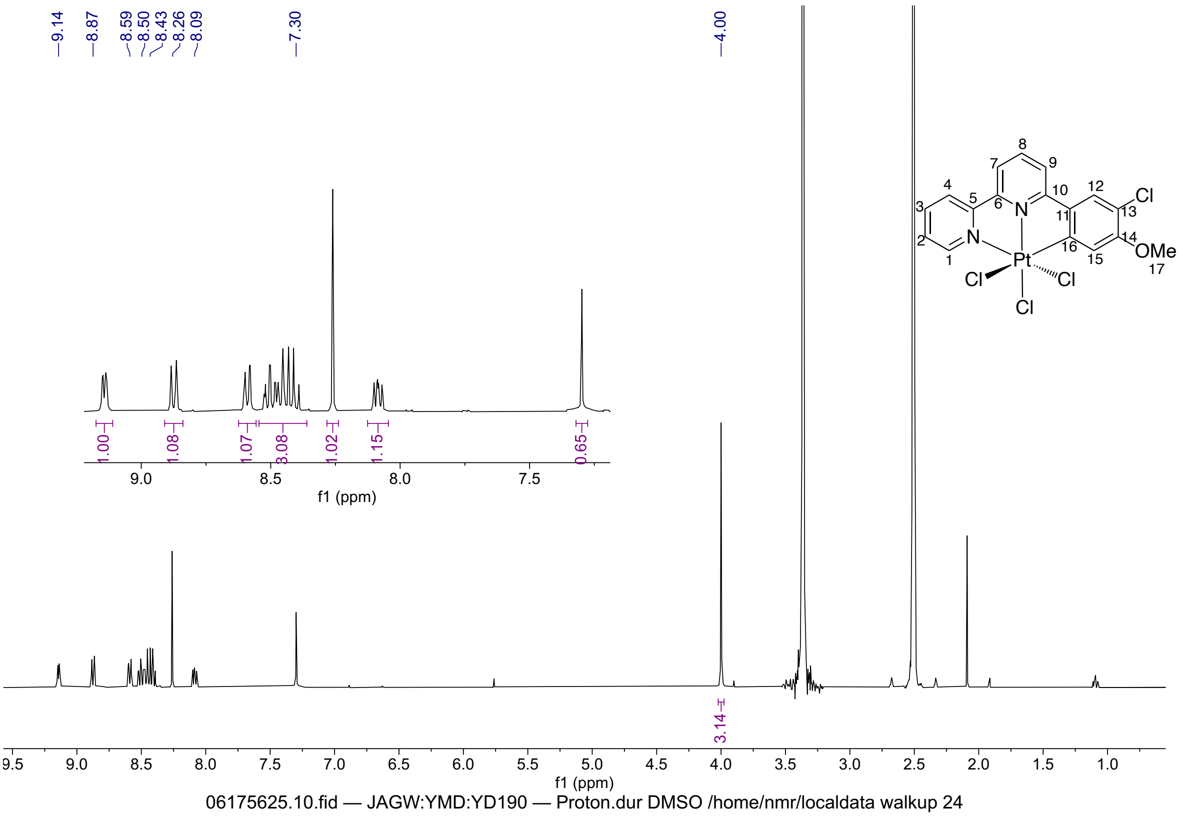


***Figure S4.52*** *$^1$H NMR spectrum of $PtL^{4Cl}Cl_3$ in $d_6$-DMSO*

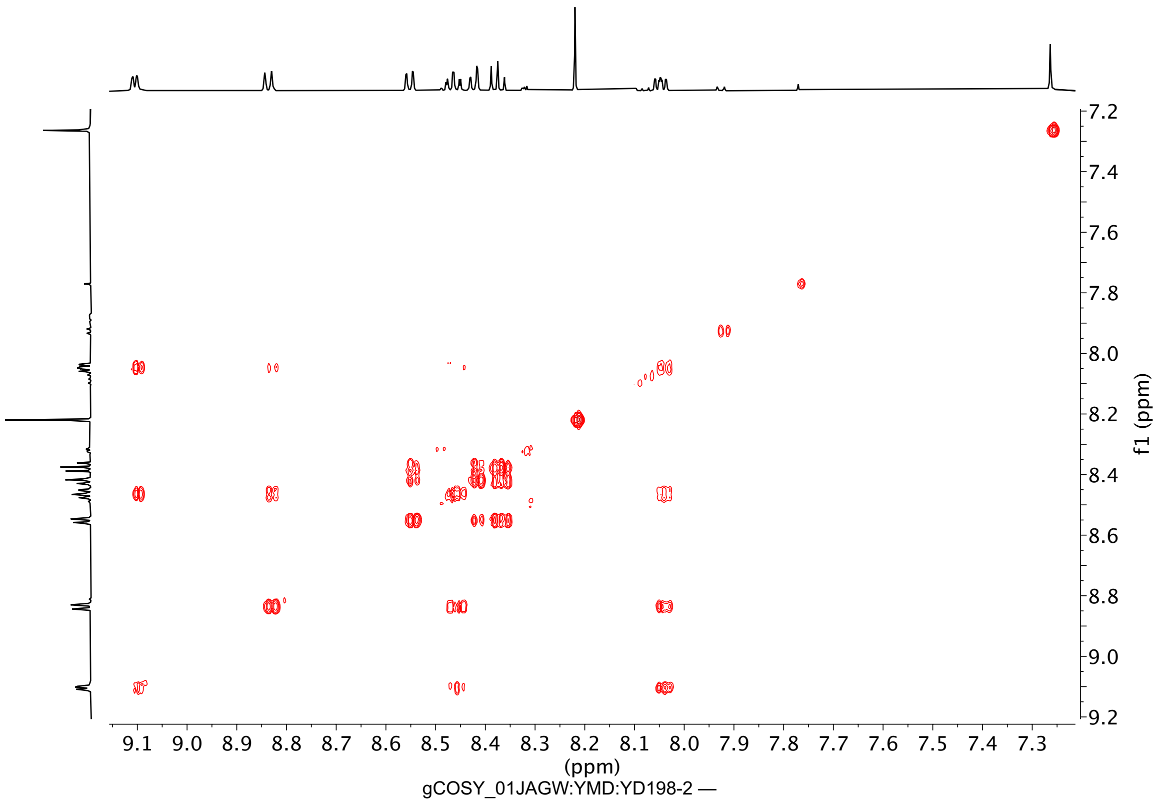


***Figure S4.53*** *COSY NMR spectrum of $PtL^{4Cl}Cl_3$ in $d_6$-DMSO*

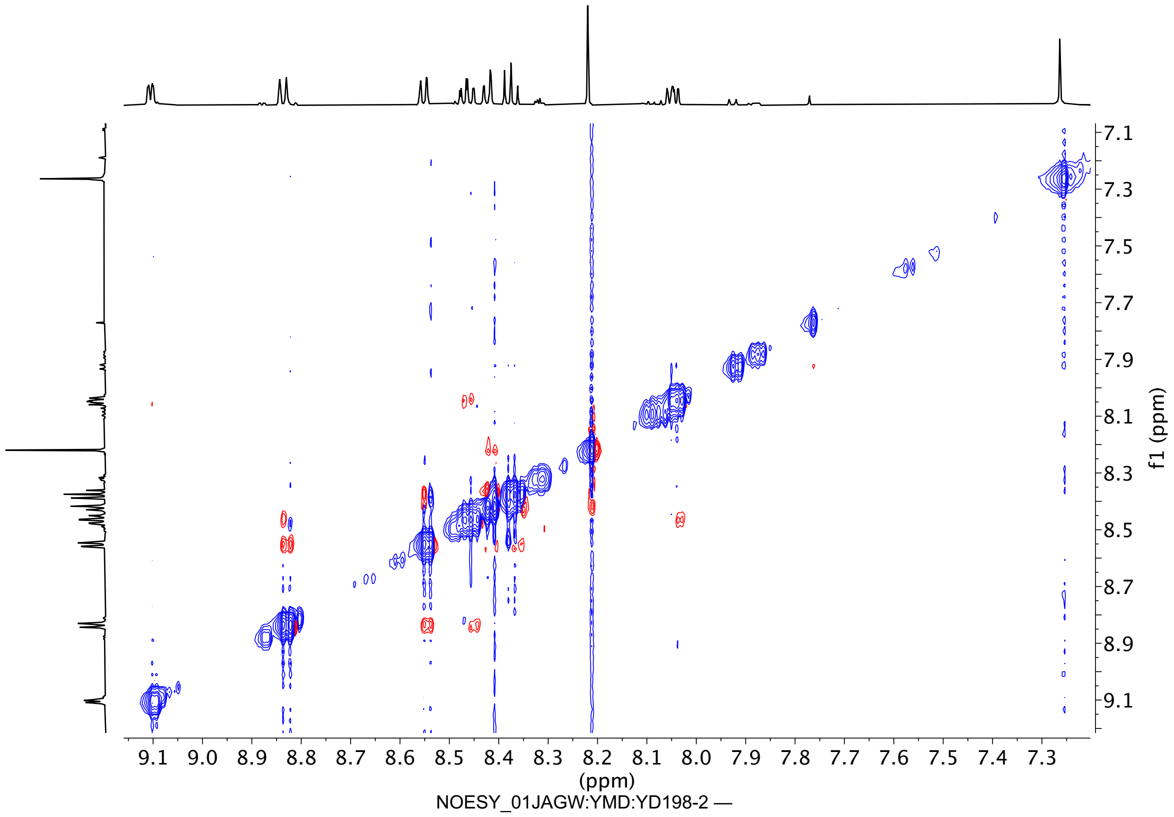


***Figure S4.54*** *NOESY NMR spectrum of PtL*$^{4Cl}$*Cl*$_3$ *in d*$_6$*-DMSO*

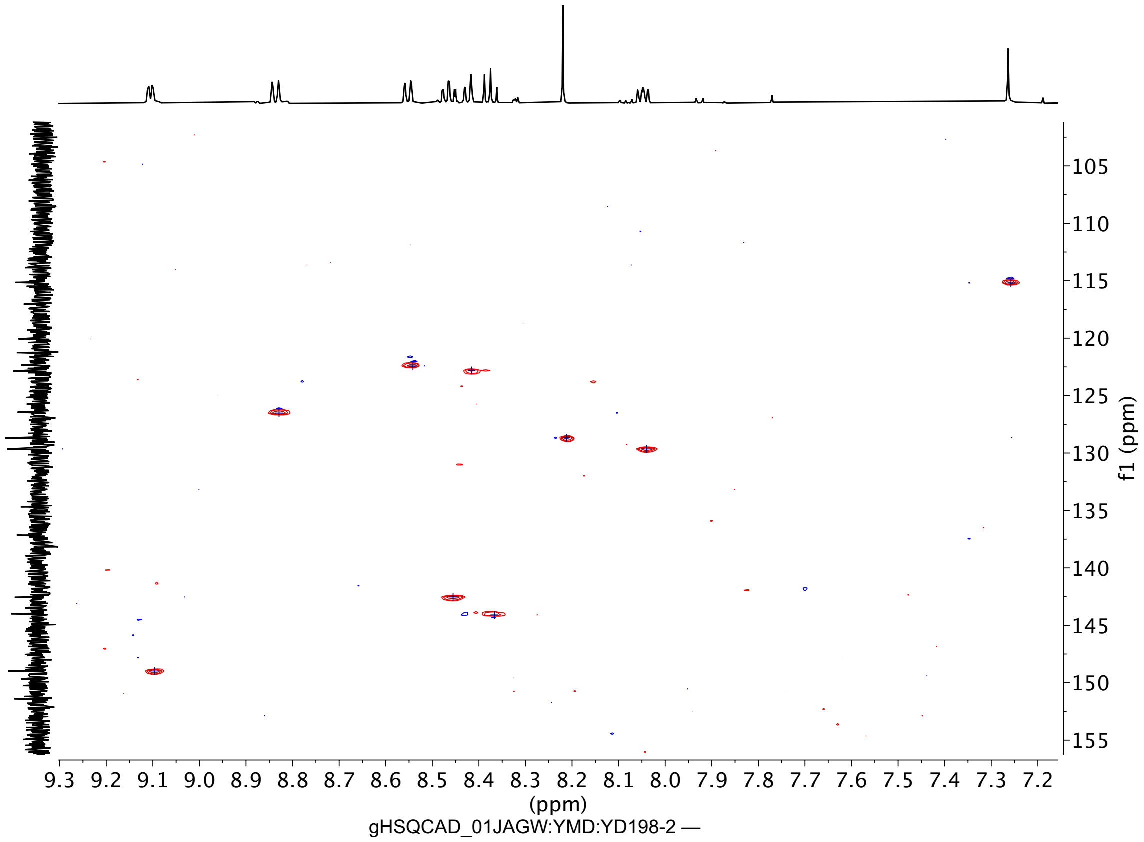


***Figure S4.55*** *HSQC NMR spectrum of PtL*$^{4Cl}$*Cl*$_3$ *in d*$_6$*-DMSO*

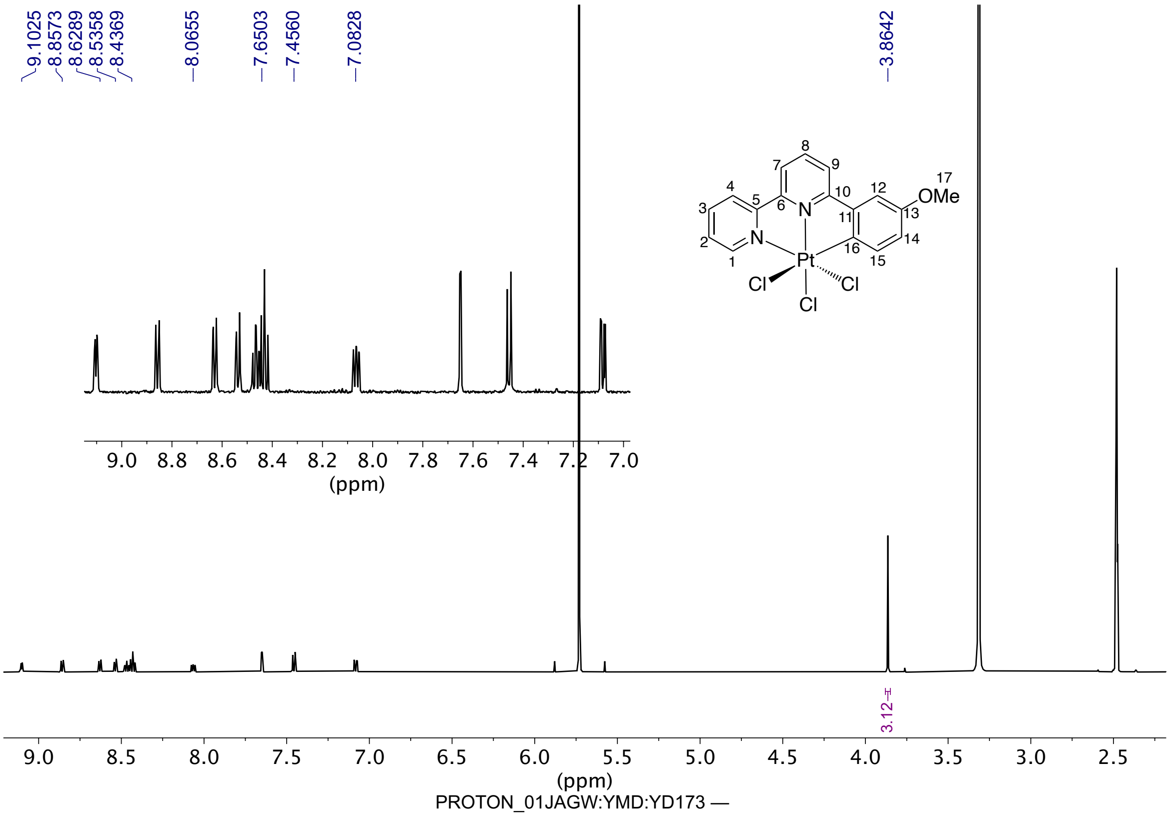


***Figure S4.56*** *$^1$H NMR spectrum of $PtL^5Cl_3$ in $d_6$-DMSO*

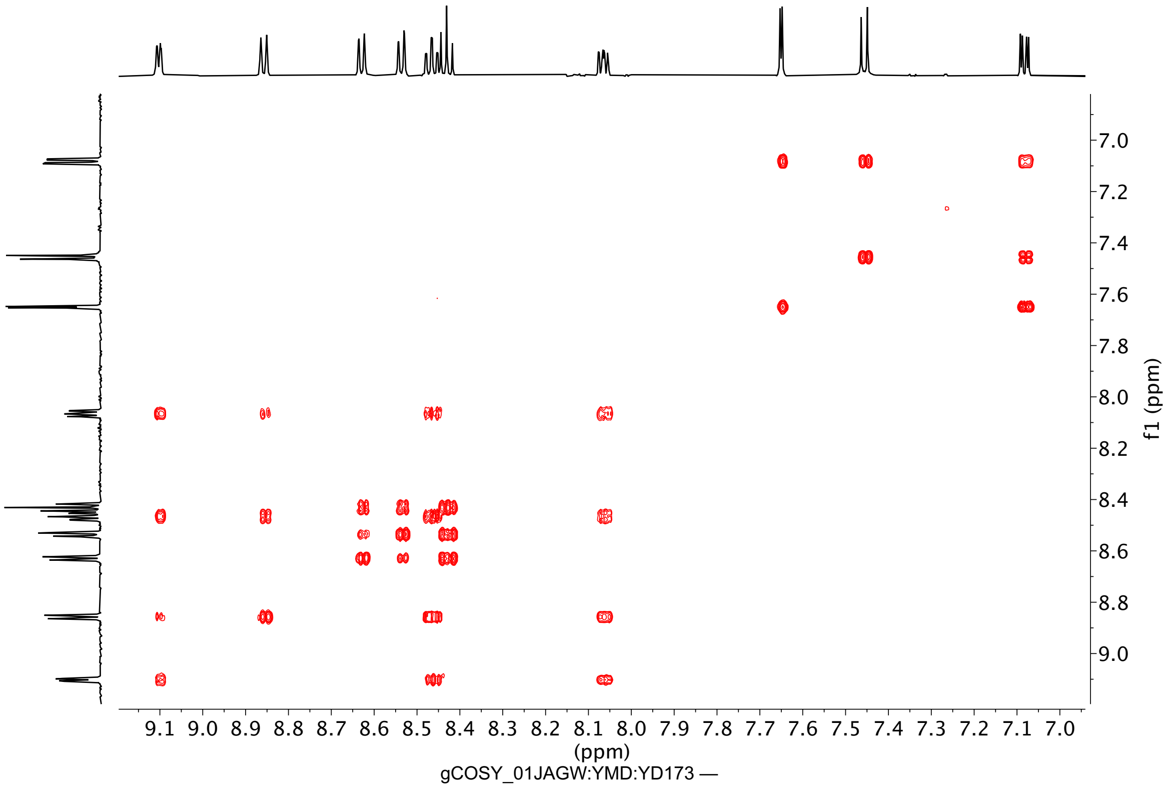


***Figure S4.57*** *COSY NMR spectrum of $PtL^5Cl_3$ in $d_6$-DMSO*

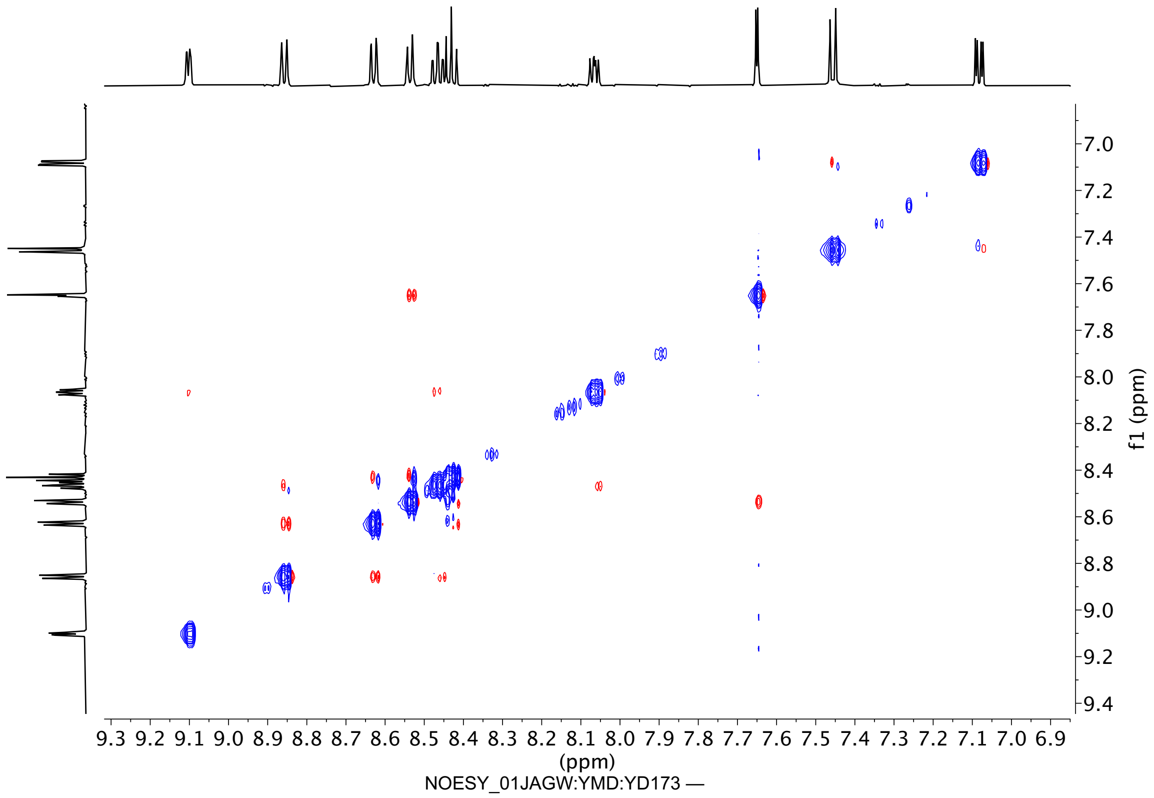


***Figure S4.58*** *NOESY NMR spectrum of $PtL^5Cl_3$ in $d_6$-DMSO*

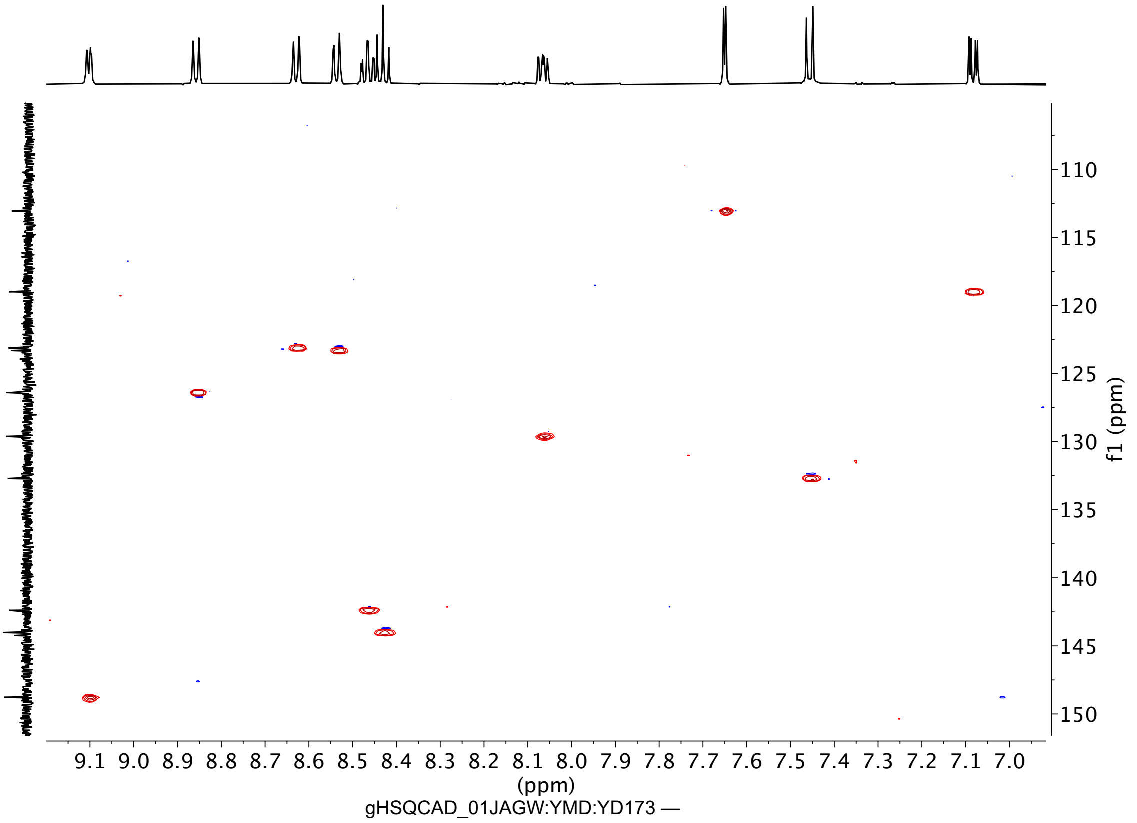


***Figure S4.59*** *HSQC NMR spectrum of $PtL^5Cl_3$ in $d_6$-DMSO*

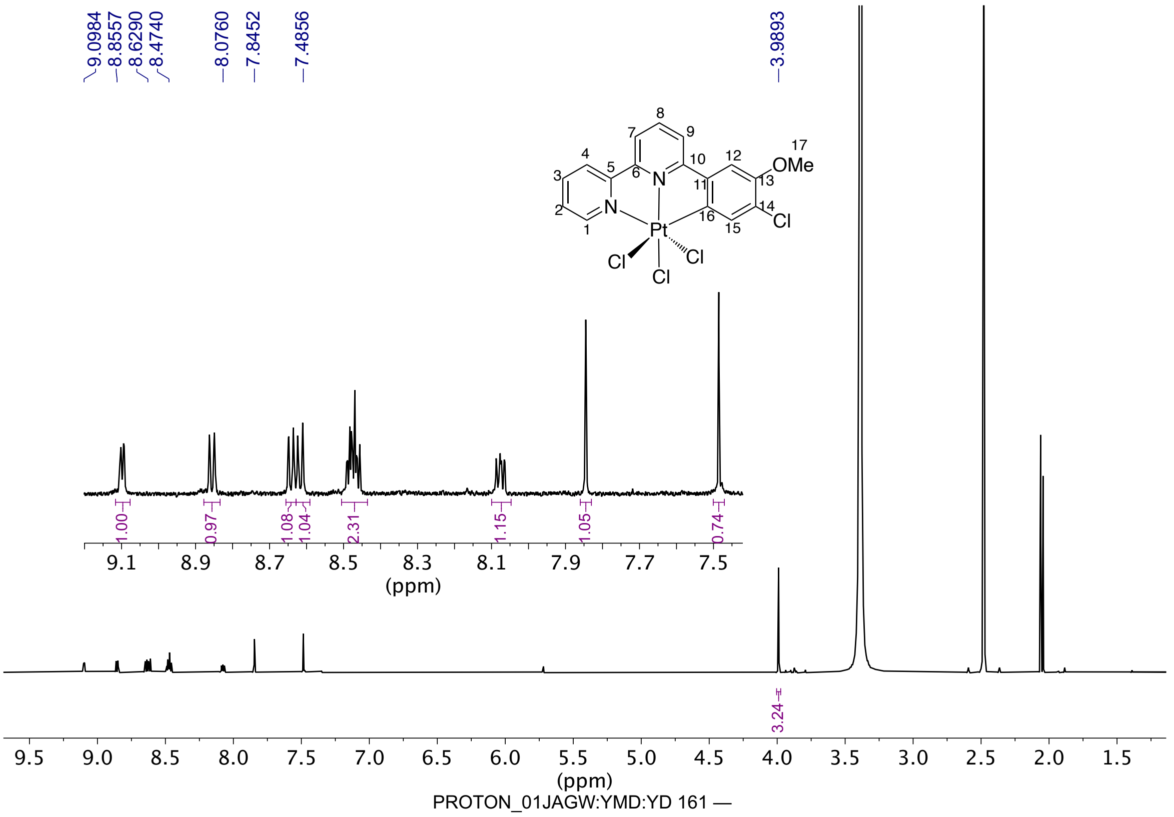


***Figure S4.60*** *$^{1}H$ NMR spectrum of $PtL^{5Cl}Cl_3$ in $d_6$-DMSO*

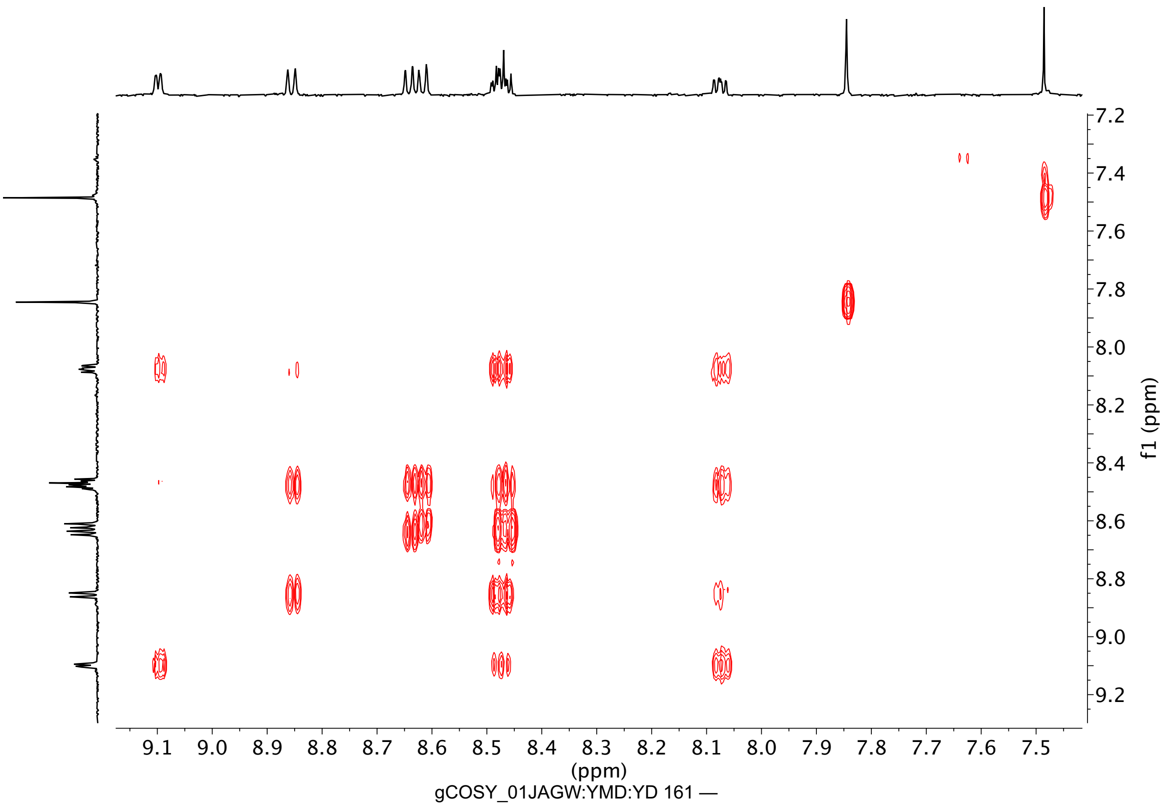


***Figure S4.61*** *COSY NMR spectrum of $PtL^{5Cl}Cl_3$ in $d_6$-DMSO*

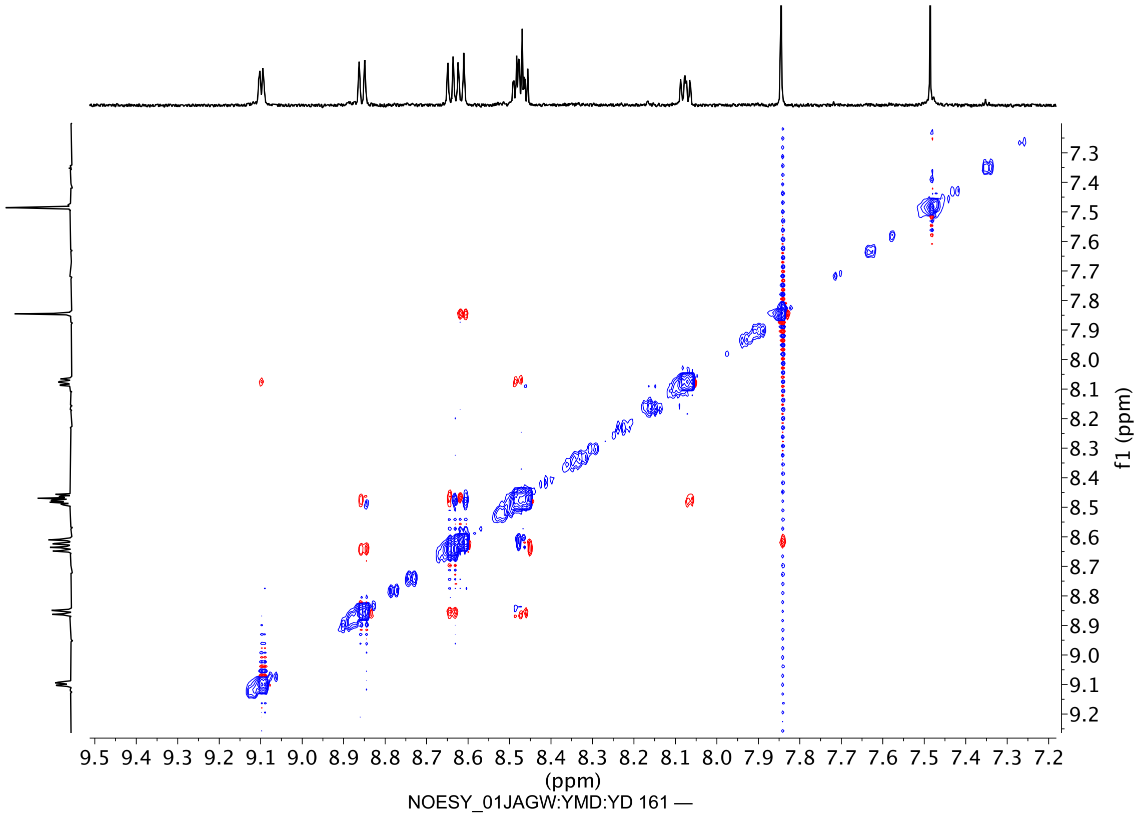


***Figure S4.62*** *NOESY NMR spectrum of $PtL^{5Cl}Cl_3$ in $d_6$-DMSO*

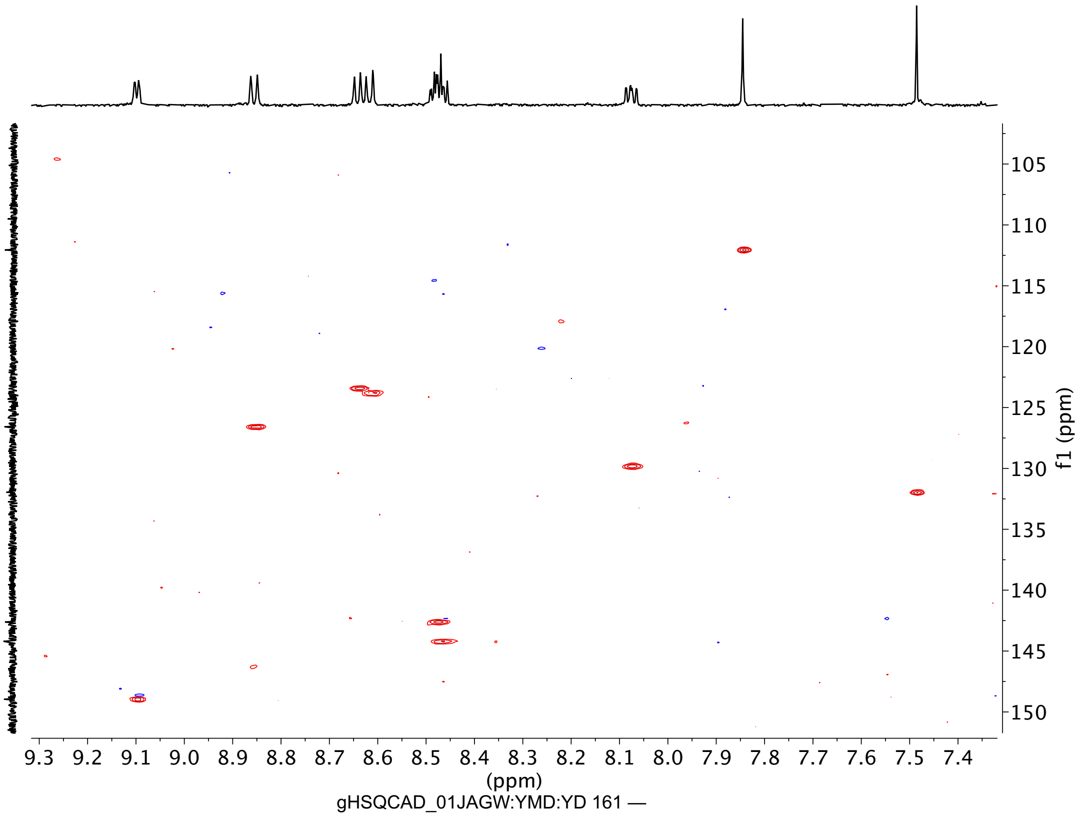


***Figure S4.63*** *HSQC NMR spectrum of $PtL^{5Cl}Cl_3$ in $d_6$-DMSO*

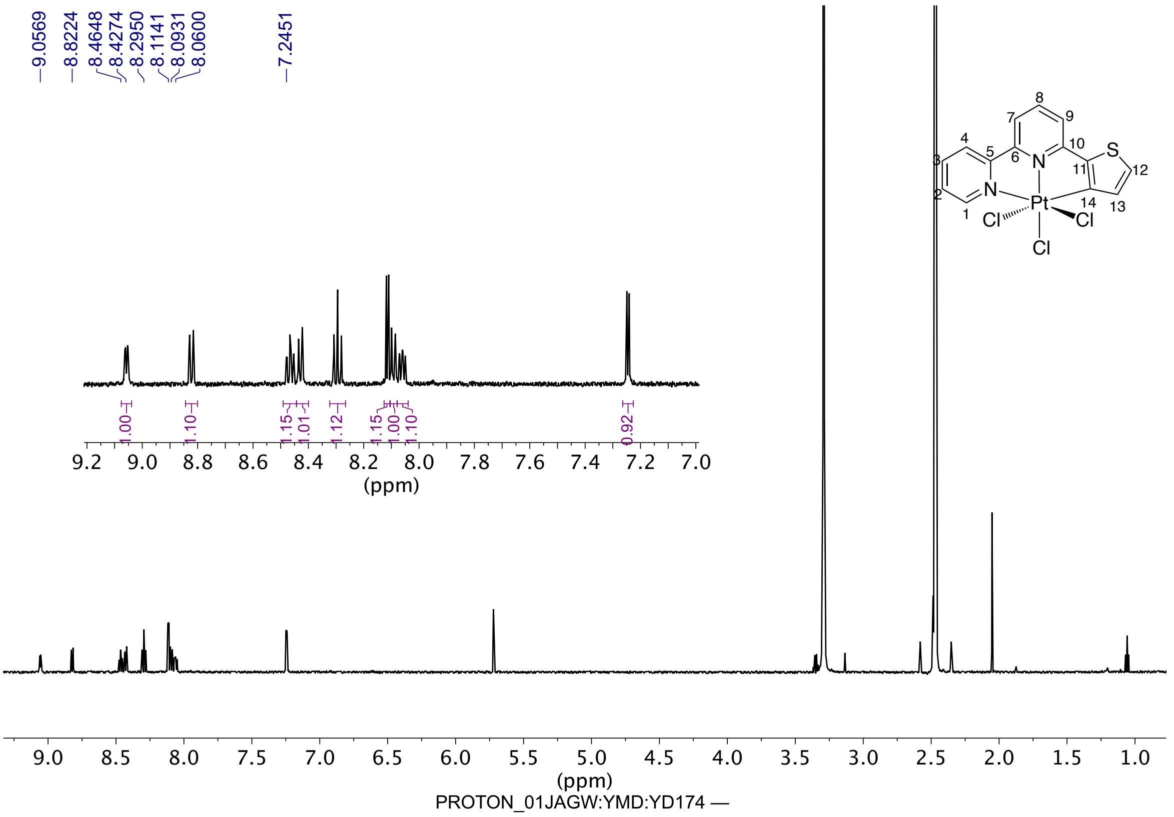


***Figure S4.64*** *$^{1}$H NMR spectrum of PtL[6]Cl$_3$ in d$_6$-DMSO*

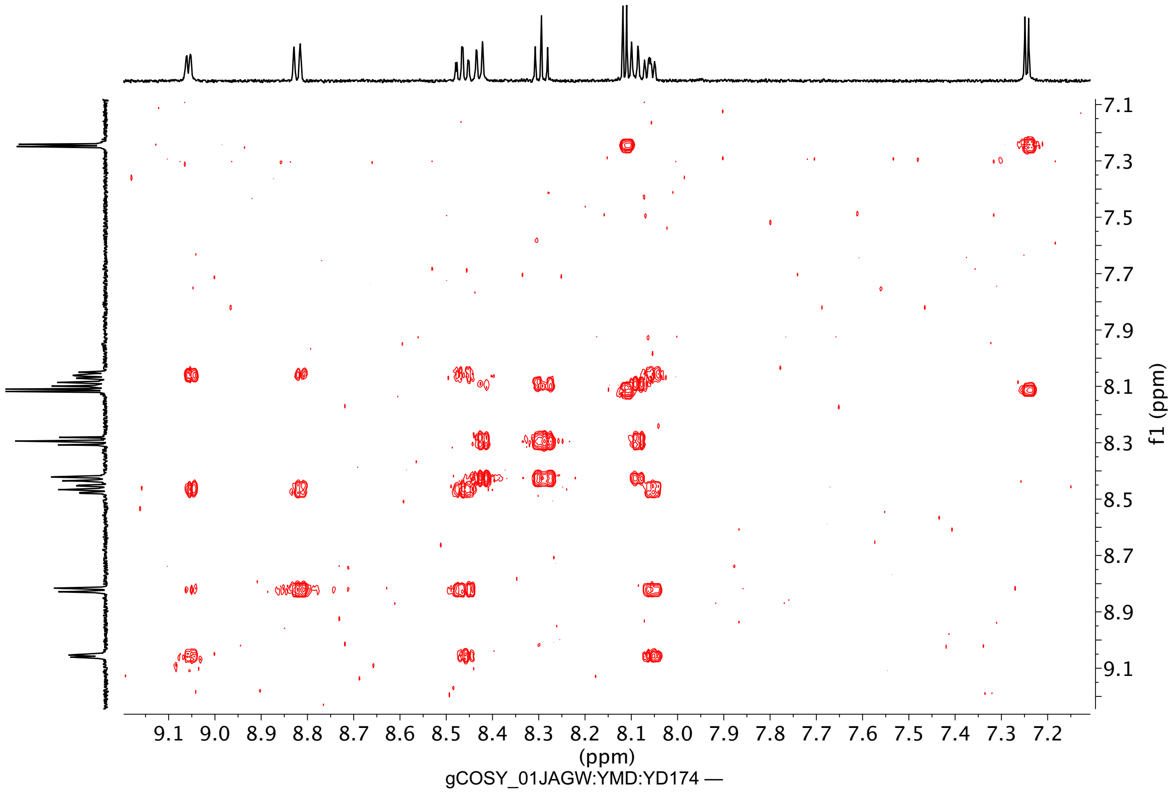


***Figure S4.65*** *COSY NMR spectrum of PtL[6]Cl$_3$ in d$_6$-DMSO*

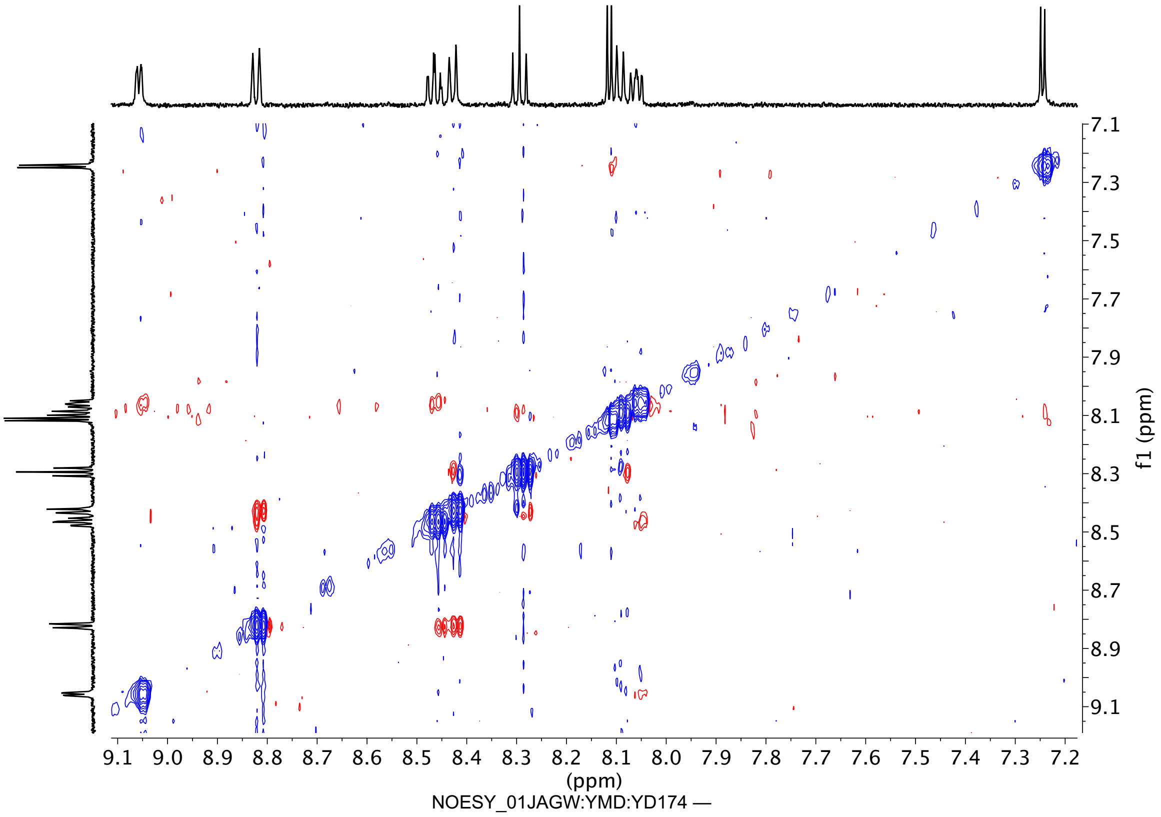


***Figure S4.66*** *NOESY NMR spectrum of $PtL^6Cl_3$ in $d_6$-DMSO*

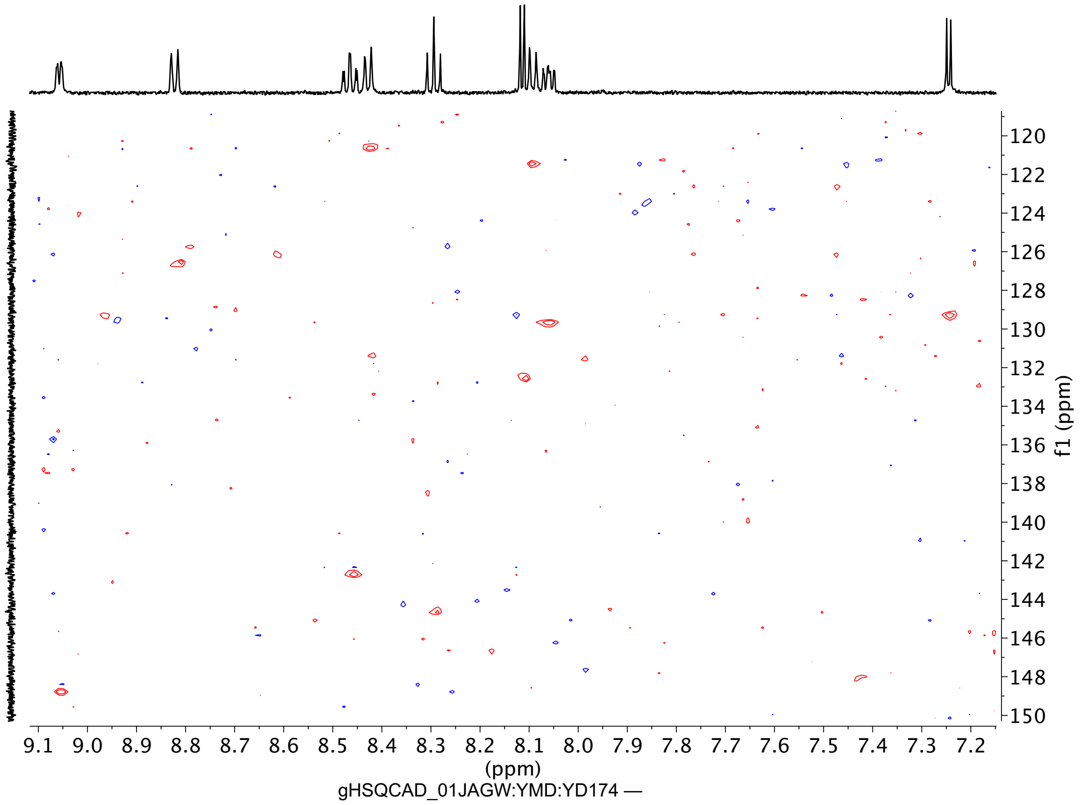


***Figure S4.67*** *HSQC NMR spectrum of $PtL^6Cl_3$ in $d_6$-DMSO*

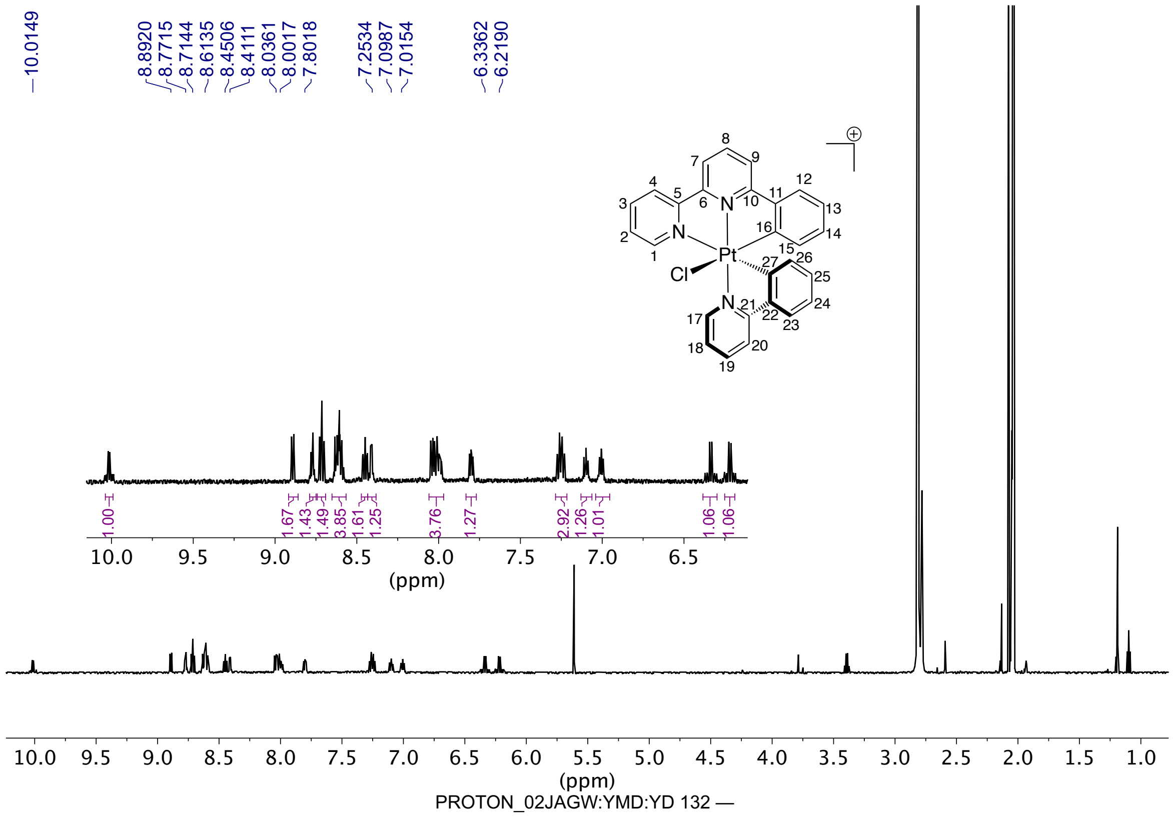

***Figure S4.68*** *$^{1}$H NMR spectrum of [PtL[1](ppy)Cl]PF₆ in d₆-acetone*

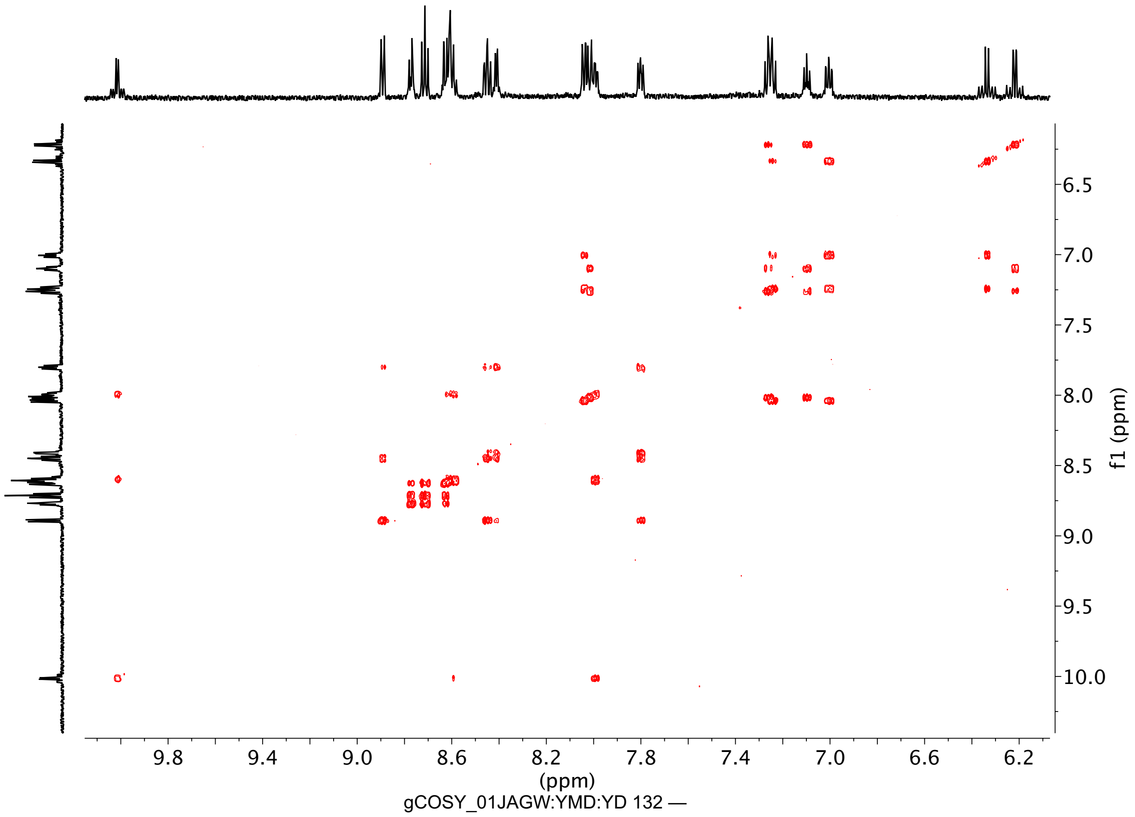

***Figure S4.69*** *COSY NMR spectrum of [PtL[1](ppy)Cl]PF₆ in d₆-acetone*

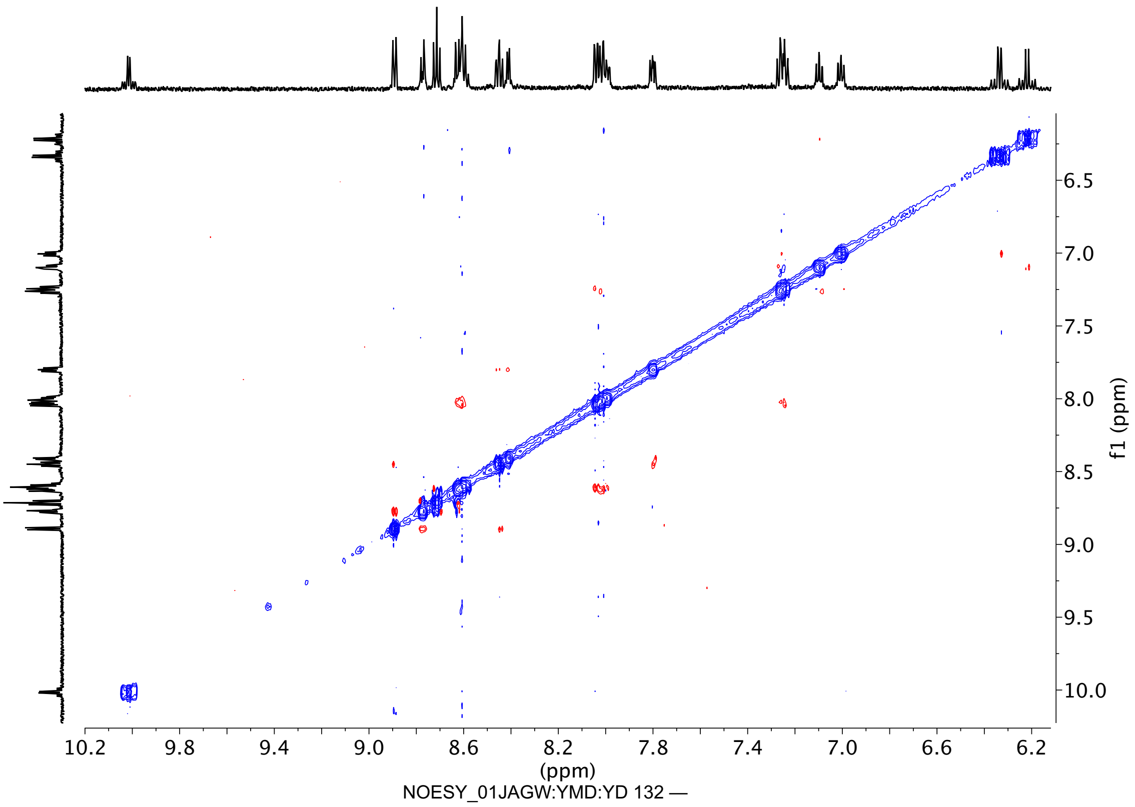


***Figure S4.70*** *NOESY NMR spectrum of [PtL[1](ppy)Cl]PF$_6$ in d$_6$-acetone*

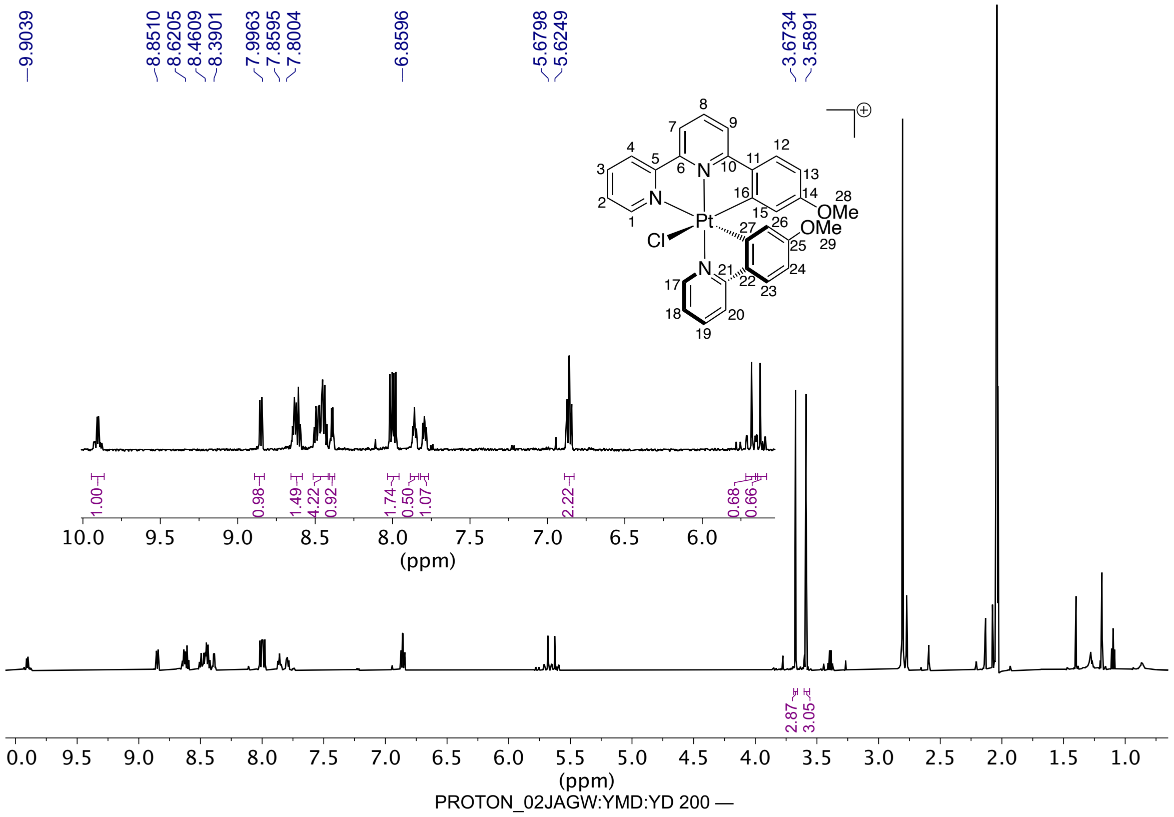


***Figure S4.71*** *$^{1}$H NMR spectrum of [PtL[4](4-MeOppy)Cl]PF$_6$ in d$_6$-acetone*

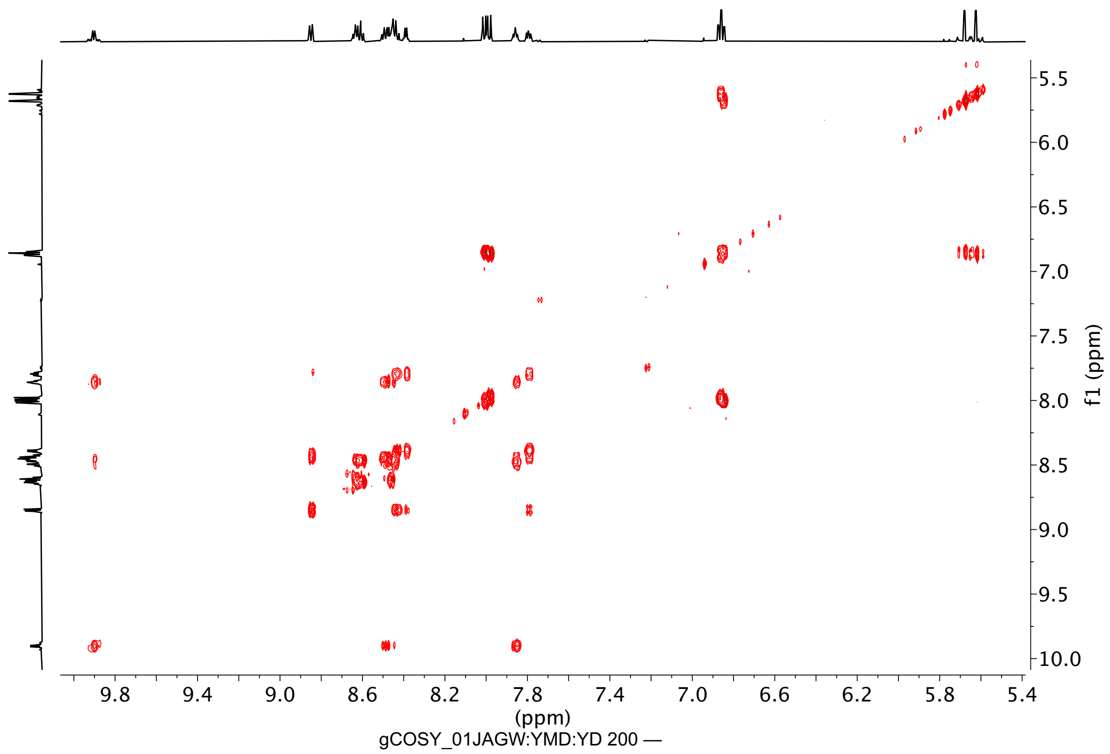


***Figure S4.72*** *COSY NMR spectrum of [PtL*[4]*(4-MeOppy)Cl]PF*$_6$ *in d*$_6$*-acetone*

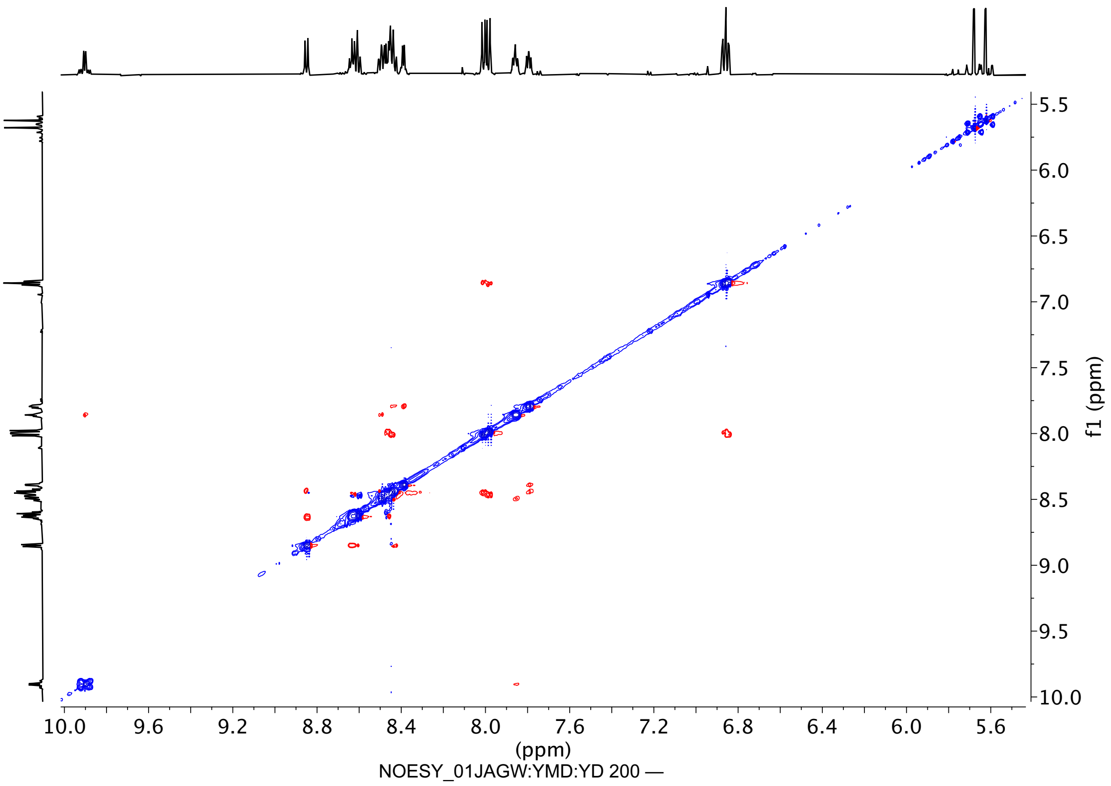


***Figure S4.73*** *NOESY NMR spectrum of [PtL*[4]*(4-MeOppy)Cl]PF*$_6$ *in d*$_6$*-acetone*

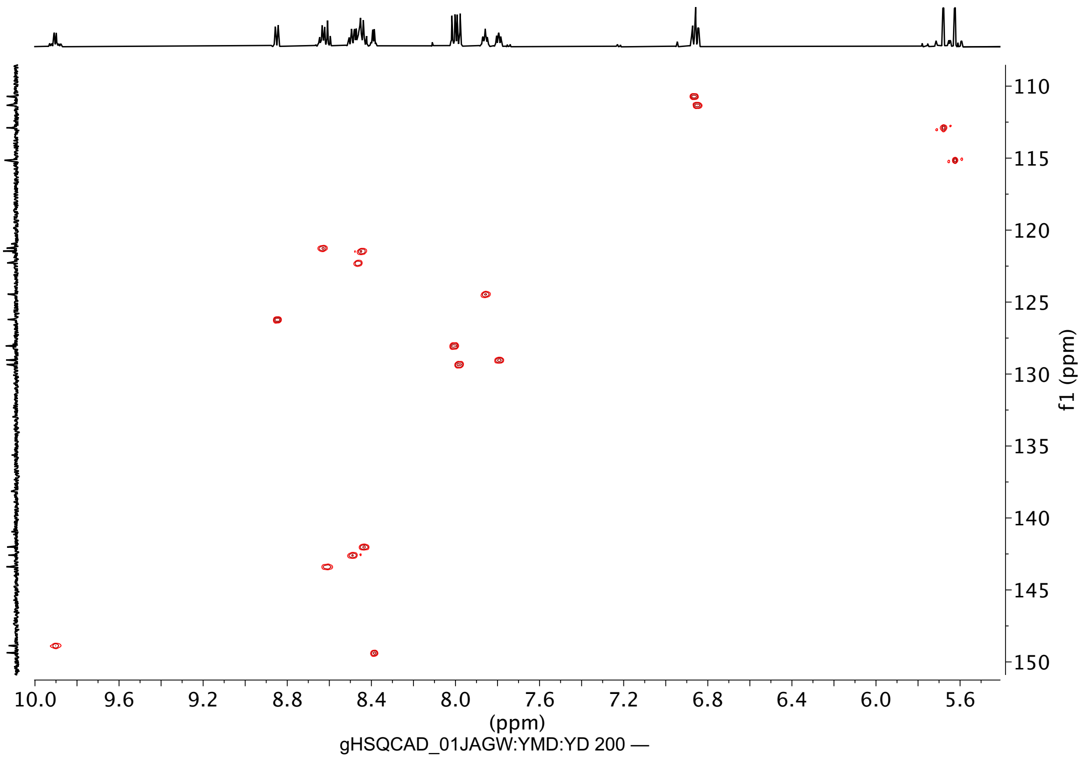


***Figure S4.74*** *HSQC NMR spectrum of [PtL$^4$(4-MeOppy)Cl]PF$_6$ in d$_6$-acetone*

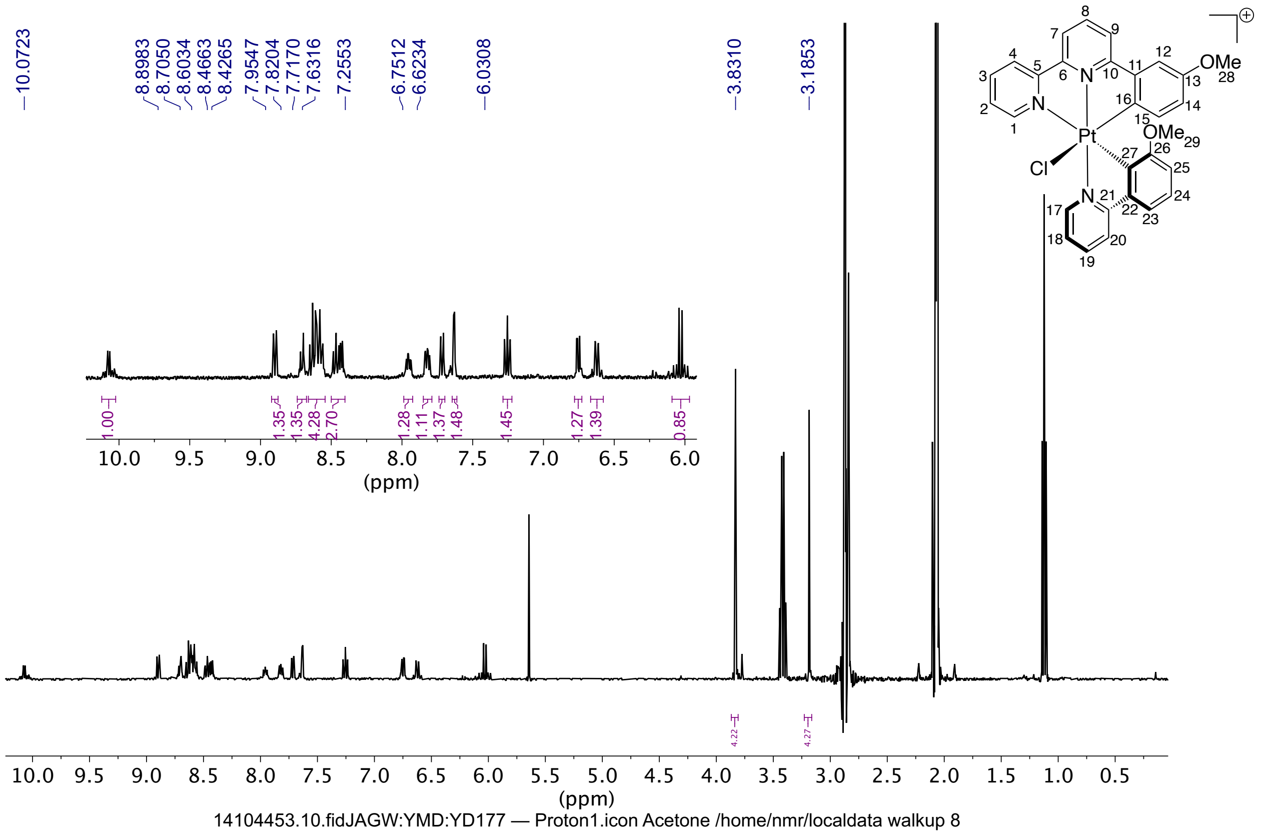


***Figure S4.75*** *$^1$H NMR spectrum of [PtL$^5$(3-MeOppy)Cl]PF$_6$ in d$_6$-acetone*

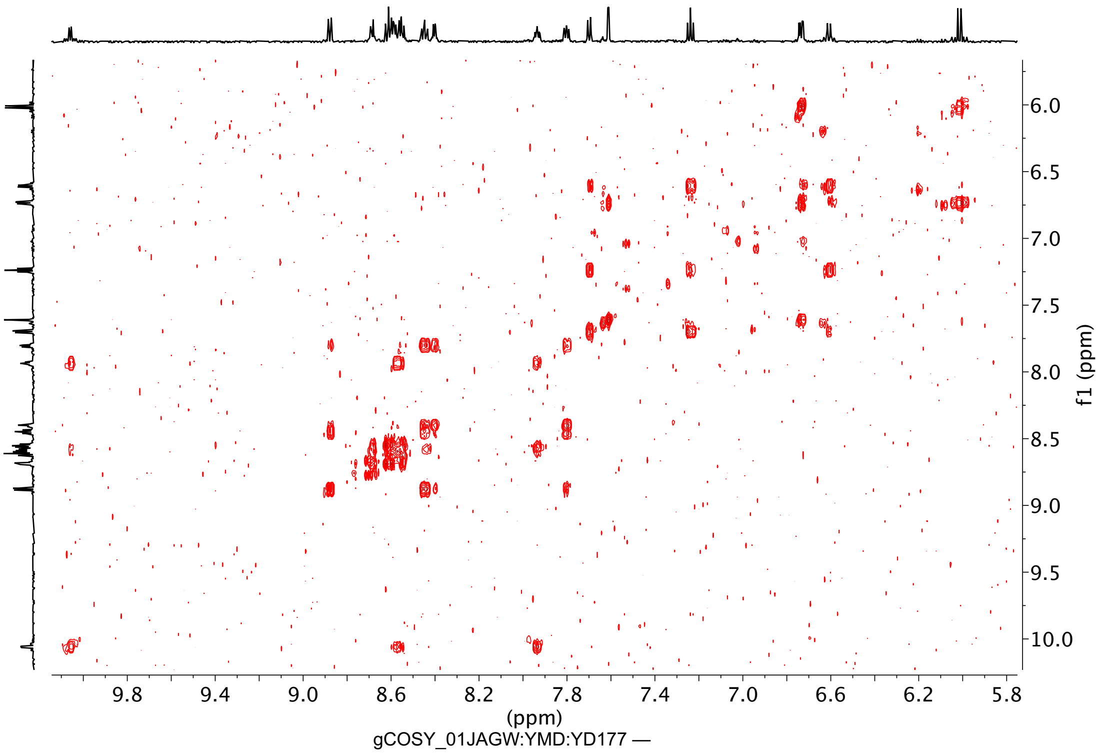


***Figure S4.76*** *COSY NMR spectrum of [PtL$^5$(3-MeOppy)Cl]PF$_6$ in d$_6$-acetone*

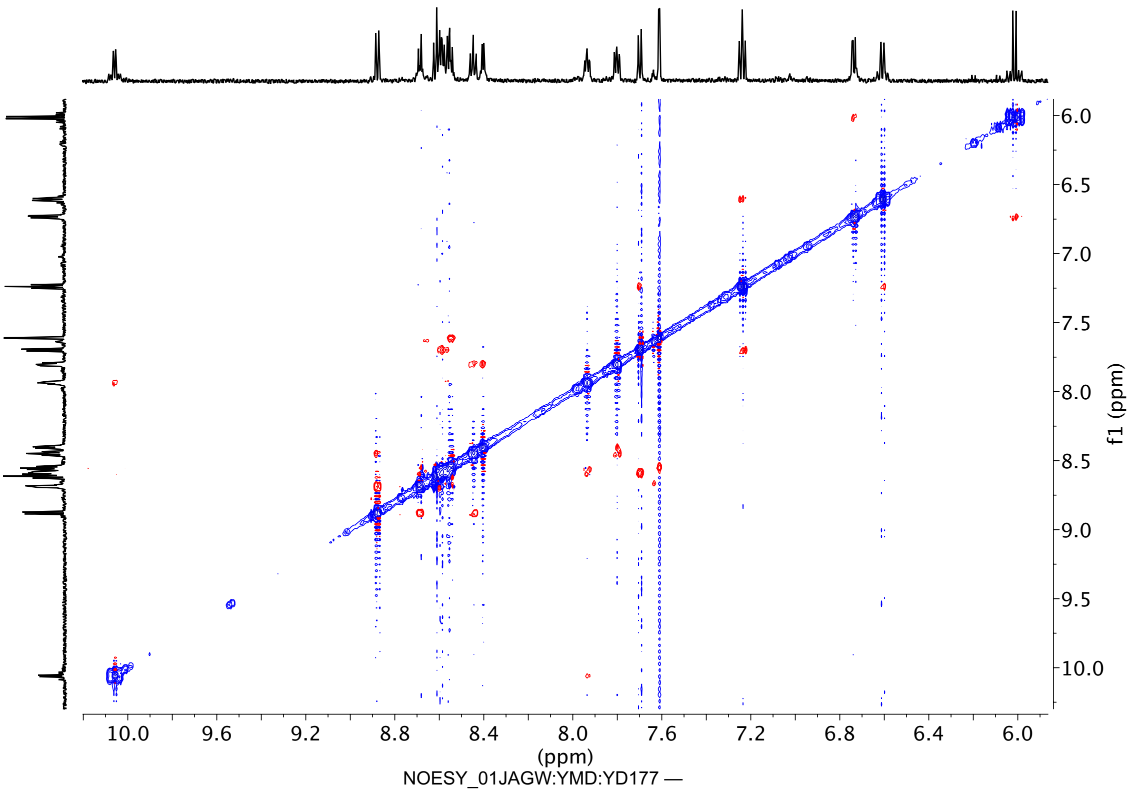


***Figure S4.77*** *NOESY NMR spectrum of [PtL$^5$(3-MeOppy)Cl]PF$_6$ in d$_6$-acetone*

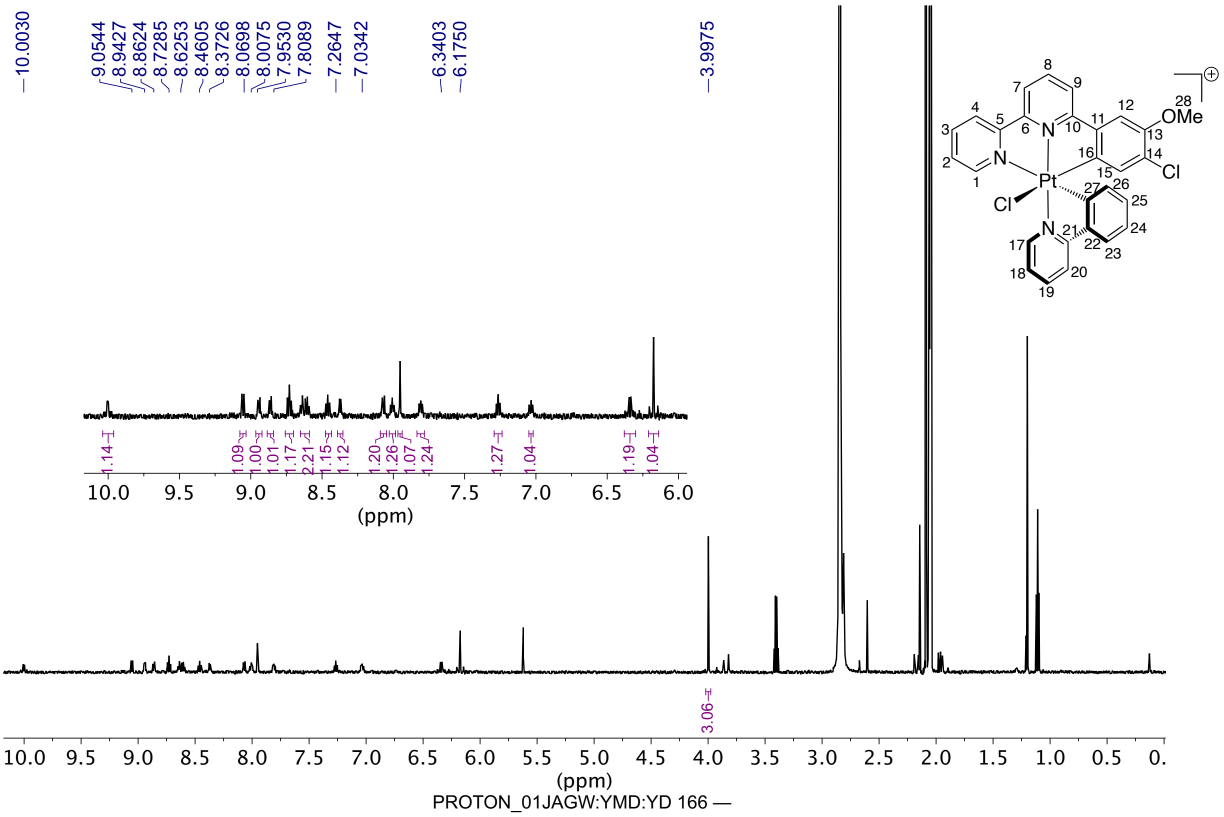


***Figure S4.78*** *$^{1}$H NMR spectrum of [PtL$^{5Cl}$(ppy)Cl]PF$_6$ in d$_6$-acetone*

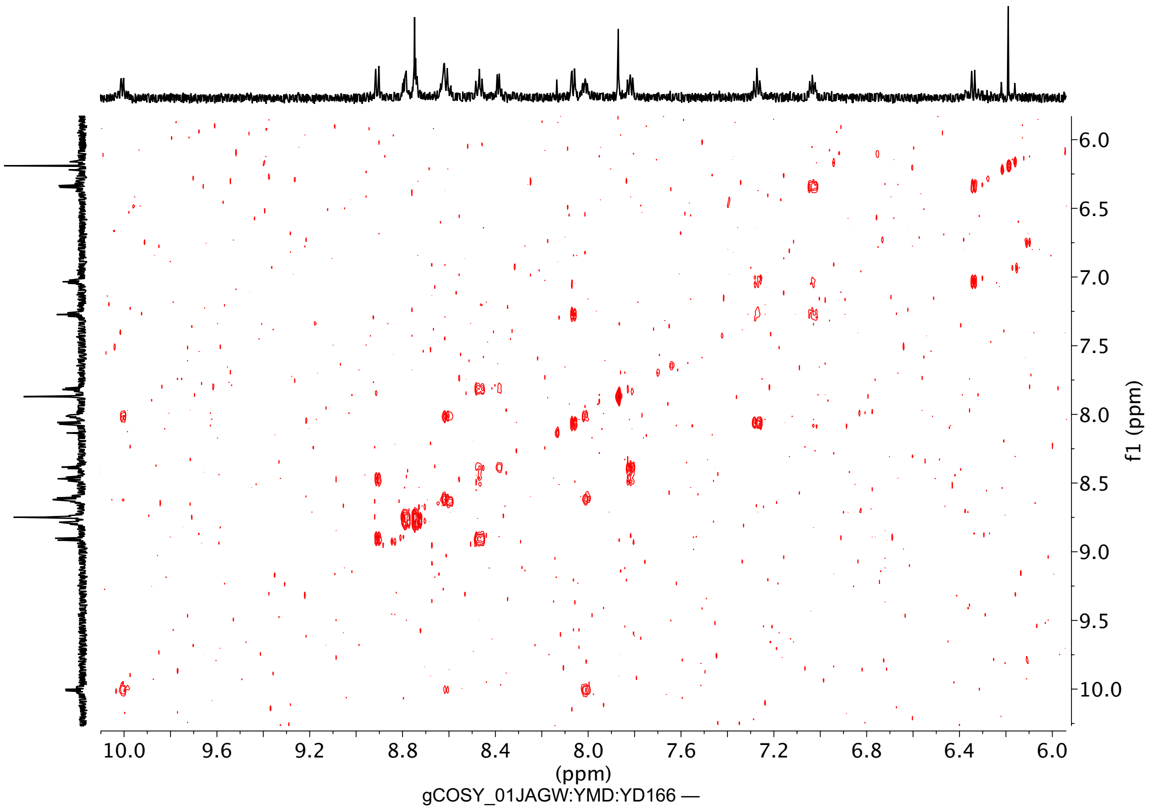


***Figure S4.79*** *COSY NMR spectrum of [PtL$^{5Cl}$(ppy)Cl]PF$_6$ in d$_6$-acetone*

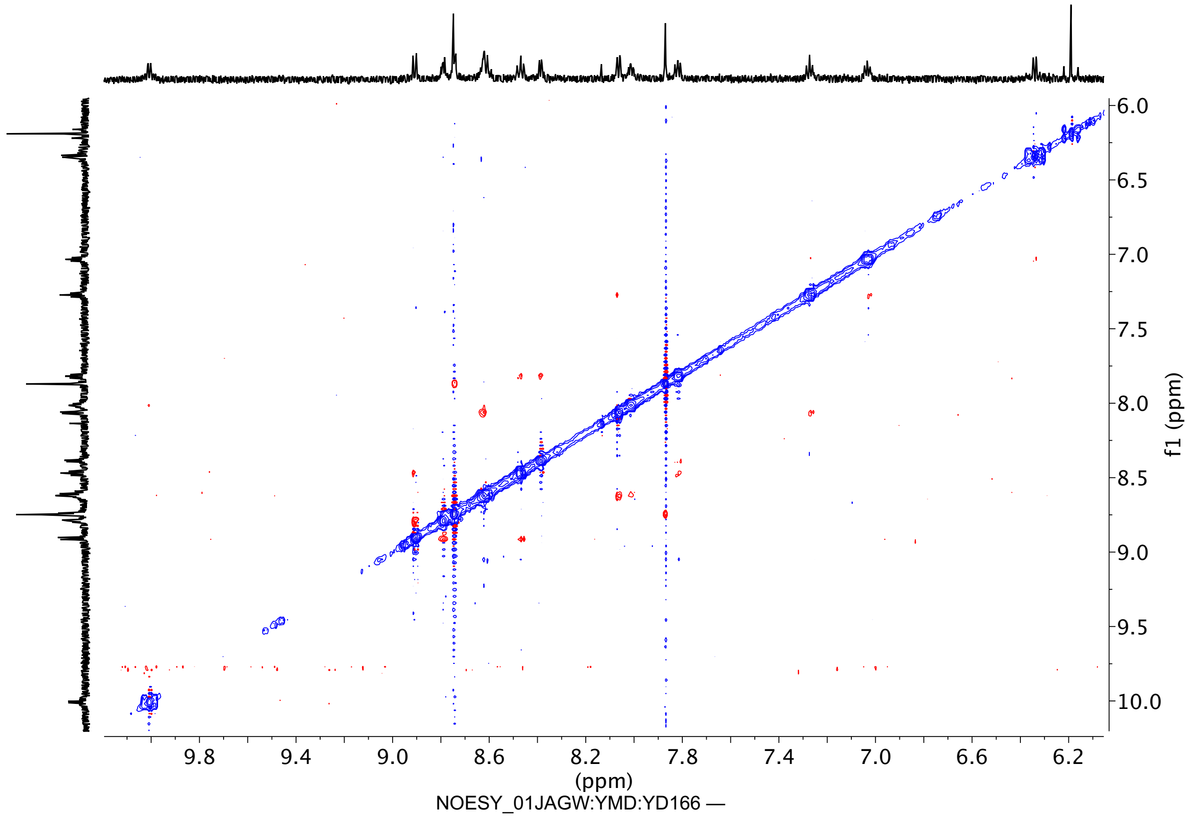


***Figure S4.80*** *NOESY NMR spectrum of [PtL*$^{5Cl}$*(ppy)Cl]PF*$_6$ *in d*$_6$*-acetone*

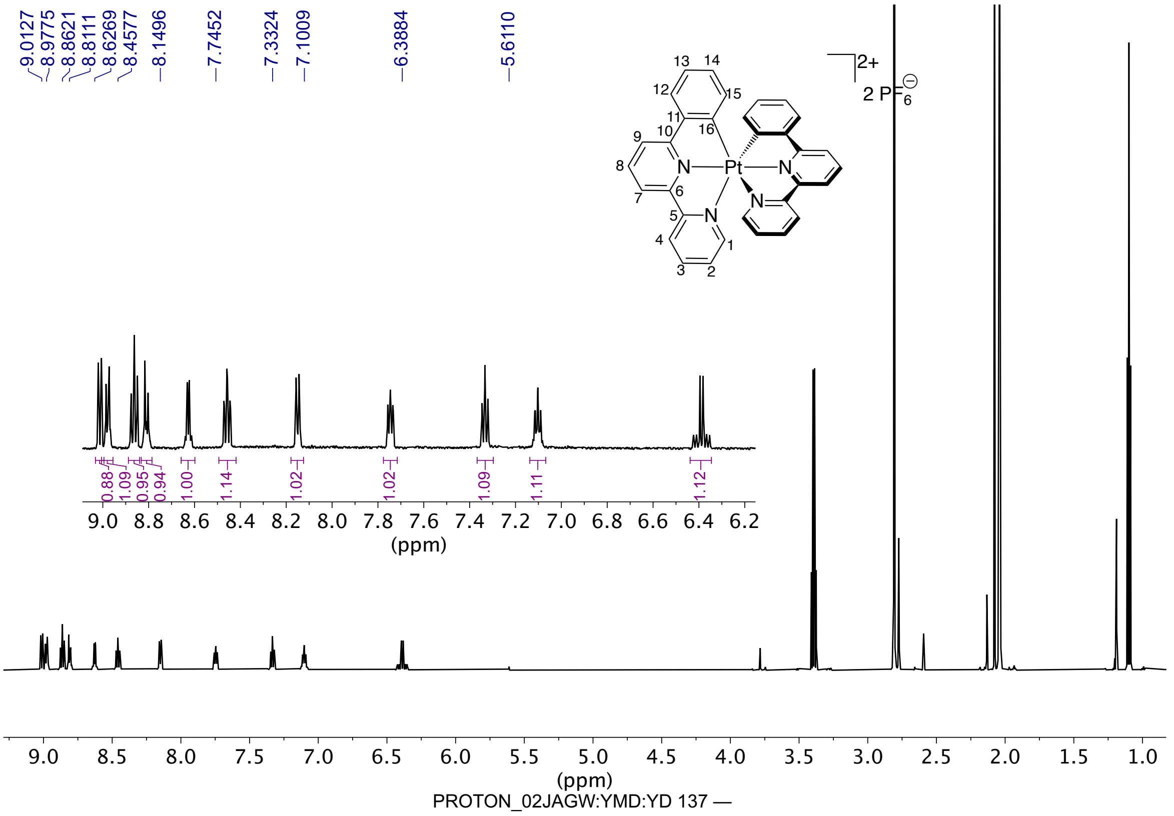


***Figure S4.81*** $^1$*H NMR spectrum of [Pt(L*$^1$*)*$_2$*][PF*$_6$*]*$_2$ *in d*$_6$*-acetone*

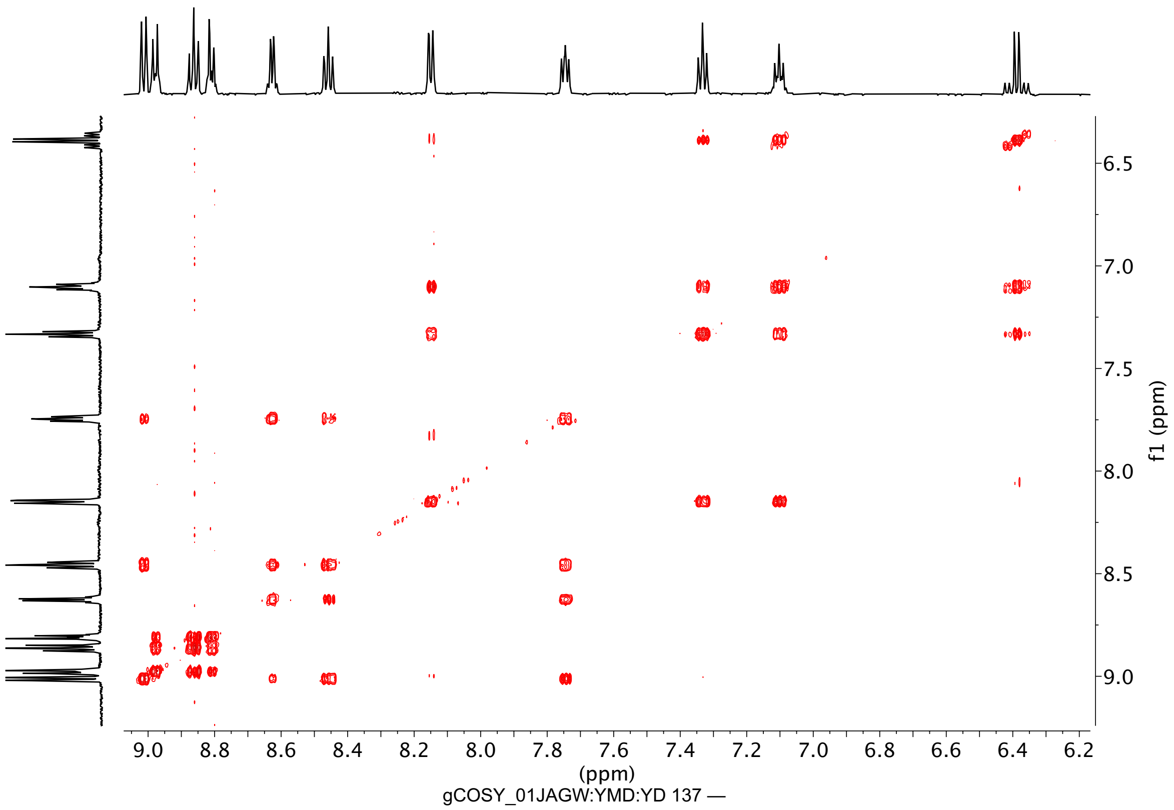


***Figure S4.82*** *COSY NMR spectrum of [Pt(L$^1$)$_2$][PF$_6$]$_2$ in d$_6$-acetone*

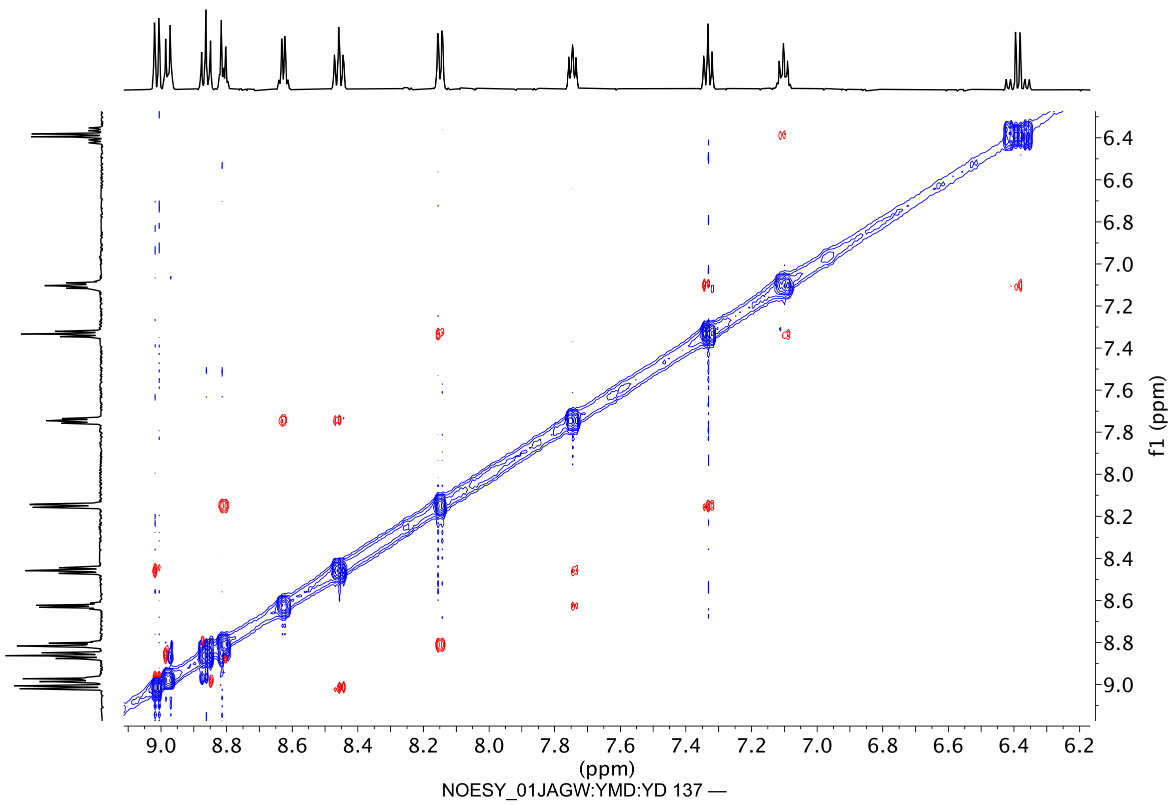


***Figure S4.83*** *NOESY NMR spectrum of [Pt(L$^1$)$_2$][PF$_6$]$_2$ in d$_6$-acetone*

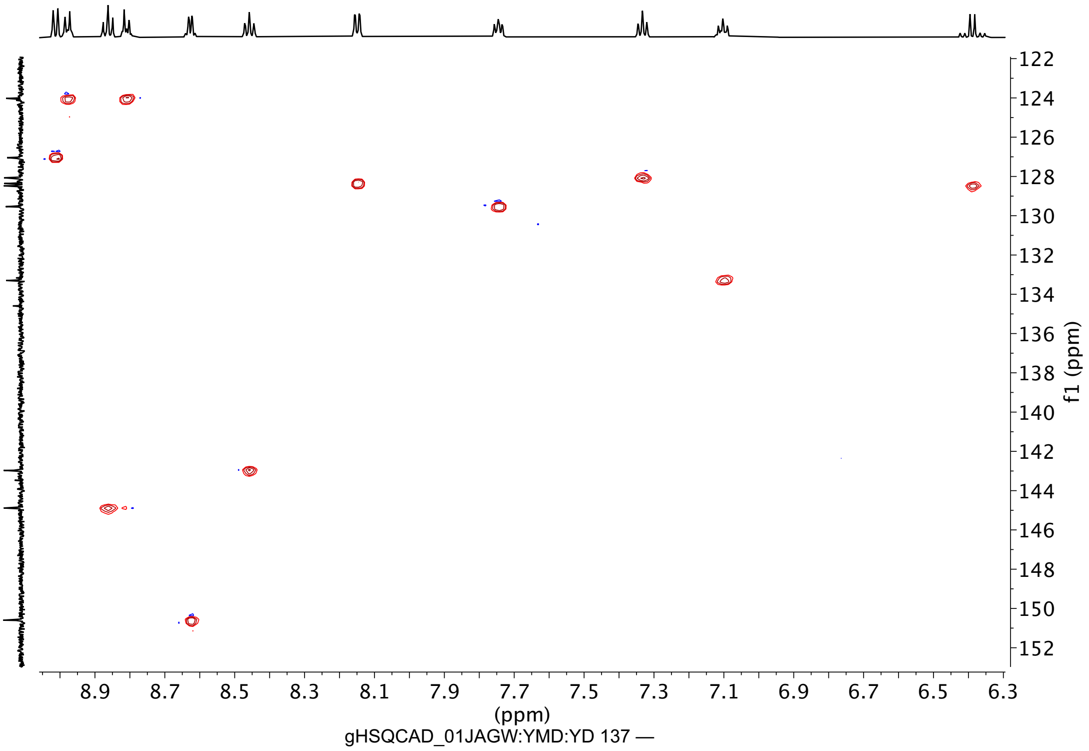


***Figure S4.84*** *HSQC NMR spectrum of [Pt(L*$^1$*)*$_2$*][PF*$_6$*]*$_2$ *in d*$_6$*-acetone*
